# Duplex DNA Retains the Conformational Features of Single Strands: Perspectives from MD Simulations and Quantum Chemical Computations

**DOI:** 10.3390/ijms232214452

**Published:** 2022-11-21

**Authors:** Amedeo Capobianco, Alessandro Landi, Andrea Peluso

**Affiliations:** Dipartimento di Chimica e Biologia “A. Zambelli”, Università di Salerno, Via Giovanni Paolo II, 132, I-84084 Fisciano, SA, Italy

**Keywords:** single-stranded DNA, DNA, DNA secondary structure

## Abstract

Molecular dynamics simulations and geometry optimizations carried out at the quantum level as well as by quantum mechanical/molecular mechanics methods predict that short, single-stranded DNA oligonucleotides adopt conformations very similar to those observed in crystallographic double-stranded B-DNA, with rise coordinates close to ≈3.3 Å. In agreement with the experimental evidence, the computational results show that DNA single strands rich in adjacent purine nucleobases assume more regular arrangements than poly-thymine. The preliminary results suggest that single-stranded poly-cytosine DNA should also retain a substantial helical order in solution. A comparison of the structures of single and double helices confirms that the B-DNA motif is a favorable arrangement also for single strands. Indeed, the optimal geometry of the complementary single helices is changed to a very small extent in the formation of the duplex.

## 1. Introduction

Although genomic DNA in the cell is assembled in double helices, single-stranded (ss) DNA is essential for many biological functions. Indeed, the double helix undergoes melting, which gives rise to segments of single-stranded DNA in important processes such as DNA replication, interaction with ss-DNA-binding-proteins and repair [1]. Furthermore, ss-DNA sequences are found in telomeres [2] and in ss-DNA viruses [3] and are often implicated in genetic damage, with ss-DNA being more exposed than ds-DNA to lesions and mutagenesis [4]. DNA has also been employed in nanostructures [5,6], the properties of which crucially depend on the conformations assumed by single helices [7]. Nonetheless, structural information on single strands is rather limited. Due to their conformational liability, single-stranded nucleic acids are difficult to study using standard X-ray diffraction techniques. Furthermore, the larger flexibility of single helices with respect to ds-DNA gives rise to large conformational heterogeneity in solution that is often very difficult to disentangle [8].

Experimental evidence accumulated over recent years has revealed that single strands rich in consecutive guanines (G) tend to form quadruplex arrangements in solution, especially with high concentrations of monovalent ions [9]. Moreover, stacking interactions between purine nucleobases, in particular, adenine (A), are far more effective than those acting between thymines (T) [10,11], so that single strands rich in purines exhibit a more regular coiling than polypyrimidines [8]. Finally, several short ss-DNA were found to undergo sequence-dependent thermal transitions, suggesting that smaller oligomers can also assume nonrandom conformations in solution [12].

The secondary structure assumed by ss-DNA and RNA depends on the specific nucleobase sequence, including possible defects, and is the result of several concomitant factors: conformational constraints imposed by the sugar-phosphate backbone, non-canonical inter-base hydrogen bonds, intra-strand stacking interactions, solvation, and ionic strength [13]. All those effects are hard to model both at the classic and the quantum levels [14,15,16]. Molecular dynamics (MD) stands as a valuable source of structural information for nucleic acids because it includes solvation at the atomistic level as well as thermal effects and allows for extensive conformational sampling. Indeed, continuous effort has been taken in the last decade, aimed at improving or developing increasingly reliable force fields for nucleic acids [15,17,18,19,20,21]. Unfortunately, MD simulations on medium-sized single strands are scattered here and there [21,22,23,24], and several studies on ss-DNA and RNA sequences are mainly aimed at testing the efficiency of algorithms and the quality of force fields employed in simulations rather than at delivering structural information [25,26,27,28]. In addition, the molecular mechanics (MM) on which MD is based cannot capture the polarization of DNA constituents resulting from the intra- and inter-molecular interactions; thus, quantum mechanical (QM) methods are required for proper modeling [14,29,30,31]. In this regard, the impressive increase in computational power in recent years has made quantum chemical computations accessible for large and meaningful fragments of nucleic acids [29,32,33]. Previous studies [14,16,34,35] demonstrated that density functional theory (DFT) may be a reliable tool for predicting realistic geometries of DNA fragments on the condition that dispersion forces and solvation, at least in continuum approximation, are included in computations.

In a recent study, optimal geometries of hexameric single strands composed of an increasing number of adjacent adenines end capped by thymines were obtained by DFT computations including solvent effects [13]. Although a limited number of cases were explored, the results revealed that ss-oligomers rich in adjacent adenines assume stacked arrangements, in line with the observed regularity of adenine tracts, which are known to confer structural rigidity to both double- and single-stranded DNA [8,13,36,37,38,39]. In contrast, much less is known for single strands of different compositions, especially those containing consecutive pyrimidines, in particular, cytosines (C). In order to fill that gap, herein, we investigated the structures of the C${}_{6}$, A${}_{2}$C${}_{2}$A${}_{2}$, and T${}_{6}$ hexameric single strands by carrying out MD simulations and geometry optimizations. We resorted to hexameric oligonucleotides because sequences composed of six nucleobases cover more than half helix repeats per turn, for both A- and B-DNA [37].

Information on the secondary structure assumed by A${}_{2}$C${}_{2}$A${}_{2}$ and T${}_{6}$ is partially available. Molecular dynamics simulations carried out in previous work showed that representative conformations of ss-A${}_{2}$C${}_{2}$A${}_{2}$ consist of two interacting blocks of three well-stacked nucleobases (AAC) and that occasional disruptions in regular coiling were predicted to occur mainly in the -CC- central dinucleotide step. Furthermore, TT steps were found to assume rather disordered arrangements in T${}_{x}$A${}_{y}$T${}_{z}$ hexameric single strands, which, however, retained substantial helicity during the simulation [40]. Based on those results, herein, we used the calf-thymus B-DNA conformation as the starting arrangement for the geometry optimizations of ss-T${}_{6}$ and ss-A${}_{2}$C${}_{2}$A${}_{2}$ carried out at the DFT level.

As for the structure of ss-C${}_{6}$, no previous investigation is available to the best of our knowledge; therefore, we have carried out new molecular dynamics simulations. Although G-rich nucleic acid sequences are expected to form quadruplexes, we have also optimized the geometry of the ss-G${}_{6}$ oligonucleotide starting from B-DNA conformation, in order to compare its structural features with those assumed by -G${}_{n}$- tracts in ds-B-DNA and with the ones of the other investigated sequences. Finally, we have also considered ss-A${}_{6}$, for which, as anticipated, there is computational and experimental evidence that conformations preserving B-DNA helicity should be highly populated in solution [13].

Optimized geometries of single-stranded nucleotides have been compared with those obtained by DFT computations of ds-G${}_{4}$ and ds-A${}_{4}$ double-stranded B-DNA, with the aim of evaluating the extent to which the geometry assumed by the double helix is dictated by the preservation of the optimal coiling of the individual single strands. A comparison has also been performed with selected structures of ss- and ds- DNA poly(A) and poly(G) sequences resolved by X-rays or NMR measurements. Being mainly interested in poly(C), optimal geometries for ss-C${}_{4}$ have also been evaluated by QM/MM ONIOM (`Our own N-layered Integrated molecular Orbital and Molecular mechanics’) computations [41]. Solvent molecules as well as counterions are included at the atomistic level in ONIOM optimizations. That is worth noting, inasmuch as previous investigations have indicated the lack of explicit solvent molecules as the major limitation of QM approaches complemented with continuum solvent models for the treatment of nucleic acids [29,33]. Given the large computational cost of ONIOM computations, we limited our QM/MM investigation to ss-C${}_{4}$, in which conformations, at least for the central step, are still modulated by the presence of the flanking nucleobases, as in more elongated sequences [42].

## 2. Results and Discussion

### 2.1. MD Simulations

**Figure 1:**
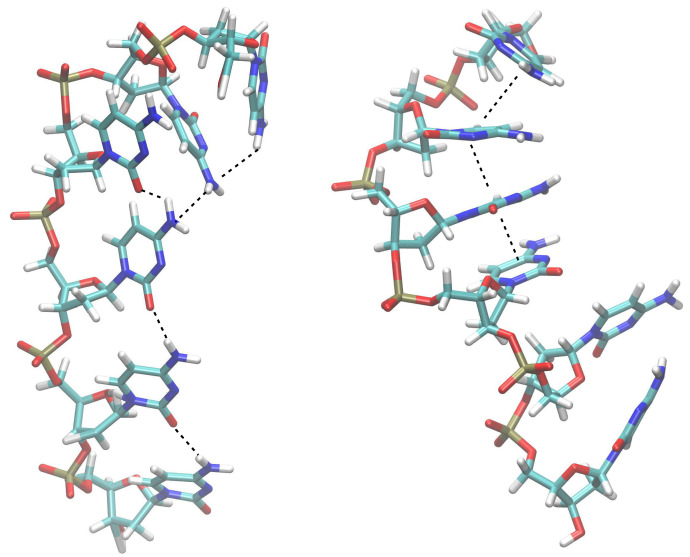
(**Left**) Irregular structure used as the starting geometry of ss-C${}_{6}$ in MD simulations; dashed lines denote hydrogen bonds. (**Right**) Snapshot of a representative structure of ss-C${}_{6}$ from MD trajectory; dashed lines indicate stacked nucleobases.

As a first step towards exploring the conformational complexity of ss-C${}_{6}$ in water, we started our MD simulations from the fully unstacked arrangement depicted in Figure 1. That ‘artificial’ structure was generated by optimizing the calf-thymus B-DNA ss-C${}_{6}$ sequence (as built by 3DNA) in the gas phase without including counterions and neglecting dispersion forces. That task was achieved by using the MMFF force field but with the attractive Lennard–Jones parameters disregarded, for the purpose of preventing the onset of stacking interactions among cytosines and forcing them to interact only via hydrogen bonds.

In order to quantify the extent of stacked configurations in C${}_{6}$ during the simulation, we adopted the criteria of Ref. [27], namely for each snapshot, two nucleobases are assumed to be stacked if (i) the distance between any pair of heavy atoms of the two bases is lower than 4 Å, (ii) the distance between the centers of mass of each base is lower than 5 Å, and (iii) the vector angle between the normals to the planes of two bases amounts to 0–45${}^{\circ }$ or 135–180${}^{\circ }$.

The occurrence of inter-base hydrogen bonds, possibly indicating a globular arrangement, was also checked. In our model, X⋯H–Y (X, Y heteroatoms of different nucleobases) form a hydrogen bond if (i) the distance between X and Y is lower than 3.3 Åand (ii) the angle *∠*XHY lies in the 150–180${}^{\circ }$ range.

MD simulations reveal that starting inter-base hydrogen bonds are quite readily disrupted (after ca. 15 ps). After that period, at most one inter-base hydrogen bond per snapshot is occasionally found. However, that hydrogen bond quickly disappears, never being detected in the subsequent snapshot. About 10${}^{4}$ hydrogen bonds have been located in $5\times 10^{4}$ snapshots, a very small number if one notes that at least 100 hydrogen bonds can form in ss-C${}_{6}$, considering only adjacent nucleobases.

Very rare occurrences of stacked pairs are found for non-adjacent nucleobases; instead, stacking between a couple of adjacent bases is a quite common event, found for ca. 60% of the simulation time for the first three CC steps, thus showing substantially ordered coiling for the first four cytosines in the $5^{\prime }\to 3^{\prime }$ direction. In contrast, the last CC step was found to be stacked only in 2% of snapshots.

In summary, the present MD simulations predict that ss-C${}_{6}$ assumes a quite regular structure, in which at least four out of six adjacent cytosines are well-stacked altogether. Interestingly, the present conclusions pair with the finding that the CCCC RNA oligomer in solution retains most of the regular helicity of standard ss-RNA, as indeed shown by the analysis of NMR spectra aided by MD simulations [43]. Therefore, based on these results, we have chosen the standard B-DNA conformation as the starting structure for both ss-C${}_{6}$ treated at the DFT level and ss-C${}_{4}$ investigated by ONIOM computations.

### 2.2. Results from DFT Geometry Optimizations

At first, we focused our attention on the rise coordinate (Figure 1), which quantifies the displacement along the helix axis of a nucleobase with respect to its predecessor. The prediction of reliable inter-base distances in DNA sequences is of great importance for the modeling of several important phenomena. As an example, it has been demonstrated that the rise and, to a lesser extent, the twisting of nucleobases, has a strong influence on the electronic coupling, the parameter regulating the hole transport (HT) in oxidized DNA [44,45,46,47,48,49,50,51,52,53], a phenomenon of enormous relevance for its biological consequences (mutagenesis, carcinogenesis, aging, etc.) [54,55,56] and also because DNA is an attractive material for applications in molecular electronics [57]. The achievement of an optimal rise coordinate for single strands is also essential for protein/DNA binding and recognition [58]. We remark that all the base step parametres can be found in Tables S4–S39 in the Supplementary Materials.

Apart from a few exceptions, predicted average rise coordinates (Table 1) are found to increase in passing from M06-2X to B3LYP-D3, confirming previous findings [13,34,59,60].

**Figure 2:**
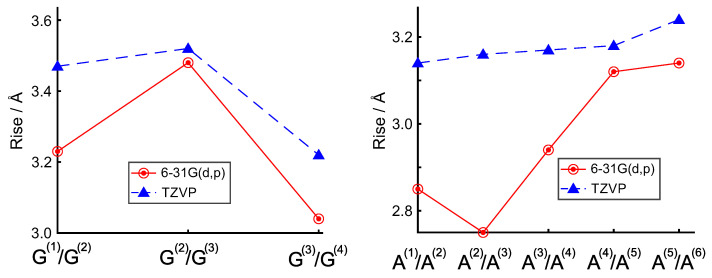
Predicted rise coordinates of ds-G${}_{4}$ ((**left**) B3LYP-D3) and ss-A${}_{6}$ ((**right**) M06-2X) using different basis sets.

Independent of the adopted functional, an increase in the rise coordinate is also found upon enlarging the size of the basis set (Figure 2). Although that effect is less pronounced for the duplexes, the conclusions of previous DFT investigations [13,29] are confirmed: A sufficiently extended basis set, such as TZVP or larger, should be used for geometry optimizations of DNA sequences; otherwise, the incompleteness of the basis set will be reflected in artificially shortened inter-base distances.

Indeed, for all single-stranded hexamers, the average rise predicted by computations with the larger basis set is in quite good agreement with the average value (3.3 Å) observed in crystallographic duplex B-DNA, especially for a B3LYP-D3 functional, in which estimates yield the smallest deviation in the rise coordinate with respect to crystallographic B-DNA.

A glance at Table 1 reveals that A${}_{6}$ exhibits the most regular rise, as expected, whereas ss-T${}_{6}$ is a notable exception. Indeed, independent of the adopted methodology, a very short average rise, never exceeding 2.7 Å is found for T${}_{6}$ by DFT optimizations, thus indicating poly(T) as the sequence more prone to give irregularities. Despite the smaller rise of ss-T${}_{6}$ with respect to ss-A${}_{6}$, the end-to-end distances, herein computed as the distance between the 5${}^{\prime }$ carbon of the first nucleotide and the 3${}^{\prime }$ carbon of the last nucleotide, are predicted to be 22.84 and 22.64 Å for T${}_{6}$ and A${}_{6}$, respectively, using B3LYP-D3/TZVP computations, with similar results obtained with the other methodologies. This is in line with the findings of Ref. [8] based on small-angle X-ray scattering (SAXS), which shows that ss-T${}_{40}$ and ss-A${}_{40}$ exhibit, on average, almost the same length in solution, even if the conformer distribution is consistent with a large prevalence of regularly stacked nucleobases only for poly(A) [8].

In agreement with the results of the MD simulation [40], the central CC step of the A${}_{2}$C${}_{2}$A${}_{2}$ sequence is predicted to be less efficiently stacked than AA steps by both the M06-2X and B3LYP-D3 functionals, even if substantially different equilibrium geometries are obtained by the tested functionals for A${}_{2}$C${}_{2}$A${}_{2}$. Indeed, a quite large rise coordinate, 3.52 Å, is predicted for the CC step at the M06-2X/TZVP level, as opposite to 2.94 Å obtained by B3LYP-D3/TZVP computations (see Tables S21 and S39 in the Supplementary Materials). The latter functional, however, finds a very large tilt, 7.77${}^{\circ }$, for the central CC step, yielding a very small overlap area for adjacent cytosines.

When the TZVP basis set is employed, both B3LYP-D3 and M06-2X functionals yield a quite regular average rise for ss-C${}_{6}$. However, irrespective of the adopted methodology, a rise larger than 4.0 Å is predicted for the terminal C${}^{\left(5\right)}$/C${}^{\left(6\right)}$-3${}^{\prime }$ step, which also has a large negative tilt (−13 and −8 degrees, according to B3LYP-D3 and M06-2X; see Tables S17 and S26 in the Supplementary Materials). Those data confirm the conclusions of the MD simulations exemplified in Figure 1, i.e., the ending CC step at the $3^{\prime }$ side assumes the most irregular conformation in C${}_{6}$.

A comparison of the average rises of ss-C${}_{6}$ and ss-G${}_{6}$ (Table 1) puts into evidence the dramatic effect of a basis set. Indeed, independent of the functional, the average rise is predicted to increase in going from C${}_{6}$ to G${}_{6}$ with the 6-31G(d,p) computations, whereas the opposite result is achieved by the more reliable TZVP basis set. Furthermore, both the M06-2X/TZVP and B3LYP-D3/TZVP predictions indicate that the average rise of ds-G${}_{4}$ is closer to that of ss-C${}_{6}$ than that of ss-G${}_{6}$. Indeed, the average rise of ss-C${}_{6}$ is very close to that of regular B-DNA. In addition, the DFT/TZVP results show that the average rise of ss-C${}_{4}$ is larger than that of ss-G${}_{4}$ and in the equilibrium geometry of ds-G${}_{4}$ (see Tables S17–S20 and S35–S38 in the Supplementary Materials). This implies that the main structural features of ss-poly(G) and ss-poly(C) are essentially retained in the formation of the duplex.

A comparison of the rise coordinates of the single strands composing ds-A${}_{4}$ and those of ss-T${}_{6}$ and ss-A${}_{6}$ reveals a very different scenario. Indeed, ss-A${}_{4}$, ds-A${}_{4}$, and ss-A${}_{4}$ in the duplex assume almost the same rise coordinates at variance with ss-T4, the rise of which is substantially increased in passing from the single strand to the double helix (see Tables S4–S7, S13–S16, S22–S25, and S31–S34 in the Supplementary Materials). Worth noting is that the geometrical changes associated with the formation of the duplex reflect the deformation energy of single strands. Indeed, rather low deformation energies, less than 1 kcal/mol, were predicted [14] for ss-G${}_{3}$, ss-C${}_{3}$, and ss-A${}_{3}$, upon duplex formation, whereas larger values, ≈1.8–2.4 kcal/mol, were found for ss-T${}_{3}$, thus indicating ss-poly(T) as the sequence undergoing the larger structural change upon duplex formation.

A final inspection of Table 1 also shows that the average rise and helical rise assume very similar values, differing by ±0.2 Å for all the investigated cases. That indicates that the mean plane of each nucleobase in single strands or the mean plane of each Watson and Crick base pair in double helices is almost normal to the DNA long axis, a distinctive characteristic of B-DNA; indeed, it is well known that other motifs, such as A-DNA exhibit larger differences (up to 0.6 Å) between rise and helical rise [37].

Predicted average twist coordinates (Table 2) are pretty close to 36${}^{\circ }$, the standard value of B-DNA, for almost all the cases, with some interesting differences. Poly(A) and poly(G), both as single strands or double helices, exhibit twist values quite larger (even by 10${}^{\circ }$) than the average value of B-DNA, in particular when the TZVP basis set is used. That outcome is in agreement with experimental facts. Indeed homo-purines steps are expected to exhibit larger twist than homo-pyrimidines due to the larger distortion caused by the electrostatic interactions among the purines functional groups [36,37]. Moreover, it has been ascertained that B-DNA exhibits rise values close to 36${}^{\circ }$ only in sequences longer than a complete helix turn (ten nucleobases for B-DNA), whereas a larger distribution can be expected for smaller DNA segments [37,61]. As an example, twist coordinates larger than 40${}^{\circ }$ were inferred for ss-GA${}_{4}$C by NMR spectra [38], in line with the prediction of the TZVP basis set.

Still, the values reported in Table 2 are in line with those of the B-DNA form, inasmuch as A-DNA spans a much smaller range [31${}^{\circ }$–35${}^{\circ }$]. The data in Table 2 also show that twist and helical twist assume similar average values, with both coordinates being found systematically larger than 36${}^{\circ }$. That is another strong indication that all the investigated oligomers, including single strands, assume conformations very compatible with the B-DNA motif in solution, although sensible deviations from the ideal structure are found.

At variance with rise, no clear trend when passing from B3LYP to M06-2X is apparent. Probably a larger number of sequences, possibly dodecamers or longer, should be investigated for a definitive assessment.

**Figure 3:**
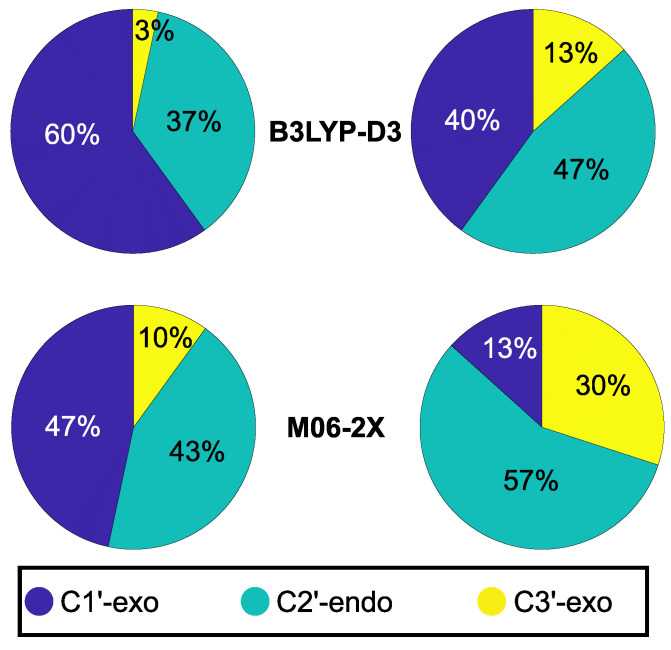
Distribution of the puckering conformations for the single-stranded hexamers.

Figure 3 shows that C1${}^{\prime }$-exo and C2${}^{\prime }$-endo—both conformations distinctive of B-DNA—are predicted to be the most populated puckerings for hexameric single strands by all methodologies, although a different behavior is exhibited by the different functionals. Computations predict a larger variety of populated conformations, including C3${}^{\prime }$-exo in passing from B3LYP-D to M06-2X or upon enlarging the size of the basis set. Nevertheless, all the predicted conformations of deoxyribose units are consistent with the B-DNA form, since the C3${}^{\prime }$-endo sugar conformation typical of A-DNA has not been detected in any of the investigated systems [37]. Identical conclusions are also reached for ds-A${}_{4}$ and ds-G${}_{4}$ duplexes (see the Supplementary Materials).

**Figure 4:**
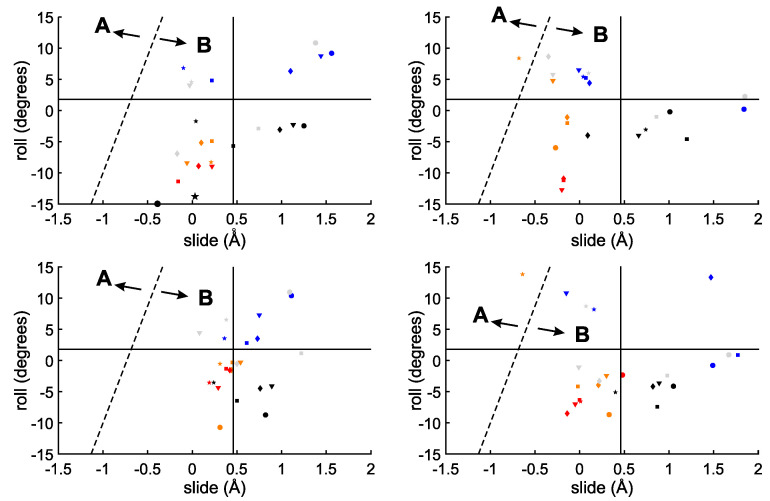
Predicted slide and roll coordinates of single-stranded hexamers. (**Top**): M06-2X; (**bottom**): B3LYP-D3. (**Left**): 6-31G(d,p); (**right**): TZVP. Blue: ss-A${}_{6}$; orange: ss-C${}_{6}$; black: ss-G${}_{6}$; red: ss-T${}_{6}$; gray: ss-A${}_{2}$C${}_{2}$A${}_{2}$. •: 5${}^{\prime }$-B${}^{\left(1\right)}$/B${}^{\left(2\right)}$ steps; ▾: B${}^{\left(2\right)}$/B${}^{\left(3\right)}$ steps; ⧫ B${}^{\left(3\right)}$/B${}^{\left(4\right)}$ steps; ▪: B${}^{\left(4\right)}$/B${}^{\left(5\right)}$ steps; and ★: B${}^{\left(5\right)}$/B${}^{\left(6\right)}$-3${}^{\prime }$ steps. The average slide and roll of the starting calf-thymus B-DNA conformation are reported as full lines. The dashed line separates the A- and B-DNA forms [62].

The similarity of the investigated single strands to the B-DNA conformation can also be inferred by the distribution of the roll and slide parameters, reported in Figure 4. Although with significant deviations with respect to calf-thymus B-DNA conformation (full lines), all the points in Figure 4 fall in the region of B-DNA, except for the terminal CC-3${}^{\prime }$ step of ss-C${}_{6}$ (orange starred points). This provides further confirmation that the larger deviation in ss-C${}_{6}$ from regular structure involves the terminal cytosine units at the 3${}^{\prime }$ end. An inspection of Figure 4 also shows that the 6-31G(d,p) basis set leads to a narrower range of conformations than TZVP. This is particularly relevant for the B3LYP-D3 (bottom panels of Figure 4) functional, for which slide coordinates between 0.0 and 1.4 Å are predicted at the 6-31G(d,p) level, while slides from −1.0 up to 2.0 Å are found with the TZVP basis set. Similar trends were found for T${}_{x}$A${}_{y}$T${}_{z}$ hexameric ss-DNA oligomers [13].

**Figure 5:**
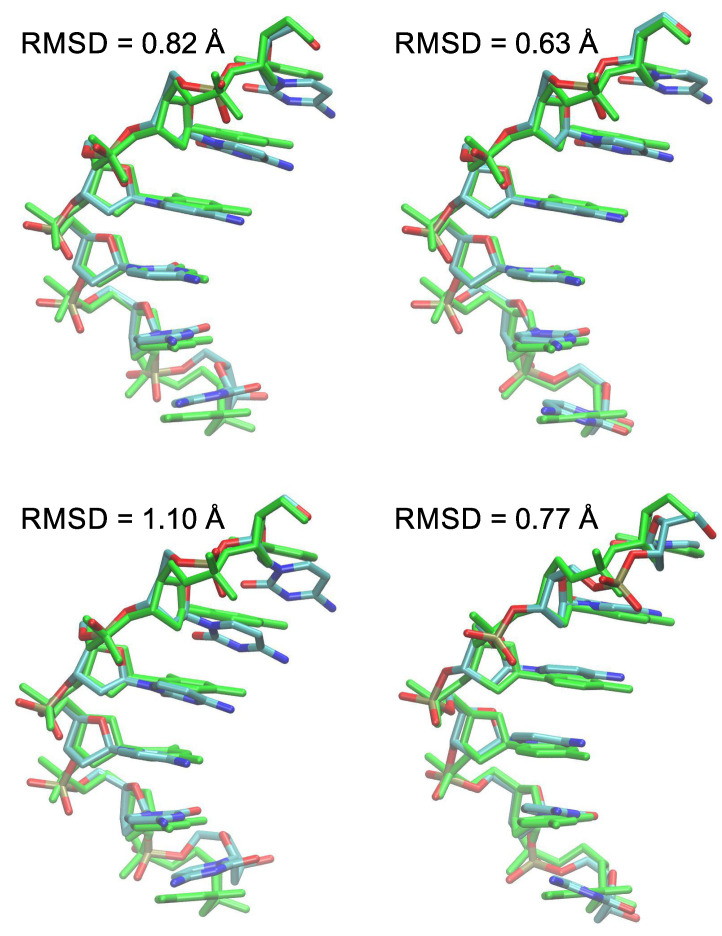
Equilibrium geometry of the ss-C${}_{6}$ single strand superimposed to the initial B-DNA geometry (green). (**Top left**) B3LYP-D3/6-31G(d,p); (**top right**) B3LYP-D3/TZVP; (**bottom left**) M06-2X/6-31G(d,p); and (**bottom right**) M06-2X/TZVP. The RMSD (Å) between superimposed structures is also reported.

The equilibrium geometries of ss-C${}_{6}$ obtained by the different DFT computations are depicted in Figure 5, where they have been superimposed onto the starting geometry, adopting calf-thymus B-DNA conformation. Apart from the large roll predicted by TZVP computations for the terminal CC-3${}^{\prime }$ step, it is seen that the optimal geometries of ss-C${}_{6}$ closely resemble the configuration of the standard B-DNA structure. Indeed, the predicted RMSD between starting and optimized geometry amount to 0.82 Å for B3LYP-D3/6-31G(d,p), 0.63 Å for for B3LYP-D3/TZVP, 1.10 Å for M06-2X/6-31G(d,p), and 0.77 Å for M06-2X/TZVP, thus confirming the previous conclusion that optimal structures predicted by the larger basis are more similar to the standard ds-B-DNA conformation.

The results of DFT computations for ss-C${}_{6}$ are somewhat paradigmatic inasmuch as the present analysis indicates that the deviations from the standard B-DNA structure most frequently found by MD simulations are also detected in the optimized geometries of short single-stranded sequences obtained by DFT computations using the standard B-DNA conformation as the starting structure.

**Figure 6:**
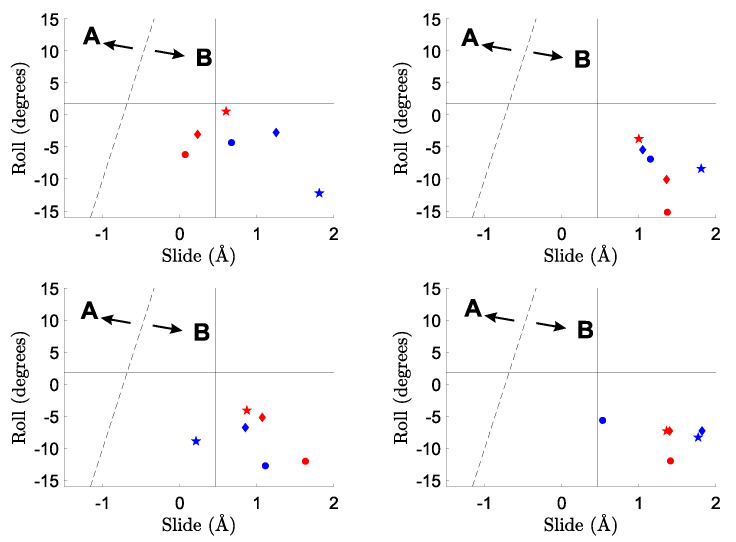
Predicted slide and roll coordinates of double-stranded tetramers. (**Top**): M06-2X; (**bottom**): B3LYP-D3. (**Left**): 6-31G(d,p); (**right**): TZVP. Blue: ds-G${}_{4}$; red: ds-A${}_{4}$. •: 5${}^{\prime }$-B${}^{\left(1\right)}$/B${}^{\left(2\right)}$ steps; ⧫ B${}^{\left(2\right)}$/B${}^{\left(3\right)}$ steps; and ★: B${}^{\left(3\right)}$/B${}^{\left(4\right)}$-3${}^{\prime }$ steps. The average slide and roll of the starting calf-thymus B-DNA conformation are reported as full lines. The dashed line separates the A- and B-DNA forms [62].

The slide–roll diagram for the investigated double helices is shown in Figure 6. At variance with the case of single strands (Figure 4), no substantial enlargement of slide and roll values is predicted upon increasing the size of the basis set. Instead, independent of the adopted methodology, the range of roll coordinates accessible to double helices is found to be much lower than that of single strands, clearly reflecting the constraints imposed by the pairing of complementary strands via hydrogen bonds.

### 2.3. ONIOM Results

According to the data in Table 3, independent of the adopted functional, very short average rise coordinates are found for ss-C${}_{4}$ by ONIOM computations employing the ME approach (see also Tables S40–S47 in the Supplementary Materials). Indeed the average rise predicted by the 6-31G(d,p) basis set never exceeds 2.5 Å. A significant increase, ≈0.25–0.35 Å, is found in passing to TZVP. However, very short rises, less than 2.8 Å, are still found even when the larger basis set is employed. The same also holds for the average twist, which is found to increase by ca. 3${}^{\circ }$ in passing from 6-31G(d,p) to TZVP. An overall analysis in terms of rigid step coordinates (Tables S41 and S45 in the Supplementary Materials) shows that severely disordered B-DNA conformations are found for ss-C${}_{4}$ with ME computations employing the 6-31G(d,p) basis set. ME predictions closely resemble those obtained with DFT/PCM computations (see above). In fact, both DFT/PCM computations in which solvation is included through a continuum model and ONIOM-ME computations, in which, although explicit solvent molecules are considered, the interaction between the DNA strand and the environment is treated by using a classic force field, seem to require sufficiently extended basis sets in order to achieve convergent results.

An inspection of Table 3 also shows that much more regular average twist (36.5–38.2${}^{\circ }$) and rise coordinates (3.0–3.2 Å) are found with ONIOM-EE computations, independently of the adopted functional and basis set. Furthermore, average twist is found to vary by less than 1${}^{\circ }$ in passing from 6-31G(d,p) to TZVP computations, and average rise is found to increase by less than 0.1 Å upon enlarging the size of the basis set.

**Figure 7:**
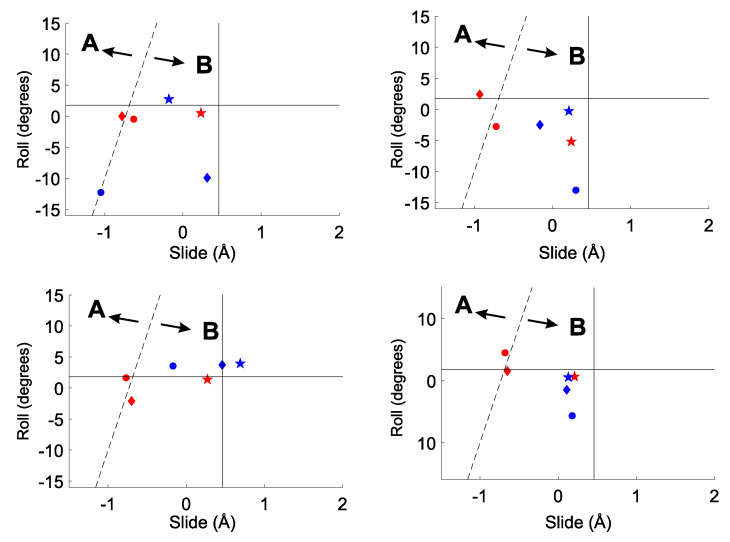
Predicted slide and roll coordinates of ss-C${}_{4}$. (**Top**): M06-2X; (**bottom**): B3LYP-D3. (**Left**): 6-31G(d,p); (**right**): TZVP. Blue: mechanical embedding; red: electrical embedding. •: 5${}^{\prime }$-C${}^{\left(1\right)}$/C${}^{\left(2\right)}$ steps; ⧫ C${}^{\left(2\right)}$/C${}^{\left(3\right)}$ steps; and ★: C${}^{\left(3\right)}$/C${}^{\left(4\right)}$-3${}^{\prime }$ steps. The average slide and roll coordinates of the starting calf-thymus B-DNA conformation are reported as full lines. The dashed line separates the A- and B-DNA forms [62].

The slide–roll diagrams obtained by ONIOM computations for ss-C${}_{4}$ are reported in Figure 7. The analysis of optimized slide/roll coordinates obtained by the ME method reveals strong similarities with the outcomes of DFT computations for single-stranded hexamers, summarized in Figure 4. Independent of the adopted functional, the slide coordinate is predicted to decrease in passing from 6-31G(d,p) to the TZVP basis set, with the latter basis set achieving more regular values. In line with the results of rise and twist, EE predictions appear to be scarcely dependent on the functional and basis set, and overall yield roll systematically close to zero, as in regular ds-B-DNA, at the expense of a larger slide. Indeed, the left panels of Figure 7, which refer to TZVP basis set, show that EE (red symbols) predictions give slide coordinates between −1 and +0.2 Å, whereas values close to zero are obtained with the ME method (blue symbols).

A summary of ONIOM results for ss-C${}_{4}$ in terms of RMSDs between optimized geometries and the regular calf-thymus B-DNA starting conformation is presented in Table 4.

As already inferred from the previous analysis of rise, twist, slide, and roll, Table 4 shows that the ONIOM-EE method (i) predicts equilibrium geometries closer to standard B-DNA conformation than ONIOM-ME and (ii) is far less sensible to the adopted basis than ONIOM-ME, with the latter method giving structures more similar to standard B-DNA upon increasing the size of the basis set. Point (i) is further confirmed by an inspection of Tables S40, S42, S44, and S46 in the Supplementary Materials, which reveals that very regular conformations, even more similar to the standard B-DNA motif than those obtained by DFT/PCM predictions for single-stranded hexamers, are found for ss-C${}_{4}$ with ONIOM computations adopting the electronic embedding. That conclusion is in line with the results of earlier studies in which it was argued that DFT optimizations using the continuum solvent approximation may give rise to sizable deviations from the known ranges of conformations of nucleic acids [29,33]. Instead, the inclusion of explicit water molecules, arranged as in an X-ray determined structure, in DFT computations was found to prevent the formation of non-native conformations [33]. However, the scarcer structural regularity of ss-C${}_{4}$ obtained by present ME computations may also depend on the adopted partition scheme. Indeed, the inclusion of the whole DNA strand in the QM layer in ME computations may well enhance the flexibility of the sugar phosphate backbone, which, oppositely, is treated at the MM level in EE computations. Although further studies are needed to achieve definitive conclusions, the present analysis reveals that ONIOM may give reliable predictions of the optimal geometries if small basis sets are also used in the QM layer, on the condition that electrostatic effects due to solvent polarization are treated at the quantum level in computations.

### 2.4. Comparison with Experimental Data

The present predictions indicate that, at variance with ss-poly(T), ss-poly(A) and poly(G) assume well-ordered structures in solution, which is in line with several pieces of experimental information. The application of elastic response and single molecule atomic-force spectroscopy carried out on poly(A) and poly(T) ss-DNA showed that, at least for sequences longer than 30 units, poly(A) adopts a stacked conformation, able to significantly enhance the polymer’s rigidity, as opposed to poly(T), in which stacking interactions are much weaker [10,11]. The electrophoretic mobilities of poly(T) ss-DNA sequences in solutions of high ionic strength were found to be consistent with the occurrence of rather elongated strands, not exhibiting any regular coiling [63]. Single-molecule Förster resonance energy transfer and SAXS experiments carried out for ss-T${}_{40}$ revealed that poly(T) lacks any secondary structure [64].

Voltammetric measurements showed that the lowering of the oxidation potential observed upon increasing the number of adjacent adenines or guanines in single-stranded DNA hexameric sequences is consistent with the presence of structured oligonucleotide conformations in solution, in which the nucleobases, especially adenines in adenine tracts, are well stacked altogether [35,40]. MD simulations and DFT geometry optimizations provided similar conclusions [13,40,65,66,67]. In line with the above results, Han et al. [68] recently showed that the staining efficiency of the SYBR Gold cyanine dye is particularly relevant for ss-DNA consisting only of adenine or A${}_{n}$/G${}_{m}$ alternate sequences because homopurine tracts confer helical order to the whole strand, which in turn, enables the binding of the dye to ss-DNA, thus ensuring efficient fluorescence emission.

The most relevant result of the present investigation is the prediction of a regular coiling for ss-poly(C). Although earlier theoretical investigations [23,24,26,69,70,71,72,73] indicated cytosine as the nucleobase more prone to give irregularities in single-stranded DNA and RNA short sequences, that conclusion was inferred mainly by the analysis of C/R and R/C steps, with R being a purine nucleobase. However, there are several indications that efficient stacking interactions can confer a regular coiling to ss-poly(C). Indeed, emi-protonated ss-poly(C) are known to assemble in highly regular quadruplexes which remain stable even at pH values higher than 7 [74]. Furthermore, hairpin DNA triplexes are formed by stacked ss-poly(C) in acidic conditions [75]. In addition, early X-ray fiber diffraction experiments demonstrated that RNA single-stranded polycytidylic acid is a right-handed helix similar to those observed in A-type DNA [76]. A regular helical arrangement for ss-C${}_{4}$ RNA was also found by NMR spectra, whereas disordered structures were detected for ss-U${}_{4}$, with U being uracil [43].

The present results strongly suggest that several structural features of ds-B-DNA are already rooted in the corresponding single strands. To further assess that point, we analyzed a few experimentally determined structures of ds-B-DNA containing homo-base sequences in terms of the rigid coordinates of the individual strands. Structural data were retrieved from the RCSB protein data bank [77]. The average rises for the TT, CC, and AA steps are found to be 2.9, 3.6, and 3.2 Å, respectively, in ds-5${}^{\prime }$-CTG${}_{4}$ACT${}_{3}$C${}_{2}$AG${}_{2}$-3${}^{\prime }$, the structure of which was resolved by NMR and is stored in the *1kbd* PDB file [78] (Table S1 in the Supplementary Materials). Those data are in excellent agreement with the theoretical predictions of Table 1, which show an increase in the average rise in passing from poly(T) to poly(A) and from poly(A) to poly(C). Furthermore (Table S1 in the Supplementary Materials), in ds-5${}^{\prime }$-CTG${}_{4}$ACT${}_{3}$C${}_{2}$AG${}_{2}$-3${}^{\prime }$, the largest values for the twist angles are detected in correspondence with the A/A and A/G steps, again in agreement with the theoretical prediction that homo-purine sequences give rise to comparatively large twist angles (see Table 2). For poly(A), structural information is available, e.g., for the ds-5${}^{\prime }$-CGCGA${}_{6}$CG-3${}^{\prime }$ sequence, in which the structure was resolved by X-ray diffraction and is stored in the *1d89* PDB file [79], and for ds-5${}^{\prime }$-G${}_{2}$CA${}_{6}$CG${}_{2}$-3${}^{\prime }$, in which the NMR-resolved structure is stored in the *1fzx* PDB [80]. For both DNA sequences, the analysis of rigid coordinates (Tables S2 and S3 of the Supplementary Materials) reveals an average rise of 3.2 Å for AA steps, whereas a rise of ≲3 Å is detected for TT steps. Furthermore, twist values of ca. 40${}^{\circ }$ are assumed by the central AA steps in ds-5${}^{\prime }$-G${}_{2}$CA${}_{6}$CG${}_{2}$-3${}^{\prime }$, whereas consecutive thymine in central TT steps are twisted by ≈33${}^{\circ }$, again in line with theoretical predictions.

## 3. Methods and Materials

### 3.1. Starting Structures and DFT Geometry Optimizations

**Figure 8:**
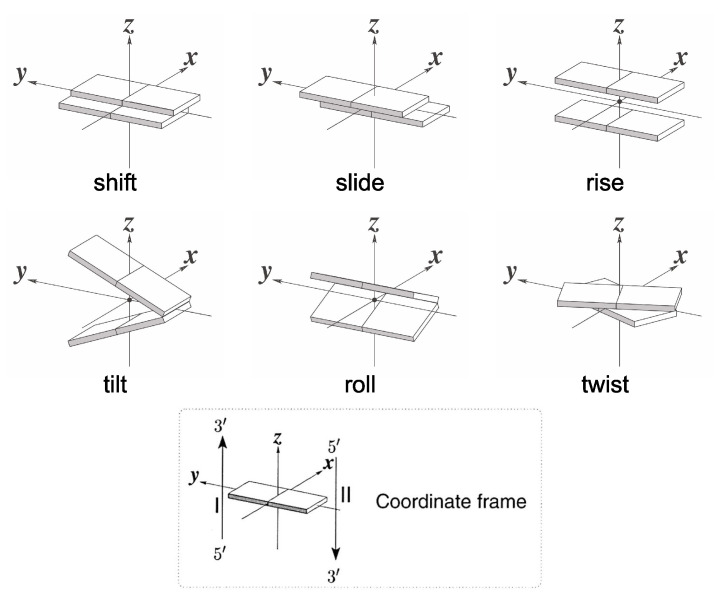
Schematic diagram of base-step rigid coordinates. For illustration purposes, helical twist is the same as twist and helical rise is the same as rise [81].

The starting geometries for all the investigated single- and double-stranded oligomers were built in B-DNA configuration by adopting the standard model based on the fiber diffraction of calf-thymus DNA. Herein, all the sequences are expressed in the $5^{\prime }\to 3^{\prime }$ direction. Final geometries were analyzed in terms of the standard rigid body coordinates of Figure 8 and torsion angles of sugar-phosphate backbone. We also considered helical twist, i.e., the angle of rotation about the helical axis that brings consecutive base pairs into coincidence and helical rise, i.e., the projection of the vector linking the geometrical centers of two consecutive base pairs (nucleobases for single strands) onto the helical axis. Please note that helical twist coincides with twists and helical rise coincides with a rise only for the null values of slide, roll, shift, and tilt [81]. The software 3DNA was used both to generate initial geometries and to analyze final structures [82]. In the following, the average rise, twist, helical rise, and helical twist are computed by averaging the values of the coordinates over all the dinucleotide steps composing a sequence.

DFT computations were performed with the Gaussian package [83], employing the M06-2X potential and the B3LYP functional in conjunction with the D3 (B3LYP-D3) parameterization for dispersion energy by Grimme, and adopting the damping function by Becke and Johnson [84,85,86,87]. Both the 6-31G(d,p) and the more extended TZVP basis sets were tested [88,89].

The solvent (water) effects were included in all DFT computations via the polarizable continuum model (PCM) [90].

Although it is known that both ss- and ds-DNA structures are affected by the presence of excess salt concentration or other biological crowders [91,92], herein, we have only considered DNA filaments in which the electrical neutrality is achieved. Therefore, we have neutralized the negative charge of phosphate units by adding five sodium ions for hexameric single strands and six sodium ions for tetrameric double strands [34]. Due to conformational liability, the very same starting structure (calf-thymus DNA) was utilized for the geometry optimizations carried out with different methodologies for each of the investigated systems.

### 3.2. MD Simulation of ss-C${}_{6}$

The starting configuration of ss-C${}_{6}$ was generated by means of an initial geometry optimization carried out using the MMFF force field in the gas phase (*vide infra*) via the Spartan package [93]. The optimized structure was formatted to be recognizable by the Amber 18 suite of programs [94]. Electrical neutrality was achieved by adding five Na${}^{+}$ ions via the `addions’ utility of Amber 18 and was immersed in a 10×10×10 Å${}^{3}$ box of explicit water molecules (ca. 3000 molecules), resulting in starting concentrations of ≈0.02 mol/dm${}^{3}$ in DNA and ≈0.1 mol/dm${}^{3}$ in sodium. Periodic boundary conditions were applied.

After a preliminary geometry optimization carried out by holding the DNA fixed, the system was equilibrated for 100 ps with a small harmonic restraint (10 kcal mol${}^{-1}$ Å${}^{-2}$) imposed on the strand, with the temperature being raised incrementally from 0 to 298 K. After equilibration, MD simulation was carried out for a production period of 0.05 μs with a sampling time of 2 fs. Snapshots were retrieved every 2 ps using the PTRAJ module of the Amber package. The AMBER OL15 force field, in conjunction with the Joung–Cheatham parameters for Na${}^{+}$ ions and the TIP3P model for water, was used in all simulations [19,95,96]. Short-range and electrostatic interactions were truncated at 12 Å. Longer-range electrostatic interactions were computed using the Particle Mesh Ewald method, with the same cutoff (12 Å) [97]. The SHAKE algorithm was used to constrain all bonds involving hydrogen atoms [98].

### 3.3. QM/MM ONIOM Computations

The starting geometry of ss-C${}_{4}$ was chosen by following the same criteria adopted in DFT optimizations: 3DNA software was used to generate the B-DNA calf-thymus conformation; the oligomer was electrically neutralized by adding three Na${}^{+}$ ions. A box of water molecules (≈12 Å radius) corresponding to ca. 2100 water molecules was added, yielding a starting concentration ≈0.02 mol/dm${}^{3}$ in ss-C${}_{4}$ and ≈0.06 mol/dm${}^{3}$ in Na${}^{+}$. A preliminary geometry optimization was carried with the AMBER software (OL15 + TIP3P force fields), in which the DNA chain was kept fixed by applying strong harmonic restraints (500 kcal mol${}^{-1}$ Å${}^{-2}$), just to minimize the positions of water molecules and counterions. Starting from MM results, final geometry optimizations were carried out at the ONIOM level by including in the computations the whole system, comprising the DNA chain, water molecules, and counterions. In all calculations, the MM layer was treated at the AMBER/TIP3P level, whereas the QM layer was treated at the DFT level. Two functionals, M06-2X and B3LYP-D3; two basis sets, 6-31G(d,p) and TZVP; and two different partition schemes (*vide infra*) were used for the QM layer, thus totaling eight geometry optimizations.

In the first partition scheme, the whole DNA sequence was included in the QM layer, while water molecules and Na${}^{+}$ counterions were left in the MM region; the mechanical embedding (ME), in which all the interactions between the QM and the MM regions are treated at the MM level, was used. In the second scheme, only DNA nucleobases (cytosines) were included in the QM region, whereas sugar-phosphate backbone atoms, water molecules, and Na${}^{+}$ counterions were left in the MM layer. Dangling bonds due to the breaking of the covalent bonds between the N1 atom of cytosine and the C1${}^{\prime }$ atom of deoxyribose were saturated by hydrogen [99], and the electronic embedding (EE) was employed. The latter method incorporates the effects of charges of the MM region into the quantum mechanical electron density, in such a way that the electrostatic interaction between the QM and MM regions is treated at the QM level, thus allowing for the polarization of the electron density by the environment.

## 4. Conclusions

MD simulations and DFT geometry optimizations carried out in conjunction with the continuum solvent approximation and QM/MM computations with explicit solvent molecules predict that the B-DNA motif typical of double helices is also representative of the conformations adopted by short, single-stranded DNA in solution, although significant deviations from ideal B-DNA are to be expected. In agreement with experimental evidence, poly(A) single strands are predicted to assume regular arrangements, whereas larger deviations from the ideal structure are found for poly(T) sequences. The present computational results point to a consistent structural regularity also for short, single-stranded poly(C) oligomers, as indeed already observed for ss-C${}_{4}$ in RNA. Furthermore, by comparing the optimal geometries of single-stranded sequences with the ones observed or predicted for the same single strands inside duplex DNA, it emerges that single strands undergo small geometry deformations in forming the double helix, thus confirming that the B-DNA coiling is a favorable arrangement also for single strands because it ensures good compromise between optimal base-stacking arrangements and the restraints imposed by the sugar-backbone conformation.

As for the tested methodologies, the present results confirm that extended basis sets are needed in the DFT computations of hydrated DNA segments in order to prevent computational artifacts such as the occurrence of underestimated rise coordinates. Overall, M06-2X and B3LYP-D3 functionals give similar results. However, sensibly shorter interbase distances are predicted by the M06-2X functional, as already pointed out in previous work [59], whereas geometries slightly more similar to the standard B-DNA conformation are obtained with B3LYP-D3 geometry optimizations, even when those functionals are used to model the QM layer in ONIOM-ME QM/MM computations.

Although ONIOM computations were carried out only for ss-C${}_{4}$, the results indicate that extended basis sets have to be employed also in ONIOM optimizations using mechanical embedding, in which the interactions between the QM (DNA) and the MM (environment) layers are treated at the MM level. Instead, ONIOM geometry optimizations employing the electric embedding, with enables the polarization of the QM electron density by the environment, are found to be much less dependent on the adopted basis set. Finally, more regular conformations are predicted with ONIOM-EE computations, especially with the B3LYP-D3 functional.

## Figures and Tables

**Table 1:** Predicted average rise and helical rise (Å) for the systems studied at the DFT level.

	B3LYP-D3				M06-2X			
	6-31G(d,p)		TZVP		6-31G(d,p)		TZVP	
	Rise	h-Rise	Rise	h-Rise	Rise	h-Rise	Rise	h-Rise
A${}_{6}$	3.14	3.25	3.30	3.34	2.96	3.09	3.18	3.15
C${}_{6}$	2.74	2.56	3.24	2.94	2.53	2.20	3.42	3.31
G${}_{6}$	3.05	3.12	3.14	3.21	2.94	3.03	3.02	3.09
T${}_{6}$	2.55	2.34	2.66	2.43	2.33	2.00	2.40	1.93
A${}_{2}$C${}_{2}$A${}_{2}$	2.92	3.02	3.12	3.08	2.75	2.73	3.18	3.15
ds-G${}_{4}$	3.43	3.25	3.56	3.33	3.25	3.09	3.40	3.19
ds-A${}_{4}$	3.25	3.03	3.26	2.95	2.98	2.96	3.12	2.81

**Table 2:** Predicted average twist and helical twist (degrees).

	B3LYP-D3				M06-2X			
	6-31G(d,p)		TZVP		6-31G(d,p)		TZVP	
	Twist	h-Twist	Twist	h-Twist	Twist	h-Twist	Twist	h-Twist
ss-A${}_{6}$	37.51	38.23	43.21	44.25	37.21	38.53	43.90	44.17
ss-C${}_{6}$	33.12	35.11	34.53	36.23	33.26	36.54	38.69	39.51
ss-G${}_{6}$	44.04	45.32	45.85	46.77	44.26	45.03	47.21	47.68
ss-T${}_{6}$	33.24	36.36	32.01	34.50	33.62	38.26	31.60	37.29
ss-A${}_{2}$C${}_{2}$A${}_{2}$	36.23	37.66	39.39	39.95	36.91	38.58	42.27	42.78
ds-G${}_{4}$	41.74	43.18	45.76	46.66	44.42	45.57	45.95	47.12
ds-A${}_{4}$	43.56	44.37	46.48	47.39	40.22	40.49	46.56	47.70

**Table 3:** Average rise (Å) and twist (degrees) of ss-C${}_{4}$ predicted by different ONIOM geometry optimizations.

	ME			EE	
	**6-31G(d,p)**	**TZVP**		**6-31G(d,p)**	**TZVP**
			**Rise **		
B3LYP-D3	2.49	2.75		3.10	3.17
M06-2X	2.41	2.77		3.03	3.10
			**Twist**		
B3LYP-D3	29.50	32.60		36.84	36.54
M06-2X	30.22	33.02		37.19	38.22

**Table 4:** RMSD (Å) between optimized geometries obtained by different ONIOM computations and the regular starting conformation (calf thymus B-DNA) for ss-C${}_{4}$.

	ME	EE
B3LYP-D3/6-31G(d,p)	0.74	0.59
B3LYP-D3/TZVP	0.66	0.61
M06-2X/6-31G(d,p)	0.82	0.59
M06-2X/TZVP	0.70	0.61

## Data Availability

Not applicable.

## Supplementary Materials

The supporting information can be downloaded at: https://www.mdpi.com/article/10.3390/ijms232214452/s1.

## References

[1] Bloomfield A.A., Crothers D.M., Tinoco J.J. (2000). Nucleic Acids: Structures, Properties, and Functions.

[2] Croy J.E., Wuttke D.S. (2006). Themes in ssDNA Recognition by Telomere-End Protection Proteins. Trends Biochem. Sci..

[3] Martin D.P., Biagini P., Lefeuvre P., Golden M., Roumagnac P., Varsani A. (2011). Recombination in Eukaryotic Single Stranded DNA Viruses. Viruses.

[4] Kim N., Jinks-Robertson S. (2012). Transcription as a Source of Genome Instability. Nat. Rev. Genet..

[5] Rothemund P. (2006). Folding DNA to Create Nanoscale Shapes and Patterns. Nature.

[6] Seeman N.C. (2010). Nanomaterials Based on DNA. Annu. Rev. Biochem..

[7] Kim D.N., Kilchherr F., Dietz H., Bathe M. (2011). Quantitative Prediction of 3D Solution Shape and Flexibility of Nucleic Acid Nanostructures. Nucleic Acids Res..

[8] Plumridge A., Meisburger S.P., Andresen K., Pollack L. (2017). The Impact of Base Stacking on the Conformations and Electrostatics of Single-Stranded DNA. Nucleic Acids Res..

[9] Huppert J.L. (2008). Four-Stranded Nucleic Acids: Structure, Function and Targeting of G-Quadruplexes. Chem. Soc. Rev..

[10] McIntosh D., Duggan G., Gouil Q., Saleh O. (2014). Sequence-Dependent Elasticity and Electrostatics of Single-Stranded DNA: Signatures of Base-Stacking. Biophys. J..

[11] Ke C., Humeniuk M., S-Gracz H., Marszalek P.E. (2007). Direct Measurements of Base Stacking Interactions in DNA by Single-Molecule Atomic-Force Spectroscopy. Phys. Rev. Lett..

[12] Ramprakash J., Lang B., Schwarz F.P. (2008). Thermodynamics of Single Strand DNA Base Stacking. Biopolymers.

[13] Capobianco A., Velardo A., Peluso A. (2018). Single-Stranded DNA Oligonucleotides Retain Rise Coordinates Characteristic of Double Helices. J. Phys. Chem. B.

[14] Zubatiuk T.A., Shishkin O.V., Gorb L., Hovorun D.M., Leszczynski J. (2013). B-DNA Characteristics Are Preserved in Double stranded d(A)_3_·d(T)_3_ and d(G)_3_·d(C)_3_ Mini-Helixes: Conclusions from DFT/M06-2X Study. Phys. Chem. Chem. Phys..

[15] Šponer J., Bussi G., Krepl M., Banáš P., Bottaro S., Cunha R.A., Gil-Ley A., Pinamonti G., Poblete S., Jurečka P. (2018). RNA Structural Dynamics As Captured by Molecular Simulations: A Comprehensive Overview. Chem. Rev..

[16] Kruse H., Mladek A., Gkionis K., Hansen A., Grimme S., Šponer J. (2015). Quantum Chemical Benchmark Study on 46 RNA Backbone Families Using a Dinucleotide Unit. J. Chem. Theory Comput..

[17] Pérez A., Marchán I., Svozil D., Šponer J., Cheatham T.E., Laughton C.A., Orozco M. (2007). Refinement of the AMBER Force Field for Nucleic Acids: Improving the Description of *α*/*γ* Conformers. Biophys. J..

[18] Ivani I., Dans P.D., Noy A., Pérez A., Faustino I., Hospital A., Walther J., Andrio P., Goñi R., Balaceanu A. (2016). Parmbsc1: A Refined Force Field for DNA Simulations. Nat. Methods.

[19] Galindo-Murillo R., Robertson J.C., Zgarbovic M., Šponer J., Otyepka M., Jurečka P., Cheatham T.E. (2016). Assessing the Current State of Amber Force Field Modifications for DNA. J. Chem. Theory Comput..

[20] Liebl K., Zacharias M. (2021). Tumuc1: A New Accurate DNA Force Field Consistent with High-Level Quantum Chemistry. J. Chem. Theory Comput..

[21] Tucker M.R., Piana S., Tan D., LeVine M.V., Shaw D.E. (2022). Development of Force Field Parameters for the Simulation of Single- and Double-Stranded DNA Molecules and DNA-Protein Complexes. J. Phys. Chem. B.

[22] Chakraborty K., Mantha S.R., Bandyopadhyay S. (2013). Molecular Dynamics Simulation of a Single-Stranded DNA with Heterogeneous Distribution of Nucleobases in Aqueous Medium. J. Chem. Phys..

[23] Norberg J., Nilsson L. (1995). Potential of Mean Force Calculations of the Stacking-Unstacking Process in Single Stranded Deoxyribonucleoside Monophosphates. Biophys. J..

[24] Sen S., Nilsson L. (2001). MD Simulations of Homomorphous PNA, DNA, and RNA Single Strands: Characterization and Comparison of Conformations and Dynamics. J. Am. Chem. Soc..

[25] Shao J., Tanner S.W., Thompson N., Cheatham T.E. (2007). Clustering Molecular Dynamics Trajectories: 1. Characterizing the Performance of Different Clustering Algorithms. J. Chem. Theory Comput..

[26] Jafilan S., Klein L., Hyun C., Florián J. (2012). Intramolecular Base Stacking of Dinucleoside Monophosphate Anions in Aqueous Solution. J. Phys. Chem. B.

[27] Brown R.F., Andrews C.T., Elcock A.H. (2015). Stacking Free Energies of All DNA and RNA Nucleoside Pairs and Dinucleoside-Monophosphates Computed Using Recently Revised AMBER Parameters and Compared with Experiment. J. Chem. Theory Comput..

[28] Hayatshahi H.S., Henriksen N.M., Cheatham T.E. (2018). Consensus Conformations of Dinucleoside Monophosphates Described with Well-Converged Molecular Dynamics Simulations. J. Chem. Theory Comput..

[29] Kruse H., Havrila M., Šponer J. (2014). QM Computations on Complete Nucleic Acids Building Blocks: Analysis of the Sarcin-Ricin RNA Motif Using DFT-D3, HF-3c, PM6-D3H, and MM Approaches. J. Chem. Theory Comput..

[30] Sponer J., Riley K.E., Hobza P. (2008). Nature and Magnitude of Aromatic Stacking of Nucleic Acid Bases. Phys. Chem. Chem. Phys..

[31] Zubatiuk T., Kukuev M.A., Korolyova A.S., Gorb L., Nyporko A., Hovorun D., Leszczynski J. (2015). Structure and Binding Energy of Double-Stranded A-DNA Mini-helices: Quantum-Chemical Study. J. Phys. Chem. B.

[32] Barone G., Fonseca Guerra C., Bickelhaupt F.M. (2013). B-DNA Structure and Stability as Function of Nucleic Acid Composition: Dispersion-Corrected DFT Study of Dinucleoside Monophosphate Single and Double Strands. ChemistryOpen.

[33] Šponer J., Mladek A., Špackova N., Cheatham T.E., Grimme S. (2013). Relative Stability of Different DNA Guanine Quadruplex Stem Topologies Derived Using Large-Scale Quantum-Chemical Computations. J. Am. Chem. Soc..

[34] Churchill C.D.M., Wetmore S.D. (2011). Developing a Computational Model that Accurately Reproduces the Structural Features of a Dinucleoside Monophosphate Unit within B-DNA. Phys. Chem. Chem. Phys..

[35] Capobianco A., Caruso T., D’Ursi A.M., Fusco S., Masi A., Scrima M., Chatgilialoglu C., Peluso A. (2015). Delocalized Hole Domains in Guanine-Rich DNA Oligonucleotides. J. Phys. Chem. B.

[36] Calladine C.R., Drew H.R., Luisi B.F., Travers A.A. (2004). Understanding DNA.

[37] Dickerson R.E., Rossmann R.G., Arnold E. (2001). Nucleic Acids. International Tables for Crystallography Volume F: Crystallography of Biological Macromolecules.

[38] Isaksson J., Acharya S., Barman J., Cheruku P., Chattopadhyaya J. (2004). Single-Stranded Adenine-Rich DNA and RNA Retain Structural Characteristics of Their Respective Double-Stranded Conformations and Show Directional Differences in Stacking Pattern. Biochemistry.

[39] Mills J.B., Vacano E., Hagerman P.J. (1999). Flexibility of Single-Stranded DNA: Use of Gapped Duplex Helices to Determine the Persistence Lengths of Poly(dT) and Poly(dA). J. Mol. Biol..

[40] Capobianco A., Caruso T., Celentano M., D’Ursi A.M., Scrima M., Peluso A. (2013). Stacking Interactions between Adenines in Oxidized Oligonucleotides. J. Phys. Chem. B.

[41] Chung L.W., Sameera W.M.C., Ramozzi R., Page A.J., Hatanaka M., Petrova G.P., Harris T.V., Li X., Ke Z., Liu F. (2015). The ONIOM Method and Its Applications. Chem. Rev..

[42] Heddi B., Oguey C., Lavelle C., Foloppe N., Hartmann B. (2010). Intrinsic Flexibility of B-DNA: The Experimental TRX Scale. Nucleic Acids Res..

[43] Tubbs J.D., Condon D.E., Kennedy S.D., Hauser M., Bevilacqua P.C., Turner D.H. (2013). The Nuclear Magnetic Resonance of CCCC RNA Reveals a Right-Handed Helix, and Revised Parameters for AMBER Force Field Torsions Improve Structural Predictions from Molecular Dynamics. Biochemistry.

[44] Senthilkumar K., Grozema F.C., Fonseca Guerra C., Bickelhaupt F.M., Lewis F.D., Berlin Y.A., Ratner M.A., Siebbeles L.D.A. (2005). Absolute Rates of Hole Transfer in DNA. J. Am. Chem. Soc..

[45] Sugiyama H., Saito I. (1996). Theoretical Studies of GG-Specific Photocleavage of DNA via Electron Transfer: Significant Lowering of Ionization Potential and 5^′^-Localization of HOMO of Stacked GG Bases in B-form DNA. J. Am. Chem. Soc..

[46] Capobianco A., Landi A., Peluso A. (2017). Modeling DNA Oxidation in Water. Phys. Chem. Chem. Phys..

[47] Borrelli R., Capobianco A., Landi A., Peluso A. (2015). Vibronic Couplings and Coherent Electron Transfer in Bridged Systems. Phys. Chem. Chem. Phys..

[48] Kubař T., Woiczikowski P.B., Cuniberti G., Elstner M. (2008). Efficient Calculation of Charge-Transfer Matrix Elements for Hole Transfer in DNA. J. Phys. Chem. B.

[49] Landi A., Borrelli R., Capobianco A., Peluso A. (2019). Transient and Enduring Electronic Resonances Drive Coherent Long Distance Charge Transport in Molecular Wires. J. Phys. Chem. Lett..

[50] Landi A., Capobianco A., Peluso A. (2020). Coherent Effects in Charge Transport in Molecular Wires: Toward a Unifying Picture of Long-Range Hole Transfer in DNA. J. Phys. Chem. Lett..

[51] Landi A., Capobianco A., Peluso A. (2021). The Time Scale of Electronic Resonance in Oxidized DNA as Modulated by Solvent Response: An MD/QM-MM Study. Molecules.

[52] Peluso A., Caruso T., Landi A., Capobianco A. (2019). The Dynamics of Hole Transfer in DNA. Molecules.

[53] Capobianco A., Carotenuto M., Caruso T., Peluso A. (2009). The Charge-Transfer Band of an Oxidized Watson-Crick Guanosine-Cytidine Complex. Angew. Chem. Int. Ed..

[54] Genereux J.C., Barton J.K. (2010). Mechanisms for DNA Charge Transport. Chem. Rev..

[55] Kanvah S., Joseph J., Schuster G.B., Barnett R.N., Cleveland C.L., Landman U. (2010). Oxidation of DNA: Damage to Nucleobases. Acc. Chem. Res..

[56] Kawai K., Majima T. (2013). Hole Transfer Kinetics of DNA. Acc. Chem. Res..

[57] Liang L., Fu Y., Wang D., Wei Y., Kobayashi N., Minari T. (2018). DNA as Functional Material in Organic-Based Electronics. Appl. Sci..

[58] Etheve L., Martin J., Lavery R. (2017). Decomposing Protein-DNA Binding and Recognition Using simplified Protein Models. Nucleic Acids Res..

[59] Smith D.A., Holroyd L.F., van Mourik T., Jones A.C. (2016). A DFT Study of 2-Aminopurine-Containing Dinucleotides: Prediction of Stacked Conformations with B-DNA Structure. Phys. Chem. Chem. Phys..

[60] Hunter R.S., van Mourik T. (2012). DNA Base Stacking: The Stacked Uracil/Uracil and Thymine/Thymine Minima. J. Comput. Chem..

[61] Kannan S., Kohlhoff K., Zacharias M. (2006). B-DNA Under Stress: Over- and Untwisting of DNA during Molecular Dynamics Simulations. Biophys. J..

[62] El Hassan M.A., Calladine C.R. (1997). Conformational Characteristics of DNA: Empirical Classifications and a Hypothesis for the Conformational Behaviour of Dinucleotide Steps. Philos. Trans. R. Soc. A.

[63] Stellwagen E., Stellwagen N.C. (2020). Electrophoretic Mobility of DNA in Solutions of High Ionic Strength. Byophys. J..

[64] Chen H., Meisburger S.P., Pabit S.A., Sutton J.L., Webb W.W., Pollack L. (2012). Ionic Strength-Dependent Persistence Lengths of Single-Stranded RNA and DNA. Proc. Natl. Acad. Sci. U.S.A..

[65] Capobianco A., Peluso A. (2014). The Oxidization Potential of AA Steps in Single Strand DNA Oligomers. RSC Adv..

[66] Capobianco A., Caruso T., Peluso A. (2015). Hole Delocalization over Adenine Tracts in Single Stranded DNA Oligonucleotides. Phys. Chem. Chem. Phys..

[67] González-Olvera J.C., Zamorano-Carrillo A., Arreola-Jardón G., Pless R.C. (2022). Residue Interactions Affecting the Deprotonation of Internal Guanine Moieties in Oligodeoxyribonucleotides, Calculated by FMO Methods. J. Mol. Model..

[68] Han X., Wang E., Cui Y., Lin Y., Chen H., An R., Liang X., Komiyama M. (2019). The Staining Efficiency of Cyanine Dyes for Single-Stranded DNA is Enormously Dependent on Nucleotide Composition. Electrophoresis.

[69] Norberg J., Nilsson L. (1996). Influence of Adjacent Bases on the Stacking-Unstacking Process of Single-stranded Oligonucleotides. Biopolymers.

[70] Erie D.A., Breslauer K.J., Olson W.K. (1993). A Monte Carlo Method for Generating Structures of Short Single-Stranded DNA Sequences. Biopolymers.

[71] Vokáčová Z., Buděšínský M., Rosenberg I., Schneider B., Šponer J., Sychrovský V. (2009). Structure and Dynamics of the ApA, ApC, CpA, and CpC RNA Dinucleoside Monophosphates Resolved with NMR Scalar Spin-Spin Couplings. J. Phys. Chem. B.

[72] Pearlman D.A., Kim S.H. (1988). Conformational Studies of Nucleic Acids. V. Sequence Specificities in the Conformational Energetics of Oligonucleotides: The Homo-Tetramers. Biopolymers.

[73] Kabeláč M., Hobza P. (2001). Potential Energy and Free Energy Surfaces of all Ten Canonical and Methylated Nucleic Acid Base Pairs: Molecular Dynamics and Quantum Chemical ab initio Studies. J. Phys. Chem. B.

[74] Kypr J., Kejnovská I., Renčiuk D., Vorlíčková M. (2009). Circular Dichroism and Conformational Polymorphism of DNA. Nucleic Acids Res..

[75] Petrov A.S., Lamm G., Pack G.R. (2004). The Triplex-Hairpin Transition in Cytosine-Rich DNA. Biophys. J..

[76] Arnott S., Chandrasekaran R., Leslie A.G.W. (1976). Structure of the Single-Stranded Polyribonucleotide Polycytidylic Acid. J. Mol. Biol..

[77] Berman H.M., Westbrook J., Feng Z., Gilliland G., Bhat T.N., Weissig H., Shindyalov I.N., Bourne P.E. (2000). The Protein Data Bank. Nucleic Acids Res..

[78] Tisné C., Hantz E., Hartmann B., Delepierre M. (1998). Solution Structure of a Non-Palindromic 16 Base-Pair DNA Related to the HIV-1 *κ*b site: Evidence for BI-BII Equilibrium Inducing a Global Dynamic Curvature of the Duplex. J. Mol. Biol..

[79] DiGabriele A.D., Steitz T.A. (1993). A DNA Dodecamer Containing an Adenine Tract Crystallizes in a Unique Lattice and Exhibits a New Bend. J. Mol. Biol..

[80] MacDonald D., Herbert K., Zhang X., Polgruto T., Lu P. (2001). Solution Structure of an A-Tract DNA Bend. J. Mol. Biol..

[81] Lu X.J., Olson W.K. (2003). 3DNA: A Software Package for the Analysis, Rebuilding and Visualization of Three-Dimensional Nucleic Acid Structures. Nucleic Acids Res..

[82] Lu X.J., Olson W.K. (2008). 3DNA: A Versatile, Integrated Software System for the Analysis, Rebuilding and Visualization of Three-dimensional Nucleic-Acid Structures. Nat. Protoc..

[83] Frisch M.J., Trucks G.W., Schlegel H.B., Scuseria G.E., Robb M.A., Cheeseman J.R., Scalmani G., Barone V., Mennucci B., Petersson G.A. (2009). Gaussian 09 Revision D.01.

[84] Zhao Y., Truhlar D.G. (2008). The M06 Suite of Density Functionals for Main Group Thermochemistry, Thermochemical Kinetics, Noncovalent Interactions, Excited States, and Transition Elements: Two New Functionals and Systematic Testing of Four M06-class Functionals and 12 Other Functionals. Theor. Chem. Acc..

[85] Becke A.D. (1993). Density-Functional Thermochemistry. III. The Role of Exact Exchange. J. Chem. Phys..

[86] Stephens P.J., Devlin F.J., Chabalowski C.F., Frisch M.J. (1994). Ab Initio Calculation of Vibrational Absorption and Circular Dichroism Spectra Using Density Functional Force Fields. J. Phys. Chem..

[87] Grimme S., Ehrlich S., Goerigk L. (2011). Effect of the Damping Function in Dispersion Corrected Density Functional Theory. J. Comput. Chem..

[88] Hehre W.J., Ditchfield R., Pople J.A. (1972). Self-Consistent Molecular Orbital Methods. XII. Further Extensions of Gaussian-Type Basis Sets for Use in Molecular Orbital Studies of Organic Molecules. J. Chem. Phys..

[89] Schäfer A., Huber C., Ahlrichs R. (1994). Fully Optimized Contracted Gaussian-Basis Sets of Triple Zeta Valence Quality for Atoms Li to Kr. J. Chem. Phys..

[90] Tomasi J., Mennucci B., Cammi R. (2005). Quantum Mechanical Continuum Solvation Models. Chem. Rev..

[91] Singh A., Singh N. (2015). Effect of Salt Concentration on the Stability of Heterogeneous DNA. Physica A.

[92] Hong F., Schreck J.S., Šulc P. (2020). Understanding DNA Interactions in Crowded Environments with a Coarse-Grained Model. Nucleic Acids Res..

[93] World T., Fund W., Archipelago S. (2007). Spartan’04.

[94] Case D.A., Ben-Shalom I.Y., Brozell S.R., Cerutti D.S., Cheatham III T.E., Cruzeiro V.W.D., Darden T.A., Duke R.E., Ghoreishi D., Gilson M.K. (2018). AMBER 18.

[95] Joung I.S., Cheatham T.E. (2008). Determination of Alkali and Halide Monovalent Ion Parameters for Use in Explicitly Solvated Biomolecular Simulations. J. Phys. Chem. B.

[96] Price D.J., Brooks C.L. (2004). A Modified TIP3P Water Potential for Simulation with Ewald Summation. J. Chem. Phys..

[97] Toukmaji A., Sagui C., Board J., Darden T. (2000). Efficient Particle-Mesh Ewald Based Approach to Fixed and Induced Dipolar Interactions. J. Chem. Phys..

[98] Miyamoto S., Kollman P.A. (1992). SETTLE: An Analytical Version of the SHAKE and RATTLE Algorithm for Rigid Water Models. J. Comput. Chem..

[99] Clemente F.R., Vreven T., Frisch M.J. (2010). Getting the Most out of ONIOM: Guidelines and Pitfalls. Quantum Biochemistry.

