# Supplementary material for: Duplex DNA Retains the Conformational Features of Single Strands: Perspectives from MD Simulations and Quantum Chemical Computations
Source: Int J Mol Sci. 2022 Nov 21;23(22):14452. doi: 10.3390/ijms232214452 (PMC9697240; doi:10.3390/ijms232214452)
Supplement: Supplementary file 1 [file ijms-23-14452-s001.zip › ijms-2016115-supplementary.pdf]

Duplex DNA Retains the Conformational Features of Single-Strands: Perspectives from MD Simulations and Quantum Chemical Computations

– Supplementary Materials –

Amedeo Capobianco, Alessandro Landi, Andrea Peluso*
Dipartimento di Chimica e Biologia “A. Zambelli”, Università di Salerno
Via Giovanni Paolo II, I-84084, Fisciano (SA), Italy.
*Email: apeluso@unisa.it

TABLE OF CONTENTS

- Rigid coordinates extracted from experimental structures (Tables S1-S3)
- Rigid coordinates extracted from optimized (DFT/PCM and ONIOM) geometries (Tables S4-S47)
- Predicted (DFT/PCM) sugar conformational parameters of double strands
- Predicted (ONIOM) sugar conformational parameters of ss-C_4
- DFT/PCM optimized geometries
- ONIOM Optimized geometries
- Structures from the MD simulation of ss-C_6

Rigid coordinates

Shift, slide and Rise are expressed in Å; tilt, roll and twist in degrees. Please note that the same order as in the following ds-5′-C T G G G G A C T T T C C A G G-3′ has been considered for all the double helices (both experimental and predicted geometries), when the analysis has been carried out in terms of the rigid coordinates of the individual single strands composing the duplex.

Rigid coordinates extracted from experimental structures

Table S1: Local base-step parameters for the individual single strands composing the ds-5′-C T G G G G A C T T T C C A G G-3′ sequence, as stored in the 1kbd PDB file, method: NMR (solution).

step	Shift	Slide	Rise	Tilt	Roll	Twist
5′-C/T	-1.90	-0.79	3.03	12.60	-35.95	30.45
T/G	1.98	-0.27	3.24	9.10	0.11	36.16
G/G	-0.14	-0.71	3.46	-1.29	0.99	35.31
G/G	0.02	-1.63	4.01	-6.33	-12.33	34.40
G/G	0.03	-1.69	3.84	-2.74	-13.28	34.96
G/A	0.29	-1.18	3.37	2.38	-5.99	36.44
A/C	0.15	-1.39	3.64	-4.91	-4.40	35.93
C/T	-0.75	-1.92	3.72	-4.20	-14.21	30.80
T/T	-0.22	-0.81	2.98	7.93	-13.52	36.36
T/T	0.39	-0.31	2.93	8.14	-5.49	38.87
T/C	1.17	-0.86	3.24	1.21	5.82	36.29
C/C	-0.33	-1.82	3.79	-11.49	5.67	32.67
C/A	-0.55	-2.01	3.21	9.86	-14.04	26.65
A/G	0.41	-1.28	3.29	4.34	2.71	30.11
G/G	0.49	-1.27	3.52	0.28	-2.34	36.92
C/C	-0.82	-1.50	3.42	-1.10	-17.10	35.59
C/T	-0.57	-1.35	3.05	5.12	-5.77	28.44
T/G	0.25	-1.95	3.42	-0.27	2.87	28.26
G/G	0.14	-1.77	3.59	-3.38	-0.07	32.77
G/A	-0.32	-0.87	3.30	3.50	-4.73	35.93
A/A	0.03	-0.07	3.19	5.48	-4.04	38.94
A/A	0.52	-0.19	3.23	5.56	-3.03	37.30
A/G	0.55	-1.61	3.86	-5.69	-0.06	33.05
G/T	0.14	-1.30	3.41	2.03	-6.21	36.18
T/C	0.15	-1.19	3.44	-4.93	0.30	36.55
C/C	-0.31	-1.71	3.87	-8.76	-11.31	33.73
C/C	-0.74	-2.15	3.83	-8.38	-15.70	30.94
C/C	-0.46	-1.20	3.26	1.42	-13.13	35.18
C/A	-1.86	0.01	3.43	11.02	-24.41	31.51
A/G-3′	1.44	-0.21	3.38	3.15	-0.75	38.16

Table S2: Local base-step parameters for the individual single strands composing the ds-5′-C G C G A A A A A A C G-3′ sequence, as stored in the 1d89 PDB file, Method: X-ray diffraction.

step	Shift	Slide	Rise	Tilt	Roll	Twist
C/G	-0.14	1.58	3.81	6.53	9.14	34.01
G/C	2.52	0.86	3.33	6.87	-11.64	46.17
C/G	-0.74	0.66	2.86	11.91	10.99	24.82
G/A	1.00	-0.09	3.32	2.69	1.62	44.61
A/A	0.22	-0.08	3.25	4.21	-1.30	31.44
A/A	0.72	-0.42	3.20	4.10	-2.79	37.36
A/A	0.79	-0.22	3.07	6.41	2.16	39.12
A/A	0.17	-0.20	3.19	3.32	-0.60	29.76
A/A	0.81	-0.13	3.33	1.02	-0.26	36.72
A/C	0.90	-0.33	2.76	11.73	6.61	31.00
C/G	0.89	-0.77	3.75	-4.62	11.30	29.79
C/G	-0.88	-0.77	3.93	-11.10	-1.34	33.04
G/T	0.86	-0.61	3.20	7.69	8.16	39.08
T/T	0.02	0.02	2.37	18.74	-2.63	28.76
T/T	0.74	0.20	3.40	3.75	2.70	35.82
T/T	0.47	-0.32	3.79	-1.84	4.31	35.97
T/T	0.71	-0.73	3.40	0.74	-3.46	37.02
T/T	0.67	-0.46	3.44	0.96	-9.21	34.00
T/C	0.26	-0.69	2.82	9.51	-1.35	36.84
C/G	0.73	0.66	3.00	10.74	4.61	31.09
G/C	-0.12	1.20	2.89	10.56	-3.39	42.44
C/G	0.26	2.29	3.69	1.09	4.36	33.94

Table S3: Local base-step parameters for the individual single strands composing the 5′-G G C A A A A A A C G G- 3′ sequence, as stored in the 1fzx PDB file, method: NMR (solution).

step	Shift	Slide	Rise	Tilt	Roll	Twist
G/G	-0.02	-0.97	3.09	2.41	1.47	32.54
G/C	0.31	-0.85	2.69	8.71	0.85	33.51
C/A	0.47	-0.56	3.29	4.39	6.32	31.87
A/A	0.44	-0.52	3.04	4.42	0.35	38.06
A/A	0.39	-0.38	3.07	4.72	-0.92	32.97
A/A	0.64	-0.82	3.13	1.30	1.50	39.89
A/A	0.52	-0.84	3.01	2.43	-1.08	38.99
A/A	0.27	-0.90	3.26	-0.99	-3.31	37.68
A/C	0.11	-1.34	3.12	-1.85	-0.19	35.70
C/G	-0.52	-0.99	2.09	13.13	4.94	30.67
G/G	-0.18	-0.70	3.11	-1.15	7.21	33.49
C/C	0.91	-0.40	2.80	11.33	9.82	30.96
C/G	0.34	-0.67	3.25	4.91	9.71	28.03
G/T	1.62	-0.68	2.77	8.16	-3.83	39.54
T/T	0.83	-0.76	2.98	4.27	5.79	38.67
T/T	0.24	-1.16	2.98	4.46	-0.58	31.86
T/T	0.29	-1.37	2.95	4.55	-5.12	33.64
T/T	0.94	-1.04	3.16	2.55	-3.17	36.41
T/T	0.18	-0.99	2.70	8.60	-0.13	34.50
T/G	-0.07	-0.89	2.72	4.21	9.03	35.69
G/C	0.75	-0.49	2.97	4.90	-4.37	34.47
C/C	1.61	-0.02	2.76	10.42	6.42	38.23

Rigid coordinates extracted from optimized (DFT/PCM) geometries

Table S4: Local base-step parameters for ss-T6 [B3LYP-D3/6-31G(d,p)]

step	Shift	Slide	Rise	Tilt	Roll	Twist
T/T	-0.71	-0.26	2.35	16.80	-15.52	27.06
T/T	0.16	0.29	2.54	12.23	-4.34	34.50
T/T	0.24	0.42	2.56	12.02	-1.55	34.31
T/T	0.33	0.38	2.59	11.19	-1.30	34.93
T/T	0.36	0.19	2.70	9.38	-3.54	35.42
ave.	0.08	0.20	2.55	12.32	-5.25	33.24

Table S5: Local base-step parameters for ss-A6 [B3LYP-D3/6-31G(d,p)]

step	Shift	Slide	Rise	Tilt	Roll	Twist
A/A	0.33	1.11	3.11	3.07	10.40	30.71
A/A	0.57	0.75	3.07	5.20	7.30	37.15
A/A	0.68	0.73	3.12	4.46	3.51	39.02
A/A	0.68	0.61	3.16	3.35	2.82	39.54
A/A	0.65	0.36	3.24	0.53	3.55	41.12
ave.	0.58	0.71	3.14	3.32	5.51	37.51

Table S6: Local base-step parameters for the individual single strands composing the ds-A4 sequence optimized at the B3LYP-D3/6-31G(d,p) level

step	Shift	Slide	Rise	Tilt	Roll	Twist
A/A	0.89	1.53	2.89	15.87	-0.47	36.94
A/A	0.96	1.49	3.14	3.84	1.07	42.02
A/A	0.14	1.72	3.20	2.17	-5.36	47.22
T/T	1.15	1.56	3.13	9.91	-18.53	48.15
T/T	0.72	0.65	3.42	-0.11	-11.43	42.01
T/T	0.42	0.19	3.05	5.09	-7.97	39.66

Table S7: Local base-step parameters for ds-A4 [B3LYP-D3/6-31G(d,p)]

step	Shift	Slide	Rise	Tilt	Roll	Twist
AA/TT	-0.22	0.87	3.11	-5.38	-4.10	39.68
AA/TT	0.50	1.63	3.24	3.85	-12.05	48.31
AA/TT	-0.13	1.07	3.41	-1.97	-5.20	42.68
ave.	0.05	1.19	3.25	-1.17	-7.12	43.56

Table S8: Local base-step parameters for ss-C6 [B3LYP-D3/6-31G(d,p)]

step	Shift	Slide	Rise	Tilt	Roll	Twist
C/C	-0.46	0.31	2.45	16.39	-10.72	29.23
C/C	0.19	0.54	2.81	8.12	-0.29	33.90
C/C	0.11	0.44	2.70	10.24	-1.52	33.49
C/C	0.14	0.45	2.76	9.32	-0.31	33.77
C/C	0.16	0.31	2.97	5.49	-0.53	35.21
ave.	0.03	0.41	2.74	9.91	-2.67	33.12

Table S9: Local base-step parameters for ss-G6 [B3LYP-D3/6-31G(d,p)]

step	Shift	Slide	Rise	Tilt	Roll	Twist
G/G	1.06	0.82	3.12	11.77	-8.73	40.46
G/G	1.06	0.89	3.09	7.09	-4.10	41.29
G/G	1.12	0.76	3.03	9.15	-4.47	41.18
G/G	1.36	0.50	3.05	8.29	-6.45	41.36
G/G	1.48	0.24	2.98	8.88	-3.53	55.91
ave.	1.22	0.64	3.05	9.04	-5.46	44.04

Table S10: Local base-step parameters for the individual single strands composing the ds-G4 sequence optimized at the B3LYP-D3/6-31G(d,p) level

step	Shift	Slide	Rise	Tilt	Roll	Twist
G/G	1.53	1.40	3.23	12.56	-10.29	42.76
G/G	1.20	0.87	3.29	4.19	-7.28	41.39
G/G	0.36	-0.10	3.26	2.19	-3.24	40.20
C/C	0.13	0.51	3.14	5.62	-14.49	40.02
C/C	0.11	0.85	3.58	-2.38	-6.24	41.43
C/C	-0.35	0.81	3.79	-6.75	-15.13	43.97

Table S11: Local base-step parameters for ds-G4 [B3LYP-D3/6-31G(d,p)]

step	Shift	Slide	Rise	Tilt	Roll	Twist
GG/CC	0.97	1.11	3.58	9.74	-12.75	43.67
GG/CC	0.56	0.85	3.50	3.33	-6.78	41.48
GG/CC	0.11	0.21	3.23	-1.65	-8.91	40.05
ave.	0.55	0.72	3.43	3.81	-9.48	41.74

Table S12: Local base-step parameters for ss-AACCAA [B3LYP-D3/6-31G(d,p)]

step	Shift	Slide	Rise	Tilt	Roll	Twist
A/A	0.28	1.09	3.12	2.51	10.97	28.48
A/C	0.58	0.08	2.66	9.90	4.45	35.14
C/C	0.35	0.50	2.63	11.91	-0.53	35.61
C/A	1.32	1.22	2.94	9.47	1.16	41.30
A/A	0.73	0.38	3.23	0.77	6.55	40.63
ave.	0.65	0.65	2.92	6.91	4.52	36.23

Table S13: Local base-step parameters for ss-T6 (B3LYP-D3/TZVP)

step	Shift	Slide	Rise	Tilt	Roll	Twist
T/T	0.19	0.48	2.78	8.65	-2.32	33.07
T/T	-0.22	-0.05	2.63	11.70	-6.99	31.09
T/T	-0.33	-0.14	2.59	12.40	-8.49	30.63
T/T	-0.13	0.00	2.59	12.74	-6.31	31.94
T/T	-0.01	0.01	2.71	10.53	-6.55	33.30
ave.	-0.10	0.06	2.66	11.20	-6.13	32.01

Table S14: Local base-step parameters for ss-A6 (B3LYP-D3/TZVP)

step	Shift	Slide	Rise	Tilt	Roll	Twist
A/A	0.24	1.49	3.28	1.78	-0.78	40.77
A/A	1.00	-0.15	3.51	-7.15	10.82	51.17
A/A	1.63	1.47	3.18	4.98	13.34	34.29
A/A	0.32	1.77	3.16	3.77	0.89	43.20
A/A	0.80	0.16	3.35	-1.91	8.20	46.64
ave.	0.80	0.94	3.30	0.29	6.49	43.21

Table S15: Local base-step parameters for the individual single strands composing the ds-A4 sequence optimized at the B3LYP-D3/TZVP level

step	Shift	Slide	Rise	Tilt	Roll	Twist
A/A	0.34	1.98	2.97	8.04	-0.55	45.40
A/A	0.30	1.86	3.11	5.13	-3.86	45.68
A/A	0.50	1.52	3.31	0.50	-5.63	48.16
T/T	0.71	1.32	3.12	9.34	-18.23	44.95
T/T	1.06	0.94	3.43	1.49	-10.58	44.01
T/T	0.62	0.76	3.08	8.11	-13.77	46.12

Table S16: Local base-step parameters for ds-A4 (B3LYP-D3/TZVP)

AA/TT	0.14	1.36	3.12	0.10	-7.31	46.94
AA/TT	0.35	1.40	3.38	-1.82	-7.32	45.40
AA/TT	0.12	1.41	3.28	4.46	-12.00	47.10
ave.	0.21	1.39	3.26	0.91	-8.88	46.48

Table S17: Local base-step parameters for ss-C6 (B3LYP-D3/TZVP)

step	Shift	Slide	Rise	Tilt	Roll	Twist
C/C	-0.43	0.33	2.82	11.05	-8.68	29.66
C/C	-0.17	0.30	3.04	5.70	-2.42	32.33
C/C	-0.22	0.21	2.95	7.23	-3.93	32.23
C/C	-0.32	-0.02	3.18	3.05	-4.14	32.07
C/C	0.92	-0.64	4.20	-12.56	13.83	46.36
ave.	-0.04	0.04	3.24	2.89	-1.07	34.53

Table S18: Local base-step parameters for ss-G6 (B3LYP-D3/TZVP)

step	Shift	Slide	Rise	Tilt	Roll	Twist
G/G	1.28	1.05	3.14	8.14	-4.12	40.81
G/G	1.30	0.89	3.11	8.11	-3.61	41.87
G/G	1.29	0.82	3.08	10.26	-4.16	41.77
G/G	1.16	0.87	3.21	5.94	-7.40	47.54
G/G	1.52	0.40	3.17	6.07	-5.09	57.26
ave.	1.31	0.81	3.14	7.70	-4.88	45.85

Table S19: Local base-step parameters for the individual single strands composing the ds-G4 sequence optimized at the B3LYP-D3/TZVP level

step	Shift	Slide	Rise	Tilt	Roll	Twist
G/G	0.96	0.99	3.30	5.31	-5.72	39.24
G/G	0.17	1.96	3.39	3.96	-9.59	48.29
G/G	0.37	1.45	3.61	-3.27	-10.36	49.37
C/C	0.91	2.09	3.21	10.27	-6.19	51.13
C/C	1.12	1.66	3.69	-5.00	-5.04	48.58
C/C	-0.45	0.02	3.77	-7.46	-5.53	37.54

Table S20: Local base-step parameters for ds-G4 (B3LYP-D3/TZVP)

step	Shift	Slide	Rise	Tilt	Roll	Twist
GG/CC	0.72	0.53	3.58	6.37	-5.66	38.35
GG/CC	-0.45	1.82	3.61	4.49	-7.30	48.31
GG/CC	-0.29	1.77	3.49	-6.87	-8.31	50.64
ave.	-0.01	1.37	3.56	1.33	-7.09	45.76

Table S21: Local base-step parameters for ss-AACCAA (B3LYP-D3/TZVP)

step	Shift	Slide	Rise	Tilt	Roll	Twist
A/A	0.18	1.67	3.17	3.72	0.92	37.34
A/C	0.19	-0.01	3.00	6.09	-1.08	33.33
C/C	-0.25	0.22	2.94	7.77	-3.29	32.26
C/A	0.69	0.98	3.14	4.07	-2.41	46.58
A/A	0.78	0.07	3.36	-2.00	8.71	47.45
ave.	0.31	0.59	3.12	3.93	0.57	39.39

Table S22: Local base-step parameters for ss-T6 [M06-2X/6-31G(d,p)]

step	Shift	Slide	Rise	Tilt	Roll	Twist
T/T	-0.70	-0.39	2.19	16.52	-14.97	27.47
T/T	0.08	0.22	2.35	13.19	-8.96	35.67
T/T	0.03	0.07	2.40	12.26	-8.92	34.78
T/T	-0.25	-0.16	2.36	13.35	-11.37	31.51
T/T	-0.01	0.03	2.33	14.27	-13.80	38.69
ave.	-0.17	-0.05	2.33	13.92	-11.60	33.62

Table S23: Local base-step parameters for ss-A6 [M06-2X/6-31G(d,p)]

step	Shift	Slide	Rise	Tilt	Roll	Twist
A/A	0.11	1.56	2.85	6.03	9.17	28.20
A/A	0.37	1.44	2.75	10.38	8.75	32.18
A/A	0.46	1.10	2.94	6.39	6.30	32.85
A/A	0.70	0.22	3.12	0.16	4.80	41.07
A/A	1.08	-0.10	3.14	-1.97	6.80	51.75
ave.	0.54	0.84	2.96	4.20	7.16	37.21

Table S24: Local base-step parameters for the individual single strands composing the ds-A4 sequence optimized at the M06-2X/6-31G(d,p) level

step	Shift	Slide	Rise	Tilt	Roll	Twist
A/A	1.12	1.10	2.97	4.92	6.00	33.70
A/A	1.03	0.32	2.95	5.81	3.38	42.06
A/A	0.59	0.07	3.04	0.51	2.29	46.93
T/T	-0.04	0.06	2.62	9.43	-14.94	36.93
T/T	0.08	0.17	3.00	2.93	-9.22	39.06
T/T	0.39	0.16	2.91	3.83	-4.75	39.22

Table S25: Local base-step parameters for ds-A4 [M06-2X/6-31G(d,p)]

step	Shift	Slide	Rise	Tilt	Roll	Twist
AA/TT	-0.35	0.60	3.07	-0.68	0.56	37.34
AA/TT	-0.47	0.23	3.03	-1.62	-3.03	41.12
AA/TT	-0.30	0.07	2.84	4.72	-6.19	42.19
ave.	-0.37	0.30	2.98	0.81	-2.89	40.22

Table S26: Local base-step parameters for ss-C6 [M06-2X/6-31G(d,p)]

step	Shift	Slide	Rise	Tilt	Roll	Twist
C/C	-0.94	-0.25	2.44	13.05	-17.59	25.91
C/C	-0.48	-0.06	2.40	12.35	-8.40	31.28
C/C	-0.14	0.10	2.46	11.69	-5.18	33.32
C/C	-0.09	0.22	2.53	10.42	-4.91	33.63
C/C	0.13	0.21	2.83	4.56	-8.30	42.16
ave.	-0.31	0.04	2.53	10.41	-8.88	33.26

Table S27: Local base-step parameters for ss-G6 [M06-2X/6-31G(d,p)]

step	Shift	Slide	Rise	Tilt	Roll	Twist
G/G	1.01	1.25	2.94	7.51	-2.47	41.83
G/G	1.14	1.13	2.94	7.58	-2.30	42.24
G/G	1.15	0.98	2.93	9.12	-3.08	41.71
G/G	1.29	0.46	3.03	6.18	-5.69	41.26
G/G	1.42	0.04	2.85	7.96	-1.73	54.28
ave.	1.20	0.77	2.94	7.67	-3.05	44.26

Table S28: Local base-step parameters for the individual single strands composing the ds-G4 sequence optimized at the M06-2X/6-31G(d,p) level

step	Shift	Slide	Rise	Tilt	Roll	Twist
G/G	0.75	1.18	2.99	6.89	-3.35	40.38
G/G	1.02	1.46	3.06	5.66	-4.94	47.08
G/G	-0.56	1.54	3.35	-1.37	-11.70	43.60
C/C	1.48	2.08	2.62	15.92	-12.52	46.65
C/C	0.24	1.00	3.77	-9.06	-0.65	47.80
C/C	-0.04	0.13	3.35	-3.86	-5.20	39.92

Table S29: Local base-step parameters for ds-G4 [M06-2X/6-31G(d,p)]

step	Shift	Slide	Rise	Tilt	Roll	Twist
GG/CC	0.41	0.67	3.23	5.37	-4.32	40.28
GG/CC	0.44	1.25	3.48	7.47	-2.74	47.19
GG/CC	-1.06	1.81	3.04	-8.73	-12.22	45.78
ave.	-0.07	1.24	3.25	1.37	-6.43	44.42

Table S30: Local base-step parameters for ss-AACCAA [M06-2X/6-31G(d,p)]

step	Shift	Slide	Rise	Tilt	Roll	Twist
A/A	0.17	1.38	2.90	4.46	10.83	24.71
A/C	0.55	-0.03	2.57	9.53	4.03	34.06
C/C	-0.59	-0.17	2.34	13.48	-6.93	29.25
C/A	0.72	0.74	2.84	6.26	-2.90	47.92
A/A	0.86	-0.01	3.12	-0.72	4.55	48.60
ave.	0.34	0.38	2.75	6.60	1.92	36.91

Table S31: Local base-step parameters for ss-T6 (M06-2X/TZVP)

step	Shift	Slide	Rise	Tilt	Roll	Twist
T/T	-0.74	-0.20	2.55	12.31	-19.36	27.76
T/T	-0.47	-0.20	2.33	14.23	-12.72	30.45
T/T	-0.43	-0.18	2.31	14.55	-10.94	30.76
T/T	-0.40	-0.18	2.32	14.38	-11.19	31.01
T/T	-0.17	0.04	2.49	13.16	-16.43	38.04
ave.	-0.44	-0.15	2.40	13.73	-14.13	31.60

Table S32: Local base-step parameters for ss-A6 (M06-2X/TZVP)

step	Shift	Slide	Rise	Tilt	Roll	Twist
A/A	0.20	1.84	3.14	1.88	0.25	41.49
A/A	0.92	-0.01	3.16	-0.73	6.50	46.26
A/A	0.69	0.11	3.17	-0.13	4.49	41.81
A/A	0.72	0.07	3.18	-0.87	5.25	43.94
A/A	0.76	0.04	3.24	-2.62	5.46	45.97
ave.	0.66	0.41	3.18	-0.49	4.39	43.90

Table S33: Local base-step parameters for the individual single strands composing the ds-A4 sequence optimized at the M06-2X/TZVP level

step	Shift	Slide	Rise	Tilt	Roll	Twist
A/A	0.20	1.72	2.90	5.88	1.38	42.41
A/A	0.42	2.03	2.78	10.54	-1.32	47.36
A/A	0.33	1.90	3.14	0.74	-7.59	47.96
T/T	0.70	0.87	3.20	4.90	-22.25	49.82
T/T	0.53	0.65	3.18	3.61	-18.55	44.66
T/T	0.43	0.31	2.90	5.30	-8.77	40.89

Table S34: Local base-step parameters for the ds-A4 sequence (M06-2X/TZVP)

step	Shift	Slide	Rise	Tilt	Roll	Twist
AA/TT	0.13	1.00	3.00	-0.22	-3.74	42.48
AA/TT	0.18	1.37	3.26	2.11	-15.20	49.69
AA/TT	0.05	1.36	3.09	-3.31	-10.08	47.49
ave.	0.12	1.24	3.12	-0.47	-9.67	46.56

Table S35: Local base-step parameters for ss-C6 (M06-2X/TZVP)

step	Shift	Slide	Rise	Tilt	Roll	Twist
C/C	-0.03	-0.27	2.93	3.89	-5.98	38.19
C/C	0.16	-0.30	3.55	-4.67	4.77	39.57
C/C	0.02	-0.14	3.32	-2.23	-1.10	38.99
C/C	-0.03	-0.14	3.25	-0.98	-2.02	37.55
C/C	0.09	-0.68	4.04	-12.81	8.38	39.16
ave.	0.04	-0.31	3.42	-3.36	0.81	38.69

Table S36: Local base-step parameters for ss-G6 (M06-2X/TZVP)

step	Shift	Slide	Rise	Tilt	Roll	Twist
G/G	1.38	1.01	2.95	6.85	-0.22	40.02
G/G	1.27	0.66	3.04	5.99	-4.01	47.52
G/G	1.42	0.09	2.94	7.52	-4.01	54.70
G/G	1.03	1.20	3.08	4.52	-4.58	44.88
G/G	1.32	0.74	3.08	3.83	-3.08	48.92
ave.	1.28	0.74	3.02	5.74	-3.18	47.21

Table S37: Local base-step parameters for the individual single strands composing the ds-G4 sequence optimized at the M06-2X/TZVP level

step	Shift	Slide	Rise	Tilt	Roll	Twist
G/G	0.48	1.73	3.10	3.02	-3.70	45.30
G/G	1.03	1.31	3.03	11.44	-4.91	41.53
G/G	0.50	1.77	3.25	-0.36	-7.43	50.53
C/C	0.63	1.86	3.08	10.74	-9.22	51.83
C/C	0.14	0.71	3.89	-10.65	-6.09	41.77
C/C	0.09	0.49	3.71	-8.94	-9.93	43.23

Table S38: Local base-step parameters for the ds-G4 sequence (M06-2X/TZVP)

step	Shift	Slide	Rise	Tilt	Roll	Twist
GG/CC	0.21	1.15	3.47	5.98	-6.89	44.49
GG/CC	0.50	1.05	3.52	11.14	-5.44	41.71
GG/CC	-0.08	1.81	3.22	-5.64	-8.38	51.65
ave.	0.21	1.33	3.40	3.83	-6.90	45.95

Table S39: Local base-step parameters for ss-AACCAA (M06-2X/TZVP)

step	Shift	Slide	Rise	Tilt	Roll	Twist
A/A	0.25	1.85	3.08	2.47	2.24	41.54
A/C	0.82	-0.30	3.04	2.03	5.77	40.32
C/C	0.25	-0.35	3.52	-4.61	8.64	39.45
C/A	0.75	0.86	2.98	5.70	-1.04	46.30
A/A	0.77	0.10	3.28	-3.19	5.98	43.72
ave.	0.57	0.43	3.18	0.48	4.32	42.27

Table S40: Local base-step parameters for ss-C4 [ONIOM-EE /B3LYP-D3/6-31G(d,p)]

step	Shift	Slide	Rise	Tilt	Roll	Twist
C/C	0.45	-0.77	3.37	-1.36	1.64	32.04
C/C	0.37	-0.70	3.04	3.03	-2.10	40.81
C/C	0.72	0.27	2.90	11.14	1.37	37.66
ave.	0.51	-0.40	3.10	4.27	0.30	36.84

Table S41: Local base-step parameters for ss-C4 [ONIOM-ME /B3LYP-D3/6-31G(d,p)]

step	Shift	Slide	Rise	Tilt	Roll	Twist
C/C	-0.54	-0.17	2.65	9.83	3.55	17.22
C/C	0.78	0.46	2.85	5.33	3.72	40.61
C/C	1.18	0.69	1.95	24.53	3.95	30.68
ave.	0.47	0.33	2.49	13.23	3.74	29.50

Table S42: Local base-step parameters for ss-C4 [ONIOM-EE /B3LYP-D3/TZVP]

step	Shift	Slide	Rise	Tilt	Roll	Twist
C/C	0.41	-0.68	3.43	-1.84	4.50	29.26
C/C	0.60	-0.65	3.18	1.21	1.54	42.70
C/C	0.68	0.21	2.91	11.54	0.66	37.66
ave.	0.56	-0.37	3.17	3.64	2.23	36.54

Table S43: Local base-step parameters for ss-C4 [ONIOM-ME /B3LYP-D3/TZVP]

step	Shift	Slide	Rise	Tilt	Roll	Twist
C/C	0.15	0.18	2.71	11.61	-5.64	33.27
C/C	-1.58	0.11	3.37	-2.12	-1.46	25.47
C/C	0.90	0.13	2.18	20.22	0.56	39.05
ave.	-0.18	0.14	2.75	9.90	-2.18	32.60

Table S44: Local base-step parameters for ss-C4 [ONIOM-EE /M06-2X/6-31G(d,p)]

step	Shift	Slide	Rise	Tilt	Roll	Twist
C/C	0.31	-0.63	3.18	0.89	-0.48	32.12
C/C	0.46	-0.78	3.21	-1.15	-0.01	41.21
C/C	0.58	0.23	2.71	12.94	0.50	38.23
ave.	0.45	-0.39	3.03	4.23	0.00	37.19

Table S45: Local base-step parameters for ss-C4 [ONIOM-ME /M06-2X/6-31G(d,p)]

step	Shift	Slide	Rise	Tilt	Roll	Twist
C/C	-0.65	-1.05	1.87	20.72	-12.28	24.75
C/C	-1.29	0.31	2.86	5.36	-9.92	24.89
C/C	1.32	-0.18	2.49	12.41	2.74	41.01
ave.	-0.21	-0.31	2.41	12.83	-6.49	30.22

Table S46: Local base-step parameters for ss-C4 [ONIOM-EE/M06-2X/TZVP]

step	Shift	Slide	Rise	Tilt	Roll	Twist
C/C	0.50	-0.72	3.20	2.00	-2.76	32.88
C/C	0.57	-0.93	3.46	-5.04	2.40	43.43
C/C	0.21	0.24	2.64	14.16	-5.19	38.35
ave.	0.43	-0.47	3.10	3.71	-1.85	38.22

Table S47: Local base-step parameters for ss-C4 [ONIOM-ME/M06-2X/TZVP]

step	Shift	Slide	Rise	Tilt	Roll	Twist
C/C	-1.61	0.30	2.97	6.11	-13.02	29.25
C/C	-0.34	-0.16	2.97	2.35	-2.50	27.91
C/C	0.67	0.21	2.38	14.15	-0.27	41.89
ave.	-0.43	0.12	2.77	7.54	-5.26	33.02

Predicted sugar conformational parameters of double strands

```
Note: v0: C4'-O4'-C1'-C2'
      v1: O4'-C1'-C2'-C3'
      v2: C1'-C2'-C3'-C4'
      v3: C2'-C3'-C4'-O4'
      v4: C3'-C4'-O4'-C1'

      tm: the amplitude of pucker
      P:  the phase angle of pseudorotation; angles are given in degrees
```

ds-G4 M06-2X/6-31G(d,p)

Strand I

base	v0	v1	v2	v3	v4	tm	P	Puckering
1 G	-11.9	31.6	-38.4	32.7	-13.2	38.4	181.2	C3'-exo
2 G	-36.5	46.0	-38.1	18.1	11.3	45.3	147.1	C2'-endo
3 G	-33.7	43.2	-36.3	18.0	9.6	42.6	148.5	C2'-endo
4 G	-10.1	28.2	-34.5	29.5	-12.3	34.5	181.9	C3'-exo

Strand II

base	v0	v1	v2	v3	v4	tm	P	Puckering
1 C	-22.6	35.7	-34.5	22.6	-0.1	36.4	161.5	C2'-endo
2 C	-16.7	31.0	-33.0	24.0	-4.7	33.5	169.4	C2'-endo
3 C	-42.3	48.9	-37.1	13.9	17.4	48.2	140.4	C1'-exo
4 C	-16.1	29.9	-31.6	23.0	-4.5	32.2	169.2	C2'-endo

ds-A4 M06-2X/6-31G(d,p)

Strand I

base	v0	v1	v2	v3	v4	tm	P	Puckering
1 T	-20.5	37.5	-39.5	29.0	-5.4	40.3	169.0	C2'-endo
2 T	-41.4	47.9	-36.2	13.5	17.2	47.2	140.2	C1'-exo
3 T	-39.3	47.3	-37.2	15.7	14.6	46.5	143.3	C1'-exo
4 T	-43.6	32.9	-10.7	-14.7	36.5	42.9	104.5	O4'-endo

Strand II

base	v0	v1	v2	v3	v4	tm	P	Puckering
1 A	-14.4	31.1	-35.0	27.8	-8.6	35.1	175.2	C2'-endo
2 A	-36.6	45.2	-36.3	16.1	12.7	44.3	144.9	C2'-endo
3 A	-38.5	46.8	-37.2	16.1	13.7	45.9	144.1	C2'-endo
4 A	-25.7	39.9	-38.5	24.9	0.3	40.7	160.9	C2'-endo

ds-G4 M06-2X/TZVP

Strand I

base	v0	v1	v2	v3	v4	tm	P	Puckering
1 G	-22.4	37.9	-38.6	26.8	-3.0	39.8	165.6	C2'-endo
2 G	-13.5	31.1	-36.0	29.2	-10.0	36.1	177.2	C2'-endo
3 G	-36.9	45.6	-37.1	16.9	12.2	44.9	145.7	C2'-endo
4 G	-10.8	27.9	-33.6	28.1	-11.0	33.6	180.2	C3'-exo

Strand II

base	v0	v1	v2	v3	v4	tm	P	Puckering
1 C	-16.3	31.0	-33.2	24.8	-5.5	33.7	170.6	C2'-endo
2 C	-11.3	27.3	-32.0	26.1	-9.5	32.0	178.3	C2'-endo
3 C	3.5	14.7	-25.8	28.3	-20.3	28.5	205.1	C3'-exo
4 C	-17.1	30.2	-31.3	22.1	-3.3	32.1	167.1	C2'-endo

****

ds-A4 M06-2X/TZVP

****

Strand I

base	v0	v1	v2	v3	v4	tm	P	Puckering
1 T	-13.9	28.8	-31.9	24.4	-6.8	32.1	173.3	C2'-endo
2 T	-10.7	23.7	-26.8	21.1	-6.7	26.9	175.5	C2'-endo
3 T	-12.0	26.2	-29.6	23.3	-7.3	29.7	175.2	C2'-endo
4 T	-21.2	33.6	-32.7	21.5	-0.3	34.4	161.8	C2'-endo

Strand II

base	v0	v1	v2	v3	v4	tm	P	Puckering
1 A	-10.9	28.1	-33.7	28.2	-11.0	33.7	180.2	C3'-exo
2 A	-7.9	28.5	-37.2	33.8	-16.3	37.5	186.8	C3'-exo
3 A	-13.8	31.9	-37.0	30.1	-10.3	37.0	177.3	C2'-endo
4 A	-22.8	38.0	-38.4	26.5	-2.5	39.8	164.9	C2'-endo

****

****

ds-G4 B3LYP-D3/6-31G(d,p)

****

Strand I

base	v0	v1	v2	v3	v4	tm	P	Puckering
1 G	-8.8	25.1	-30.6	26.2	-11.0	30.6	182.0	C3'-exo
2 G	-38.4	45.0	-34.5	13.0	15.7	44.4	141.0	C1'-exo
3 G	-38.2	46.0	-36.3	15.0	14.3	45.3	143.2	C1'-exo
4 G	-38.8	44.2	-32.7	11.1	17.1	43.6	138.5	C1'-exo

Strand II

base	v0	v1	v2	v3	v4	tm	P	Puckering
1 C	-16.9	31.3	-33.1	24.1	-4.6	33.7	169.1	C2'-endo
2 C	-36.9	45.7	-37.2	16.8	12.3	45.0	145.7	C2'-endo
3 C	-38.5	45.9	-36.0	14.6	14.7	45.3	142.7	C1'-exo
4 C	-15.9	34.1	-38.5	30.3	-9.1	38.6	174.8	C2'-endo

****

****

ds-A4 B3LYP-D3/6-31G(d,p)

****

Strand I

base	v0	v1	v2	v3	v4	tm	P	Puckering
1 T	-13.5	30.8	-35.5	28.5	-9.5	35.6	176.7	C2'-endo
2 T	-39.1	45.9	-35.5	13.8	15.6	45.3	141.5	C1'-exo
3 T	-39.7	46.0	-34.7	12.5	16.8	45.4	139.9	C1'-exo
4 T	-42.7	44.4	-29.2	5.3	23.1	44.9	130.5	C1'-exo

Strand II

base	v0	v1	v2	v3	v4	tm	P	Puckering
1 A	-10.7	26.4	-31.1	25.5	-9.5	31.1	178.8	C2'-endo
2 A	-36.5	44.4	-35.3	15.1	13.1	43.6	144.0	C2'-endo
3 A	-37.3	44.4	-34.7	14.0	14.3	43.8	142.5	C1'-exo
4 A	-6.8	21.0	-26.2	22.8	-10.2	26.3	183.6	C3'-exo

****

****

ds-G4 B3LYP-3D/TZVP

****

Strand I

base	v0	v1	v2	v3	v4	tm	P	Puckering
1 G	-17.5	34.4	-37.6	28.3	-7.0	38.0	171.9	C2'-endo
2 G	-37.2	44.7	-35.1	14.4	14.0	43.9	143.0	C1'-exo
3 G	-14.2	32.2	-37.2	29.8	-9.9	37.3	176.7	C2'-endo
4 G	-8.1	25.9	-32.8	28.8	-13.2	32.9	184.6	C3'-exo

Strand II

base	v0	v1	v2	v3	v4	tm	P	Puckering
1 C	-13.1	29.0	-33.1	26.2	-8.4	33.2	175.8	C2'-endo
2 C	-11.3	26.9	-31.4	25.5	-9.1	31.4	177.9	C2'-endo
3 C	-1.3	20.5	-30.6	30.4	-18.4	31.8	196.0	C3'-exo
4 C	-12.2	26.4	-29.8	23.2	-7.1	29.9	174.8	C2'-endo

****

****

ds-A4 B3LYP-3D/TZVP

****

Strand I

base	v0	v1	v2	v3	v4	tm	P	Puckering
1 T	-14.8	32.1	-36.5	28.7	-8.8	36.6	175.2	C2'-endo
2 T	-38.6	44.7	-33.9	12.3	16.2	44.2	140.2	C1'-exo
3 T	-39.8	44.9	-32.9	10.6	18.0	44.5	137.7	C1'-exo
4 T	-20.9	33.2	-32.2	20.9	-0.1	33.9	161.5	C2'-endo

Strand II

base	v0	v1	v2	v3	v4	tm	P	Puckering
1 A	-7.8	25.2	-32.1	28.2	-12.9	32.2	184.7	C3'-exo
2 A	-11.6	30.0	-36.0	30.1	-11.7	36.0	180.1	C3'-exo
3 A	-13.4	31.7	-37.2	30.3	-10.7	37.2	178.0	C2'-endo
4 A	-26.5	40.1	-38.1	23.8	1.5	40.7	159.3	C2'-endo

****

Predicted (ONIOM) sugar conformational parameters of ss-C4

```
Note: v0: C4'-O4'-C1'-C2'
      v1: O4'-C1'-C2'-C3'
      v2: C1'-C2'-C3'-C4'
      v3: C2'-C3'-C4'-O4'
      v4: C3'-C4'-O4'-C1'

      tm: the amplitude of pucker
      P:  the phase angle of pseudorotation; angles are given in degrees
```

****

****

ONIOM-EE B3LYP-D3/6-31G(d,p)

****

base	v0	v1	v2	v3	v4	tm	P	Puckering
1 C	-21.4	34.8	-33.5	22.6	-1.0	35.2	162.5	C2'-endo
2 C	-38.7	41.8	-28.4	6.8	20.1	41.7	133.0	C1'-exo
3 C	-22.4	32.8	-29.3	17.3	3.0	32.2	155.6	C2'-endo
4 C	-28.4	40.2	-35.5	21.0	4.4	39.3	154.6	C2'-endo

****

****

ONIOM-ME B3LYP-D3/6-31G(d,p)

****

base	v0	v1	v2	v3	v4	tm	P	Puckering
1 C	-12.1	31.0	-36.4	30.3	-12.0	36.4	179.6	C2'-endo
2 C	-26.7	42.1	-39.9	26.3	-0.2	42.2	161.0	C2'-endo
3 C	-39.6	29.8	-9.4	-13.5	33.3	38.9	104.0	O4'-endo
4 C	-31.7	44.8	-40.0	23.1	5.2	44.3	154.5	C2'-endo

****

****

ONIOM-EE B3LYP-D3/TZVP

****

base	v0	v1	v2	v3	v4	tm	P	Puckering
1 C	-21.2	34.5	-33.3	22.5	-1.0	34.9	162.6	C2'-endo
2 C	-39.8	43.3	-29.5	7.3	20.4	43.0	133.4	C1'-exo
3 C	-22.0	33.0	-30.0	18.4	2.1	32.5	157.2	C2'-endo
4 C	-28.0	40.0	-35.7	21.5	3.8	39.3	155.4	C2'-endo

****

****

ONIOM-ME B3LYP-D3/TZVP

****

base	v0	v1	v2	v3	v4	tm	P	Puckering
1 C	-7.9	26.2	-33.1	29.4	-13.9	33.2	185.2	C3'-exo
2 C	-21.7	38.2	-39.1	28.0	-4.3	40.1	167.1	C2'-endo
3 C	-30.4	39.2	-32.4	15.6	9.0	38.3	147.8	C2'-endo
4 C	-11.0	28.5	-33.6	28.3	-11.1	33.6	179.9	C2'-endo

****

****
ONIOM-EE M06-2X/6-31G(d,p)
****

base	v0	v1	v2	v3	v4	tm	P	Puckering
1 C	-27.4	36.4	-30.7	15.9	7.1	35.5	149.9	C2'-endo
2 C	-38.5	42.2	-29.2	7.7	19.4	41.9	134.2	C1'-exo
3 C	-22.8	33.3	-29.9	17.6	3.1	32.8	155.7	C2'-endo
4 C	-26.6	39.2	-35.7	22.4	2.4	38.7	157.4	C2'-endo

****

****
ONIOM-ME M06-2X/6-31G(d,p)
****

base	v0	v1	v2	v3	v4	tm	P	Puckering
1 C	-18.0	33.2	-34.5	25.0	-4.8	35.2	168.6	C2'-endo
2 C	-27.2	39.5	-35.3	21.3	3.4	38.7	155.8	C2'-endo
3 C	-41.2	43.3	-28.1	5.0	22.5	43.4	130.3	C1'-exo
4 C	-14.2	33.5	-38.3	31.9	-11.2	38.4	177.8	C2'-endo

****

****
ONIOM-EE M06-2X/TZVP
****

base	v0	v1	v2	v3	v4	tm	P	Puckering
1 C	-24.6	36.1	-32.6	19.6	3.0	35.6	156.3	C2'-endo
2 C	-37.6	43.8	-32.2	11.5	16.3	42.7	139.0	C1'-exo
3 C	-21.0	32.9	-30.7	19.5	0.8	32.8	159.6	C2'-endo
4 C	-21.3	35.9	-35.3	25.1	-2.6	36.6	164.8	C2'-endo

****

****
ONIOM-ME M06-2X/TZVP
****

base	v0	v1	v2	v3	v4	tm	P	Puckering
1 C	-11.7	26.3	-29.8	23.8	-8.0	29.9	176.1	C2'-endo
2 C	1.2	18.8	-30.0	31.7	-21.0	32.1	200.8	C3'-exo
3 C	-27.4	39.0	-34.7	20.1	4.3	38.4	154.6	C2'-endo
4 C	1.5	19.2	-30.9	32.8	-21.7	33.2	201.1	C3'-exo

****

DFT optimized geometries

```
ss-A6 B3LYP-D3/6-31G(d,p)
O        11.99417       -4.55600       -0.15851
C        12.51285       -3.37469       -0.75914
C        11.44401       -2.29521       -0.75350
O        10.37420       -2.65337       -1.65135
C        10.78212       -2.06780        0.61403
O        10.44335       -0.66947        0.68287
C         9.52789       -2.92934        0.52489
C         9.13971       -2.71221       -0.93219
N         8.31196       -3.75675       -1.50193
C         8.00098       -5.00592       -0.99742
N         7.20478       -5.70476       -1.77702
C         6.98258       -4.87663       -2.86870
C         6.19097       -5.01030       -4.02587
N         5.40107       -6.09485       -4.24231
N         6.17107       -3.99939       -4.91540
C         6.86834       -2.88662       -4.63329
N         7.62771       -2.62353       -3.56324
C         7.65388       -3.65985       -2.71396
P         9.91397       -0.04900        2.09978
O        10.99241       -0.11781        3.16173
O         8.59509       -0.67522        2.52984
O         9.71674        1.49998        1.64807
C         9.57045        1.99241        0.30619
C         8.12279        2.11145       -0.13996
O         7.62042        0.83201       -0.56918
C         7.15922        2.60992        0.96014
O         6.30066        3.59996        0.35368
C         6.37683        1.35307        1.32360
C         6.29362        0.68877       -0.04236
```

N	5.92156	-0.70122	-0.06163
C	6.20247	-1.69213	0.86414
N	5.72265	-2.87027	0.52098
C	5.09581	-2.65170	-0.70144
C	4.38713	-3.48598	-1.58671
N	4.14706	-4.80606	-1.32650
N	3.88148	-2.94973	-2.71303
C	4.05732	-1.63923	-2.93854
N	4.70327	-0.74452	-2.18179
C	5.20789	-1.30834	-1.07831
P	5.36246	4.51531	1.33092
O	6.19989	5.45274	2.17684
O	4.40149	3.65881	2.14404
O	4.58009	5.39110	0.20794
C	4.38196	5.05235	-1.17437
C	3.04668	4.37785	-1.44746
O	3.13199	2.96666	-1.17383
C	1.87613	4.91474	-0.59470
O	0.73851	5.05086	-1.47463
C	1.65455	3.79573	0.41617
C	1.94234	2.58855	-0.46318
N	2.16962	1.33703	0.20719
C	2.84729	1.09706	1.38995
N	2.93802	-0.18476	1.68195
C	2.28643	-0.82910	0.63546
C	2.04236	-2.18305	0.33711
N	2.45634	-3.20401	1.14458
N	1.34379	-2.48344	-0.77380
C	0.91815	-1.48233	-1.55759
N	1.10506	-0.16652	-1.40146
C	1.79895	0.10162	-0.28898
P	-0.58138	5.84350	-0.92211
O	-0.30187	7.32296	-0.75461
O	-1.14254	5.18541	0.33090
O	-1.56757	5.64361	-2.19779
C	-1.50366	4.60609	-3.19050
C	-2.41106	3.42134	-2.89590
O	-1.76042	2.50113	-1.99885
C	-3.75635	3.79409	-2.23429
O	-4.77843	3.00338	-2.88194
C	-3.55522	3.34887	-0.79078
C	-2.72944	2.09235	-1.01980
N	-2.04931	1.54535	0.12271
C	-1.41239	2.21404	1.15358
N	-0.81948	1.40297	2.00639
C	-1.07618	0.12838	1.51276
C	-0.71226	-1.16302	1.93814
N	0.05251	-1.38599	3.04729
N	-1.16226	-2.22319	1.24189
C	-1.92017	-2.00623	0.15693
N	-2.29825	-0.83727	-0.37249
C	-1.84546	0.19693	0.34601
P	-6.34826	3.33515	-2.56159
O	-6.76388	4.66023	-3.16649
O	-6.64740	3.22645	-1.07236
O	-7.05667	2.13778	-3.39992
C	-6.47595	0.88383	-3.79488
C	-6.81310	-0.26856	-2.85948
O	-5.90107	-0.29885	-1.74463
C	-8.23112	-0.20827	-2.25222
O	-8.74356	-1.55848	-2.25722
C	-7.96126	0.26355	-0.82859
C	-6.67361	-0.49946	-0.54889
N	-5.90076	-0.07989	0.58881
C	-5.67042	1.20391	1.04852
N	-4.86637	1.24293	2.09261
C	-4.54843	-0.08840	2.33728
C	-3.72900	-0.72431	3.28846
N	-3.01588	-0.03359	4.22777
N	-3.66907	-2.06867	3.29448
C	-4.36406	-2.74702	2.36890
N	-5.12962	-2.26277	1.38480
C	-5.18894	-0.92641	1.41764
P	-10.31041	-1.82479	-1.86732
O	-11.23776	-1.24577	-2.91596
O	-10.62677	-1.36230	-0.45149
O	-10.33832	-3.44419	-1.98863
C	-9.22494	-4.33465	-1.81477

C	-9.06625	-4.86197	-0.39917
O	-8.37778	-3.90052	0.42090
C	-10.38470	-5.21120	0.33265
O	-10.22178	-6.52836	0.86152
C	-10.45240	-4.14886	1.43406
C	-8.96820	-3.94481	1.72372
N	-8.60763	-2.74744	2.44007
C	-8.91708	-1.44329	2.09097
N	-8.31587	-0.54890	2.84705
C	-7.57084	-1.29926	3.74841
C	-6.66673	-0.95158	4.77063
N	-6.34339	0.34152	5.05982
N	-6.10485	-1.93737	5.49890
C	-6.38508	-3.20988	5.17870
N	-7.16739	-3.67049	4.19517
C	-7.73769	-2.66975	3.51131
H	13.38781	-2.99531	-0.21145
H	12.81709	-3.55382	-1.79956
H	11.89636	-1.35838	-1.09698
H	11.43378	-2.33156	1.45030
H	9.82649	-3.96489	0.69022
H	8.59795	-1.77008	-1.06571
H	8.37451	-5.33986	-0.04235
H	6.80384	-2.09498	-5.37626
H	5.61048	-6.94121	-3.73358
H	5.01736	-6.20493	-5.16999
H	8.75978	-2.62994	1.23266
H	10.01429	2.99160	0.31203
H	10.12272	1.36207	-0.39266
H	8.08708	2.81372	-0.98240
H	7.68925	3.05363	1.80498
H	6.98045	0.73780	1.99415
H	5.57445	1.20336	-0.68924
H	6.80004	-1.47367	1.73857
H	3.61865	-1.25857	-3.85761
H	4.83773	-5.26497	-0.74552
H	3.88773	-5.34398	-2.14569
H	5.41323	1.58368	1.76545
H	4.40869	6.00271	-1.71436
H	5.19507	4.41761	-1.53072
H	2.80631	4.52447	-2.50798
H	2.10285	5.87990	-0.13790
H	2.40969	3.86757	1.20202
H	1.12244	2.40734	-1.16649
H	3.26871	1.91765	1.95564
H	0.35270	-1.78229	-2.43623
H	3.25992	-2.99816	1.72441
H	2.51408	-4.10016	0.67405
H	0.65331	3.81306	0.83250
H	-1.83452	5.07428	-4.12139
H	-0.47581	4.25997	-3.31285
H	-2.61561	2.91161	-3.84577
H	-3.99254	4.85471	-2.33898
H	-2.95491	4.09430	-0.26491
H	-3.34791	1.28100	-1.41791
H	-1.41037	3.29553	1.18589
H	-2.26289	-2.89768	-0.36191
H	0.66790	-0.62787	3.31234
H	0.49062	-2.29879	3.07109
H	-4.49699	3.17417	-0.28250
H	-6.89719	0.66557	-4.78003
H	-5.39241	0.97778	-3.88589
H	-6.71126	-1.20069	-3.42892
H	-8.90325	0.44226	-2.81494
H	-7.76242	1.33734	-0.83492
H	-6.87303	-1.56574	-0.40054
H	-6.11376	2.05268	0.54391
H	-4.28463	-3.82965	2.42286
H	-2.77261	0.91574	3.97519
H	-2.24831	-0.55868	4.62900
H	-8.77732	0.02313	-0.15588
H	-9.42600	-5.18251	-2.47529
H	-8.30013	-3.85053	-2.13444
H	-8.46264	-5.77788	-0.45679
H	-11.24504	-5.16319	-0.34207
H	-10.87258	-3.22374	1.03104
H	-8.56114	-4.78059	2.30359
H	-9.58711	-1.25002	1.26121

H	-5.89934	-3.96457	5.79323
H	-6.48137	1.00493	4.30808
H	-5.46350	0.45205	5.54870
H	-11.02290	-4.47556	2.30609
H	-11.04168	-6.75955	1.31963
H	12.68982	-5.22565	-0.17213

****

ss-C6 B3LYP-D3/6-31G(d,p)

O	11.95513	-4.96098	-0.58174
C	12.52073	-3.93052	-1.39014
C	11.53995	-2.77352	-1.50277
O	10.39730	-3.16572	-2.28372
C	10.98124	-2.28839	-0.15777
O	10.70880	-0.87662	-0.29566
C	9.68658	-3.07584	-0.03166
C	9.20808	-3.11717	-1.48262
N	8.35702	-4.25211	-1.81171
C	7.16682	-4.02793	-2.56995
O	6.93788	-2.89975	-3.01000
N	6.33258	-5.08975	-2.76051
C	6.67947	-6.30065	-2.34095
N	5.77530	-7.29430	-2.49383
C	7.94559	-6.58220	-1.72837
C	8.75072	-5.51754	-1.48754
P	10.37056	-0.01427	1.05332
O	11.60197	0.15905	1.91865
O	9.18044	-0.59885	1.80212
O	10.01943	1.41907	0.37500
C	9.51222	1.64772	-0.95020
C	7.99454	1.69829	-1.02918
O	7.45441	0.37154	-1.12615
C	7.30522	2.36333	0.18665
O	6.33858	3.30034	-0.33396
C	6.62233	1.18706	0.87882
C	6.25101	0.34548	-0.33630
N	5.85773	-1.02998	-0.11879
C	4.85696	-1.58737	-0.97995
O	4.32909	-0.86808	-1.82872
N	4.52716	-2.89725	-0.80167
C	5.15126	-3.63209	0.10491
N	4.76003	-4.92837	0.23854
C	6.17233	-3.11141	0.96229
C	6.49945	-1.80336	0.80096
P	5.63113	4.34808	0.70143
O	6.64792	5.30225	1.29645
O	4.79420	3.62560	1.74904
O	4.69868	5.17106	-0.34458
C	4.23025	4.71219	-1.62288
C	2.88695	4.00490	-1.56529
O	3.05836	2.63921	-1.15873
C	1.86708	4.63058	-0.58321
O	0.62231	4.76940	-1.30033
C	1.75870	3.57943	0.51785
C	1.94625	2.31118	-0.30680
N	2.23081	1.08043	0.39962
C	1.65110	-0.13094	-0.09698
O	0.91909	-0.08465	-1.08651
N	1.93123	-1.28547	0.57054
C	2.75276	-1.28259	1.60844
N	2.95765	-2.46683	2.24705
C	3.38690	-0.09606	2.09638
C	3.10135	1.06260	1.44783
P	-0.57883	5.64595	-0.62230
O	-0.17992	7.10156	-0.48170
O	-1.06559	5.02338	0.67982
O	-1.69605	5.51976	-1.79501
C	-1.76427	4.50960	-2.81472
C	-2.62998	3.32045	-2.43392
O	-1.89200	2.40796	-1.60643
C	-3.91050	3.68726	-1.64746
O	-5.00432	2.96880	-2.25734
C	-3.61688	3.15917	-0.24680
C	-2.79742	1.92305	-0.59838
N	-2.04219	1.28781	0.46038
C	-1.95216	-0.14154	0.45920
O	-2.52849	-0.77880	-0.42348

N	-1.23821	-0.73087	1.45914
C	-0.61179	0.00439	2.36424
N	0.04891	-0.65341	3.35565
C	-0.64105	1.43510	2.36153
C	-1.36345	2.03188	1.37815
P	-6.52797	3.38489	-1.83904
O	-6.84618	4.80259	-2.27121
O	-6.79490	3.12980	-0.36115
O	-7.35978	2.35414	-2.77879
C	-6.87873	1.13445	-3.36614
C	-7.14872	-0.09458	-2.51458
O	-6.14990	-0.22094	-1.49094
C	-8.51629	-0.08653	-1.79192
O	-9.10886	-1.38525	-1.99521
C	-8.13009	0.11914	-0.33013
C	-6.81794	-0.65807	-0.29391
N	-5.93932	-0.45896	0.83964
C	-5.21530	-1.58971	1.33811
O	-5.36534	-2.68674	0.79838
N	-4.39648	-1.39420	2.41017
C	-4.23885	-0.18541	2.92676
N	-3.43081	-0.06966	4.01552
C	-4.90880	0.97220	2.41758
C	-5.74323	0.78378	1.36201
P	-10.68221	-1.60074	-1.60901
O	-11.56880	-0.68528	-2.43034
O	-10.92177	-1.49359	-0.10901
O	-10.87667	-3.12743	-2.13276
C	-9.83552	-4.10232	-2.29479
C	-9.52569	-4.89334	-1.03841
O	-8.72562	-4.11412	-0.13904
C	-10.75766	-5.38586	-0.23558
O	-10.64223	-6.80638	-0.14127
C	-10.58893	-4.68840	1.11990
C	-9.07033	-4.53898	1.18532
N	-8.54183	-3.58946	2.14451
C	-7.50997	-4.01035	3.03897
O	-7.14041	-5.18743	3.02407
N	-6.98775	-3.07933	3.88730
C	-7.40887	-1.82166	3.84905
N	-6.84133	-0.94261	4.71592
C	-8.44033	-1.37337	2.96479
C	-8.96815	-2.29499	2.11885
H	13.45696	-3.55544	-0.95261
H	12.73901	-4.29478	-2.40221
H	12.04548	-1.94465	-2.01026
H	11.67931	-2.44936	0.66785
H	9.91649	-4.07452	0.33922
H	8.62576	-2.23312	-1.73918
H	9.74157	-5.60992	-1.05587
H	8.24723	-7.58803	-1.46853
H	4.94996	-7.10673	-3.04297
H	6.03441	-8.25567	-2.34024
H	8.98093	-2.59162	0.63386
H	12.54661	-5.72390	-0.61255
H	9.90816	2.62307	-1.24507
H	9.89160	0.88848	-1.63696
H	7.72616	2.26696	-1.92945
H	8.01311	2.89225	0.82724
H	7.36408	0.67103	1.49088
H	5.43031	0.80342	-0.89061
H	7.29898	-1.32812	1.35836
H	6.68448	-3.73157	1.68598
H	4.24535	-5.31700	-0.53933
H	5.37393	-5.56838	0.72020
H	5.77475	1.50434	1.47830
H	4.12243	5.61345	-2.23226
H	4.97149	4.05765	-2.08494
H	2.45188	4.03320	-2.57335
H	2.18607	5.60930	-0.22005
H	2.59122	3.71315	1.21129
H	1.06187	2.10452	-0.91118
H	3.56914	2.00803	1.70305
H	4.08366	-0.11710	2.92380
H	2.70595	-3.29880	1.73089
H	3.76610	-2.55439	2.84498
H	0.80758	3.63449	1.03870
H	-2.21191	5.00178	-3.68230

H	-0.76175	4.16756	-3.07728
H	-2.92793	2.81283	-3.36102
H	-4.12174	4.75777	-1.67323
H	-3.00306	3.89150	0.28105
H	-3.43376	1.14027	-1.01374
H	-1.40766	3.10968	1.25666
H	-0.09636	2.02147	3.08951
H	0.26103	-1.62585	3.17873
H	0.74064	-0.14764	3.88903
H	-4.52750	2.94701	0.30481
H	-7.42212	1.03130	-4.30927
H	-5.81072	1.20977	-3.57803
H	-7.11082	-0.97342	-3.17182
H	-9.18709	0.68347	-2.17688
H	-7.95450	1.18304	-0.16031
H	-7.00227	-1.73246	-0.33686
H	-6.26058	1.60253	0.87138
H	-4.74218	1.95858	2.82962
H	-2.78924	-0.83401	4.17694
H	-3.08338	0.84650	4.25806
H	-8.89133	-0.25903	0.34509
H	-10.20417	-4.79747	-3.05392
H	-8.92663	-3.62463	-2.66569
H	-8.96066	-5.78521	-1.34605
H	-11.69432	-5.10317	-0.72657
H	-11.05974	-3.70296	1.08705
H	-8.59221	-5.49212	1.41804
H	-9.72107	-2.05086	1.37281
H	-8.76608	-0.34189	2.94272
H	-5.96484	-1.22509	5.13202
H	-6.93728	0.04457	4.52955
H	-11.00110	-5.27069	1.94699
H	-11.42595	-7.12970	0.32401

****

ss-G6 B3LYP-D3/6-31G(d,p)

O	10.70709	-5.39826	-0.40572
C	11.70497	-4.40232	-0.61677
C	11.07820	-3.01762	-0.54921
O	10.19771	-2.80511	-1.66921
C	10.22178	-2.76401	0.69861
O	10.27749	-1.34478	0.95618
C	8.83616	-3.18014	0.23236
C	8.84124	-2.71730	-1.22314
N	7.98134	-3.50577	-2.08509
C	7.83429	-4.88605	-2.12590
N	6.90484	-5.27557	-2.96730
C	6.41640	-4.09985	-3.52193
C	5.36025	-3.87780	-4.46306
O	4.59969	-4.67995	-5.00326
N	5.23867	-2.49027	-4.74845
C	5.96826	-1.47554	-4.17807
N	5.70466	-0.20716	-4.60937
N	6.91413	-1.68599	-3.28550
C	7.08806	-2.99482	-2.99679
P	9.63320	-0.72884	2.32891
O	10.59084	-0.85360	3.49567
O	8.25464	-1.30842	2.60458
O	9.56794	0.83989	1.90317
C	9.41217	1.35257	0.57001
C	7.97154	1.64407	0.18226
O	7.31453	0.44720	-0.27179
C	7.09532	2.21551	1.31949
O	6.35164	3.31320	0.75233
C	6.17524	1.04790	1.66096
C	5.98503	0.44774	0.27492
N	5.44288	-0.88511	0.21376
C	5.70026	-1.96552	1.04804
N	5.10201	-3.06585	0.65099
C	4.41348	-2.70253	-0.49812
C	3.56084	-3.46859	-1.35648
O	3.24104	-4.65396	-1.30096
N	3.04267	-2.64727	-2.39662
C	3.27879	-1.30669	-2.56238
N	2.68766	-0.69611	-3.64273
N	4.07678	-0.61466	-1.77987
C	4.61227	-1.35176	-0.78004

P	5.52518	4.32263	1.73651
O	6.46721	5.15772	2.58026
O	4.47372	3.58378	2.55082
O	4.84361	5.27911	0.61092
C	4.64030	4.95731	-0.77404
C	3.27242	4.36501	-1.06576
O	3.24204	2.96680	-0.72655
C	2.10903	5.02607	-0.29224
O	1.04164	5.22559	-1.24169
C	1.73643	3.96349	0.73548
C	1.98412	2.70488	-0.08455
N	2.06302	1.46711	0.64675
C	2.64789	1.22537	1.88400
N	2.55324	-0.03355	2.24896
C	1.87726	-0.65770	1.20930
C	1.44937	-2.01500	1.05578
O	1.56865	-2.97441	1.81445
N	0.78121	-2.18238	-0.18977
C	0.51611	-1.19688	-1.10554
N	-0.12282	-1.57326	-2.26023
N	0.90227	0.05099	-0.95139
C	1.56954	0.26070	0.20645
P	-0.23439	6.15996	-0.82790
O	0.17150	7.61388	-0.69591
O	-0.95315	5.61791	0.39866
O	-1.13401	5.98342	-2.17079
C	-1.03054	4.92741	-3.13940
C	-1.98559	3.77408	-2.88511
O	-1.46437	2.89945	-1.86733
C	-3.39564	4.19861	-2.41409
O	-4.33386	3.38917	-3.14958
C	-3.38424	3.81883	-0.93871
C	-2.55735	2.54113	-1.00479
N	-2.04897	2.04655	0.24910
C	-1.57416	2.77293	1.33437
N	-1.20227	2.00384	2.33304
C	-1.44079	0.70876	1.89401
C	-1.27565	-0.54551	2.56319
O	-0.87171	-0.79097	3.69781
N	-1.67953	-1.61237	1.71388
C	-2.18964	-1.49087	0.44720
N	-2.47071	-2.65648	-0.22269
N	-2.34425	-0.33448	-0.15945
C	-1.96294	0.71842	0.60088
P	-5.93790	3.69303	-3.06729
O	-6.27966	5.00586	-3.74299
O	-6.47445	3.58234	-1.64822
O	-6.46957	2.47049	-4.00347
C	-5.76220	1.24698	-4.25226
C	-6.02968	0.16511	-3.22408
O	-5.33995	0.45602	-1.99818
C	-7.52765	-0.04730	-2.86463
O	-7.84289	-1.42707	-3.17452
C	-7.57801	0.35424	-1.39171
C	-6.17306	-0.01439	-0.93365
N	-5.74468	0.53881	0.32359
C	-5.77276	1.85577	0.76402
N	-5.30097	1.98778	1.98470
C	-4.95731	0.69975	2.37810
C	-4.42230	0.20220	3.60867
O	-4.12902	0.79883	4.64397
N	-4.25702	-1.21105	3.54283
C	-4.57477	-2.01692	2.47814
N	-4.30718	-3.35765	2.57488
N	-5.06387	-1.54669	1.35041
C	-5.23123	-0.20808	1.35678
P	-8.64864	-2.49243	-2.24079
O	-8.88139	-3.72279	-3.08894
O	-9.86245	-1.88260	-1.56566
O	-7.59721	-2.81247	-1.02649
C	-6.57378	-3.80677	-1.14998
C	-6.80389	-4.92215	-0.14401
O	-6.69163	-4.43048	1.20535
C	-8.18109	-5.58855	-0.20848
O	-7.99761	-6.93656	0.22189
C	-9.01122	-4.77215	0.79081
C	-7.97915	-4.26251	1.80754
N	-8.19109	-2.87885	2.21490

C	-8.79513	-1.82653	1.53054
N	-8.71654	-0.68794	2.17938
C	-8.01655	-0.98421	3.34002
C	-7.59349	-0.13409	4.41151
O	-7.77523	1.06870	4.58465
N	-6.84557	-0.88026	5.36615
C	-6.53887	-2.21459	5.28470
N	-5.66770	-2.71082	6.21342
N	-6.96730	-2.99992	4.31613
C	-7.67819	-2.33546	3.37328
H	12.49457	-4.46475	0.14597
H	12.17539	-4.51445	-1.60244
H	11.88416	-2.27806	-0.60146
H	10.57739	-3.31742	1.57188
H	8.75570	-4.26472	0.29331
H	8.48912	-1.68694	-1.31804
H	8.47199	-5.52620	-1.53371
H	4.49827	-2.25773	-5.39999
H	6.07322	0.50048	-3.98754
H	4.74916	-0.02825	-4.89493
H	8.04950	-2.71836	0.81680
H	9.96873	2.29367	0.55486
H	9.85683	0.66700	-0.15406
H	7.99191	2.37368	-0.63742
H	7.68663	2.56981	2.16609
H	6.71439	0.34058	2.29538
H	5.32485	1.07167	-0.33609
H	6.36861	-1.86151	1.89048
H	2.42596	-3.12694	-3.04173
H	2.62395	0.30675	-3.51223
H	1.79798	-1.09437	-3.92348
H	5.25333	1.37518	2.13099
H	4.72623	5.90578	-1.31160
H	5.41992	4.27905	-1.12473
H	3.08339	4.47934	-2.14090
H	2.39340	5.98342	0.14920
H	2.44359	4.00265	1.56736
H	1.19805	2.56335	-0.83347
H	3.15077	2.02095	2.41663
H	0.44265	-3.12191	-0.36107
H	-0.56870	-0.78809	-2.71907
H	-0.74437	-2.36924	-2.17196
H	0.71410	4.06983	1.08516
H	-1.28773	5.38332	-4.09955
H	-0.00605	4.55469	-3.18681
H	-2.08706	3.21453	-3.82395
H	-3.59813	5.25628	-2.59547
H	-2.83932	4.57910	-0.37456
H	-3.13501	1.72284	-1.44501
H	-1.50313	3.85081	1.28331
H	-1.59131	-2.53408	2.12542
H	-3.10592	-2.50221	-0.99617
H	-2.76211	-3.43230	0.36259
H	-4.38483	3.67833	-0.54314
H	-6.11896	0.89137	-5.22295
H	-4.68972	1.43792	-4.31823
H	-5.65318	-0.78033	-3.63728
H	-8.18464	0.57012	-3.47847
H	-7.68733	1.44117	-1.34513
H	-6.07770	-1.09527	-0.82078
H	-6.13082	2.63891	0.10865
H	-3.89021	-1.62733	4.39188
H	-4.96835	-3.89913	2.01486
H	-4.23011	-3.71801	3.51780
H	-8.37018	-0.12761	-0.82392
H	-6.55260	-4.22116	-2.16137
H	-5.61905	-3.32322	-0.92850
H	-6.02164	-5.67545	-0.28140
H	-8.60102	-5.54216	-1.21932
H	-9.50050	-3.94670	0.27833
H	-7.97557	-4.84342	2.73125
H	-9.25749	-1.95391	0.56292
H	-6.40716	-0.31380	6.08314
H	-5.63644	-3.72049	6.25430
H	-5.61509	-2.25530	7.11422
H	-9.77930	-5.37991	1.27319
H	-8.86717	-7.35930	0.23381
H	11.11630	-6.26302	-0.53799

****

ss-T6 B3LYP-D3/6-31G(d,p)

C	-8.66480	-2.48534	2.07449
N	-8.06618	-3.73031	2.06537
C	-6.99913	-4.03775	2.90796
N	-6.65231	-3.00815	3.76113
C	-7.20648	-1.72626	3.84498
C	-8.29778	-1.48788	2.91486
C	-8.50543	-4.74265	1.12257
O	-8.23627	-4.30355	-0.21097
C	-8.96053	-5.19094	-1.07810
C	-10.11725	-5.79637	-0.24078
C	-9.99919	-5.05757	1.09806
C	-9.37955	-4.47675	-2.34839
O	-10.51899	-3.61694	-2.19061
P	-10.46856	-2.06893	-1.69622
O	-10.65289	-1.95837	-0.18882
O	-9.84899	-7.19373	-0.12380
O	-6.42633	-5.11958	2.92434
O	-6.76002	-0.91108	4.65100
C	-8.95607	-0.13957	2.93043
O	-8.94235	-1.70675	-2.15889
C	-8.44562	-0.36678	-1.97817
C	-7.07274	-0.29690	-2.68909
O	-6.07733	-0.32762	-1.65226
C	-6.72723	-0.78985	-0.45931
C	-8.08889	-0.10740	-0.51782
C	-6.87518	0.92105	-3.57464
O	-7.44206	2.12512	-3.03400
P	-6.68719	3.22370	-2.10521
O	-6.92993	2.96239	-0.62409
N	-5.88446	-0.50774	0.68686
C	-5.13596	-1.56203	1.20840
N	-4.36377	-1.21280	2.29743
C	-4.23240	0.04093	2.90258
C	-5.02053	1.08771	2.27277
C	-5.78599	0.77093	1.20181
O	-5.16908	-2.70576	0.77342
O	-3.47965	0.18791	3.86317
C	-4.93875	2.47332	2.84033
O	-5.13917	2.91271	-2.52907
C	-4.07998	3.67419	-1.91256
C	-2.77447	3.32623	-2.66755
O	-2.02015	2.46377	-1.79864
C	-2.93356	1.96951	-0.80706
C	-3.80061	3.18284	-0.49611
C	-1.94126	4.52759	-3.07806
O	-1.90232	5.56799	-2.08818
P	-0.80770	5.73038	-0.89866
O	-1.30370	5.11325	0.40270
N	-2.18663	1.38017	0.28750
C	-2.08453	-0.01046	0.32066
N	-1.34241	-0.49050	1.38020
C	-0.68685	0.23477	2.37983
C	-0.82127	1.67641	2.25066
C	-1.54096	2.17363	1.21806
O	-2.62138	-0.75516	-0.48922
O	-0.04617	-0.35062	3.25005
C	-0.13925	2.54219	3.26730
O	0.42081	4.86779	-1.54808
C	1.64786	4.74510	-0.79947
C	2.69885	4.11706	-1.74674
O	2.88228	2.76011	-1.30975
C	1.74758	2.43128	-0.49456
C	1.51507	3.70524	0.30849
C	4.03042	4.84611	-1.79125
O	4.46945	5.33356	-0.51296
P	5.38047	4.52701	0.56481
O	4.52147	3.79463	1.58776
N	2.01948	1.21503	0.24693
C	1.44087	0.03343	-0.21479
N	1.75690	-1.07515	0.54322
C	2.59087	-1.15238	1.66318
C	3.18347	0.11807	2.04910
C	2.88533	1.22179	1.32474
O	0.69372	-0.03000	-1.18277

O	2.78767	-2.23259	2.21513
C	4.10871	0.13578	3.22897
O	6.13028	3.48779	-0.44966
C	7.09300	2.56090	0.09344
C	7.80649	1.89333	-1.10770
O	7.30212	0.54895	-1.18488
C	6.07624	0.52853	-0.43699
C	6.40071	1.38705	0.77929
C	9.32380	1.89533	-1.02753
O	9.84125	1.71167	0.30088
P	10.20760	0.29223	1.00035
O	9.00475	-0.33053	1.69648
N	5.67470	-0.84297	-0.19884
C	4.63185	-1.35538	-0.97023
N	4.31292	-2.66492	-0.67968
C	4.96053	-3.53760	0.19789
C	6.07763	-2.94014	0.91383
C	6.38015	-1.64107	0.68067
O	4.01489	-0.70378	-1.80323
O	4.58590	-4.70380	0.30171
C	6.84841	-3.80726	1.86445
O	10.63171	-0.55022	-0.33743
C	10.94911	-1.95270	-0.21695
C	11.47947	-2.40563	-1.58458
O	10.31801	-2.80365	-2.33915
C	9.15943	-2.79273	-1.50134
C	9.68025	-2.77573	-0.06559
C	12.48551	-3.54414	-1.52611
O	11.96721	-4.60307	-0.72284
N	8.32775	-3.94944	-1.81740
C	7.07212	-3.73133	-2.37181
N	6.32587	-4.88184	-2.52496
C	6.69184	-6.19728	-2.22009
C	8.06142	-6.34235	-1.74296
C	8.80577	-5.22506	-1.57785
O	6.64537	-2.62647	-2.68751
O	5.89257	-7.11529	-2.38540
C	8.57586	-7.72438	-1.47104
O	11.39412	0.50413	1.91639
O	6.36604	5.49416	1.18852
O	-0.43724	7.19360	-0.76784
O	-7.11170	4.60882	-2.54839
O	-11.47909	-1.26480	-2.48888
H	13.42640	-3.15790	-1.10927
H	12.68006	-3.88253	-2.55176
H	11.95132	-1.55859	-2.09363
H	11.67384	-2.10074	0.58778
H	9.94298	-3.77854	0.27003
H	8.54578	-1.91743	-1.71598
H	9.84300	-5.26084	-1.26052
H	5.39086	-4.74591	-2.88921
H	8.98391	-2.32185	0.62986
H	12.57198	-5.35335	-0.78876
H	9.68285	2.87904	-1.34082
H	9.73155	1.13888	-1.70084
H	7.52106	2.43657	-2.01819
H	7.78693	3.09624	0.74360
H	7.12104	0.88053	1.42372
H	5.26901	0.97847	-1.01860
H	7.22141	-1.15219	1.16190
H	3.54957	-3.05022	-1.22176
H	5.53030	1.70816	1.34082
H	3.91590	5.73571	-2.41613
H	4.79130	4.19885	-2.23103
H	2.28453	4.11633	-2.76369
H	1.95147	5.72874	-0.43667
H	2.31831	3.85492	1.03166
H	0.88338	2.20799	-1.12325
H	3.33983	2.18403	1.53775
H	1.34036	-1.94277	0.22880
H	0.54462	3.75569	0.78971
H	-2.40131	4.98214	-3.95947
H	-0.92940	4.20659	-3.33117
H	-3.04137	2.77713	-3.58009
H	-4.31935	4.73798	-1.96350
H	-3.22340	3.94332	0.03214
H	-3.53864	1.16044	-1.22075
H	-1.62531	3.24158	1.04502

H	-1.26257	-1.49879	1.42663
H	-4.71757	2.94965	0.03413
H	-7.39816	0.75196	-4.51965
H	-5.81165	1.06013	-3.77647
H	-6.96618	-1.18973	-3.31893
H	-9.16403	0.34775	-2.38422
H	-7.98967	0.96840	-0.36555
H	-6.84058	-1.87557	-0.48518
H	-6.35097	1.52513	0.66335
H	-3.82438	-1.96886	2.70046
H	-8.82570	-0.53109	0.15626
H	-9.68447	-5.23208	-3.07748
H	-8.53673	-3.91389	-2.75389
H	-8.30309	-6.02163	-1.37207
H	-11.08743	-5.62392	-0.71705
H	-10.57436	-4.13013	1.05683
H	-7.92429	-5.63797	1.35339
H	-9.44589	-2.35113	1.33001
H	-5.88451	-3.21091	4.38983
H	-10.32603	-5.66412	1.94519
H	-10.59036	-7.59206	0.35271
H	9.61111	-7.69459	-1.12456
H	7.96789	-8.22180	-0.70829
H	8.52752	-8.34586	-2.37128
H	7.62494	-3.23448	2.37559
H	6.18386	-4.24282	2.61722
H	7.32349	-4.64092	1.33572
H	4.47339	1.14646	3.42394
H	3.59688	-0.22877	4.12544
H	4.96950	-0.52050	3.06197
H	-0.31056	3.59999	3.05691
H	-0.51202	2.32214	4.27298
H	0.94012	2.35708	3.27758
H	-5.57137	3.16298	2.27754
H	-5.26093	2.48065	3.88675
H	-3.90927	2.84590	2.81950
H	-9.78675	-0.10252	2.22287
H	-9.33950	0.09262	3.92910
H	-8.24132	0.64926	2.67238

****

ss-A2C2A2 B3LYP-D3/6-31G(d,p)

O	11.96435	-4.60221	-0.75714
C	12.28320	-3.45272	-1.53313
C	11.17193	-2.42824	-1.38625
O	9.97652	-2.90944	-2.03389
C	10.77734	-2.13825	0.07145
O	10.42375	-0.74380	0.14483
C	9.55117	-3.02225	0.26866
C	8.89483	-2.89986	-1.10102
N	7.97702	-3.97271	-1.42953
C	7.68366	-5.11871	-0.71322
N	6.74812	-5.85988	-1.26505
C	6.40196	-5.16932	-2.41828
C	5.43734	-5.40104	-3.41797
N	4.57794	-6.45282	-3.36700
N	5.32293	-4.51268	-4.42368
C	6.10218	-3.41907	-4.40143
N	7.02981	-3.07043	-3.50201
C	7.14348	-3.98897	-2.53302
P	10.18722	-0.06991	1.61539
O	11.44602	-0.15116	2.45540
O	8.95558	-0.63774	2.30777
O	9.93266	1.47176	1.16881
C	9.66286	1.95348	-0.15671
C	8.17847	2.07764	-0.45431
O	7.62135	0.78805	-0.77191
C	7.33886	2.63635	0.71610
O	6.40629	3.57807	0.14569
C	6.61767	1.39893	1.23623
C	6.37213	0.66936	-0.07681
N	6.00316	-0.71736	0.02871
C	6.36117	-1.64159	0.99820
N	5.77451	-2.80966	0.83725
C	4.98481	-2.65293	-0.29726
C	4.08279	-3.49831	-0.96970

N	3.78197	-4.75556	-0.52410
N	3.44892	-3.03250	-2.06109
C	3.68158	-1.76960	-2.44914
N	4.49201	-0.86408	-1.88963
C	5.11753	-1.36065	-0.81618
P	5.47162	4.48355	1.13545
O	6.30359	5.47064	1.92849
O	4.56469	3.61981	2.00207
O	4.63271	5.30661	0.01376
C	4.33703	4.87038	-1.32299
C	3.02728	4.11112	-1.44478
O	3.20935	2.73244	-1.08365
C	1.88268	4.65156	-0.55210
O	0.72273	4.80770	-1.39725
C	1.68128	3.53310	0.46569
C	2.00973	2.32465	-0.40258
N	2.23461	1.05782	0.25959
C	1.69262	-0.12306	-0.34112
O	1.07221	-0.03015	-1.40161
N	1.89001	-1.30580	0.30462
C	2.60967	-1.35952	1.41377
N	2.72128	-2.57290	2.02560
C	3.22461	-0.20607	1.99872
C	3.00990	0.98394	1.37914
P	-0.55545	5.64222	-0.81210
O	-0.20489	7.10154	-0.60053
O	-1.14429	4.97645	0.42458
O	-1.55503	5.53135	-2.08825
C	-1.54059	4.52100	-3.11020
C	-2.42959	3.32659	-2.80551
O	-1.74819	2.40618	-1.93972
C	-3.76726	3.67661	-2.10921
O	-4.81346	2.98697	-2.82672
C	-3.58423	3.10629	-0.70741
C	-2.72586	1.88761	-1.01968
N	-2.04811	1.24049	0.08427
C	-1.91146	-0.18421	0.05068
O	-2.38779	-0.81246	-0.89528
N	-1.26074	-0.78254	1.08852
C	-0.72202	-0.05670	2.05574
N	-0.10754	-0.71866	3.07324
C	-0.79588	1.37189	2.08829
C	-1.46382	1.97554	1.07167
P	-6.36906	3.38242	-2.51524
O	-6.72526	4.72836	-3.11431
O	-6.68820	3.27511	-1.03099
O	-7.11935	2.22273	-3.37424
C	-6.56121	0.96633	-3.78977
C	-6.84893	-0.17622	-2.83082
O	-5.93341	-0.14140	-1.72102
C	-8.26713	-0.16719	-2.21855
O	-8.72706	-1.53503	-2.23100
C	-8.01288	0.30778	-0.79240
C	-6.68872	-0.39527	-0.52629
N	-5.93635	0.04038	0.61980
C	-5.85650	1.30141	1.18238
N	-5.05923	1.35209	2.23050
C	-4.58492	0.05225	2.37048
C	-3.72328	-0.56269	3.29735
N	-3.14119	0.12394	4.32546
N	-3.50340	-1.88617	3.19832
C	-4.10428	-2.56230	2.20782
N	-4.92246	-2.09561	1.25724
C	-5.12697	-0.77976	1.38534
P	-10.28233	-1.86994	-1.85454
O	-11.22774	-1.28724	-2.88529
O	-10.61702	-1.46633	-0.42470
O	-10.24924	-3.48495	-2.02650
C	-9.09101	-4.33061	-1.93965
C	-8.85188	-4.90464	-0.55448
O	-8.19174	-3.93872	0.28518
C	-10.12562	-5.35557	0.19967
O	-9.86776	-6.67346	0.68721
C	-10.22788	-4.32938	1.33180
C	-8.75070	-4.05592	1.59735
N	-8.43991	-2.86554	2.34696
C	-8.84632	-1.57097	2.06822
N	-8.29775	-0.67547	2.86220

C	-7.48789	-1.41497	3.71537
C	-6.60281	-1.05685	4.75063
N	-6.37435	0.23891	5.11131
N	-5.96338	-2.03649	5.42105
C	-6.15268	-3.30730	5.03451
N	-6.91189	-3.77150	4.03484
C	-7.56088	-2.78010	3.40998
H	13.22378	-2.99049	-1.20024
H	12.38715	-3.70253	-2.59799
H	11.48972	-1.49605	-1.86658
H	11.58352	-2.35105	0.77678
H	9.89916	-4.04399	0.42767
H	8.34075	-1.95977	-1.19615
H	8.17821	-5.34450	0.21851
H	5.95608	-2.72560	-5.22646
H	4.82573	-7.23801	-2.78306
H	4.05256	-6.65679	-4.20500
H	8.92188	-2.69411	1.09194
H	10.11342	2.94856	-0.20794
H	10.13570	1.30958	-0.89985
H	8.06327	2.74407	-1.31847
H	7.95111	3.13685	1.46898
H	7.30256	0.81852	1.85791
H	5.57511	1.15202	-0.65267
H	7.09159	-1.38789	1.75493
H	3.13721	-1.44148	-3.33040
H	4.51349	-5.20918	0.00926
H	3.36779	-5.35350	-1.23031
H	5.71037	1.64917	1.77828
H	4.26260	5.78630	-1.91508
H	5.15532	4.26184	-1.71249
H	2.70144	4.17194	-2.49182
H	2.13107	5.61246	-0.09798
H	2.42059	3.64534	1.26103
H	1.21310	2.13804	-1.12457
H	3.45450	1.91527	1.71707
H	3.84323	-0.27243	2.88374
H	2.50839	-3.37019	1.43750
H	3.51333	-2.70870	2.63815
H	0.67441	3.52946	0.87011
H	-1.91797	5.01480	-4.00953
H	-0.51918	4.18190	-3.29283
H	-2.65839	2.82970	-3.75788
H	-3.96659	4.75014	-2.11739
H	-3.02461	3.82854	-0.11058
H	-3.31749	1.10638	-1.49962
H	-1.53028	3.05391	0.97421
H	-0.32514	1.95237	2.87061
H	0.12400	-1.68751	2.90153
H	0.54613	-0.20968	3.64955
H	-4.53396	2.87187	-0.23998
H	-7.03123	0.73990	-4.75084
H	-5.48353	1.05930	-3.93403
H	-6.71260	-1.11524	-3.38222
H	-8.96501	0.46045	-2.77632
H	-7.85997	1.38915	-0.79552
H	-6.83486	-1.47287	-0.39820
H	-6.38135	2.13497	0.73723
H	-3.89860	-3.62949	2.17665
H	-3.05439	1.12203	4.18976
H	-2.31647	-0.31958	4.70986
H	-8.81293	0.02715	-0.11504
H	-9.28417	-5.16032	-2.62522
H	-8.20305	-3.79326	-2.27745
H	-8.20036	-5.78120	-0.66941
H	-11.00497	-5.34311	-0.45177
H	-10.70365	-3.41664	0.96306
H	-8.28738	-4.88690	2.14055
H	-9.54194	-1.38316	1.25792
H	-5.60559	-4.05568	5.60358
H	-6.55272	0.92653	4.38961
H	-5.49559	0.37909	5.59571
H	-10.76219	-4.70877	2.20539
H	-10.65956	-6.96827	1.15809
H	12.68943	-5.23339	-0.84933

****

ds-A4 B3LYP-D3/6-31G(d,p)

O	4.05982	-6.83406	-5.46682
C	5.17242	-6.30504	-6.18658
C	5.77189	-5.13262	-5.42594
O	4.89573	-3.99181	-5.47188
C	6.02217	-5.40156	-3.93566
O	7.16046	-4.59784	-3.56470
C	4.75034	-4.88028	-3.28446
C	4.41129	-3.67495	-4.15882
N	2.98678	-3.35685	-4.25527
C	2.59030	-2.02874	-4.08058
O	3.35731	-1.13540	-3.73721
N	1.25680	-1.78983	-4.32673
C	0.32823	-2.68501	-4.82684
O	-0.81336	-2.31005	-5.13523
C	0.79950	-4.05171	-4.98182
C	-0.16320	-5.09166	-5.47159
C	2.10180	-4.31049	-4.71769
P	7.88892	-4.76203	-2.10996
O	8.94439	-5.84904	-2.14347
O	6.87942	-4.93085	-0.98604
O	8.64678	-3.32257	-2.03071
C	8.29846	-2.14750	-2.77639
C	7.41883	-1.18152	-2.00782
O	6.06904	-1.66321	-1.94658
C	7.84650	-0.91600	-0.54534
O	7.77034	0.50979	-0.37442
C	6.76209	-1.62206	0.26350
C	5.56175	-1.35134	-0.63568
N	4.35037	-2.11445	-0.41643
C	3.12890	-1.46493	-0.64485
O	3.02785	-0.25105	-0.77178
N	2.03942	-2.30147	-0.69729
C	2.03948	-3.67701	-0.57566
O	0.99565	-4.33013	-0.76810
C	3.30818	-4.27322	-0.21717
C	3.38678	-5.75613	-0.01090
C	4.40064	-3.47118	-0.19398
P	8.29071	1.25890	0.97702
O	9.79761	1.16972	1.10922
O	7.54508	0.78963	2.21894
O	7.87373	2.78103	0.58131
C	6.94440	3.15593	-0.44890
C	5.53930	3.39071	0.06954
O	4.86630	2.14767	0.33037
C	5.47376	4.20673	1.38066
O	4.47094	5.21767	1.17661
C	5.01271	3.17346	2.40105
C	4.08704	2.33690	1.52445
N	3.66145	1.04848	2.03820
C	2.30396	0.72434	1.95495
O	1.44428	1.51354	1.57570
N	1.99223	-0.55238	2.36396
C	2.87403	-1.52408	2.80212
O	2.47022	-2.67073	3.06409
C	4.25768	-1.10824	2.91353
C	5.25990	-2.09216	3.44004
C	4.58706	0.14219	2.50548
P	4.26371	6.42650	2.25561
O	5.46758	7.34810	2.28092
O	3.87747	5.90160	3.63244
O	3.03607	7.19692	1.52429
C	2.06388	6.58061	0.65606
C	0.86343	6.01122	1.38126
O	1.18829	4.74857	1.98855
C	0.28040	6.90831	2.50302
O	-1.09848	7.12398	2.18669
C	0.45911	6.04900	3.76105
C	0.37260	4.65036	3.15420
N	0.83708	3.54106	3.96728
C	-0.01828	2.45720	4.15359
O	-1.19487	2.45540	3.79362
N	0.54909	1.38540	4.79894
C	1.85815	1.27312	5.23516
O	2.26787	0.21060	5.73121
C	2.67578	2.45918	5.07184
C	4.07869	2.43083	5.60337
C	2.13490	3.52462	4.43017

O	-4.42759	-7.50666	4.25414
C	-5.64956	-7.37077	4.97849
C	-6.28835	-6.03256	4.64310
O	-5.56170	-4.95266	5.26089
C	-6.32248	-5.73357	3.13766
O	-7.56840	-5.06876	2.84191
C	-5.13315	-4.80809	2.92821
C	-4.93567	-4.12826	4.28414
N	-3.53068	-3.94690	4.62028
C	-2.56214	-4.93325	4.70923
N	-1.36456	-4.46090	4.98103
C	-1.54798	-3.08958	5.07336
C	-0.65783	-2.02244	5.31766
N	0.66271	-2.18756	5.52694
N	-1.18046	-0.77373	5.37154
C	-2.49450	-0.59325	5.15201
N	-3.41959	-1.51590	4.88969
C	-2.89005	-2.75099	4.86250
P	-8.02561	-4.96506	1.26980
O	-9.03948	-6.03246	0.91385
O	-6.80518	-4.93895	0.36425
O	-8.82632	-3.54762	1.29992
C	-8.52581	-2.42598	2.14288
C	-7.59763	-1.40436	1.50750
O	-6.22457	-1.78619	1.69192
C	-7.78958	-1.20075	-0.01309
O	-7.79460	0.22275	-0.23117
C	-6.53925	-1.84382	-0.61079
C	-5.52476	-1.49426	0.46821
N	-4.27938	-2.21389	0.47897
C	-4.01977	-3.53724	0.15352
N	-2.81148	-3.92519	0.50028
C	-2.23812	-2.80595	1.09503
C	-0.98330	-2.56702	1.69984
N	-0.04092	-3.51324	1.86467
N	-0.74697	-1.32199	2.17881
C	-1.69684	-0.37553	2.08135
N	-2.92061	-0.49937	1.56536
C	-3.13476	-1.73450	1.08901
P	-8.26864	0.83090	-1.67037
O	-9.73969	0.56051	-1.91865
O	-7.36700	0.37214	-2.80678
O	-8.06294	2.41410	-1.35734
C	-7.29603	2.97468	-0.27921
C	-5.87154	3.31702	-0.67532
O	-5.05823	2.13140	-0.72357
C	-5.74253	3.98818	-2.06090
O	-4.80471	5.07400	-1.92538
C	-5.13954	2.88622	-2.92460
C	-4.23304	2.22019	-1.89692
N	-3.72582	0.91309	-2.21770
C	-4.37252	-0.13996	-2.84717
N	-3.65701	-1.24526	-2.86328
C	-2.47881	-0.91085	-2.20816
C	-1.33859	-1.65925	-1.84483
N	-1.17606	-2.96274	-2.12491
N	-0.36121	-1.00897	-1.17121
C	-0.50489	0.29004	-0.85418
N	-1.54771	1.07801	-1.11782
C	-2.50495	0.42330	-1.79211
P	-4.99472	6.39747	-2.86973
O	-6.17342	7.21962	-2.38867
O	-4.99657	6.04228	-4.34248
O	-3.56322	7.11558	-2.56073
C	-3.16180	7.39232	-1.20881
C	-1.65461	7.27429	-1.09044
O	-1.25249	5.90325	-1.30591
C	-0.84757	8.11723	-2.08630
O	0.32742	8.55383	-1.40083
C	-0.53835	7.12649	-3.20766
C	-0.44438	5.78220	-2.47630
N	-0.89697	4.65520	-3.27455
C	-2.06508	4.56898	-4.01602
N	-2.23989	3.38945	-4.57035
C	-1.13729	2.65181	-4.17221
C	-0.75747	1.31794	-4.42380
N	-1.48887	0.47914	-5.18147
N	0.40944	0.89135	-3.88838

C	1.13396	1.72973	-3.12304
N	0.86231	2.99912	-2.80854
C	-0.29021	3.41266	-3.36185
H	5.95144	-7.06928	-6.31933
H	4.87031	-5.94958	-7.17991
H	6.71442	-4.86154	-5.91315
H	6.22173	-6.45561	-3.72579
H	3.97609	-5.64599	-3.34086
H	4.88997	-2.77060	-3.78528
H	2.54060	-5.28726	-4.89179
H	0.95202	-0.80180	-4.16761
H	4.91875	-4.60891	-2.25072
H	9.24297	-1.64498	-3.00461
H	7.80955	-2.42372	-3.71181
H	7.44095	-0.22375	-2.54455
H	8.85801	-1.26430	-0.32534
H	6.98776	-2.69020	0.29716
H	5.26726	-0.30198	-0.57724
H	5.39311	-3.88710	-0.07589
H	1.11922	-1.81389	-0.87383
H	6.67059	-1.21007	1.26317
H	7.31854	4.09877	-0.85801
H	6.93137	2.40652	-1.24072
H	4.99037	3.94367	-0.70415
H	6.42703	4.67669	1.63158
H	5.86919	2.57844	2.72646
H	3.16759	2.88234	1.30615
H	5.61842	0.48513	2.49817
H	0.97724	-0.80317	2.32790
H	4.52222	3.64082	3.24633
H	1.71107	7.37199	-0.00802
H	2.53993	5.79897	0.06097
H	0.07804	5.84752	0.63200
H	0.81396	7.86053	2.57379
H	1.45642	6.21046	4.17746
H	-0.65940	4.41265	2.88653
H	2.70624	4.42501	4.21689
H	-0.09625	0.58679	5.00094
H	-0.30293	6.24308	4.51883
H	-1.45116	7.75742	2.82668
H	-6.35468	-8.16925	4.70826
H	-5.47938	-7.41564	6.06178
H	-7.30595	-6.02823	5.04671
H	-6.24365	-6.64833	2.54704
H	-4.25047	-5.38750	2.65878
H	-5.37182	-3.12568	4.30752
H	-2.83389	-5.97293	4.58333
H	-2.82522	0.44170	5.17168
H	1.09134	-3.02083	5.15115
H	1.24920	-1.34970	5.56673
H	-5.33525	-4.08380	2.14793
H	-9.48808	-1.94478	2.33779
H	-8.10359	-2.76664	3.09091
H	-7.77156	-0.44259	2.00658
H	-8.72165	-1.63418	-0.38047
H	-6.67727	-2.92668	-0.64907
H	-5.26467	-0.43097	0.43246
H	-4.78956	-4.16213	-0.27391
H	-1.42879	0.60002	2.47496
H	-0.03971	-4.29133	1.22037
H	0.85706	-3.22914	2.26197
H	-6.30172	-1.44072	-1.59095
H	-7.80831	3.90000	-0.00044
H	-7.29251	2.29912	0.57705
H	-5.46964	4.00264	0.08160
H	-6.70372	4.35498	-2.42628
H	-5.92509	2.18848	-3.22465
H	-3.35481	2.84017	-1.68823
H	-5.38500	-0.02485	-3.20944
H	0.32420	0.73132	-0.31006
H	-1.96240	-3.46360	-2.50541
H	-0.44263	-3.48969	-1.64611
H	-4.60118	3.27317	-3.78676
H	-3.47774	8.40494	-0.93487
H	-3.62361	6.67861	-0.52232
H	-1.38202	7.54911	-0.06660
H	-1.42175	8.97777	-2.44851
H	-1.36652	7.11669	-3.91701

H	0.58050	5.53993	-2.18272
H	-2.75624	5.39278	-4.10292
H	2.03988	1.29908	-2.70524
H	-2.42783	0.76703	-5.41167
H	-1.28093	-0.52418	-5.15573
H	0.38020	7.36399	-3.74830
H	0.89458	9.00703	-2.03996
H	0.33186	-6.05811	-5.59069
H	-0.99452	-5.21306	-4.76835
H	-0.60005	-4.80158	-6.43250
H	4.42628	-6.07335	0.09668
H	2.83082	-6.06467	0.88130
H	2.94646	-6.28538	-0.86195
H	6.25236	-1.64076	3.50060
H	4.97378	-2.43870	4.43843
H	5.31778	-2.97911	2.79966
H	4.55654	3.40739	5.50339
H	4.08282	2.14818	6.66073
H	4.68686	1.68967	5.07350
H	3.62485	-7.48973	-6.02711
H	-4.01825	-8.34317	4.51097

****

ds-G4 B3LYP-D3/6-31G(d,p)

O	4.73390	-6.29000	-5.71095
C	5.98091	-5.95139	-6.31513
C	6.63600	-4.82189	-5.53554
O	5.93957	-3.58036	-5.75200
C	6.65059	-5.04432	-4.01687
O	7.86429	-4.45425	-3.50719
C	5.42442	-4.28076	-3.54408
C	5.30256	-3.13884	-4.55173
N	3.92618	-2.77292	-4.83371
C	2.90753	-3.61706	-5.25850
N	1.75931	-2.99837	-5.40445
C	2.02587	-1.67874	-5.05962
C	1.17738	-0.53414	-5.01949
O	-0.03354	-0.47395	-5.30586
N	1.86829	0.61136	-4.60436
C	3.20005	0.65864	-4.27266
N	3.68997	1.87023	-3.91218
N	4.00640	-0.39324	-4.33133
C	3.37059	-1.51897	-4.71190
P	8.30503	-4.74870	-1.95627
O	9.29741	-5.89032	-1.87243
O	7.08464	-4.92338	-1.06809
O	9.12276	-3.37685	-1.63949
C	8.86492	-2.09178	-2.22517
C	7.91390	-1.22370	-1.42031
O	6.54735	-1.56811	-1.69682
C	8.08592	-1.31873	0.11451
O	8.10412	0.03667	0.59475
C	6.82128	-2.04883	0.55617
C	5.82423	-1.49054	-0.45117
N	4.57509	-2.18617	-0.58489
C	4.33255	-3.55469	-0.50488
N	3.09256	-3.86827	-0.79382
C	2.47445	-2.65382	-1.07571
C	1.13561	-2.35138	-1.45960
O	0.20725	-3.15180	-1.68687
N	0.92812	-0.97359	-1.57935
C	1.88664	-0.00389	-1.39497
N	1.47899	1.26279	-1.55507
N	3.15522	-0.27964	-1.10536
C	3.37920	-1.59653	-0.94802
P	8.47336	0.39439	2.14228
O	9.93727	0.11877	2.42871
O	7.52797	-0.26858	3.13211
O	8.22618	2.00336	2.08500
C	7.53777	2.69732	1.03325
C	6.06216	2.89936	1.31508
O	5.33481	1.66532	1.19835
C	5.74753	3.45588	2.72184
O	4.76660	4.49147	2.51749
C	5.13878	2.25210	3.43269
C	4.37299	1.63334	2.27050
N	3.86777	0.30213	2.45101

C	4.51824	-0.82882	2.93415
N	3.74611	-1.89186	2.93483
C	2.52012	-1.44823	2.44360
C	1.30053	-2.13927	2.17462
O	1.05793	-3.35628	2.29504
N	0.30744	-1.27259	1.70093
C	0.47215	0.07052	1.44737
N	-0.58602	0.72027	0.94935
N	1.61619	0.71201	1.65806
C	2.58144	-0.08621	2.13948
P	4.33005	5.49468	3.72983
O	5.48278	6.38857	4.14304
O	3.70538	4.73270	4.88988
O	3.24684	6.39862	2.92463
C	2.45761	5.97972	1.79373
C	1.09698	5.42755	2.17132
O	1.20786	4.05799	2.59814
C	0.38323	6.19278	3.31163
O	-0.94626	6.47036	2.85668
C	0.39689	5.17919	4.45987
C	0.28668	3.87496	3.67604
N	0.60818	2.65630	4.37042
C	1.82096	2.29605	4.94659
N	1.85025	1.03595	5.31621
C	0.60050	0.53060	4.96661
C	0.05720	-0.78640	5.04467
O	0.60735	-1.82257	5.46376
N	-1.25658	-0.83697	4.54879
C	-1.95052	0.22600	4.01806
N	-3.22484	-0.00439	3.62507
N	-1.44202	1.44758	3.91893
C	-0.18048	1.52773	4.37728
O	-5.98392	-8.25641	3.79956
C	-7.17843	-7.66922	4.31264
C	-7.39101	-6.29599	3.69404
O	-6.38783	-5.37251	4.15566
C	-7.30366	-6.25420	2.16236
O	-8.13880	-5.15048	1.75119
C	-5.83375	-5.93595	1.92927
C	-5.56894	-4.94159	3.05771
N	-4.18523	-4.86076	3.51866
C	-3.59227	-3.58689	3.66879
O	-4.23451	-2.58339	3.32037
N	-2.34466	-3.50883	4.19156
C	-1.68638	-4.61259	4.57347
N	-0.46608	-4.45600	5.09230
C	-2.28305	-5.91423	4.46411
C	-3.53832	-5.98242	3.95630
P	-8.45025	-4.84073	0.17673
O	-9.52385	-5.75893	-0.36892
O	-7.17765	-4.83051	-0.65810
O	-9.08289	-3.35036	0.32834
C	-8.84083	-2.42214	1.39759
C	-7.80117	-1.36745	1.06117
O	-6.47581	-1.89206	1.22933
C	-7.87035	-0.82455	-0.38371
O	-7.73414	0.61017	-0.28764
C	-6.63760	-1.44312	-1.03781
C	-5.67313	-1.39337	0.14337
N	-4.44932	-2.17002	0.08084
C	-3.24227	-1.57559	0.52408
O	-3.19324	-0.34018	0.65817
N	-2.18198	-2.37734	0.78118
C	-2.24937	-3.70024	0.57854
N	-1.22141	-4.45400	0.97595
C	-3.40830	-4.29664	-0.01593
C	-4.49393	-3.50467	-0.20064
P	-8.11738	1.54808	-1.56846
O	-9.60563	1.51154	-1.84986
O	-7.26157	1.21573	-2.78359
O	-7.73229	3.00542	-0.96015
C	-6.88347	3.28595	0.16504
C	-5.47530	3.69686	-0.23357
O	-4.67665	2.53783	-0.52420
C	-5.39924	4.59518	-1.48780
O	-4.40994	5.60903	-1.21730
C	-4.88594	3.63713	-2.55778
C	-3.91522	2.81972	-1.71227

N	-3.42481	1.56446	-2.24726
C	-2.08041	1.19752	-1.99556
O	-1.29103	2.05708	-1.56267
N	-1.70534	-0.08071	-2.23927
C	-2.57508	-0.97817	-2.72366
N	-2.17717	-2.24947	-2.83683
C	-3.90313	-0.59633	-3.09325
C	-4.29616	0.66503	-2.79186
P	-4.35468	6.94648	-2.16421
O	-5.42688	7.92911	-1.73967
O	-4.33204	6.59175	-3.63714
O	-2.85062	7.45567	-1.78269
C	-2.40004	7.51345	-0.41926
C	-0.90894	7.24895	-0.35316
O	-0.63002	5.91148	-0.82593
C	-0.00769	8.18704	-1.16650
O	1.17667	8.37659	-0.38797
C	0.26351	7.38872	-2.43882
C	0.28043	5.94544	-1.92768
N	-0.13988	4.95372	-2.92017
C	0.72988	3.90692	-3.29018
O	1.89545	3.89962	-2.84638
N	0.25971	2.94511	-4.12224
C	-0.99866	2.98485	-4.58292
N	-1.44219	1.94630	-5.30060
C	-1.84871	4.10615	-4.32542
C	-1.38996	5.04240	-3.46311
H	6.66267	-6.81378	-6.31626
H	5.84500	-5.62184	-7.35330
H	7.66056	-4.70434	-5.90353
H	6.61716	-6.10569	-3.75909
H	4.54882	-4.92836	-3.58775
H	5.78304	-2.22778	-4.18750
H	3.11253	-4.65928	-5.46092
H	1.29651	1.46848	-4.45206
H	4.58987	1.83279	-3.45601
H	3.04097	2.58931	-3.57552
H	5.55387	-3.91478	-2.53350
H	9.83627	-1.59085	-2.26361
H	8.48839	-2.20779	-3.24364
H	8.08960	-0.18152	-1.71788
H	9.00835	-1.82719	0.40311
H	6.95269	-3.11976	0.38495
H	5.57780	-0.44874	-0.22454
H	5.13498	-4.24441	-0.29009
H	-0.03657	-0.65810	-1.81278
H	2.14356	2.00583	-1.41730
H	0.48888	1.50711	-1.63024
H	6.56601	-1.84316	1.59136
H	8.00882	3.68229	0.96352
H	7.66766	2.17550	0.08495
H	5.68497	3.61674	0.57408
H	6.62552	3.87176	3.22123
H	5.93328	1.57016	3.74729
H	3.50243	2.24345	2.01233
H	5.55884	-0.77561	3.22671
H	-0.60113	-1.69796	1.43079
H	-0.45503	1.66285	0.62263
H	-1.50273	0.28168	0.82487
H	4.51336	2.53982	4.27287
H	2.30389	6.87566	1.18702
H	3.00651	5.24109	1.20659
H	0.46663	5.46200	1.27419
H	0.90680	7.11908	3.56485
H	1.36076	5.20936	4.97501
H	-0.73143	3.74004	3.29393
H	2.60961	3.03110	5.04801
H	-1.67109	-1.78638	4.46837
H	-3.54470	0.62032	2.89760
H	-3.53311	-0.97789	3.50555
H	-0.41972	5.32986	5.16952
H	-1.38392	7.00153	3.53612
H	-8.05355	-8.29423	4.08459
H	-7.12277	-7.54594	5.40182
H	-8.37269	-5.93075	4.01393
H	-7.64229	-7.17861	1.68724
H	-5.24816	-6.84711	2.05376
H	-5.83735	-3.92853	2.76217

H	-4.10490	-6.90552	3.89443
H	-1.76818	-6.80150	4.80761
H	-0.05040	-3.51905	5.16326
H	0.07512	-5.26172	5.36184
H	-5.66336	-5.51526	0.94597
H	-9.79626	-1.92066	1.57618
H	-8.54588	-2.95397	2.30375
H	-7.94829	-0.52663	1.75167
H	-8.80816	-1.07395	-0.88359
H	-6.85591	-2.47906	-1.30927
H	-5.35861	-0.36704	0.33502
H	-5.45057	-3.89498	-0.53579
H	-3.44987	-5.35676	-0.22787
H	-0.39770	-4.04007	1.43260
H	-1.24161	-5.44807	0.81475
H	-6.31468	-0.87452	-1.90311
H	-7.35419	4.12183	0.69034
H	-6.84065	2.42555	0.83468
H	-5.03296	4.23793	0.61270
H	-6.35946	5.05565	-1.72920
H	-5.71195	3.01630	-2.91210
H	-3.02853	3.40453	-1.46682
H	-5.32108	0.99833	-2.92548
H	-4.59606	-1.31036	-3.51770
H	-1.28123	-2.56132	-2.43148
H	-2.84701	-2.94873	-3.11605
H	-4.42470	4.17679	-3.37890
H	-2.61778	8.50282	-0.00217
H	-2.91226	6.75654	0.17838
H	-0.63100	7.31023	0.70291
H	-0.49420	9.14772	-1.37183
H	-0.55675	7.53607	-3.14469
H	1.27425	5.63674	-1.60615
H	-1.99073	5.88772	-3.15976
H	-2.83402	4.18706	-4.76024
H	-0.90080	1.06973	-5.33683
H	-2.40989	1.92623	-5.58228
H	1.19976	7.66417	-2.92922
H	1.80896	8.87509	-0.92386
H	4.31028	-6.96430	-6.25759
H	-5.81579	-9.07074	4.29108

****
B3LYP-D3/TZVP
****

ss-A6 B3LYP-D3/TZVP

O	12.29931	-4.13487	-0.38301
C	12.62564	-2.79038	-0.02665
C	11.35687	-1.97670	0.10286
O	10.75315	-1.78306	-1.19460
C	10.27207	-2.62281	0.97972
O	9.59768	-1.56308	1.67947
C	9.33199	-3.24495	-0.04294
C	9.37962	-2.18799	-1.13396
N	8.95000	-2.60949	-2.44411
C	8.88705	-3.87996	-2.98339
N	8.47707	-3.89317	-4.22732
C	8.26710	-2.56021	-4.54119
C	7.81168	-1.91031	-5.70255
N	7.43856	-2.58046	-6.80995
N	7.72663	-0.56804	-5.68981
C	8.04213	0.08821	-4.56687
N	8.46119	-0.41556	-3.40653
C	8.55763	-1.74738	-3.44802
P	8.86342	-1.87755	3.11790
O	9.91025	-2.04651	4.20986
O	7.84236	-3.00005	2.99685
O	8.01911	-0.48784	3.25861
C	8.63260	0.80192	3.12089
C	7.68291	1.74142	2.40276
O	7.47179	1.30603	1.04329
C	6.28419	1.85436	3.02230
O	5.84930	3.20576	2.78266
C	5.50510	0.80397	2.24663
C	6.10561	0.91865	0.85284
N	6.02620	-0.29043	0.06219
C	6.19008	-1.60169	0.46267

N	5.95966	-2.46313	-0.50106
C	5.61580	-1.68786	-1.59770
C	5.23371	-2.00319	-2.91162
N	5.15186	-3.27430	-3.36519
N	4.89517	-0.99666	-3.73550
C	4.95428	0.25981	-3.27817
N	5.32467	0.67839	-2.06898
C	5.64438	-0.33509	-1.26354
P	4.28230	3.71890	2.90274
O	4.34586	5.18857	3.23636
O	3.47150	2.72627	3.71765
O	3.73679	3.52023	1.34989
C	4.17218	4.40519	0.30994
C	3.09175	4.56004	-0.74475
O	2.91521	3.36116	-1.52519
C	1.71251	4.88689	-0.17569
O	1.04366	5.73928	-1.12713
C	1.05286	3.51914	-0.06502
C	1.68107	2.71587	-1.20452
N	1.92715	1.32444	-0.86739
C	2.61259	0.86392	0.23808
N	2.83229	-0.42552	0.21323
C	2.26749	-0.85376	-0.97693
C	2.19623	-2.11059	-1.59631
N	2.71768	-3.22954	-1.04125
N	1.56288	-2.20083	-2.77945
C	1.05397	-1.09030	-3.32671
N	1.08800	0.15560	-2.85158
C	1.70677	0.22268	-1.66939
P	-0.29826	6.57161	-0.66090
O	-0.54863	7.62909	-1.72135
O	-0.18771	7.02771	0.78330
O	-1.47193	5.41923	-0.69346
C	-1.89618	4.88952	-1.95626
C	-2.82493	3.72307	-1.71206
O	-2.07441	2.58947	-1.22363
C	-3.91361	3.99521	-0.65870
O	-5.11611	3.34285	-1.09380
C	-3.37546	3.29332	0.58187
C	-2.74007	2.07444	-0.06403
N	-1.78956	1.33994	0.73400
C	-1.05490	1.74403	1.82300
N	-0.24670	0.81173	2.27266
C	-0.45618	-0.27969	1.43722
C	0.04333	-1.59516	1.40603
N	0.91744	-2.08386	2.31589
N	-0.40998	-2.42272	0.45008
C	-1.31131	-1.97661	-0.42793
N	-1.86340	-0.76518	-0.49126
C	-1.40864	0.03793	0.47023
P	-6.57375	4.03104	-0.76837
O	-6.72501	5.32216	-1.56102
O	-6.82664	4.13631	0.72850
O	-7.52241	2.82466	-1.32157
C	-7.38028	2.27836	-2.64090
C	-7.61362	0.78083	-2.59357
O	-6.55287	0.12166	-1.87190
C	-8.91225	0.35429	-1.90037
O	-9.36362	-0.83244	-2.58156
C	-8.45515	0.10460	-0.47137
C	-7.05445	-0.46046	-0.66357
N	-6.14949	-0.19994	0.43606
C	-6.04696	0.92615	1.22990
N	-5.13186	0.81244	2.16347
C	-4.59509	-0.45211	1.98378
C	-3.60499	-1.17593	2.66662
N	-2.94723	-0.68000	3.73874
N	-3.33516	-2.42728	2.25933
C	-4.01422	-2.92944	1.22201
N	-4.96194	-2.33791	0.49489
C	-5.21760	-1.09840	0.91676
P	-10.54155	-1.81694	-1.98204
O	-11.27417	-2.39863	-3.18077
O	-11.35196	-1.13952	-0.89593
O	-9.65270	-2.99437	-1.24459
C	-8.86743	-3.87999	-2.05706
C	-8.57550	-5.16207	-1.30690
O	-7.66577	-4.93666	-0.21325

C	-9.80019	-5.84272	-0.69424
O	-9.56255	-7.25388	-0.75135
C	-9.80697	-5.31330	0.73722
C	-8.32129	-5.10521	1.04348
N	-8.04861	-3.96163	1.89466
C	-8.55698	-2.68304	1.77411
N	-8.03038	-1.83394	2.62079
C	-7.11461	-2.58226	3.34313
C	-6.22072	-2.25833	4.37647
N	-6.12578	-1.01186	4.89964
N	-5.44807	-3.23653	4.88089
C	-5.54121	-4.46621	4.36203
N	-6.32991	-4.88635	3.37229
C	-7.10283	-3.90564	2.89793
H	13.15206	-2.75828	0.93373
H	13.26308	-2.32732	-0.78606
H	11.61642	-1.00181	0.52067
H	10.68628	-3.34508	1.68142
H	9.76841	-4.17842	-0.39293
H	8.76338	-1.32671	-0.87117
H	9.14488	-4.75285	-2.40857
H	7.94202	1.16671	-4.61095
H	7.60429	-3.57007	-6.88046
H	7.22336	-2.06500	-7.64724
H	8.33475	-3.41415	0.34747
H	8.86171	1.19992	4.11226
H	9.55475	0.72604	2.54550
H	8.14678	2.72892	2.37684
H	6.28436	1.66449	4.09490
H	5.72790	-0.16804	2.67779
H	5.60218	1.68123	0.26010
H	6.50585	-1.87129	1.45860
H	4.66791	1.03018	-3.98479
H	5.61051	-4.00437	-2.84526
H	5.05967	-3.40969	-4.35921
H	4.43521	0.95981	2.26934
H	4.39466	5.38776	0.73053
H	5.07756	4.00554	-0.15288
H	3.40930	5.34858	-1.42851
H	1.77023	5.39802	0.78330
H	1.31345	3.08441	0.89528
H	1.05145	2.69477	-2.09267
H	2.94357	1.55530	0.99115
H	0.55745	-1.22344	-4.28136
H	3.43120	-3.11009	-0.33747
H	2.84740	-4.01381	-1.66191
H	-0.02541	3.56694	-0.15917
H	-2.42239	5.66104	-2.52242
H	-1.03615	4.55323	-2.53993
H	-3.30071	3.45375	-2.65703
H	-4.08218	5.06031	-0.51233
H	-2.61561	3.92317	1.04129
H	-3.50010	1.35436	-0.37158
H	-1.15493	2.72852	2.24633
H	-1.63109	-2.68900	-1.17920
H	1.48852	-1.42871	2.82417
H	1.36885	-2.95192	2.07016
H	-4.14994	3.04262	1.29817
H	-8.11513	2.74289	-3.30244
H	-6.37982	2.46815	-3.02888
H	-7.60905	0.40847	-3.61930
H	-9.68806	1.11594	-1.95995
H	-8.41692	1.06145	0.04124
H	-7.06735	-1.54451	-0.77476
H	-6.63993	1.81170	1.06580
H	-3.75672	-3.94336	0.93924
H	-2.98247	0.31068	3.91425
H	-2.12791	-1.17350	4.05479
H	-9.11211	-0.55834	0.07612
H	-9.41074	-4.12020	-2.97340
H	-7.92622	-3.39304	-2.32215
H	-8.08876	-5.84681	-2.00411
H	-10.71569	-5.58638	-1.23072
H	-10.34450	-4.36918	0.76747
H	-7.88218	-5.96068	1.55484
H	-9.29752	-2.45334	1.02743
H	-4.88743	-5.21137	4.80105
H	-6.47125	-0.24710	4.33936

H	-5.28910	-0.81712	5.42903
H	-10.27231	-6.00419	1.43805
H	-10.30073	-7.70160	-0.32079
H	13.12074	-4.62359	-0.50357

****

ss-C6 B3LYP-D3/TZVP

O	12.50861	-4.70658	-0.83341
C	12.97675	-3.58686	-1.59156
C	11.91704	-2.50349	-1.62018
O	10.77381	-2.92919	-2.38729
C	11.37232	-2.11301	-0.24267
O	11.03055	-0.71364	-0.30169
C	10.12820	-2.97622	-0.11571
C	9.60803	-2.98712	-1.54809
N	8.80851	-4.14659	-1.91093
C	7.64220	-3.95782	-2.70716
O	7.36608	-2.83120	-3.11484
N	6.87972	-5.04961	-2.97161
C	7.25996	-6.25614	-2.57986
N	6.43189	-7.28575	-2.83161
C	8.50241	-6.49031	-1.91087
C	9.23756	-5.40095	-1.60014
P	10.66656	0.08574	1.08825
O	11.90583	0.28273	1.94970
O	9.50448	-0.57865	1.82556
O	10.25072	1.53034	0.45656
C	9.68671	1.78005	-0.84123
C	8.17204	1.74340	-0.88606
O	7.70224	0.39249	-1.03225
C	7.46000	2.32323	0.35744
O	6.48111	3.26773	-0.12091
C	6.80389	1.09831	0.98428
C	6.49223	0.28547	-0.26191
N	6.15927	-1.11145	-0.09226
C	5.14181	-1.67481	-0.91917
O	4.53669	-0.95050	-1.70705
N	4.88586	-3.00012	-0.78557
C	5.58506	-3.74755	0.05125
N	5.27623	-5.05948	0.12813
C	6.62037	-3.21438	0.87581
C	6.87583	-1.89206	0.75972
P	5.71788	4.25221	0.94887
O	6.69328	5.22138	1.60265
O	4.88495	3.45111	1.94956
O	4.77690	5.08707	-0.08936
C	4.31667	4.66206	-1.38217
C	2.97799	3.95190	-1.36282
O	3.14550	2.56330	-1.02932
C	1.94752	4.52189	-0.36231
O	0.71864	4.71699	-1.09037
C	1.81771	3.41087	0.67243
C	2.02517	2.18859	-0.20824
N	2.31054	0.93080	0.44658
C	1.69497	-0.25568	-0.05101
O	0.92722	-0.18101	-1.00858
N	1.98548	-1.42407	0.57453
C	2.85400	-1.46475	1.57028
N	3.08440	-2.66120	2.15369
C	3.52378	-0.30118	2.05314
C	3.22067	0.87282	1.45542
P	-0.50006	5.57487	-0.40278
O	-0.09688	7.02721	-0.18659
O	-1.02717	4.88931	0.85826
O	-1.58307	5.50957	-1.62036
C	-1.65039	4.53281	-2.67204
C	-2.55432	3.35584	-2.36505
O	-1.86037	2.37404	-1.57526
C	-3.84294	3.70921	-1.59069
O	-4.93798	3.07820	-2.28487
C	-3.60622	3.08025	-0.22304
C	-2.80387	1.85154	-0.62365
N	-2.09283	1.14494	0.41896
C	-2.10211	-0.28121	0.41005
O	-2.71929	-0.87610	-0.47205
N	-1.42978	-0.91999	1.39992
C	-0.74026	-0.24249	2.30167

N	-0.10746	-0.94532	3.26612
C	-0.66975	1.18223	2.30172
C	-1.36171	1.83050	1.33796
P	-6.47897	3.48254	-1.88961
O	-6.74532	4.95844	-2.14839
O	-6.82736	3.03151	-0.47058
O	-7.27696	2.60960	-3.01233
C	-6.84024	1.39229	-3.63602
C	-7.19994	0.12991	-2.87797
O	-6.23203	-0.13138	-1.84590
C	-8.58853	0.13656	-2.19512
O	-9.31461	-1.00110	-2.70306
C	-8.24603	0.02671	-0.71446
C	-6.93439	-0.74361	-0.75753
N	-6.11503	-0.69059	0.43689
C	-5.62158	-1.90047	1.00137
O	-5.87153	-2.97247	0.45112
N	-4.90001	-1.81239	2.14772
C	-4.60164	-0.63559	2.67501
N	-3.87694	-0.62073	3.81124
C	-5.04554	0.59427	2.10252
C	-5.80090	0.51364	0.98437
P	-10.81246	-1.37721	-2.12850
O	-11.59916	-1.97508	-3.28262
O	-11.43245	-0.21392	-1.37580
O	-10.47108	-2.54045	-1.00945
C	-9.98453	-3.81028	-1.47494
C	-10.14966	-4.85910	-0.39780
O	-9.23569	-4.61456	0.68774
C	-11.54941	-4.93267	0.22403
O	-11.82971	-6.31859	0.45670
C	-11.39268	-4.14757	1.52227
C	-9.94287	-4.43096	1.91536
N	-9.29105	-3.36246	2.66599
C	-8.68028	-3.64181	3.91772
O	-8.76962	-4.77262	4.39723
N	-8.02013	-2.62720	4.53557
C	-7.95413	-1.42257	3.98720
N	-7.30228	-0.45776	4.66425
C	-8.55724	-1.11752	2.72845
C	-9.19851	-2.12757	2.10399
H	13.88650	-3.17017	-1.14557
H	13.19904	-3.88160	-2.62124
H	12.34835	-1.62407	-2.10219
H	12.09793	-2.28290	0.55239
H	10.42419	-3.96810	0.21394
H	8.97999	-2.12377	-1.74687
H	10.20238	-5.46746	-1.11757
H	8.83845	-7.48452	-1.65977
H	5.57882	-7.11964	-3.33979
H	6.68437	-8.23184	-2.60457
H	9.41726	-2.55870	0.58394
H	13.16254	-5.41109	-0.89780
H	10.01337	2.78644	-1.10285
H	10.08974	1.07704	-1.56896
H	7.85545	2.32495	-1.75734
H	8.15206	2.82606	1.02994
H	7.54125	0.58658	1.60005
H	5.66237	0.72231	-0.81244
H	7.67668	-1.40243	1.29938
H	7.19322	-3.83789	1.54487
H	4.66764	-5.44441	-0.57628
H	5.88417	-5.69588	0.61534
H	5.93124	1.36324	1.56780
H	4.21085	5.57836	-1.96309
H	5.06124	4.02542	-1.85768
H	2.55110	4.02632	-2.36769
H	2.27046	5.46861	0.06584
H	2.62872	3.50507	1.39294
H	1.15335	2.00581	-0.83213
H	3.70353	1.80548	1.72182
H	4.25250	-0.35207	2.84754
H	2.74138	-3.48642	1.68840
H	3.86600	-2.77461	2.77706
H	0.85795	3.43523	1.17369
H	-2.05874	5.06432	-3.53166
H	-0.65255	4.17315	-2.91776
H	-2.84292	2.90413	-3.31894

H	-4.01385	4.78260	-1.54333
H	-2.99735	3.75647	0.37492
H	-3.44132	1.11069	-1.10023
H	-1.34483	2.90908	1.23340
H	-0.08246	1.72313	3.02771
H	-0.02121	-1.94145	3.14230
H	0.57548	-0.49389	3.85104
H	-4.53566	2.85533	0.28555
H	-7.35053	1.36884	-4.59882
H	-5.76477	1.42112	-3.80745
H	-7.18244	-0.69730	-3.59430
H	-9.15000	1.03994	-2.41670
H	-8.07601	1.02956	-0.32515
H	-7.09794	-1.80042	-0.95811
H	-6.17710	1.38918	0.46571
H	-4.78304	1.54871	2.53253
H	-3.44652	-1.47910	4.11504
H	-3.49062	0.24163	4.15590
H	-9.02613	-0.46206	-0.14443
H	-10.54402	-4.11662	-2.36111
H	-8.92762	-3.72241	-1.73646
H	-9.90462	-5.82792	-0.83908
H	-12.30400	-4.49650	-0.43275
H	-11.54194	-3.08761	1.32326
H	-9.85637	-5.31974	2.53302
H	-9.65140	-2.02501	1.12829
H	-8.48914	-0.13590	2.28823
H	-6.75787	-0.71973	5.47022
H	-7.08402	0.41664	4.21695
H	-12.09510	-4.46219	2.29246
H	-12.69620	-6.38373	0.87616

****

ss-G6 B3LYP-D3/TZVP

O	10.78930	-5.54665	-0.29819
C	11.67585	-4.49857	-0.69593
C	10.95266	-3.16738	-0.66127
O	9.93693	-3.10969	-1.68446
C	10.22870	-2.86684	0.65540
O	10.25794	-1.43695	0.82868
C	8.81678	-3.35513	0.37792
C	8.64261	-2.96364	-1.08149
N	7.67411	-3.75357	-1.80796
C	7.36344	-5.09791	-1.67185
N	6.43896	-5.48992	-2.50807
C	6.12322	-4.36265	-3.24852
C	5.15655	-4.16333	-4.27601
O	4.35036	-4.95382	-4.75940
N	5.19310	-2.82575	-4.73913
C	6.00536	-1.82778	-4.26433
N	5.90599	-0.61268	-4.85994
N	6.88020	-2.01941	-3.30332
C	6.89138	-3.27990	-2.83213
P	9.74976	-0.74920	2.23232
O	10.79828	-0.89120	3.32670
O	8.36299	-1.24415	2.63593
O	9.72975	0.81250	1.75358
C	9.52129	1.29925	0.41910
C	8.07209	1.56615	0.06679
O	7.40813	0.34657	-0.31551
C	7.22142	2.19063	1.19371
O	6.50622	3.29562	0.60886
C	6.27341	1.06246	1.57674
C	6.07898	0.39857	0.22500
N	5.51023	-0.92197	0.23359
C	5.72320	-1.94978	1.14079
N	5.06927	-3.03751	0.82495
C	4.38805	-2.72689	-0.34153
C	3.48316	-3.49897	-1.12568
O	3.08466	-4.64804	-0.96165
N	3.01112	-2.74394	-2.22782
C	3.32272	-1.43831	-2.50326
N	2.77364	-0.89217	-3.62318
N	4.15591	-0.73683	-1.77278
C	4.65399	-1.41562	-0.72154
P	5.71924	4.37396	1.56385
O	6.70609	5.24528	2.32942

O	4.67782	3.69805	2.45309
O	5.01558	5.27626	0.39819
C	4.76997	4.88730	-0.96196
C	3.39782	4.28866	-1.19104
O	3.36917	2.90708	-0.78219
C	2.24798	4.99079	-0.43763
O	1.18278	5.18076	-1.38883
C	1.86311	3.97277	0.62585
C	2.11302	2.67806	-0.12819
N	2.18720	1.48378	0.67082
C	2.76275	1.31930	1.92293
N	2.63140	0.09844	2.37471
C	1.93816	-0.58333	1.38681
C	1.46203	-1.92533	1.33925
O	1.54832	-2.82091	2.17404
N	0.78475	-2.16960	0.11912
C	0.55553	-1.25359	-0.87284
N	-0.09432	-1.69323	-1.98660
N	0.98577	-0.01628	-0.81194
C	1.65967	0.26476	0.31986
P	-0.07531	6.17433	-1.03697
O	0.35268	7.63507	-1.05658
O	-0.78369	5.75672	0.24983
O	-1.00465	5.88198	-2.34883
C	-0.94252	4.74198	-3.21935
C	-1.92316	3.64225	-2.86511
O	-1.41484	2.83167	-1.78567
C	-3.31628	4.12895	-2.41712
O	-4.28079	3.26389	-3.04655
C	-3.29141	3.88642	-0.91528
C	-2.49690	2.59104	-0.87370
N	-1.97700	2.19464	0.40786
C	-1.48499	2.99724	1.42705
N	-1.11738	2.30369	2.47422
C	-1.37373	0.98207	2.14402
C	-1.22257	-0.21468	2.90360
O	-0.81729	-0.37007	4.05146
N	-1.63729	-1.33991	2.14787
C	-2.14726	-1.31241	0.87696
N	-2.44310	-2.51335	0.29869
N	-2.29711	-0.20343	0.19420
C	-1.90267	0.89938	0.86091
P	-5.85912	3.70162	-3.18149
O	-6.06186	4.55224	-4.42730
O	-6.39998	4.29084	-1.88524
O	-6.52135	2.21351	-3.32886
C	-5.95197	1.17919	-4.14074
C	-6.09191	-0.15845	-3.43778
O	-5.36032	-0.14897	-2.19465
C	-7.52555	-0.57007	-3.07284
O	-7.57244	-2.01136	-3.20522
C	-7.65079	-0.06021	-1.64686
C	-6.26373	-0.34519	-1.09728
N	-5.87529	0.45809	0.03694
C	-5.91973	1.83185	0.22118
N	-5.49181	2.18718	1.40771
C	-5.15734	1.00331	2.04771
C	-4.66884	0.74588	3.36110
O	-4.41712	1.52691	4.27431
N	-4.49033	-0.64856	3.56428
C	-4.76583	-1.63854	2.65542
N	-4.49799	-2.92932	2.99835
N	-5.21840	-1.38314	1.45161
C	-5.39325	-0.07562	1.20443
P	-8.47738	-3.04566	-2.31168
O	-8.53431	-4.35611	-3.07465
O	-9.79441	-2.43383	-1.85571
O	-7.59835	-3.22149	-0.93172
C	-6.53639	-4.16904	-0.79494
C	-6.78669	-5.05774	0.40828
O	-6.76942	-4.30391	1.63726
C	-8.13370	-5.77843	0.40762
O	-7.93814	-7.02287	1.08872
C	-9.04593	-4.82668	1.18324
C	-8.09539	-4.08621	2.13104
N	-8.37695	-2.66313	2.25849
C	-8.95687	-1.78898	1.34692
N	-8.96502	-0.55279	1.77146

C	-8.35432	-0.59051	3.01328
C	-8.06256	0.45661	3.93461
O	-8.31032	1.65623	3.86285
N	-7.37878	-0.05475	5.06617
C	-7.04076	-1.36579	5.27664
N	-6.29207	-1.64491	6.37478
N	-7.34626	-2.32814	4.43497
C	-7.98000	-1.89203	3.32714
H	12.53762	-4.43970	-0.02149
H	12.04346	-4.66474	-1.71294
H	11.68465	-2.38230	-0.85968
H	10.69616	-3.35739	1.50886
H	8.79267	-4.43438	0.50068
H	8.31889	-1.92776	-1.17648
H	7.86043	-5.72214	-0.94953
H	4.51987	-2.60972	-5.46266
H	6.35099	0.14270	-4.36187
H	5.04085	-0.36976	-5.31745
H	8.08848	-2.88851	1.02750
H	10.06323	2.24397	0.37063
H	9.94985	0.60854	-0.30580
H	8.06496	2.25279	-0.78494
H	7.83040	2.54214	2.02465
H	6.78386	0.37607	2.25103
H	5.43597	1.00105	-0.41907
H	6.40095	-1.82102	1.96948
H	2.35141	-3.22959	-2.82191
H	2.81542	0.11558	-3.65303
H	1.90089	-1.27762	-3.95226
H	5.35709	1.43272	2.01844
H	4.84345	5.80549	-1.54554
H	5.53453	4.19002	-1.30008
H	3.18979	4.34448	-2.26348
H	2.55268	5.95062	-0.02460
H	2.55557	4.04697	1.46395
H	1.33145	2.49664	-0.86816
H	3.28821	2.13450	2.39715
H	0.40802	-3.10389	0.02372
H	-0.47515	-0.95368	-2.55821
H	-0.70732	-2.48889	-1.88509
H	0.83997	4.09402	0.95925
H	-1.19440	5.11785	-4.21160
H	0.06726	4.33581	-3.23967
H	-2.04321	3.01247	-3.75094
H	-3.49891	5.16693	-2.68860
H	-2.72432	4.67854	-0.42693
H	-3.09690	1.75036	-1.22605
H	-1.39296	4.06407	1.28974
H	-1.54515	-2.22794	2.62459
H	-3.05979	-2.42503	-0.49626
H	-2.71320	-3.26108	0.92340
H	-4.28448	3.80875	-0.48969
H	-6.47045	1.14391	-5.10213
H	-4.89417	1.37201	-4.31418
H	-5.65850	-0.92028	-4.08860
H	-8.26954	-0.14405	-3.74383
H	-7.82953	1.01235	-1.68635
H	-6.19240	-1.37095	-0.74785
H	-6.25094	2.51159	-0.55050
H	-4.15674	-0.89990	4.48662
H	-5.09529	-3.58793	2.50135
H	-4.41228	-3.13439	3.98277
H	-8.43726	-0.53704	-1.07517
H	-6.45254	-4.78855	-1.68914
H	-5.60703	-3.61860	-0.64486
H	-5.97617	-5.78592	0.46642
H	-8.49665	-5.95144	-0.60730
H	-9.52867	-4.14409	0.49141
H	-8.12609	-4.48246	3.14373
H	-9.34230	-2.11743	0.39672
H	-7.07767	0.64686	5.73019
H	-6.20780	-2.62140	6.61032
H	-6.29011	-1.00627	7.15436
H	-9.81928	-5.35948	1.73343
H	-8.78751	-7.47816	1.13412
H	11.24505	-6.38769	-0.41177

****

ss-T6 B3LYP-D3/TZVP

O	12.15368	-4.72965	-2.21787
C	12.00965	-3.55474	-3.01352
C	10.84383	-2.68971	-2.57619
O	9.62306	-3.44301	-2.74673
C	10.87415	-2.25109	-1.09777
O	10.49952	-0.86536	-1.07586
C	9.80959	-3.12716	-0.44580
C	8.80918	-3.22748	-1.58444
N	7.80188	-4.26973	-1.50837
C	6.60247	-4.02930	-2.17263
O	6.35590	-2.99193	-2.76428
N	5.69770	-5.06235	-2.09126
C	5.84949	-6.29258	-1.45090
O	4.93865	-7.10987	-1.46645
C	7.14197	-6.47978	-0.81386
C	7.40649	-7.76326	-0.09260
C	8.03908	-5.47893	-0.88557
P	10.59694	0.01029	0.31071
O	12.02975	0.08164	0.81492
O	9.59815	-0.48369	1.35752
O	10.15438	1.46395	-0.28415
C	9.45061	1.70412	-1.51322
C	7.94527	1.72876	-1.36282
O	7.41508	0.39173	-1.27497
C	7.42565	2.48918	-0.12186
O	6.41680	3.40261	-0.59019
C	6.83013	1.38198	0.73919
C	6.32776	0.43847	-0.34174
N	5.97904	-0.90771	0.06932
C	4.81920	-1.46921	-0.45288
O	4.07916	-0.90345	-1.23925
N	4.55434	-2.73627	0.01691
C	5.30281	-3.49912	0.91346
O	4.92248	-4.62048	1.22978
C	6.52067	-2.85715	1.36858
C	7.41314	-3.59567	2.31702
C	6.79821	-1.61401	0.92477
P	5.76328	4.51604	0.42398
O	6.80980	5.50990	0.90598
O	4.97883	3.85301	1.55615
O	4.77184	5.26671	-0.63185
C	4.22164	4.71248	-1.83687
C	2.88447	4.02671	-1.65101
O	3.06101	2.69123	-1.14282
C	1.90833	4.73701	-0.68611
O	0.65269	4.86051	-1.38010
C	1.81067	3.76674	0.48497
C	1.97358	2.44216	-0.24400
N	2.26657	1.27703	0.56751
C	1.57017	0.10378	0.30403
O	0.73814	-0.01418	-0.58013
N	1.87591	-0.93012	1.15996
C	2.81182	-0.94476	2.19273
O	2.96733	-1.95746	2.86632
C	3.54775	0.29476	2.34757
C	4.60313	0.37399	3.40670
C	3.24438	1.33010	1.53714
P	-0.56651	5.74572	-0.72636
O	-0.18306	7.21304	-0.60539
O	-1.06475	5.12980	0.58169
O	-1.66586	5.58241	-1.92022
C	-1.73007	4.52718	-2.89255
C	-2.59737	3.35772	-2.47647
O	-1.87440	2.47146	-1.60037
C	-3.89847	3.74112	-1.73656
O	-4.97664	3.04640	-2.39186
C	-3.66589	3.21171	-0.32795
C	-2.81335	1.99006	-0.62990
N	-2.10541	1.39464	0.48712
C	-2.14130	0.01236	0.62543
O	-2.71550	-0.73841	-0.14614
N	-1.49173	-0.45282	1.74657
C	-0.78595	0.27953	2.69953
O	-0.25889	-0.29259	3.64712
C	-0.73627	1.70474	2.43819
C	0.02519	2.57906	3.38565

C	-1.38872	2.18565	1.35959
P	-6.53026	3.41767	-2.00795
O	-6.87079	4.84680	-2.40267
O	-6.83453	3.09646	-0.54439
O	-7.29549	2.39199	-3.01897
C	-6.77365	1.16704	-3.55855
C	-7.05802	-0.05625	-2.71192
O	-6.10445	-0.16697	-1.63830
C	-8.45816	-0.09349	-2.05909
O	-9.02986	-1.37803	-2.36619
C	-8.15202	0.04160	-0.57335
C	-6.81456	-0.67783	-0.50274
N	-6.02264	-0.48075	0.69680
C	-5.40609	-1.58925	1.26500
O	-5.48388	-2.72111	0.81707
N	-4.71079	-1.30948	2.42026
C	-4.52407	-0.07260	3.03579
O	-3.86876	0.00654	4.06869
C	-5.14119	1.03860	2.33865
C	-4.97754	2.41624	2.90174
C	-5.84986	0.78327	1.21940
P	-10.60745	-1.67930	-2.02204
O	-11.52712	-0.75958	-2.81288
O	-10.87268	-1.66776	-0.51697
O	-10.71323	-3.18312	-2.64693
C	-9.64030	-4.12994	-2.75557
C	-9.38654	-4.94397	-1.50585
O	-8.63969	-4.18094	-0.54573
C	-10.64331	-5.47604	-0.77745
O	-10.59101	-6.90602	-0.84426
C	-10.48856	-4.93293	0.64617
C	-8.98265	-4.70985	0.73463
N	-8.53049	-3.79373	1.76664
C	-7.55984	-4.22478	2.66051
O	-7.05910	-5.33792	2.64579
N	-7.21475	-3.27999	3.60277
C	-7.69074	-1.97503	3.72931
O	-7.26841	-1.25251	4.62621
C	-8.67228	-1.59713	2.73121
C	-9.23885	-0.21160	2.77577
C	-9.03880	-2.51339	1.81008
H	12.93414	-2.98635	-2.90834
H	11.88559	-3.81365	-4.06962
H	10.80869	-1.79367	-3.20088
H	11.85809	-2.37530	-0.64749
H	10.25720	-4.09054	-0.21027
H	8.26693	-2.28907	-1.68891
H	9.01008	-5.57683	-0.42873
H	4.81470	-4.90159	-2.55918
H	9.40175	-2.67993	0.45292
H	11.37373	-5.27646	-2.37338
H	9.77544	2.69009	-1.84636
H	9.73378	0.96622	-2.26147
H	7.53385	2.21413	-2.25287
H	8.21439	3.04337	0.38318
H	7.62736	0.91409	1.31391
H	5.43837	0.84246	-0.82187
H	7.71777	-1.10892	1.19701
H	3.70598	-3.15875	-0.33703
H	6.05182	1.75098	1.39501
H	4.07884	5.56180	-2.50517
H	4.92865	4.02017	-2.29091
H	2.40597	3.96951	-2.63334
H	2.26155	5.72370	-0.39354
H	2.65014	3.93627	1.15710
H	1.07268	2.19498	-0.80260
H	3.78149	2.27089	1.58934
H	1.35865	-1.78552	1.00490
H	0.87063	3.86589	1.01223
H	-2.17101	4.98002	-3.78071
H	-0.72889	4.17467	-3.13436
H	-2.87135	2.81059	-3.38346
H	-4.08573	4.81261	-1.76250
H	-3.09438	3.94477	0.23843
H	-3.41844	1.19237	-1.05682
H	-1.36436	3.23924	1.10342
H	-1.52578	-1.45500	1.87973
H	-4.59731	2.98273	0.17273

H	-7.27398	1.04092	-4.51870
H	-5.70151	1.25517	-3.72680
H	-6.96321	-0.93344	-3.35898
H	-9.10788	0.69541	-2.43235
H	-8.03244	1.09575	-0.33035
H	-6.95128	-1.75376	-0.59628
H	-6.30996	1.57803	0.64353
H	-4.27597	-2.10706	2.86555
H	-8.92436	-0.40750	0.03636
H	-9.94240	-4.81392	-3.54855
H	-8.72345	-3.62450	-3.05641
H	-8.79148	-5.81519	-1.80349
H	-11.55695	-5.11243	-1.24663
H	-11.01000	-3.98085	0.73263
H	-8.45624	-5.64639	0.91265
H	-9.74526	-2.28099	1.01923
H	-6.52062	-3.57625	4.27676
H	-10.86034	-5.62512	1.39975
H	-11.41639	-7.25267	-0.48498
H	8.41426	-7.77474	0.32061
H	6.69491	-7.89903	0.72481
H	7.29494	-8.61826	-0.76225
H	8.29446	-3.00228	2.55665
H	6.88689	-3.82276	3.24669
H	7.73714	-4.54880	1.89401
H	5.03226	1.37380	3.44914
H	4.18577	0.13504	4.38684
H	5.40655	-0.34179	3.21744
H	-0.07262	3.62697	3.10703
H	-0.34411	2.45616	4.40568
H	1.08644	2.31936	3.39533
H	-5.53964	3.14166	2.31586
H	-5.33130	2.45565	3.93389
H	-3.92701	2.71645	2.91108
H	-10.00896	-0.08423	2.01682
H	-9.67929	-0.00572	3.75322
H	-8.46107	0.53769	2.61097

****

ss-A2C2A2 B3LYP-D3/TZVP

O	12.07931	-4.82353	-1.25535
C	12.21194	-3.59782	-1.97735
C	11.03280	-2.70001	-1.67266
O	9.83025	-3.23986	-2.26132
C	10.73194	-2.53353	-0.17505
O	10.29637	-1.17942	0.03310
C	9.57228	-3.49363	0.05156
C	8.81440	-3.32580	-1.25553
N	7.90257	-4.39022	-1.59760
C	7.82062	-5.67595	-1.09896
N	6.87939	-6.39027	-1.66419
C	6.30953	-5.54174	-2.59932
C	5.24976	-5.69495	-3.51145
N	4.55583	-6.84347	-3.63153
N	4.93598	-4.65494	-4.30441
C	5.62022	-3.51271	-4.17154
N	6.62451	-3.24389	-3.33779
C	6.93037	-4.29488	-2.57260
P	10.52103	-0.48943	1.50826
O	12.01010	-0.33163	1.78608
O	9.73092	-1.21037	2.59437
O	9.76709	0.92889	1.23767
C	9.82389	1.67033	0.01103
C	8.42031	2.07696	-0.39876
O	7.66178	0.92492	-0.81860
C	7.59953	2.72492	0.72218
O	6.70916	3.66427	0.09028
C	6.83356	1.54364	1.29723
C	6.52003	0.75546	0.03486
N	6.26122	-0.64853	0.24908
C	6.81197	-1.49842	1.19199
N	6.29938	-2.70529	1.15077
C	5.36488	-2.66161	0.12863
C	4.46655	-3.60928	-0.38644
N	4.33505	-4.84912	0.14195
N	3.67694	-3.24998	-1.41054
C	3.75725	-1.99953	-1.87928

N	4.54509	-1.01153	-1.45737
C	5.33098	-1.39376	-0.44944
P	5.75325	4.65215	0.99109
O	6.57350	5.72181	1.69650
O	4.84258	3.85643	1.92491
O	4.92437	5.36369	-0.22036
C	4.56250	4.76865	-1.47780
C	3.23581	4.03472	-1.47196
O	3.39991	2.68643	-0.99827
C	2.13093	4.67566	-0.60104
O	0.96231	4.82283	-1.43473
C	1.90881	3.64123	0.49554
C	2.20304	2.36100	-0.26983
N	2.41914	1.15632	0.50053
C	1.78718	-0.05152	0.08195
O	1.05767	-0.04077	-0.90748
N	2.02373	-1.16865	0.81448
C	2.84341	-1.14373	1.85131
N	3.01299	-2.29290	2.54242
C	3.51873	0.04277	2.26774
C	3.27476	1.16715	1.55741
P	-0.27743	5.75778	-0.89988
O	0.12977	7.22315	-0.82989
O	-0.85825	5.21881	0.40769
O	-1.31714	5.56122	-2.14019
C	-1.35162	4.49462	-3.10279
C	-2.32424	3.38288	-2.75648
O	-1.71459	2.43488	-1.85985
C	-3.62566	3.84763	-2.07105
O	-4.70965	3.11358	-2.67504
C	-3.42550	3.40514	-0.62833
C	-2.69579	2.09230	-0.86271
N	-2.03721	1.46776	0.26216
C	-2.06745	0.04610	0.36176
O	-2.65659	-0.60771	-0.49786
N	-1.44556	-0.52301	1.42454
C	-0.78052	0.21349	2.29748
N	-0.20872	-0.41918	3.34579
C	-0.68018	1.63196	2.18571
C	-1.32490	2.21246	1.14867
P	-6.25439	3.65913	-2.53037
O	-6.57713	4.61087	-3.67349
O	-6.54248	4.18110	-1.12929
O	-7.04211	2.23447	-2.66194
C	-6.75377	1.26422	-3.67569
C	-7.11169	-0.11340	-3.15296
O	-6.22464	-0.48834	-2.07853
C	-8.52831	-0.23330	-2.58116
O	-8.96607	-1.57975	-2.85522
C	-8.30738	0.03086	-1.10081
C	-6.95757	-0.62789	-0.85465
N	-6.20387	-0.06487	0.24106
C	-6.00042	1.26502	0.55164
N	-5.26493	1.43211	1.62555
C	-4.96370	0.14875	2.05376
C	-4.21595	-0.35034	3.13057
N	-3.60758	0.45650	4.03356
N	-4.14364	-1.68187	3.29556
C	-4.76188	-2.47543	2.41381
N	-5.46783	-2.11934	1.34036
C	-5.54073	-0.79483	1.20489
P	-10.34068	-2.20674	-2.19816
O	-10.89232	-3.20701	-3.19974
O	-11.27052	-1.11737	-1.69664
O	-9.76777	-2.99269	-0.86676
C	-9.02538	-4.21054	-1.04054
C	-9.13723	-5.08393	0.19201
O	-8.40471	-4.53138	1.30191
C	-10.56227	-5.30025	0.70298
O	-10.61131	-6.62418	1.24696
C	-10.70905	-4.22565	1.77737
C	-9.28743	-4.07931	2.32789
N	-8.93736	-2.72293	2.70858
C	-9.16838	-1.56457	1.99349
N	-8.61318	-0.50544	2.52637
C	-7.96529	-0.98112	3.65526
C	-7.17282	-0.35970	4.63382
N	-6.91531	0.97110	4.63190

N	-6.67279	-1.11824	5.62513
C	-6.92569	-2.43208	5.62400
N	-7.64089	-3.13384	4.74390
C	-8.14424	-2.36047	3.77838
H	13.12742	-3.07066	-1.68664
H	12.24494	-3.77981	-3.05586
H	11.22850	-1.71721	-2.10645
H	11.60013	-2.75344	0.44381
H	9.97117	-4.50280	0.13497
H	8.23054	-2.40363	-1.25705
H	8.47071	-6.02183	-0.31387
H	5.31426	-2.70497	-4.82671
H	4.69890	-7.59033	-2.97319
H	3.73551	-6.86319	-4.21437
H	8.98409	-3.24370	0.92761
H	10.42987	2.56456	0.17148
H	10.26899	1.07230	-0.78263
H	8.49892	2.76816	-1.24043
H	8.22232	3.23747	1.45488
H	7.49485	0.98304	1.95148
H	5.63729	1.15089	-0.46842
H	7.61541	-1.19520	1.84751
H	3.09282	-1.76316	-2.70155
H	5.06692	-5.19311	0.74245
H	3.83629	-5.53513	-0.40227
H	5.94682	1.85446	1.83399
H	4.48741	5.60430	-2.17332
H	5.34996	4.09689	-1.81712
H	2.87381	4.00297	-2.50430
H	2.42747	5.64894	-0.21581
H	2.64948	3.79246	1.27885
H	1.39813	2.12630	-0.96239
H	3.75790	2.11422	1.76706
H	4.20304	0.04559	3.10217
H	2.67491	-3.14419	2.12085
H	3.78492	-2.37817	3.18272
H	0.90708	3.69383	0.90145
H	-1.67510	4.95829	-4.03496
H	-0.35590	4.07663	-3.24110
H	-2.58452	2.87104	-3.68724
H	-3.78668	4.91840	-2.17707
H	-2.77944	4.12126	-0.12274
H	-3.37389	1.34005	-1.25800
H	-1.28393	3.27863	0.95959
H	-0.11011	2.21954	2.88873
H	-0.14105	-1.42373	3.30784
H	0.46050	0.06493	3.92058
H	-4.36156	3.29884	-0.09484
H	-7.33997	1.48178	-4.57224
H	-5.69345	1.28245	-3.92729
H	-6.98437	-0.82756	-3.96830
H	-9.22527	0.46968	-3.03406
H	-8.24155	1.10424	-0.94735
H	-7.06384	-1.68661	-0.61932
H	-6.39901	2.06641	-0.05207
H	-4.67352	-3.54025	2.59669
H	-3.46169	1.42063	3.77959
H	-2.89936	0.03669	4.61573
H	-9.09464	-0.36832	-0.47549
H	-9.41544	-4.75972	-1.89958
H	-7.97447	-3.97280	-1.21971
H	-8.68607	-6.04826	-0.04968
H	-11.29970	-5.18595	-0.09368
H	-11.04538	-3.30117	1.31519
H	-9.12587	-4.68444	3.21880
H	-9.73920	-1.58717	1.08167
H	-6.48835	-2.99470	6.44111
H	-7.03808	1.46349	3.75961
H	-6.12676	1.26851	5.18702
H	-11.41792	-4.50456	2.55498
H	-11.48906	-6.76392	1.62224
H	12.81342	-5.39839	-1.49749

****

ds-A4 B3LYP-D3/TZVP

O	-3.09665	-6.89812	6.10368
C	-4.23031	-6.36297	6.79317

C	-4.86081	-5.25063	5.97945
O	-4.00221	-4.09389	5.92821
C	-5.15239	-5.60618	4.51834
O	-6.30958	-4.83802	4.13763
C	-3.91061	-5.11006	3.79594
C	-3.58330	-3.84324	4.57611
N	-2.17860	-3.45198	4.59002
C	-1.86799	-2.10933	4.38470
O	-2.69755	-1.26513	4.08406
N	-0.54137	-1.79773	4.54408
C	0.46154	-2.63030	4.99760
O	1.59207	-2.18077	5.21663
C	0.07578	-4.01376	5.20517
C	1.11118	-4.99520	5.65859
C	-1.21778	-4.34479	5.01214
P	-7.11603	-5.12624	2.73731
O	-8.02648	-6.33990	2.86626
O	-6.17440	-5.19005	1.53774
O	-8.04830	-3.78383	2.70432
C	-7.76920	-2.54126	3.36847
C	-7.03465	-1.52877	2.51684
O	-5.63720	-1.84890	2.42631
C	-7.54118	-1.38232	1.06504
O	-7.69950	0.02991	0.84078
C	-6.39521	-1.95391	0.23997
C	-5.20968	-1.52508	1.09068
N	-3.92898	-2.15700	0.84497
C	-2.77665	-1.37508	0.98082
O	-2.79975	-0.16120	1.08312
N	-1.60265	-2.08749	0.97723
C	-1.46169	-3.46034	0.90457
O	-0.35148	-3.98271	1.06420
C	-2.67840	-4.19674	0.64994
C	-2.61516	-5.68117	0.46934
C	-3.84375	-3.51709	0.66801
P	-8.40063	0.64041	-0.50779
O	-9.90409	0.40063	-0.51052
O	-7.70429	0.16954	-1.78361
O	-8.11887	2.22572	-0.22742
C	-7.12052	2.77100	0.64971
C	-5.80739	3.08462	-0.03312
O	-5.04178	1.88591	-0.24978
C	-5.93536	3.77712	-1.40864
O	-5.05543	4.91466	-1.38978
C	-5.42071	2.72117	-2.37438
C	-4.36997	2.04570	-1.50757
N	-3.86641	0.76416	-1.96044
C	-2.49039	0.53440	-1.91712
O	-1.67440	1.38598	-1.60308
N	-2.11553	-0.73633	-2.28356
C	-2.94762	-1.77690	-2.65345
O	-2.47657	-2.89297	-2.90582
C	-4.35608	-1.46199	-2.70697
C	-5.32239	-2.52702	-3.12362
C	-4.74424	-0.22189	-2.34259
P	-5.18743	6.11608	-2.50766
O	-6.07748	7.22478	-1.96192
O	-5.56194	5.57857	-3.88052
O	-3.61664	6.56897	-2.57737
C	-2.85269	6.78033	-1.37942
C	-1.40649	6.42373	-1.64039
O	-1.30559	5.02583	-1.97741
C	-0.74061	7.19970	-2.78594
O	0.59812	7.49701	-2.36858
C	-0.77350	6.21076	-3.94396
C	-0.63977	4.86821	-3.22917
N	-1.21326	3.73466	-3.94674
C	-0.42776	2.60179	-4.13940
O	0.73479	2.51371	-3.77003
N	-1.05843	1.58053	-4.80711
C	-2.35006	1.57444	-5.29544
O	-2.80079	0.56348	-5.84827
C	-3.09879	2.79860	-5.10636
C	-4.47375	2.90199	-5.69003
C	-2.50572	3.79646	-4.41722
O	4.80315	-7.35155	-6.11210
C	5.87941	-6.43490	-6.31989
C	5.80813	-5.32029	-5.29869

O	4.66651	-4.47365	-5.55407
C	5.65774	-5.79319	-3.84403
O	6.35455	-4.85672	-3.00434
C	4.16233	-5.66281	-3.59657
C	3.87737	-4.37946	-4.35992
N	2.50159	-4.13798	-4.71315
C	1.46835	-5.03120	-4.92411
N	0.34278	-4.44800	-5.25219
C	0.63875	-3.09505	-5.26688
C	-0.14339	-1.94727	-5.49508
N	-1.44975	-1.98900	-5.79990
N	0.47729	-0.74927	-5.42582
C	1.78300	-0.69332	-5.13207
N	2.60553	-1.70562	-4.88127
C	1.97743	-2.88491	-4.94764
P	7.23690	-5.39551	-1.72850
O	8.42899	-6.20296	-2.22600
O	6.36580	-6.08166	-0.68536
O	7.65918	-3.94568	-1.11185
C	8.24720	-2.90432	-1.90460
C	7.70686	-1.55740	-1.45933
O	6.30814	-1.43612	-1.77711
C	7.80051	-1.29172	0.04537
O	7.88844	0.13550	0.20963
C	6.46041	-1.79169	0.55874
C	5.52807	-1.37063	-0.57034
N	4.33775	-2.17452	-0.71809
C	4.18310	-3.53905	-0.54784
N	2.98660	-3.96008	-0.87746
C	2.31173	-2.82592	-1.29763
C	1.01085	-2.62337	-1.79439
N	0.13689	-3.61714	-1.98214
N	0.66304	-1.36181	-2.12817
C	1.55643	-0.37115	-2.00062
N	2.80953	-0.45726	-1.56239
C	3.13571	-1.70776	-1.21600
P	8.48853	0.78835	1.59655
O	9.98501	1.02343	1.44216
O	8.06170	0.00169	2.82694
O	7.64092	2.18537	1.60893
C	7.54563	3.03399	0.45778
C	6.27115	3.85167	0.54114
O	5.11450	2.99715	0.45751
C	6.09068	4.65062	1.83475
O	5.30762	5.80470	1.48097
C	5.30498	3.69178	2.71277
C	4.39424	3.00657	1.70101
N	4.00720	1.65706	2.04721
C	4.77447	0.66734	2.63651
N	4.16344	-0.49144	2.68121
C	2.93492	-0.27063	2.08282
C	1.86219	-1.12833	1.77625
N	1.86661	-2.43707	2.04384
N	0.79078	-0.58297	1.16235
C	0.79692	0.71798	0.84103
N	1.76803	1.60240	1.05292
C	2.81886	1.05565	1.67609
P	5.00548	7.04427	2.52081
O	5.77958	8.27371	2.06863
O	5.17239	6.63033	3.97366
O	3.39630	7.23336	2.26179
C	2.90379	7.41086	0.92633
C	1.40606	7.20757	0.89140
O	1.06611	5.84388	1.22622
C	0.59100	8.08220	1.84408
O	-0.65575	8.35289	1.18888
C	0.41575	7.19112	3.06743
C	0.36321	5.78414	2.46823
N	0.94761	4.76615	3.32191
C	2.16397	4.81461	3.97805
N	2.47475	3.68565	4.56565
C	1.41875	2.83835	4.28307
C	1.19947	1.47899	4.56252
N	2.06002	0.71826	5.26056
N	0.05547	0.93144	4.10484
C	-0.80401	1.67408	3.39104
N	-0.67801	2.95328	3.04788
C	0.45918	3.48789	3.50756

H	-4.98470	-7.14008	6.95761
H	-3.93541	-5.95487	7.76425
H	-5.79019	-4.96296	6.47399
H	-5.34178	-6.66977	4.37558
H	-3.12274	-5.85369	3.88427
H	-4.12402	-2.99069	4.17587
H	-1.58747	-5.34067	5.21446
H	-0.30611	-0.79185	4.37852
H	-4.10715	-4.91039	2.75216
H	-8.74259	-2.12353	3.62793
H	-7.20732	-2.72190	4.28321
H	-7.14570	-0.55722	3.00857
H	-8.48785	-1.89349	0.89785
H	-6.48376	-3.03887	0.21978
H	-5.03845	-0.45444	0.99589
H	-4.79001	-4.03586	0.61580
H	-0.72882	-1.51633	1.05525
H	-6.38486	-1.55341	-0.76429
H	-7.53949	3.70555	1.02435
H	-6.94824	2.09894	1.48807
H	-5.24553	3.74970	0.63000
H	-6.95646	4.08913	-1.61924
H	-6.22288	2.02416	-2.61248
H	-3.49561	2.68310	-1.39103
H	-5.79207	0.04878	-2.28896
H	-1.08858	-0.93122	-2.26248
H	-5.02873	3.16634	-3.27680
H	-2.92264	7.82728	-1.07567
H	-3.23210	6.15106	-0.57548
H	-0.85629	6.60611	-0.71802
H	-1.27780	8.12194	-3.01379
H	-1.73033	6.28879	-4.45626
H	0.40245	4.60972	-3.06245
H	-3.03456	4.71012	-4.18844
H	-0.47799	0.73908	-5.00845
H	0.02642	6.37425	-4.66438
H	1.04766	7.95446	-3.08934
H	6.84650	-6.93790	-6.20798
H	5.83188	-5.99604	-7.32116
H	6.71711	-4.72152	-5.38509
H	6.03610	-6.80359	-3.69899
H	3.65372	-6.50720	-4.05774
H	4.19626	-3.50833	-3.78559
H	1.60793	-6.09323	-4.81491
H	2.20382	0.30370	-5.07723
H	-1.95133	-2.83935	-5.60107
H	-1.97417	-1.11310	-5.81587
H	3.90899	-5.58896	-2.54416
H	9.33194	-2.92396	-1.77504
H	8.01012	-3.04671	-2.95864
H	8.25176	-0.78355	-2.00337
H	8.65524	-1.78662	0.50510
H	6.49668	-2.87334	0.65001
H	5.17959	-0.34625	-0.44078
H	4.98693	-4.17778	-0.21908
H	1.20743	0.61091	-2.29672
H	0.32986	-4.51363	-1.57019
H	-0.80309	-3.41083	-2.32008
H	6.18449	-1.35131	1.50853
H	8.41053	3.70134	0.41670
H	7.51516	2.43167	-0.45029
H	6.25683	4.53140	-0.31257
H	7.03895	4.95661	2.27498
H	5.99694	2.97951	3.15333
H	3.46385	3.55505	1.55715
H	5.78353	0.83960	2.97347
H	-0.09499	1.07700	0.34082
H	2.65363	-2.83983	2.52134
H	1.10815	-3.03394	1.71304
H	4.75528	4.19179	3.49915
H	3.14309	8.41692	0.57116
H	3.36944	6.68166	0.26206
H	1.08788	7.39103	-0.13483
H	1.10567	9.01527	2.08188
H	1.28057	7.29991	3.71778
H	-0.65719	5.45457	2.27672
H	2.78483	5.69412	3.95718
H	-1.68746	1.15158	3.04305

H	2.97427	1.09340	5.45276
H	1.94835	-0.29622	5.24995
H	-0.48191	7.42413	3.63787
H	-1.22717	8.82412	1.80702
H	0.67021	-5.98028	5.80836
H	1.91345	-5.08406	4.92218
H	1.57158	-4.67393	6.59528
H	-3.61859	-6.09781	0.39008
H	-2.05647	-5.94598	-0.43159
H	-2.10789	-6.15498	1.31225
H	-6.34339	-2.14841	-3.10190
H	-5.10346	-2.87737	-4.13471
H	-5.25855	-3.39525	-2.46375
H	-4.95110	3.83066	-5.38103
H	-4.42625	2.87154	-6.78162
H	-5.09545	2.06153	-5.37582
H	-2.66821	-7.54175	6.67854
H	4.83343	-8.01537	-6.80954

****

ds-G4 B3LYP-D3/TZVP

O	5.59152	-6.52795	-6.37415
C	6.71960	-5.67424	-6.57667
C	6.80256	-4.64725	-5.46563
O	5.70955	-3.71034	-5.54638
C	6.74208	-5.22555	-4.04800
O	7.52715	-4.34849	-3.22041
C	5.26367	-5.12106	-3.70891
C	4.90201	-3.79378	-4.35838
N	3.51152	-3.64407	-4.71342
C	2.60912	-4.60955	-5.13702
N	1.44105	-4.11199	-5.44235
C	1.56477	-2.74720	-5.22719
C	0.62591	-1.68987	-5.36021
O	-0.56052	-1.76487	-5.71732
N	1.18439	-0.45089	-5.02791
C	2.48440	-0.24784	-4.63642
N	2.86466	1.02580	-4.42798
N	3.35771	-1.23300	-4.49850
C	2.84904	-2.43990	-4.78558
P	7.87779	-4.72263	-1.66115
O	8.92098	-5.82881	-1.58128
O	6.61915	-5.00233	-0.84369
O	8.58281	-3.31510	-1.22437
C	8.37413	-2.02948	-1.83140
C	7.27358	-1.20921	-1.19228
O	5.97606	-1.63170	-1.65501
C	7.21985	-1.26769	0.35043
O	7.01104	0.06514	0.85130
C	5.96669	-2.08357	0.62324
C	5.08873	-1.60845	-0.52254
N	3.91637	-2.38639	-0.80506
C	3.77534	-3.76781	-0.82055
N	2.57491	-4.14217	-1.18003
C	1.87772	-2.96330	-1.41084
C	0.54301	-2.72792	-1.83612
O	-0.32802	-3.56176	-2.13066
N	0.25665	-1.36149	-1.91518
C	1.14873	-0.34693	-1.67813
N	0.71397	0.90353	-1.90230
N	2.39972	-0.56215	-1.30188
C	2.70115	-1.86273	-1.18589
P	8.12848	0.77159	1.81776
O	9.50023	0.73802	1.15380
O	8.06826	0.22394	3.23706
O	7.47914	2.26953	1.86551
C	7.21735	3.01290	0.66700
C	5.88866	3.73661	0.77881
O	4.79082	2.80667	0.76367
C	5.70024	4.56052	2.05638
O	4.81954	5.64193	1.69829
C	5.00505	3.57931	2.98781
C	4.09179	2.83781	2.02211
N	3.73580	1.49270	2.40221
C	4.54537	0.49251	2.92563
N	3.94835	-0.66872	2.95884
C	2.68445	-0.44754	2.42719

C	1.62089	-1.34021	2.12740
O	1.59346	-2.57527	2.25449
N	0.50861	-0.66743	1.61643
C	0.44534	0.67735	1.35159
N	-0.70564	1.13914	0.85853
N	1.46864	1.49938	1.54347
C	2.53814	0.89263	2.07651
P	4.56316	6.94533	2.66659
O	5.37025	8.13008	2.15580
O	4.72282	6.60320	4.13956
O	2.95880	7.15608	2.39914
C	2.44077	7.23342	1.06437
C	0.94298	7.01496	1.07540
O	0.62050	5.66702	1.48012
C	0.14779	7.92658	2.01097
O	-1.12442	8.14968	1.38771
C	0.02603	7.09266	3.27991
C	-0.04496	5.65970	2.74830
N	0.56436	4.68173	3.62594
C	1.81213	4.75920	4.23257
N	2.12549	3.66889	4.87834
C	1.04586	2.81951	4.69894
C	0.86446	1.46631	5.07825
O	1.64959	0.73960	5.70935
N	-0.36033	0.95993	4.63245
C	-1.29461	1.66533	3.91577
N	-2.44644	1.02705	3.63634
N	-1.11001	2.91787	3.52471
C	0.06828	3.42885	3.91467
O	-2.02148	-7.66260	4.98347
C	-3.17105	-7.65308	5.83635
C	-4.10285	-6.53224	5.42348
O	-3.54386	-5.24401	5.75762
C	-4.41069	-6.49197	3.92246
O	-5.80410	-6.15386	3.78571
C	-3.47846	-5.40810	3.40185
C	-3.32215	-4.45882	4.58430
N	-2.00039	-3.83584	4.68307
C	-1.86821	-2.43595	4.63904
O	-2.85085	-1.73495	4.35694
N	-0.65623	-1.90088	4.91686
C	0.40287	-2.67443	5.17729
N	1.54719	-2.08348	5.51745
C	0.30660	-4.09997	5.13179
C	-0.91240	-4.62967	4.90634
P	-6.51028	-6.08465	2.29826
O	-7.96587	-6.48128	2.47996
O	-5.70185	-6.83506	1.25486
O	-6.39073	-4.48829	1.92724
C	-7.06673	-3.53304	2.75445
C	-7.10363	-2.19053	2.05710
O	-5.78217	-1.65926	1.87121
C	-7.73568	-2.20292	0.66344
O	-8.38676	-0.92981	0.50726
C	-6.53656	-2.41163	-0.25184
C	-5.38567	-1.74314	0.49523
N	-4.07963	-2.41554	0.41132
C	-2.94957	-1.64125	0.76938
O	-3.07690	-0.41843	0.90136
N	-1.77101	-2.26973	0.96751
C	-1.64131	-3.58494	0.77542
N	-0.48419	-4.15521	1.10742
C	-2.72943	-4.35878	0.27382
C	-3.92550	-3.74580	0.15802
P	-8.90566	-0.27444	-0.90882
O	-10.29418	0.29405	-0.66728
O	-8.73725	-1.20889	-2.09430
O	-7.81367	0.94329	-1.10320
C	-7.63685	1.86895	-0.02225
C	-6.47831	2.79444	-0.31513
O	-5.25762	2.04388	-0.43869
C	-6.59719	3.63170	-1.59426
O	-6.03230	4.91778	-1.27819
C	-5.79163	2.83107	-2.60687
C	-4.71110	2.17172	-1.75602
N	-4.26448	0.86215	-2.22698
C	-2.88790	0.54671	-2.14958
O	-2.08278	1.42750	-1.83691

N	-2.50249	-0.71934	-2.43005
C	-3.38282	-1.64677	-2.80937
N	-2.93853	-2.88642	-3.02358
C	-4.76559	-1.32858	-2.96984
C	-5.15922	-0.08441	-2.62597
P	-5.65039	6.06712	-2.39238
O	-6.04637	7.41430	-1.81295
O	-6.16803	5.72524	-3.77845
O	-4.01065	5.92378	-2.44185
C	-3.27696	6.13793	-1.22435
C	-1.79506	6.21588	-1.50803
O	-1.29242	4.94254	-1.95670
C	-1.38758	7.23137	-2.57916
O	-0.12083	7.77010	-2.17936
C	-1.27398	6.37726	-3.83756
C	-0.78480	5.03726	-3.28833
N	-1.22178	3.86661	-4.04687
C	-0.27699	2.89457	-4.43265
O	0.92880	3.11262	-4.25104
N	-0.72788	1.74895	-4.99489
C	-2.03166	1.54861	-5.20206
N	-2.41616	0.37607	-5.71422
C	-2.99764	2.55172	-4.88647
C	-2.54923	3.66972	-4.27999
H	7.65029	-6.25297	-6.57956
H	6.63991	-5.14384	-7.53037
H	7.73723	-4.09701	-5.58999
H	7.13002	-6.24238	-3.99221
H	4.74268	-5.94629	-4.18642
H	5.14463	-2.95678	-3.70424
H	2.88080	-5.64911	-5.19963
H	0.53506	0.35870	-5.05030
H	3.74001	1.14632	-3.94525
H	2.16163	1.76404	-4.33141
H	5.08300	-5.13017	-2.64170
H	9.31421	-1.49272	-1.70088
H	8.17600	-2.14332	-2.89589
H	7.43251	-0.16855	-1.48995
H	8.12055	-1.70468	0.77531
H	6.18321	-3.14379	0.49589
H	4.73705	-0.58942	-0.35126
H	4.61503	-4.40679	-0.59508
H	-0.72270	-1.11438	-2.15729
H	1.32072	1.63917	-1.57965
H	-0.29062	1.09920	-1.90043
H	5.53775	-1.87971	1.59956
H	8.01739	3.74251	0.52059
H	7.18160	2.34842	-0.19651
H	5.79690	4.39039	-0.09090
H	6.63856	4.94647	2.45296
H	5.74869	2.90858	3.41083
H	3.14878	3.36469	1.88341
H	5.55904	0.67842	3.23878
H	-0.32426	-1.24994	1.39713
H	-0.77828	2.12801	0.69737
H	-1.54999	0.56650	0.80774
H	4.45643	4.06757	3.78395
H	2.66118	8.21479	0.63545
H	2.90082	6.46374	0.44350
H	0.59615	7.15618	0.05122
H	0.65770	8.87621	2.18505
H	0.91703	7.23143	3.88733
H	-1.07141	5.32667	2.60081
H	2.43887	5.62844	4.13433
H	-0.51863	-0.05486	4.79435
H	-2.98963	1.39478	2.87253
H	-2.54846	0.02787	3.82940
H	-0.84875	7.35017	3.87506
H	-1.68148	8.64024	2.00381
H	-3.71612	-8.59964	5.75831
H	-2.87957	-7.50403	6.87996
H	-5.03443	-6.64771	5.97951
H	-4.22959	-7.44941	3.43925
H	-2.52099	-5.85745	3.14513
H	-4.02989	-3.63590	4.54669
H	-1.10227	-5.69483	4.91366
H	1.16546	-4.72782	5.31199
H	1.61674	-1.06215	5.55785

H	2.38044	-2.63509	5.62927
H	-3.87013	-4.90553	2.52675
H	-8.08797	-3.86544	2.95630
H	-6.53545	-3.43094	3.70319
H	-7.65102	-1.49983	2.70019
H	-8.47707	-2.99308	0.55002
H	-6.40094	-3.48155	-0.35997
H	-5.21033	-0.73009	0.14693
H	-4.80462	-4.29969	-0.11858
H	-2.62896	-5.41384	0.07353
H	0.28631	-3.59538	1.48816
H	-0.32805	-5.12745	0.90411
H	-6.69754	-1.98793	-1.23361
H	-8.54689	2.45746	0.12229
H	-7.42403	1.31890	0.89577
H	-6.38079	3.47010	0.53672
H	-7.63145	3.76736	-1.90741
H	-6.45376	2.09729	-3.05944
H	-3.81089	2.77581	-1.70788
H	-6.19939	0.20017	-2.61080
H	-5.48180	-2.07093	-3.28471
H	-1.98216	-3.14184	-2.75221
H	-3.59435	-3.62070	-3.23031
H	-5.38793	3.45414	-3.39615
H	-3.59994	7.06979	-0.75475
H	-3.46838	5.31271	-0.53557
H	-1.30767	6.46531	-0.56628
H	-2.12530	8.02904	-2.68331
H	-2.25531	6.28077	-4.29738
H	0.29874	4.98109	-3.26959
H	-3.20980	4.44652	-3.92270
H	-4.04833	2.39416	-5.07108
H	-1.74480	-0.39669	-5.77597
H	-3.39826	0.16806	-5.78531
H	-0.58372	6.79377	-4.56953
H	0.18529	8.37034	-2.86973
H	5.51258	-7.11028	-7.13749
H	-1.42228	-8.35482	5.28406

****

M06-2X/6-31G(d,p)

****

ss-A6 M06-2X/6-31G(d,p)

O	11.21183	-4.69655	-0.47239
C	11.83508	-3.58210	-1.08219
C	10.89481	-2.39239	-1.02349
O	9.71258	-2.65103	-1.78533
C	10.38631	-2.04180	0.37477
O	10.03075	-0.65542	0.34097
C	9.13703	-2.90311	0.49703
C	8.59915	-2.84540	-0.93107
N	7.89142	-4.04956	-1.33621
C	7.64983	-5.20988	-0.63916
N	6.89800	-6.06829	-1.28142
C	6.62692	-5.43750	-2.47880
C	5.81477	-5.78234	-3.57571
N	5.06809	-6.91223	-3.58026
N	5.73534	-4.93590	-4.61054
C	6.38263	-3.76406	-4.52340
N	7.13335	-3.29589	-3.53027
C	7.22524	-4.18269	-2.53104
P	9.69372	0.08651	1.74704
O	10.89990	0.08857	2.65194
O	8.44757	-0.47308	2.39875
O	9.42964	1.58518	1.20108
C	9.24911	1.98763	-0.15569
C	7.78268	2.04464	-0.53610
O	7.29458	0.72181	-0.77392
C	6.88716	2.64833	0.56689
O	6.00635	3.59752	-0.04854
C	6.11492	1.43971	1.08309
C	5.99579	0.63311	-0.19997
N	5.64680	-0.75464	-0.04586
C	5.92466	-1.60096	1.00490
N	5.51700	-2.83098	0.80782
C	4.94456	-2.79938	-0.45105
C	4.27112	-3.76351	-1.22482

N	3.98467	-5.00591	-0.74455
N	3.84194	-3.42306	-2.44533
C	4.00945	-2.15749	-2.85246
N	4.56183	-1.13890	-2.19780
C	5.02160	-1.52282	-1.00286
P	5.27800	4.64706	0.96157
O	6.28964	5.56065	1.60662
O	4.39291	3.92365	1.95305
O	4.40216	5.49669	-0.09598
C	4.09635	5.16001	-1.44810
C	2.73627	4.50034	-1.57158
O	2.84118	3.12258	-1.20574
C	1.66573	5.11438	-0.64501
O	0.45667	5.24164	-1.40493
C	1.51382	4.05780	0.44501
C	1.72543	2.80092	-0.38287
N	2.02170	1.59339	0.34316
C	2.59542	1.43348	1.58391
N	2.76476	0.17911	1.92489
C	2.28136	-0.53418	0.84208
C	2.11684	-1.90838	0.58572
N	2.42133	-2.86867	1.50530
N	1.58144	-2.28496	-0.58112
C	1.19220	-1.33926	-1.44602
N	1.25687	-0.01723	-1.31397
C	1.81531	0.32591	-0.14985
P	-0.71329	6.17870	-0.77459
O	-0.26050	7.61415	-0.68282
O	-1.22354	5.61990	0.53600
O	-1.83881	6.04097	-1.92655
C	-1.85082	5.13661	-3.02980
C	-2.67977	3.90215	-2.73568
O	-1.94481	3.01831	-1.88459
C	-4.00203	4.20116	-1.99763
O	-4.99632	3.32595	-2.55120
C	-3.68071	3.78907	-0.56541
C	-2.83435	2.56376	-0.86884
N	-2.07363	2.00575	0.21353
C	-1.54387	2.61506	1.32564
N	-0.93650	1.78198	2.13761
C	-1.08059	0.54864	1.52625
C	-0.69360	-0.75943	1.87842
N	-0.00405	-1.04367	3.01866
N	-1.05931	-1.77249	1.08255
C	-1.75744	-1.50229	-0.02880
N	-2.15485	-0.32001	-0.48923
C	-1.79100	0.66688	0.33457
P	-6.55665	3.60928	-2.19602
O	-6.99202	4.94494	-2.74211
O	-6.83263	3.43472	-0.71757
O	-7.25241	2.40875	-3.02048
C	-6.62193	1.36227	-3.75664
C	-6.72034	0.03952	-3.02025
O	-5.73750	-0.01798	-1.97871
C	-8.08690	-0.18540	-2.33825
O	-8.43408	-1.55741	-2.56577
C	-7.76228	0.09286	-0.87656
C	-6.36632	-0.50575	-0.79917
N	-5.58098	-0.13007	0.34549
C	-5.40930	1.13357	0.85544
N	-4.65394	1.16680	1.92835
C	-4.32612	-0.15931	2.14603
C	-3.56373	-0.80429	3.13651
N	-2.92297	-0.11048	4.11867
N	-3.50213	-2.14287	3.13190
C	-4.13098	-2.80567	2.15027
N	-4.82716	-2.31517	1.12740
C	-4.90005	-0.98213	1.18072
P	-9.80281	-2.19525	-1.94557
O	-10.51819	-2.94561	-3.03512
O	-10.59158	-1.16718	-1.18438
O	-9.19338	-3.24211	-0.85036
C	-8.42813	-4.34757	-1.33068
C	-8.19768	-5.32586	-0.19676
O	-7.37562	-4.72977	0.80876
C	-9.46875	-5.78703	0.52192
O	-9.20876	-7.10372	0.97785
C	-9.56546	-4.80451	1.68464

C	-8.09235	-4.56098	2.01877
N	-7.82736	-3.23562	2.54277
C	-8.15250	-2.03986	1.94649
N	-7.68602	-0.99060	2.57481
C	-6.99899	-1.52697	3.64821
C	-6.23504	-0.95003	4.68101
N	-6.06335	0.39334	4.79593
N	-5.67427	-1.75663	5.59360
C	-5.83110	-3.08102	5.45917
N	-6.49308	-3.75065	4.51980
C	-7.06290	-2.91947	3.63888
H	12.77322	-3.31510	-0.57584
H	12.06068	-3.78120	-2.13756
H	11.40194	-1.52532	-1.45985
H	11.12825	-2.22172	1.15933
H	9.45165	-3.91377	0.75310
H	7.90394	-2.00673	-1.07183
H	8.04797	-5.36826	0.35213
H	6.27107	-3.10814	-5.38455
H	5.30998	-7.64535	-2.93053
H	4.65161	-7.19033	-4.45655
H	8.43379	-2.51591	1.23219
H	9.67330	2.99237	-0.23333
H	9.78497	1.31871	-0.83254
H	7.67700	2.64738	-1.44756
H	7.47812	3.14631	1.34106
H	6.74143	0.89468	1.79552
H	5.24950	1.06699	-0.87933
H	6.47823	-1.24949	1.86691
H	3.63611	-1.93674	-3.85067
H	4.61752	-5.36131	-0.03915
H	3.73085	-5.68314	-1.45487
H	5.16130	1.71898	1.52696
H	4.08726	6.10332	-2.00066
H	4.86355	4.50484	-1.86684
H	2.39628	4.58212	-2.61186
H	1.96516	6.09497	-0.26397
H	2.33070	4.16424	1.16514
H	0.84750	2.57890	-1.00514
H	2.90247	2.29339	2.16530
H	0.76413	-1.70705	-2.37654
H	3.14360	-2.62308	2.17047
H	2.52380	-3.79732	1.10884
H	0.54375	4.11204	0.93443
H	-2.30414	5.67792	-3.86465
H	-0.83482	4.84225	-3.30143
H	-2.90747	3.39854	-3.68386
H	-4.31837	5.24260	-2.10732
H	-3.06140	4.55520	-0.09011
H	-3.45781	1.74393	-1.25072
H	-1.62399	3.68864	1.45297
H	-2.02517	-2.36678	-0.63250
H	0.56298	-0.28873	3.38381
H	0.46712	-1.94183	2.99863
H	-4.57547	3.58605	0.01894
H	-7.15903	1.27908	-4.70585
H	-5.57519	1.60054	-3.95687
H	-6.53859	-0.77209	-3.73516
H	-8.86664	0.46727	-2.73676
H	-7.69855	1.17680	-0.72690
H	-6.40181	-1.60366	-0.79458
H	-5.88852	1.98670	0.38159
H	-4.05359	-3.89015	2.19611
H	-2.68454	0.84669	3.88515
H	-2.17197	-0.62450	4.56323
H	-8.48383	-0.33828	-0.18653
H	-8.96457	-4.84543	-2.14605
H	-7.46213	-3.98873	-1.70433
H	-7.67636	-6.20098	-0.60086
H	-10.34557	-5.75247	-0.13570
H	-10.03724	-3.87766	1.35234
H	-7.71816	-5.25864	2.77452
H	-8.74831	-2.02931	1.04199
H	-5.34440	-3.68601	6.22128
H	-6.18610	0.92768	3.94401
H	-5.25144	0.65865	5.33919
H	-10.11910	-5.20621	2.53545
H	-9.97619	-7.40510	1.47981

H	11.77932	-5.46587	-0.59443

****

ss-C6 M06-2X/6-31G(d,p)

O	11.95424	-4.90387	-1.34812
C	12.26844	-3.85331	-2.24634
C	11.22266	-2.76278	-2.11013
O	9.96107	-3.24352	-2.57897
C	10.97974	-2.30518	-0.66641
O	10.56431	-0.93373	-0.73609
C	9.81504	-3.18315	-0.23537
C	9.00068	-3.22426	-1.52625
N	8.13463	-4.38176	-1.66116
C	6.78969	-4.19454	-2.07335
O	6.41265	-3.07454	-2.40213
N	5.97483	-5.28715	-2.07401
C	6.46715	-6.48637	-1.81188
N	5.60670	-7.51868	-1.77763
C	7.86304	-6.72665	-1.56835
C	8.65308	-5.63170	-1.50223
P	10.48199	-0.07482	0.64092
O	11.85042	0.10298	1.24718
O	9.46531	-0.65358	1.60226
O	9.97939	1.34163	0.04735
C	9.38295	1.58068	-1.22731
C	7.86837	1.58479	-1.16727
O	7.37218	0.24609	-1.14312
C	7.29106	2.28572	0.08551
O	6.24624	3.16367	-0.35860
C	6.71223	1.12245	0.88290
C	6.24086	0.24410	-0.27069
N	5.86810	-1.11667	0.03487
C	4.66630	-1.63813	-0.51758
O	3.99540	-0.93732	-1.26745
N	4.29803	-2.89806	-0.15025
C	5.09523	-3.63435	0.59663
N	4.65090	-4.86175	0.96444
C	6.36561	-3.17533	1.07849
C	6.69860	-1.89693	0.77580
P	5.69664	4.28029	0.68630
O	6.79814	5.23151	1.08227
O	4.99256	3.64433	1.86654
O	4.62318	5.03871	-0.25127
C	4.20427	4.68555	-1.56901
C	2.83365	4.03701	-1.55171
O	2.94773	2.67335	-1.14556
C	1.85296	4.69946	-0.56063
O	0.57809	4.77647	-1.21338
C	1.80702	3.69155	0.58288
C	1.88751	2.40241	-0.22757
N	2.17441	1.17816	0.48674
C	1.45098	0.00256	0.14128
O	0.63369	0.04738	-0.77162
N	1.68306	-1.12205	0.87653
C	2.61121	-1.12926	1.80946
N	2.74646	-2.25961	2.55273
C	3.42923	0.00848	2.11021
C	3.16654	1.14280	1.41565
P	-0.55161	5.74482	-0.56203
O	-0.09585	7.18232	-0.56940
O	-0.98644	5.25490	0.80322
O	-1.74108	5.54594	-1.63605
C	-1.81284	4.59718	-2.70047
C	-2.70241	3.42250	-2.34014
O	-1.98740	2.50622	-1.51128
C	-3.96268	3.83016	-1.54589
O	-5.06525	3.08401	-2.07970
C	-3.62907	3.36200	-0.13348
C	-2.87477	2.08245	-0.47435
N	-2.10758	1.44837	0.57414
C	-2.12412	0.02807	0.65956
O	-2.78508	-0.61439	-0.14913
N	-1.41586	-0.55386	1.66863
C	-0.68613	0.18044	2.48252
N	-0.03963	-0.45590	3.49430
C	-0.59198	1.60676	2.37914
C	-1.32120	2.19167	1.39714

P	-6.56516	3.56619	-1.68260
O	-6.82969	4.96106	-2.19213
O	-6.83307	3.40167	-0.20116
O	-7.43857	2.50281	-2.52857
C	-6.96976	1.35787	-3.23835
C	-7.18574	0.08679	-2.44201
O	-6.18722	-0.02019	-1.42704
C	-8.54717	0.03205	-1.71157
O	-9.05660	-1.29783	-1.87053
C	-8.15414	0.26222	-0.25777
C	-6.83327	-0.49940	-0.24786
N	-5.95647	-0.31963	0.88669
C	-5.28880	-1.45914	1.41766
O	-5.48726	-2.56030	0.91535
N	-4.47504	-1.26989	2.49430
C	-4.26959	-0.05970	2.97357
N	-3.47466	0.05692	4.06651
C	-4.88042	1.11526	2.42408
C	-5.71045	0.92794	1.36897
P	-10.62452	-1.59660	-1.53321
O	-11.47744	-1.32863	-2.74602
O	-11.05716	-0.91560	-0.26141
O	-10.51009	-3.18261	-1.20977
C	-9.83903	-4.05864	-2.11566
C	-9.22518	-5.18609	-1.31112
O	-8.26268	-4.63677	-0.40965
C	-10.22632	-5.97929	-0.45563
O	-9.79822	-7.33113	-0.48702
C	-10.04163	-5.36625	0.92710
C	-8.54784	-5.04913	0.91987
N	-8.15368	-3.99472	1.84165
C	-7.18422	-4.26742	2.83971
O	-6.69093	-5.38961	2.91000
N	-6.86232	-3.26452	3.70588
C	-7.40863	-2.07136	3.57595
N	-7.04450	-1.11082	4.45615
C	-8.38189	-1.75988	2.56937
C	-8.70365	-2.75691	1.71333
H	13.25666	-3.42621	-2.02847
H	12.26526	-4.20378	-3.28581
H	11.52822	-1.90313	-2.71687
H	11.87124	-2.39577	-0.03758
H	10.19114	-4.17265	0.02826
H	8.35127	-2.35030	-1.61714
H	9.72778	-5.67854	-1.34439
H	8.25990	-7.72420	-1.44077
H	4.63989	-7.35260	-2.01007
H	5.92478	-8.46584	-1.65622
H	9.26795	-2.75452	0.60088
H	12.56329	-5.63424	-1.50594
H	9.72363	2.57252	-1.53653
H	9.72414	0.84273	-1.95674
H	7.48819	2.10200	-2.05883
H	8.04910	2.86097	0.62432
H	7.51966	0.62520	1.42934
H	5.37461	0.68939	-0.76706
H	7.63147	-1.43302	1.09506
H	7.01802	-3.80633	1.66803
H	3.88261	-5.23384	0.42461
H	5.31563	-5.53440	1.31550
H	5.91982	1.44440	1.55680
H	4.15250	5.61743	-2.13899
H	4.92643	4.01491	-2.03910
H	2.40589	4.08948	-2.56143
H	2.17565	5.70052	-0.26061
H	2.70184	3.81293	1.20183
H	0.94965	2.22591	-0.76118
H	3.72918	2.06450	1.56261
H	4.21586	-0.03150	2.85279
H	2.37397	-3.10046	2.13264
H	3.60144	-2.38276	3.07558
H	0.90045	3.79112	1.17741
H	-2.24956	5.12498	-3.55280
H	-0.81654	4.23996	-2.96938
H	-3.01392	2.92015	-3.26539
H	-4.17330	4.90070	-1.62000
H	-2.96038	4.09150	0.33375
H	-3.56473	1.31841	-0.84316

H	-1.30726	3.26581	1.21686
H	0.03664	2.19053	3.03927
H	0.10531	-1.44846	3.36401
H	0.71460	0.03171	3.95603
H	-4.52442	3.20846	0.46581
H	-7.55530	1.30555	-4.16045
H	-5.91238	1.46380	-3.49007
H	-7.11708	-0.77149	-3.12312
H	-9.25689	0.76643	-2.10371
H	-7.98636	1.33044	-0.08814
H	-7.00945	-1.57581	-0.31924
H	-6.20568	1.75161	0.85634
H	-4.67936	2.10377	2.81673
H	-2.87233	-0.73057	4.26379
H	-3.10065	0.96564	4.29662
H	-8.91320	-0.12867	0.41620
H	-10.55261	-4.45871	-2.84410
H	-9.04670	-3.52301	-2.64579
H	-8.73088	-5.88028	-2.00166
H	-11.25109	-5.87714	-0.83253
H	-10.62641	-4.44638	1.00879
H	-7.95009	-5.92025	1.18872
H	-9.40386	-2.62418	0.89135
H	-8.81885	-0.77405	2.48792
H	-6.20843	-1.28292	4.99615
H	-7.26698	-0.14768	4.25608
H	-10.31975	-6.04556	1.73558
H	-10.37437	-7.83909	0.09768

****

ss-G6 M06-2X/6-31G(d,p)

O	10.34908	-5.35523	-0.85550
C	11.25915	-4.29660	-1.08922
C	10.56782	-2.97056	-0.83077
O	9.49969	-2.77460	-1.76069
C	9.91011	-2.83611	0.54511
O	9.82827	-1.42686	0.79601
C	8.52033	-3.40293	0.29775
C	8.24257	-2.85876	-1.09959
N	7.33785	-3.66343	-1.89493
C	7.07612	-5.01799	-1.85421
N	6.20549	-5.40013	-2.74981
C	5.88636	-4.24450	-3.43338
C	4.94507	-4.01060	-4.48992
O	4.17445	-4.78736	-5.03657
N	4.97330	-2.65223	-4.87948
C	5.71589	-1.65696	-4.29533
N	5.58655	-0.40843	-4.82622
N	6.54747	-1.86573	-3.30440
C	6.58626	-3.16311	-2.92161
P	9.40762	-0.87345	2.26380
O	10.53735	-1.03628	3.24801
O	8.10008	-1.46932	2.73587
O	9.21693	0.69293	1.90802
C	9.21537	1.29676	0.61534
C	7.81580	1.68925	0.18143
O	7.10982	0.54320	-0.30579
C	6.95179	2.26503	1.31882
O	6.16958	3.32134	0.74977
C	6.05700	1.08636	1.68327
C	5.81176	0.53007	0.28864
N	5.28318	-0.80638	0.21280
C	5.54085	-1.87449	1.04828
N	4.97631	-2.98351	0.64694
C	4.31637	-2.63423	-0.51242
C	3.47927	-3.41536	-1.37657
O	3.13811	-4.58568	-1.29076
N	3.00997	-2.62062	-2.44762
C	3.22909	-1.27755	-2.61580
N	2.65781	-0.70314	-3.72206
N	3.98127	-0.56235	-1.81991
C	4.49689	-1.28933	-0.79941
P	5.44111	4.37898	1.74551
O	6.45918	5.22427	2.46799
O	4.45808	3.69259	2.66839
O	4.65917	5.27925	0.65342

C	4.53775	5.02736	-0.74594
C	3.18682	4.43161	-1.08952
O	3.15564	3.04483	-0.73791
C	2.01472	5.09089	-0.33409
O	0.93218	5.21656	-1.26511
C	1.67900	4.05289	0.72905
C	1.91430	2.78876	-0.08290
N	2.01371	1.56087	0.65996
C	2.58829	1.35140	1.89803
N	2.54913	0.09867	2.27090
C	1.92009	-0.55218	1.22997
C	1.55026	-1.93023	1.08057
O	1.68691	-2.86738	1.85226
N	0.92345	-2.13285	-0.17114
C	0.61145	-1.16103	-1.08618
N	-0.01689	-1.57982	-2.23089
N	0.93418	0.09836	-0.94287
C	1.58530	0.33803	0.22096
P	-0.28964	6.22016	-0.88784
O	0.17589	7.65436	-0.89539
O	-0.97056	5.81011	0.39921
O	-1.26189	5.95610	-2.15206
C	-1.11777	4.96288	-3.16642
C	-2.04014	3.78394	-2.92638
O	-1.50523	2.94537	-1.89773
C	-3.45227	4.18995	-2.45411
O	-4.37310	3.30513	-3.10198
C	-3.39548	3.88952	-0.96192
C	-2.57372	2.60923	-1.01271
N	-2.03407	2.14834	0.23918
C	-1.58677	2.90154	1.30574
N	-1.16037	2.16997	2.30254
C	-1.33449	0.87080	1.87466
C	-1.10961	-0.36961	2.56039
O	-0.69987	-0.57745	3.69225
N	-1.45782	-1.45673	1.72521
C	-1.99664	-1.37204	0.46792
N	-2.23407	-2.56434	-0.17103
N	-2.21973	-0.24136	-0.15031
C	-1.87377	0.83679	0.59728
P	-5.96697	3.61632	-3.04675
O	-6.29026	4.89278	-3.78165
O	-6.50826	3.57633	-1.63465
O	-6.50665	2.35226	-3.90330
C	-5.75017	1.19614	-4.25359
C	-5.89666	0.08713	-3.23482
O	-5.19836	0.42856	-2.03454
C	-7.36431	-0.22336	-2.82893
O	-7.56722	-1.62674	-3.06425
C	-7.39117	0.21245	-1.36829
C	-5.96131	-0.10066	-0.95906
N	-5.52876	0.46450	0.28920
C	-5.57578	1.78003	0.70075
N	-5.11044	1.94891	1.91263
C	-4.75659	0.68051	2.32678
C	-4.20706	0.21780	3.56815
O	-3.92576	0.83805	4.58383
N	-4.01078	-1.18413	3.52537
C	-4.32104	-2.01591	2.47985
N	-3.99107	-3.33988	2.60596
N	-4.83598	-1.59162	1.35338
C	-5.01759	-0.25153	1.33360
P	-8.41323	-2.64235	-2.12246
O	-8.62919	-3.89985	-2.91881
O	-9.63068	-1.99771	-1.51028
O	-7.39139	-2.91501	-0.88173
C	-6.33959	-3.87085	-0.99936
C	-6.55101	-4.98216	0.01081
O	-6.40301	-4.48697	1.34497
C	-7.94186	-5.61441	-0.01678
O	-7.78485	-6.93960	0.46135
C	-8.73380	-4.74945	0.96967
C	-7.66441	-4.25376	1.95309
N	-7.81154	-2.85344	2.32559
C	-8.37168	-1.79459	1.63225
N	-8.25370	-0.65292	2.25711
C	-7.56948	-0.96127	3.41430
C	-7.14061	-0.11550	4.49075

O	-7.30033	1.08417	4.65260
N	-6.42894	-0.87215	5.45215
C	-6.14774	-2.21058	5.37438
N	-5.28809	-2.71000	6.30764
N	-6.58252	-2.99240	4.41489
C	-7.27691	-2.31429	3.46672
H	12.13873	-4.36831	-0.43426
H	11.60720	-4.29528	-2.12992
H	11.29994	-2.16738	-0.96695
H	10.46817	-3.33795	1.34221
H	8.58216	-4.49026	0.29619
H	7.79551	-1.85843	-1.05653
H	7.57264	-5.66725	-1.14800
H	4.33159	-2.41110	-5.62680
H	5.96579	0.31218	-4.22637
H	4.67633	-0.18004	-5.20570
H	7.79797	-3.06124	1.03421
H	10.77013	-6.18108	-1.11932
H	9.82762	2.19997	0.69220
H	9.65269	0.62663	-0.12704
H	7.89735	2.43489	-0.61918
H	7.55363	2.64430	2.15010
H	6.63063	0.36533	2.27523
H	5.12266	1.17161	-0.27592
H	6.18826	-1.76092	1.90983
H	2.40713	-3.10728	-3.10323
H	2.60525	0.30495	-3.63468
H	1.77312	-1.11028	-4.00462
H	5.15296	1.39742	2.20251
H	4.63268	5.99650	-1.24389
H	5.33440	4.36618	-1.09231
H	3.02143	4.54131	-2.16880
H	2.28075	6.07263	0.06904
H	2.41334	4.11006	1.53953
H	1.11389	2.63896	-0.81872
H	3.04463	2.17129	2.44078
H	0.63115	-3.08861	-0.34892
H	-0.48902	-0.81445	-2.69803
H	-0.61876	-2.38787	-2.11441
H	0.66321	4.15897	1.10452
H	-1.39397	5.44209	-4.10998
H	-0.08332	4.61862	-3.22835
H	-2.12665	3.21304	-3.85970
H	-3.69369	5.22931	-2.69480
H	-2.82815	4.67370	-0.45028
H	-3.16617	1.78004	-1.42037
H	-1.58071	3.98420	1.25222
H	-1.31541	-2.37258	2.14021
H	-2.89351	-2.45118	-0.93223
H	-2.49030	-3.33266	0.44176
H	-4.38489	3.76870	-0.52791
H	-6.15018	0.84419	-5.20860
H	-4.69550	1.44915	-4.38074
H	-5.46791	-0.82987	-3.66107
H	-8.08283	0.32513	-3.44242
H	-7.52120	1.30092	-1.34428
H	-5.80729	-1.18115	-0.86382
H	-5.96862	2.54240	0.03639
H	-3.60259	-1.57479	4.36985
H	-4.62954	-3.92019	2.06058
H	-3.90710	-3.67471	3.55870
H	-8.15097	-0.27760	-0.76190
H	-6.30192	-4.28830	-2.01041
H	-5.39884	-3.36047	-0.76705
H	-5.78726	-5.75151	-0.14043
H	-8.37187	-5.58882	-1.02528
H	-9.21279	-3.92166	0.44669
H	-7.67467	-4.80668	2.89522
H	-8.85478	-1.92900	0.67381
H	-5.98700	-0.31432	6.17504
H	-5.26621	-3.71944	6.35241
H	-5.22869	-2.24857	7.20455
H	-9.50362	-5.32869	1.48310
H	-8.65676	-7.35183	0.49062

****

ss-T6 M06-2X/6-31G(d,p)

C	-8.30232	-3.03551	1.66690
N	-7.53201	-4.18251	1.67968
C	-6.53192	-4.37688	2.62182
N	-6.44104	-3.37104	3.56170
C	-7.17361	-2.19085	3.62631
C	-8.19119	-2.05560	2.58981
C	-7.74328	-5.22076	0.68439
O	-7.45809	-4.70379	-0.60569
C	-8.22199	-5.42115	-1.57931
C	-9.14921	-6.37108	-0.79955
C	-9.17117	-5.74784	0.59164
C	-8.95400	-4.42589	-2.45538
O	-9.86061	-3.69974	-1.62596
P	-10.07583	-2.10152	-1.76345
O	-10.57046	-1.60448	-0.42744
O	-8.51121	-7.63770	-0.79145
O	-5.81021	-5.35790	2.65868
O	-6.94777	-1.36503	4.49918
C	-9.07723	-0.84837	2.64668
O	-8.53049	-1.66203	-2.04163
C	-8.16554	-0.28652	-1.88613
C	-6.78107	-0.09808	-2.55139
O	-5.82838	-0.04399	-1.48528
C	-6.47514	-0.59864	-0.34220
C	-7.87320	-0.00264	-0.41964
C	-6.68515	1.15116	-3.40121
O	-7.29770	2.27002	-2.76035
P	-6.55446	3.41465	-1.89581
O	-6.84936	3.24118	-0.42011
N	-5.70767	-0.31948	0.84997
C	-5.03226	-1.37268	1.45138
N	-4.39065	-1.02991	2.62205
C	-4.26834	0.23259	3.19675
C	-4.92127	1.29765	2.44485
C	-5.59856	0.97223	1.32263
O	-5.02925	-2.51443	1.02414
O	-3.64079	0.38240	4.23475
C	-4.79799	2.69846	2.96259
O	-5.00936	3.05602	-2.24764
C	-3.97725	3.90998	-1.74155
C	-2.66898	3.51335	-2.46199
O	-1.92427	2.72306	-1.53115
C	-2.83086	2.32226	-0.50612
C	-3.67532	3.56979	-0.28667
C	-1.84785	4.70453	-2.91037
O	-1.80908	5.70921	-1.89833
P	-0.59361	6.01334	-0.87804
O	-1.00564	5.65861	0.53578
N	-2.08834	1.82374	0.63030
C	-2.10226	0.45505	0.86394
N	-1.40098	0.07162	1.98740
C	-0.63619	0.86720	2.83688
C	-0.63048	2.28181	2.48639
C	-1.33621	2.68180	1.40637
O	-2.70501	-0.34827	0.17356
O	-0.02581	0.36880	3.77135
C	0.16940	3.21099	3.34756
O	0.51596	4.98305	-1.46694
C	1.81636	4.95293	-0.86674
C	2.73120	4.13457	-1.80733
O	2.86394	2.84283	-1.20938
C	1.81440	2.72427	-0.25150
C	1.78967	4.10135	0.39765
C	4.09721	4.75532	-2.01471
O	4.63800	5.25217	-0.79124
P	5.70702	4.51041	0.16858
O	5.03827	4.06182	1.45149
N	2.06864	1.58702	0.60329
C	1.31383	0.44047	0.39309
N	1.61613	-0.59459	1.25165
C	2.61119	-0.64208	2.22508
C	3.38016	0.58944	2.35392
C	3.08566	1.62270	1.53620
O	0.43286	0.34920	-0.44427
O	2.79737	-1.66065	2.87401
C	4.47508	0.62333	3.37529
O	6.10109	3.25424	-0.78289
C	7.10405	2.34046	-0.32147

C	7.52463	1.47377	-1.53231
O	6.90925	0.19732	-1.34334
C	5.85089	0.39693	-0.40717
C	6.48699	1.31674	0.62626
C	9.02411	1.32091	-1.69377
O	9.70797	1.19837	-0.44632
P	10.12856	-0.17321	0.29632
O	9.08127	-0.60992	1.29893
N	5.35685	-0.88523	0.04134
C	4.13641	-1.31635	-0.46323
N	3.74088	-2.55203	-0.00115
C	4.45971	-3.43691	0.79635
C	5.77095	-2.94617	1.20626
C	6.14874	-1.70998	0.81462
O	3.43887	-0.65329	-1.21160
O	3.99458	-4.52945	1.08490
C	6.62534	-3.86158	2.02886
O	10.17275	-1.16577	-0.99212
C	10.41738	-2.56368	-0.78431
C	10.60402	-3.18710	-2.17448
O	9.28961	-3.54787	-2.61237
C	8.34436	-3.29391	-1.58208
C	9.15082	-3.24147	-0.28996
C	11.50269	-4.40850	-2.19257
O	11.06249	-5.31882	-1.19903
N	7.33325	-4.33869	-1.60619
C	6.03153	-3.99694	-1.92727
N	5.14079	-5.04311	-1.82092
C	5.40825	-6.36752	-1.47822
C	6.82405	-6.66397	-1.27268
C	7.70436	-5.64743	-1.35660
O	5.68252	-2.87659	-2.26180
O	4.50197	-7.18100	-1.39926
C	7.20763	-8.08527	-0.99863
O	11.50069	0.00180	0.89249
O	6.90126	5.40420	0.39025
O	-0.12875	7.43939	-1.03335
O	-6.91983	4.77688	-2.42888
O	-10.93540	-1.73293	-2.94543
H	12.53707	-4.08896	-2.00798
H	11.45195	-4.85627	-3.19266
H	11.01430	-2.43684	-2.85911
H	11.28905	-2.70323	-0.13673
H	9.38897	-4.24457	0.06739
H	7.81773	-2.35220	-1.76059
H	8.77768	-5.79249	-1.25144
H	4.17300	-4.81026	-2.01247
H	8.66034	-2.66620	0.49017
H	11.57468	-6.13153	-1.28064
H	9.41762	2.22852	-2.15898
H	9.24234	0.46610	-2.33789
H	7.12535	1.93789	-2.44402
H	7.94297	2.89574	0.10676
H	7.28212	0.79650	1.16898
H	5.00147	0.89869	-0.88270
H	7.11711	-1.28834	1.08390
H	2.83980	-2.86889	-0.34002
H	5.77720	1.78172	1.30621
H	3.99577	5.61754	-2.67953
H	4.77254	4.02830	-2.46997
H	2.23629	4.04110	-2.78300
H	2.17928	5.97297	-0.71200
H	2.70517	4.27453	0.97224
H	0.85870	2.52376	-0.74671
H	3.65437	2.55180	1.56407
H	1.06394	-1.43465	1.12332
H	0.90081	4.29765	0.99389
H	-2.32995	5.15773	-3.78128
H	-0.83947	4.38729	-3.18219
H	-2.92274	2.90771	-3.34160
H	-4.24686	4.95671	-1.90807
H	-3.07401	4.36910	0.15826
H	-3.45972	1.49302	-0.84691
H	-1.34244	3.72413	1.08615
H	-1.42035	-0.92133	2.18947
H	-4.58411	3.39305	0.28639
H	-7.23887	0.98832	-4.32999
H	-5.64119	1.36485	-3.63861

H	-6.57507	-0.97094	-3.18484
H	-8.92709	0.36391	-2.32686
H	-7.84281	1.07757	-0.24425
H	-6.52335	-1.68873	-0.42144
H	-6.09894	1.72749	0.71566
H	-3.92270	-1.79362	3.09601
H	-8.59990	-0.49099	0.22350
H	-9.51336	-4.95338	-3.23512
H	-8.23285	-3.75128	-2.92223
H	-7.55347	-6.02403	-2.20583
H	-10.14466	-6.42933	-1.25501
H	-9.89011	-4.92518	0.62144
H	-7.04782	-6.02328	0.93694
H	-9.02845	-2.96506	0.85827
H	-5.73207	-3.51013	4.27222
H	-9.40295	-6.46781	1.37916
H	-9.06018	-8.24694	-0.28216
H	8.28697	-8.17761	-0.86658
H	6.71103	-8.44990	-0.09501
H	6.89758	-8.73532	-1.82182
H	7.52338	-3.34888	2.37956
H	6.06905	-4.22956	2.89517
H	6.92849	-4.73711	1.44178
H	4.91490	1.62097	3.43969
H	4.08752	0.34559	4.35942
H	5.26582	-0.09413	3.12542
H	-0.01418	4.25089	3.06871
H	-0.09614	3.07556	4.39953
H	1.24267	3.00620	3.25663
H	-5.45225	3.37418	2.40745
H	-5.06651	2.73870	4.02161
H	-3.76598	3.05953	2.87534
H	-9.83933	-0.89749	1.86573
H	-9.56623	-0.79021	3.62369
H	-8.49493	0.07196	2.52514

****

ss-A2C2A2 M06-2X/6-31G(d,p)

O	10.87859	-5.35159	-1.76882
C	11.07351	-4.30023	-2.69527
C	10.14827	-3.15908	-2.32457
O	8.79008	-3.55358	-2.55496
C	10.23020	-2.76449	-0.84252
O	10.03792	-1.34892	-0.76696
C	9.04420	-3.50254	-0.23509
C	8.03496	-3.38736	-1.36839
N	6.99844	-4.40090	-1.33037
C	6.79008	-5.38213	-0.38875
N	5.71370	-6.09610	-0.59403
C	5.17340	-5.55430	-1.74324
C	3.98478	-5.81457	-2.44856
N	3.08465	-6.73186	-2.01378
N	3.70763	-5.08722	-3.53788
C	4.54811	-4.09583	-3.87000
N	5.67250	-3.71793	-3.26649
C	5.94309	-4.49230	-2.20887
P	10.34251	-0.61277	0.64978
O	11.77805	-0.82768	1.06031
O	9.34093	-0.99848	1.71710
O	10.11750	0.92888	0.21560
C	9.64851	1.39857	-1.04656
C	8.15218	1.63201	-1.02468
O	7.47196	0.37433	-1.04358
C	7.65694	2.37017	0.23830
O	6.67686	3.31887	-0.19668
C	7.00110	1.25357	1.04226
C	6.40689	0.44936	-0.10376
N	5.95193	-0.87534	0.22030
C	6.37000	-1.71620	1.22853
N	5.65841	-2.81289	1.32177
C	4.71587	-2.69165	0.31584
C	3.61752	-3.47848	-0.07785
N	3.24555	-4.60133	0.60331
N	2.87266	-3.07692	-1.11398
C	3.16607	-1.90801	-1.69955
N	4.13487	-1.04877	-1.39347

C	4.88178	-1.49754	-0.38057
P	6.09367	4.40614	0.86071
O	7.19166	5.30066	1.37694
O	5.28100	3.74962	1.95691
O	5.14704	5.25279	-0.13994
C	4.64997	4.81433	-1.40370
C	3.31404	4.11013	-1.29441
O	3.48922	2.77596	-0.81718
C	2.31629	4.79315	-0.32712
O	1.08108	4.94534	-1.03759
C	2.17477	3.76838	0.79417
C	2.34225	2.48979	-0.01702
N	2.54935	1.26092	0.71469
C	1.77795	0.12074	0.35727
O	1.00788	0.18880	-0.59493
N	1.93010	-1.00379	1.11191
C	2.80748	-1.03914	2.09681
N	2.87106	-2.17589	2.83538
C	3.65267	0.07021	2.43233
C	3.47809	1.20348	1.70845
P	-0.05646	5.91272	-0.40000
O	0.45253	7.32508	-0.26009
O	-0.61820	5.34317	0.88573
O	-1.16134	5.84551	-1.57660
C	-1.18298	4.96346	-2.69894
C	-2.11560	3.78989	-2.47405
O	-1.48553	2.81148	-1.64382
C	-3.43300	4.17334	-1.76333
O	-4.48670	3.45710	-2.42203
C	-3.22103	3.62505	-0.35812
C	-2.47096	2.34929	-0.71738
N	-1.82576	1.61708	0.34914
C	-1.92613	0.19949	0.34948
O	-2.53018	-0.36283	-0.55799
N	-1.36763	-0.47526	1.39530
C	-0.69205	0.17109	2.32131
N	-0.22632	-0.54917	3.37933
C	-0.48644	1.58947	2.29485
C	-1.08101	2.26829	1.28179
P	-6.02909	3.94081	-2.21616
O	-6.44431	4.85926	-3.33531
O	-6.26477	4.44642	-0.81786
O	-6.76673	2.49907	-2.32782
C	-6.59879	1.68873	-3.48857
C	-6.95851	0.25908	-3.12778
O	-6.03469	-0.23291	-2.15192
C	-8.34915	0.08845	-2.50308
O	-8.78734	-1.22855	-2.86311
C	-8.04918	0.20857	-1.01819
C	-6.69505	-0.48660	-0.91992
N	-5.87884	-0.02062	0.17842
C	-5.64823	1.27703	0.56795
N	-4.91292	1.37787	1.64902
C	-4.64365	0.06673	1.99232
C	-3.91793	-0.51348	3.04667
N	-3.32190	0.24278	4.01101
N	-3.86136	-1.84868	3.13257
C	-4.48448	-2.57290	2.19179
N	-5.17931	-2.14975	1.13897
C	-5.22816	-0.81603	1.08954
P	-10.13441	-1.87831	-2.20715
O	-10.83252	-2.67351	-3.27441
O	-10.93959	-0.84260	-1.47416
O	-9.50673	-2.87951	-1.07711
C	-8.76116	-4.01264	-1.52477
C	-8.60381	-5.00011	-0.38560
O	-7.80986	-4.43516	0.65866
C	-9.91417	-5.43826	0.27530
O	-9.70786	-6.76946	0.71638
C	-10.03176	-4.47339	1.45094
C	-8.56734	-4.26172	1.84284
N	-8.29868	-2.94349	2.38416
C	-8.59894	-1.74696	1.77785
N	-8.11123	-0.70087	2.39424
C	-7.43666	-1.24117	3.47314
C	-6.66125	-0.66651	4.49745
N	-6.46688	0.67570	4.59187
N	-6.11548	-1.47161	5.41997

C	-6.29872	-2.79413	5.30090
N	-6.97375	-3.46279	4.36929
C	-7.52840	-2.63219	3.47841
H	12.10772	-3.93040	-2.67472
H	10.84266	-4.61807	-3.72034
H	10.38386	-2.28722	-2.94532
H	11.18858	-3.03645	-0.38977
H	9.33225	-4.54824	-0.10720
H	7.54070	-2.40522	-1.37588
H	7.46399	-5.51403	0.44543
H	4.26557	-3.52318	-4.75067
H	3.40834	-7.44270	-1.37402
H	2.35501	-6.99799	-2.65901
H	8.70849	-3.06351	0.70424
H	10.15526	2.34978	-1.23088
H	9.90341	0.69488	-1.84180
H	7.87326	2.21990	-1.90853
H	8.46396	2.88473	0.76773
H	7.77469	0.66051	1.53898
H	5.54527	0.96907	-0.54566
H	7.23849	-1.46708	1.82684
H	2.51967	-1.62380	-2.52634
H	3.99664	-5.07687	1.08887
H	2.64256	-5.22270	0.07437
H	6.26711	1.62901	1.75369
H	4.51690	5.71953	-2.00225
H	5.37531	4.16329	-1.89670
H	2.85587	4.08488	-2.29292
H	2.66771	5.77212	0.01054
H	3.01015	3.88073	1.49153
H	1.46744	2.31692	-0.65031
H	4.06427	2.10886	1.86511
H	4.38989	0.01066	3.22263
H	2.46939	-2.99965	2.40115
H	3.71977	-2.34240	3.35774
H	1.21696	3.85195	1.30582
H	-1.55304	5.55237	-3.54300
H	-0.17793	4.60235	-2.92757
H	-2.35654	3.34510	-3.44836
H	-3.62073	5.25012	-1.79406
H	-2.57210	4.30682	0.20088
H	-3.14211	1.63460	-1.20127
H	-0.99281	3.34794	1.16319
H	0.10120	2.09829	3.04828
H	-0.14706	-1.54406	3.21606
H	0.52243	-0.14781	3.92588
H	-4.15784	3.45985	0.16928
H	-7.24414	2.05152	-4.29632
H	-5.55565	1.71594	-3.82004
H	-6.88354	-0.35710	-4.02999
H	-9.07024	0.82906	-2.85940
H	-7.95499	1.26389	-0.75991
H	-6.80391	-1.56724	-0.76545
H	-6.04923	2.11664	0.00917
H	-4.41092	-3.65212	2.31065
H	-3.13180	1.20232	3.75084
H	-2.55956	-0.21516	4.49640
H	-8.81567	-0.23950	-0.39293
H	-9.28314	-4.49605	-2.35786
H	-7.77161	-3.68600	-1.86542
H	-8.08824	-5.88520	-0.77550
H	-10.76355	-5.36827	-0.41506
H	-10.47655	-3.53370	1.11759
H	-8.23360	-4.97123	2.60648
H	-9.19364	-1.73503	0.87357
H	-5.82361	-3.39961	6.06988
H	-6.59978	1.19868	3.73494
H	-5.64385	0.93943	5.11925
H	-10.62379	-4.87733	2.27427
H	-10.50063	-7.05713	1.18585
H	11.47629	-6.07238	-1.99672

****

ds-A4 M06-2X/6-31G(d,p)

O	5.91969	7.85260	4.26297
C	6.96658	7.13651	4.89501
C	7.13148	5.79118	4.21336

O	5.97025	4.98715	4.43873
C	7.28006	5.86644	2.68865
O	8.02363	4.70167	2.30267
C	5.84138	5.73290	2.21642
C	5.34029	4.68104	3.19719
N	3.90274	4.65188	3.40492
C	3.26740	3.41746	3.38072
O	3.85326	2.36939	3.14336
N	1.92268	3.45703	3.63952
C	1.17543	4.55756	4.00720
O	-0.01554	4.43378	4.29100
C	1.90889	5.82008	4.06371
C	1.16625	7.05260	4.47933
C	3.23023	5.79502	3.79008
P	8.53796	4.51930	0.77117
O	9.69677	5.43722	0.47757
O	7.40356	4.63662	-0.22443
O	9.08091	2.99942	0.86017
C	8.76583	2.02695	1.85560
C	7.71032	1.04773	1.38545
O	6.41182	1.63450	1.48130
C	7.87294	0.60244	-0.08570
O	7.66172	-0.81512	-0.11259
C	6.71672	1.31416	-0.77873
C	5.67994	1.20503	0.33118
N	4.47120	1.98838	0.21772
C	3.26151	1.36607	0.52285
O	3.15357	0.16152	0.70051
N	2.17690	2.20581	0.57873
C	2.18880	3.57483	0.43486
O	1.16522	4.24316	0.61666
C	3.46765	4.16030	0.06415
C	3.50874	5.63654	-0.18678
C	4.54469	3.34648	0.00388
P	8.03037	-1.65860	-1.45007
O	9.51602	-1.64012	-1.70516
O	7.20565	-1.22141	-2.64343
O	7.57459	-3.13161	-0.96448
C	6.84722	-3.46819	0.21736
C	5.39861	-3.78463	-0.09579
O	4.66005	-2.57825	-0.30504
C	5.22059	-4.62547	-1.37676
O	4.22122	-5.61126	-1.09593
C	4.69208	-3.59967	-2.37195
C	3.80674	-2.79281	-1.42942
N	3.30785	-1.51345	-1.89111
C	1.96003	-1.22924	-1.70009
O	1.15092	-2.05427	-1.30156
N	1.58759	0.05793	-2.01713
C	2.41618	1.06107	-2.47749
O	1.99392	2.21204	-2.62826
C	3.78503	0.66778	-2.76177
C	4.67996	1.67939	-3.40920
C	4.17279	-0.57911	-2.41418
P	4.00467	-6.84232	-2.13781
O	5.27267	-7.64109	-2.29576
O	3.39273	-6.38579	-3.44450
O	2.97036	-7.71650	-1.25452
C	1.86963	-7.16640	-0.53381
C	0.73167	-6.65631	-1.39852
O	0.98194	-5.33266	-1.84657
C	0.39939	-7.49190	-2.65331
O	-0.72403	-8.29053	-2.32983
C	0.12973	-6.42199	-3.72473
C	0.06582	-5.12753	-2.90936
N	0.44509	-3.92278	-3.62281
C	-0.45586	-2.87424	-3.70520
O	-1.61504	-2.95070	-3.32430
N	0.05156	-1.73034	-4.27920
C	1.33135	-1.54617	-4.76903
O	1.68371	-0.45079	-5.21189
C	2.19667	-2.71306	-4.72537
C	3.56170	-2.59431	-5.33267
C	1.71991	-3.83030	-4.13420
O	-6.45453	6.32713	-5.85583
C	-7.45158	5.37850	-6.18318
C	-7.49795	4.32325	-5.09647
O	-6.29727	3.54241	-5.12673

C	-7.58399	4.89744	-3.67458
O	-8.33949	3.95873	-2.90336
C	-6.12666	4.89960	-3.23415
C	-5.66393	3.59104	-3.85522
N	-4.23294	3.49472	-4.04037
C	-3.26593	4.46404	-3.88964
N	-2.05246	4.03905	-4.12215
C	-2.22427	2.70970	-4.44702
C	-1.30528	1.67735	-4.72823
N	0.01673	1.87893	-4.76813
N	-1.79910	0.44598	-4.97107
C	-3.12776	0.25241	-4.91088
N	-4.08143	1.13192	-4.63691
C	-3.56536	2.34928	-4.39975
P	-8.77119	4.32986	-1.38151
O	-9.78569	5.44515	-1.36785
O	-7.57028	4.59043	-0.49874
O	-9.49253	2.94069	-0.97262
C	-9.42675	1.70628	-1.68665
C	-8.35272	0.78755	-1.14050
O	-7.05959	1.22351	-1.57428
C	-8.29798	0.75091	0.40168
O	-8.09720	-0.61757	0.77873
C	-7.05518	1.57792	0.70627
C	-6.18106	1.12957	-0.45507
N	-4.98724	1.89352	-0.69606
C	-4.74152	3.22754	-0.44633
N	-3.49981	3.57761	-0.67383
C	-2.88793	2.40981	-1.09366
C	-1.55297	2.08930	-1.43752
N	-0.56158	2.98144	-1.43370
N	-1.27787	0.81208	-1.77700
C	-2.26383	-0.09903	-1.75716
N	-3.54084	0.07893	-1.43934
C	-3.79199	1.35526	-1.11445
P	-8.32484	-1.04262	2.33004
O	-9.77110	-0.88394	2.72399
O	-7.36038	-0.32977	3.25357
O	-7.94710	-2.61067	2.24134
C	-7.41769	-3.32341	1.12527
C	-5.93942	-3.60614	1.30589
O	-5.17641	-2.43200	1.00672
C	-5.56287	-4.01240	2.74768
O	-4.63140	-5.09399	2.63554
C	-4.89435	-2.75033	3.27490
C	-4.18045	-2.28520	2.01367
N	-3.71202	-0.92620	2.02572
C	-4.39505	0.17745	2.47995
N	-3.69833	1.28635	2.40803
C	-2.48398	0.88257	1.88856
C	-1.29682	1.58861	1.60562
N	-1.17448	2.90885	1.79216
N	-0.24163	0.87987	1.16521
C	-0.36041	-0.44656	0.98786
N	-1.43171	-1.20585	1.18683
C	-2.46623	-0.48587	1.64604
P	-4.11357	-5.89478	3.96030
O	-4.60417	-7.31689	3.91296
O	-4.39970	-5.11950	5.21641
O	-2.50584	-5.83566	3.70554
C	-1.99511	-6.38711	2.49269
C	-0.51775	-6.07132	2.40094
O	-0.33289	-4.65694	2.31726
C	0.32556	-6.53815	3.59019
O	1.61080	-6.82658	3.06462
C	0.34858	-5.30045	4.48017
C	0.37683	-4.17541	3.44564
N	-0.23627	-2.94346	3.90198
C	-1.53367	-2.75797	4.32067
N	-1.82643	-1.50918	4.58629
C	-0.65361	-0.82673	4.32198
C	-0.30152	0.53917	4.36554
N	-1.13962	1.52077	4.75861
N	0.95363	0.86652	4.01223
C	1.80933	-0.08266	3.59910
N	1.58807	-1.38684	3.50850
C	0.34111	-1.70033	3.88340
H	7.91743	7.68278	4.83200

H	6.74211	6.95636	5.95370
H	8.00527	5.28723	4.64152
H	7.79013	6.77483	2.35229
H	5.32458	6.68532	2.34343
H	5.61709	3.67737	2.86278
H	3.86114	6.67619	3.88495
H	1.47003	2.51349	3.71314
H	5.79038	5.40069	1.18438
H	9.69188	1.47785	2.04840
H	8.43903	2.50948	2.77944
H	7.75790	0.15582	2.02432
H	8.85933	0.84228	-0.49266
H	6.98077	2.36164	-0.95833
H	5.35260	0.16594	0.43773
H	5.54398	3.72671	-0.20859
H	1.26986	1.72557	0.80677
H	6.43750	0.80849	-1.69906
H	7.32120	-4.36421	0.62849
H	6.90460	-2.66332	0.95251
H	4.96963	-4.33562	0.75124
H	6.14649	-5.11510	-1.69128
H	5.52248	-2.98752	-2.73972
H	2.92486	-3.37775	-1.15219
H	5.20364	-0.91490	-2.53325
H	0.57952	0.27991	-1.89356
H	4.15716	-4.07913	-3.18761
H	1.49205	-7.98852	0.08001
H	2.21138	-6.36629	0.13147
H	-0.18234	-6.65540	-0.78124
H	1.25500	-8.11237	-2.93562
H	0.96564	-6.37579	-4.42552
H	-0.94012	-4.95093	-2.51698
H	2.31622	-4.73464	-4.00742
H	-0.63047	-0.96620	-4.44171
H	-0.79719	-6.61432	-4.26830
H	-0.85151	-8.92898	-3.04133
H	-8.44333	5.84577	-6.25533
H	-7.23690	4.88187	-7.13856
H	-8.35915	3.66901	-5.27449
H	-8.05823	5.88347	-3.64325
H	-5.63246	5.74235	-3.72037
H	-5.97273	2.72798	-3.25122
H	-3.51891	5.47038	-3.58921
H	-3.45134	-0.76934	-5.09885
H	0.39327	2.70333	-4.32391
H	0.63380	1.07462	-4.88116
H	-6.01064	4.93913	-2.15268
H	-10.39974	1.22570	-1.55338
H	-9.25718	1.88410	-2.75029
H	-8.54169	-0.22767	-1.51241
H	-9.21424	1.13401	0.86063
H	-7.28952	2.64077	0.59000
H	-5.85059	0.09105	-0.32015
H	-5.54523	3.88114	-0.12745
H	-1.96855	-1.10775	-2.03919
H	-0.72761	3.91985	-1.10799
H	0.37178	2.71342	-1.74153
H	-6.64320	1.36645	1.69044
H	-7.95613	-4.27440	1.07879
H	-7.58104	-2.77325	0.19651
H	-5.65005	-4.41320	0.62156
H	-6.43336	-4.31835	3.33286
H	-5.66874	-2.02077	3.53860
H	-3.30374	-2.90873	1.79086
H	-5.41086	0.08052	2.85295
H	0.53784	-0.94587	0.63468
H	-2.00477	3.44074	2.00302
H	-0.35089	3.39726	1.43861
H	-4.24409	-2.93543	4.12714
H	-2.15125	-7.47177	2.47631
H	-2.50987	-5.93667	1.63676
H	-0.12470	-6.53456	1.48892
H	-0.11316	-7.41211	4.08628
H	-0.56959	-5.24647	5.06896
H	1.39818	-3.90546	3.15984
H	-2.20492	-3.60398	4.41917
H	2.78424	0.28503	3.29113
H	-2.12852	1.31254	4.75694

H	-0.86652	2.47783	4.53636
H	1.21113	-5.26901	5.14860
H	2.19777	-7.04313	3.79984
H	1.83435	7.91477	4.51857
H	0.35673	7.27105	3.77671
H	0.70991	6.91642	5.46391
H	4.49049	5.94504	-0.55177
H	2.75516	5.91689	-0.92823
H	3.28189	6.19383	0.72965
H	5.63487	1.22940	-3.68894
H	4.20298	2.07668	-4.31002
H	4.87118	2.52753	-2.74176
H	4.04915	-3.56950	-5.39150
H	3.48892	-2.17870	-6.34122
H	4.19824	-1.91863	-4.74944
H	5.76360	8.66492	4.75794
H	-6.43571	6.99688	-6.54847

****

ds-G4 M06-2X/6-31G(d,p)

O	4.87120	-6.94170	-5.58613
C	6.09158	-6.34958	-5.99283
C	6.46386	-5.23952	-5.02672
O	5.49636	-4.19007	-5.07461
C	6.51598	-5.65441	-3.55443
O	7.39084	-4.71397	-2.91647
C	5.08250	-5.42338	-3.10141
C	4.75171	-4.14117	-3.86020
N	3.35172	-3.95857	-4.17101
C	2.38544	-4.89891	-4.47288
N	1.23026	-4.37113	-4.77406
C	1.44300	-3.00783	-4.68857
C	0.55368	-1.90256	-4.86554
O	-0.65431	-1.93572	-5.12556
N	1.21333	-0.68026	-4.71356
C	2.54175	-0.52800	-4.40159
N	3.01200	0.73837	-4.37150
N	3.36111	-1.53967	-4.18825
C	2.75626	-2.73563	-4.33304
P	7.90598	-4.97419	-1.39827
O	8.93605	-6.07473	-1.35674
O	6.75314	-5.18794	-0.44317
O	8.62804	-3.55384	-1.11840
C	8.54140	-2.37078	-1.91239
C	7.59884	-1.34892	-1.30752
O	6.23975	-1.70681	-1.56699
C	7.71678	-1.22314	0.22424
O	7.58199	0.16817	0.53196
C	6.48749	-1.97826	0.71616
C	5.49712	-1.54105	-0.35538
N	4.26943	-2.27681	-0.46796
C	4.07490	-3.64092	-0.36123
N	2.86505	-4.01114	-0.68690
C	2.22159	-2.83435	-1.02301
C	0.88668	-2.58699	-1.47416
O	-0.01410	-3.40542	-1.69396
N	0.66327	-1.22599	-1.67854
C	1.59415	-0.22923	-1.52888
N	1.18525	1.00887	-1.86183
N	2.83786	-0.44453	-1.14258
C	3.07900	-1.74809	-0.90110
P	8.21664	0.75202	1.90596
O	9.71844	0.60438	1.89320
O	7.55633	0.16929	3.13461
O	7.77591	2.30187	1.75975
C	7.20831	2.91666	0.60396
C	5.76234	3.28703	0.87095
O	4.95461	2.10959	0.91526
C	5.55753	3.99663	2.22438
O	4.57587	5.02077	2.02865
C	4.97992	2.89331	3.10165
C	4.12093	2.16991	2.07380
N	3.70345	0.83566	2.41047
C	4.47257	-0.18029	2.94863
N	3.85569	-1.33221	2.96233
C	2.61912	-1.07221	2.40190
C	1.52436	-1.94201	2.10369

O	1.43667	-3.16391	2.28130
N	0.46443	-1.23908	1.53606
C	0.46121	0.09748	1.22532
N	-0.63612	0.54443	0.60200
N	1.47783	0.90852	1.46392
C	2.50864	0.26730	2.04569
P	4.72025	6.40405	2.87642
O	5.87458	7.22054	2.35609
O	4.71557	6.14092	4.35881
O	3.28468	7.06114	2.49518
C	2.97557	7.30977	1.12285
C	1.48201	7.18444	0.89694
O	1.05378	5.83553	1.12898
C	0.59394	8.05266	1.79197
O	-0.57045	8.33974	1.02974
C	0.28010	7.12032	2.95448
C	0.20353	5.75563	2.26495
N	0.61993	4.65931	3.11705
C	1.78835	4.59056	3.85502
N	1.94156	3.44857	4.46649
C	0.82064	2.72458	4.12028
C	0.46588	1.38234	4.43451
O	1.12884	0.57317	5.09338
N	-0.77609	1.03432	3.90290
C	-1.55242	1.85157	3.11605
N	-2.75192	1.35482	2.74621
N	-1.19667	3.07299	2.75187
C	-0.01180	3.44975	3.28099
O	-3.58897	-7.23998	4.92592
C	-4.70328	-6.94265	5.75143
C	-5.42819	-5.74481	5.16887
O	-4.64587	-4.56163	5.34964
C	-5.67911	-5.86976	3.66010
O	-6.94029	-5.24890	3.38388
C	-4.54085	-5.06760	3.05319
C	-4.29342	-3.98570	4.09681
N	-2.90333	-3.54222	4.17842
C	-2.57925	-2.18269	4.02573
O	-3.44259	-1.38167	3.65640
N	-1.30859	-1.79467	4.31425
C	-0.38549	-2.68087	4.68968
N	0.80362	-2.22098	5.07886
C	-0.66931	-4.08689	4.74611
C	-1.94870	-4.45626	4.52430
P	-7.64159	-5.48693	1.93427
O	-8.60318	-6.64777	1.97292
O	-6.60484	-5.58854	0.83910
O	-8.51890	-4.12741	1.83286
C	-8.24872	-2.91732	2.53770
C	-7.37809	-1.96251	1.75174
O	-6.02649	-2.42222	1.73147
C	-7.78837	-1.78529	0.27225
O	-7.70561	-0.38105	0.01063
C	-6.67936	-2.52149	-0.47464
C	-5.51716	-2.12818	0.42618
N	-4.23563	-2.77879	0.26606
C	-3.08415	-2.03307	0.61559
O	-3.16399	-0.80248	0.68800
N	-1.93671	-2.70643	0.86107
C	-1.85155	-4.02014	0.63691
N	-0.75300	-4.65094	1.04441
C	-2.93257	-4.74287	0.02621
C	-4.11780	-4.09662	-0.07086
P	-8.33045	0.27663	-1.33737
O	-9.81696	0.47272	-1.19214
O	-7.88595	-0.44956	-2.58111
O	-7.51684	1.68297	-1.29498
C	-7.35901	2.42423	-0.08546
C	-6.09583	3.25520	-0.19323
O	-4.95270	2.39674	-0.26879
C	-6.04178	4.15121	-1.43600
O	-5.38304	5.35484	-1.03309
C	-5.21390	3.32784	-2.41305
C	-4.24383	2.59053	-1.49107
N	-3.80185	1.28254	-1.96976
C	-2.42970	0.96156	-1.96405
O	-1.60376	1.85980	-1.75662
N	-2.06488	-0.32497	-2.19414

C	-2.98031	-1.25468	-2.47024
N	-2.58798	-2.52232	-2.57818
C	-4.36647	-0.91961	-2.62964
C	-4.72884	0.34344	-2.31545
P	-5.05053	6.53005	-2.11849
O	-5.33361	7.85641	-1.46840
O	-5.71141	6.25364	-3.43949
O	-3.44261	6.32815	-2.31488
C	-2.63273	6.50974	-1.14851
C	-1.17735	6.34931	-1.51894
O	-0.95268	5.01522	-1.97676
C	-0.69640	7.28012	-2.64180
O	0.64565	7.62605	-2.33590
C	-0.76345	6.38312	-3.87365
C	-0.38455	5.03029	-3.27487
N	-0.89240	3.87168	-3.99685
C	-0.02595	2.80737	-4.29668
O	1.19040	2.96311	-4.14285
N	-0.56324	1.64824	-4.75689
C	-1.87825	1.53486	-4.94302
N	-2.35581	0.35952	-5.35952
C	-2.77509	2.63005	-4.70704
C	-2.23501	3.75181	-4.18361
H	6.90770	-7.08533	-6.01468
H	6.00261	-5.90760	-6.99332
H	7.43615	-4.83378	-5.32759
H	6.87949	-6.67670	-3.40679
H	4.46664	-6.25570	-3.43974
H	5.05126	-3.25648	-3.28677
H	2.61358	-5.95532	-4.47160
H	0.61502	0.16630	-4.77108
H	3.89842	0.83287	-3.89730
H	2.34417	1.50518	-4.24957
H	5.00436	-5.30454	-2.02441
H	9.54843	-1.94432	-1.94046
H	8.22515	-2.60828	-2.93009
H	7.81391	-0.37235	-1.76006
H	8.66706	-1.60863	0.60468
H	6.66104	-3.05699	0.63641
H	5.21623	-0.48905	-0.21759
H	4.89269	-4.29093	-0.07299
H	-0.30055	-0.93535	-1.92983
H	1.82044	1.75512	-1.62332
H	0.18795	1.23574	-1.88339
H	6.20157	-1.69111	1.72685
H	7.77871	3.82918	0.40453
H	7.27242	2.25620	-0.26255
H	5.41464	3.94759	0.06654
H	6.49140	4.42003	2.60723
H	5.78745	2.23300	3.43583
H	3.20434	2.73388	1.86041
H	5.49393	-0.00160	3.26930
H	-0.39010	-1.78576	1.31524
H	-0.67618	1.52315	0.36040
H	-1.50408	0.00439	0.58181
H	4.40251	3.28138	3.94083
H	3.30962	8.31652	0.84854
H	3.48276	6.57984	0.48396
H	1.28516	7.43538	-0.15181
H	1.10015	8.97239	2.10840
H	1.10112	7.13765	3.67360
H	-0.81334	5.51165	1.94061
H	2.47830	5.41973	3.90758
H	-1.06223	0.04625	4.06359
H	-3.20308	1.82029	1.97087
H	-2.93608	0.35472	2.84391
H	-0.64958	7.37323	3.46830
H	-1.20317	8.79117	1.60228
H	-5.40019	-7.79022	5.79206
H	-4.39058	-6.70061	6.77463
H	-6.38307	-5.61986	5.69120
H	-5.69132	-6.91500	3.33571
H	-3.66001	-5.70193	2.92585
H	-4.88590	-3.09251	3.89093
H	-2.30209	-5.47851	4.63459
H	0.08902	-4.80451	5.02756
H	0.99017	-1.21585	5.02173
H	1.57313	-2.86373	5.17792

H	-4.83926	-4.64486	2.10058
H	-9.21921	-2.44271	2.70790
H	-7.78240	-3.13113	3.50214
H	-7.42733	-0.97947	2.23944
H	-8.79558	-2.15873	0.06388
H	-6.85999	-3.59854	-0.41319
H	-5.31773	-1.05734	0.33629
H	-5.03471	-4.60027	-0.35572
H	-2.84400	-5.79150	-0.22299
H	0.01704	-4.12416	1.46842
H	-0.61295	-5.62078	0.81082
H	-6.60092	-2.17662	-1.50337
H	-8.22387	3.08022	0.06493
H	-7.26882	1.74416	0.76518
H	-6.01626	3.87798	0.70430
H	-7.03506	4.38861	-1.82696
H	-5.87326	2.63060	-2.93438
H	-3.33180	3.16805	-1.32079
H	-5.76705	0.65609	-2.32627
H	-5.10086	-1.65227	-2.93428
H	-1.62254	-2.80300	-2.37723
H	-3.26627	-3.23992	-2.77927
H	-4.72035	3.96071	-3.14737
H	-2.79777	7.50738	-0.72724
H	-2.90269	5.75633	-0.39866
H	-0.58401	6.53045	-0.61772
H	-1.32740	8.17247	-2.72879
H	-1.78240	6.36952	-4.26694
H	0.69737	4.90477	-3.22467
H	-2.82701	4.60785	-3.87169
H	-3.84000	2.53046	-4.87060
H	-1.74429	-0.46256	-5.34140
H	-3.35275	0.22228	-5.42005
H	-0.07791	6.69403	-4.66460
H	0.98993	8.17233	-3.05329
H	4.60581	-7.57834	-6.25935
H	-3.09789	-7.96848	5.32338

****
M06-2X/TZVP
****

ss-A6 M06-2X/TZVP

O	11.30706	-5.06718	-1.36046
C	11.90219	-3.77829	-1.36253
C	10.85354	-2.75104	-1.00031
O	9.88393	-2.64270	-2.05054
C	10.05523	-3.09429	0.26259
O	9.69863	-1.85895	0.88826
C	8.80364	-3.75298	-0.29815
C	8.58344	-2.88899	-1.52862
N	7.77325	-3.47426	-2.57008
C	7.46266	-4.79110	-2.81695
N	6.77101	-4.96735	-3.90900
C	6.62546	-3.69726	-4.42729
C	5.97258	-3.19854	-5.56872
N	5.28733	-3.99447	-6.40642
N	6.02900	-1.88304	-5.81039
C	6.67054	-1.09568	-4.93984
N	7.30054	-1.44048	-3.82543
C	7.24295	-2.75826	-3.61564
P	9.46558	-1.80952	2.50353
O	10.79488	-1.87013	3.22323
O	8.42626	-2.81374	2.94879
O	8.78634	-0.33834	2.59093
C	9.46096	0.80967	2.07828
C	8.44064	1.77477	1.51001
O	7.77327	1.19393	0.38381
C	7.32405	2.17790	2.47619
O	6.93810	3.50453	2.09627
C	6.25485	1.13626	2.18629
C	6.40662	0.93997	0.68486
N	6.02482	-0.37416	0.21705
C	6.17582	-1.59729	0.82583
N	5.66209	-2.58435	0.13937
C	5.14050	-1.98325	-0.98735
C	4.43535	-2.47989	-2.09543
N	4.13927	-3.78974	-2.23215

N	4.00541	-1.61225	-3.01769
C	4.26948	-0.31121	-2.85018
N	4.93527	0.27222	-1.86272
C	5.34955	-0.61227	-0.95486
P	5.54060	4.20196	2.57411
O	5.78680	5.68620	2.68543
O	4.94778	3.49760	3.77000
O	4.55923	3.88755	1.30188
C	4.76541	4.59567	0.07762
C	3.47243	4.66512	-0.70827
O	3.08515	3.37789	-1.19972
C	2.27251	5.16267	0.09674
O	1.40620	5.81771	-0.83625
C	1.68296	3.86875	0.63501
C	1.94638	2.88870	-0.50490
N	2.18193	1.52920	-0.06872
C	2.89067	1.10159	1.02523
N	2.93636	-0.19798	1.14729
C	2.21728	-0.66173	0.06411
C	1.87739	-1.95109	-0.37083
N	2.25858	-3.06302	0.30549
N	1.11892	-2.07637	-1.46402
C	0.73137	-0.97020	-2.10793
N	1.00405	0.29387	-1.80848
C	1.74419	0.39470	-0.70391
P	-0.12122	6.24935	-0.44110
O	-0.45473	7.49758	-1.21999
O	-0.30660	6.28680	1.05606
O	-0.98820	4.98018	-1.00553
C	-1.12804	4.83614	-2.42083
C	-2.26415	3.88961	-2.74381
O	-1.94093	2.54926	-2.35735
C	-3.58557	4.20738	-2.03983
O	-4.62423	3.77769	-2.92759
C	-3.49253	3.35420	-0.78563
C	-2.78652	2.10861	-1.30420
N	-2.00089	1.41866	-0.30734
C	-1.23228	1.96655	0.68624
N	-0.64120	1.08270	1.44411
C	-1.04582	-0.12915	0.92094
C	-0.78090	-1.45874	1.28139
N	0.02219	-1.77686	2.32662
N	-1.37770	-2.43653	0.59239
C	-2.17410	-2.11026	-0.43047
N	-2.47160	-0.89965	-0.88675
C	-1.88983	0.05892	-0.16641
P	-6.18449	3.64335	-2.45886
O	-7.05028	4.01166	-3.63793
O	-6.43279	4.36194	-1.15511
O	-6.30377	2.03995	-2.15072
C	-6.31764	1.12768	-3.25081
C	-6.95235	-0.18362	-2.83700
O	-6.13288	-0.88769	-1.89839
C	-8.31413	-0.04967	-2.15389
O	-9.03603	-1.24601	-2.47070
C	-7.92909	0.04302	-0.68563
C	-6.72474	-0.88929	-0.60641
N	-5.74480	-0.50369	0.38489
C	-5.33131	0.75906	0.72193
N	-4.44243	0.78437	1.67882
C	-4.25463	-0.54610	1.99688
C	-3.45824	-1.19533	2.95171
N	-2.65308	-0.51333	3.80363
N	-3.53502	-2.52670	3.04785
C	-4.34925	-3.18562	2.21678
N	-5.12762	-2.68895	1.26362
C	-5.05278	-1.36001	1.20284
P	-10.38082	-1.71272	-1.66768
O	-11.24520	-2.47490	-2.64196
O	-11.01156	-0.56258	-0.92270
O	-9.76295	-2.73606	-0.54707
C	-9.21550	-3.97643	-1.00286
C	-9.16745	-4.97316	0.13390
O	-8.19487	-4.59406	1.11160
C	-10.48337	-5.13390	0.89408
O	-10.55199	-6.49217	1.30832
C	-10.31046	-4.18810	2.07581
C	-8.80615	-4.25308	2.34414

N	-8.24978	-3.00318	2.82317
C	-8.43121	-1.76424	2.26065
N	-7.72479	-0.82239	2.82148
C	-7.02362	-1.47671	3.81399
C	-6.07530	-1.04008	4.75165
N	-5.69045	0.25543	4.84013
N	-5.56233	-1.93548	5.60291
C	-5.94317	-3.21323	5.49901
N	-6.80454	-3.74958	4.64358
C	-7.32354	-2.83442	3.82193
H	12.71119	-3.71880	-0.62718
H	12.30597	-3.53299	-2.34888
H	11.34178	-1.78374	-0.86584
H	10.61819	-3.73243	0.94536
H	9.04901	-4.77415	-0.58505
H	8.10988	-1.93685	-1.26783
H	7.76669	-5.57859	-2.14624
H	6.67395	-0.03867	-5.18348
H	5.32389	-4.99417	-6.29673
H	4.91687	-3.61008	-7.26013
H	7.96926	-3.72668	0.39658
H	10.02002	1.29359	2.88244
H	10.15013	0.52308	1.28198
H	8.96965	2.66869	1.17474
H	7.64431	2.17699	3.51807
H	6.50980	0.22672	2.72432
H	5.78114	1.63855	0.12224
H	6.69510	-1.71618	1.76655
H	3.89240	0.34491	-3.62747
H	4.63389	-4.45822	-1.66217
H	3.81408	-4.10414	-3.13375
H	5.26057	1.45532	2.47718
H	5.10625	5.61148	0.29181
H	5.52350	4.08017	-0.51810
H	3.63794	5.31591	-1.56856
H	2.55462	5.85767	0.88827
H	2.22612	3.58808	1.53341
H	1.10174	2.83334	-1.19666
H	3.35061	1.81176	1.69487
H	0.12133	-1.13248	-2.98985
H	3.06028	-2.96472	0.91427
H	2.22633	-3.92306	-0.22402
H	0.63125	3.96523	0.87522
H	-1.33419	5.80979	-2.87169
H	-0.20090	4.43740	-2.84172
H	-2.41291	3.90580	-3.82496
H	-3.70250	5.26909	-1.82195
H	-2.87337	3.88152	-0.06424
H	-3.49587	1.36983	-1.68754
H	-1.16018	3.03666	0.79910
H	-2.62819	-2.94418	-0.95438
H	0.68016	-1.06715	2.61994
H	0.38660	-2.71951	2.32079
H	-4.45894	3.14893	-0.34069
H	-6.88852	1.55593	-4.07826
H	-5.29243	0.94047	-3.58176
H	-7.04678	-0.80330	-3.73038
H	-8.87640	0.81923	-2.49720
H	-7.64546	1.07055	-0.47342
H	-7.01545	-1.91020	-0.34586
H	-5.73252	1.62276	0.21448
H	-4.36724	-4.26339	2.33621
H	-2.39167	0.42291	3.52518
H	-1.91515	-1.05483	4.23194
H	-8.73606	-0.24045	-0.02037
H	-9.83848	-4.38089	-1.80427
H	-8.20477	-3.81068	-1.38566
H	-8.86970	-5.93819	-0.28194
H	-11.34265	-4.86992	0.27380
H	-10.60595	-3.18273	1.78251
H	-8.54950	-4.99993	3.09665
H	-9.10673	-1.64194	1.42745
H	-5.48544	-3.89593	6.20632
H	-5.85975	0.83490	4.02937
H	-4.80959	0.41727	5.30815
H	-10.89265	-4.49161	2.94416
H	-11.34807	-6.61112	1.83795
H	11.96614	-5.71109	-1.63707

****

ss-C6 M06-2X/TZVP

O	12.01473	-5.53846	-0.45413
C	13.00482	-4.53219	-0.62613
C	12.33818	-3.17504	-0.58783
O	11.51859	-2.99018	-1.74887
C	11.41467	-2.97313	0.61748
O	11.52204	-1.59527	0.99350
C	10.04619	-3.31346	0.05148
C	10.15493	-2.81070	-1.38390
N	9.31887	-3.52274	-2.34051
C	8.43969	-2.80487	-3.18277
O	8.39109	-1.58272	-3.11022
N	7.67167	-3.51966	-4.04506
C	7.76145	-4.83473	-4.10229
N	6.94172	-5.48026	-4.94444
C	8.69222	-5.58231	-3.31081
C	9.44849	-4.87336	-2.44965
P	10.61741	-0.97144	2.20529
O	11.48502	-0.01470	2.98708
O	9.91193	-2.05967	2.97680
O	9.47858	-0.14409	1.37135
C	9.90208	1.00752	0.63686
C	8.69483	1.78352	0.16200
O	7.98243	1.03990	-0.82913
C	7.67936	2.10636	1.26153
O	7.14359	3.39730	0.95209
C	6.65292	0.99398	1.10902
C	6.65800	0.75131	-0.39743
N	6.30758	-0.60061	-0.80621
C	5.45322	-0.79158	-1.91708
O	5.05299	0.18070	-2.54547
N	5.09260	-2.06414	-2.22759
C	5.62117	-3.08724	-1.58799
N	5.19598	-4.32092	-1.90804
C	6.59587	-2.93009	-0.54979
C	6.89865	-1.66322	-0.20231
P	5.91454	4.04903	1.81197
O	6.16615	5.53259	1.92502
O	5.67014	3.28362	3.08903
O	4.64090	3.76749	0.82375
C	4.59310	4.47092	-0.42036
C	3.20576	4.36556	-1.01121
O	2.93868	3.02268	-1.42292
C	2.08047	4.74390	-0.04349
O	1.07277	5.39690	-0.82283
C	1.62419	3.39387	0.48821
C	1.82835	2.48001	-0.71682
N	2.11956	1.08874	-0.39687
C	1.43713	0.05154	-1.07635
O	0.57264	0.33164	-1.89627
N	1.77668	-1.23030	-0.77687
C	2.72331	-1.49164	0.10222
N	2.97204	-2.77840	0.39754
C	3.46581	-0.46313	0.76833
C	3.12887	0.80658	0.46862
P	-0.35810	5.84423	-0.16794
O	-0.75509	7.16323	-0.78439
O	-0.32090	5.75826	1.33774
O	-1.34844	4.65642	-0.70523
C	-1.61741	4.61097	-2.10969
C	-2.76639	3.66812	-2.38438
O	-2.38405	2.31817	-2.11404
C	-4.01632	3.93211	-1.54021
O	-5.14019	3.64916	-2.37966
C	-3.87165	2.93370	-0.40252
C	-3.18792	1.75126	-1.08634
N	-2.33918	0.94464	-0.21966
C	-2.40662	-0.46695	-0.28420
O	-3.20316	-0.99988	-1.04687
N	-1.58016	-1.17903	0.52696
C	-0.72482	-0.56786	1.32119
N	0.02641	-1.32239	2.14099
C	-0.58399	0.85650	1.35683
C	-1.40674	1.55848	0.55310
P	-6.67511	3.72583	-1.81956

O	-7.52962	4.35015	-2.89597
O	-6.72520	4.34264	-0.44343
O	-7.04359	2.13916	-1.66315
C	-7.16190	1.36984	-2.86289
C	-7.72234	0.00380	-2.54122
O	-6.77495	-0.75726	-1.78735
C	-9.00704	0.02768	-1.70792
O	-9.80825	-1.06759	-2.16045
C	-8.49202	-0.17531	-0.29161
C	-7.30271	-1.10563	-0.51344
N	-6.24734	-0.99945	0.48188
C	-5.65836	-2.17396	1.00407
O	-6.00601	-3.26669	0.57476
N	-4.73552	-2.03215	1.99125
C	-4.32635	-0.83605	2.36458
N	-3.43920	-0.75715	3.36857
C	-4.80912	0.36961	1.75895
C	-5.76843	0.22466	0.82373
P	-11.22965	-1.43650	-1.43823
O	-12.17214	-1.91705	-2.51507
O	-11.69528	-0.31378	-0.54565
O	-10.79552	-2.68662	-0.47455
C	-10.44194	-3.91957	-1.10931
C	-10.43667	-5.03046	-0.08608
O	-9.35818	-4.84267	0.83345
C	-11.71283	-5.12221	0.75687
O	-11.96892	-6.50620	0.96128
C	-11.31826	-4.41931	2.04973
C	-9.83809	-4.77424	2.16743
N	-9.03460	-3.79064	2.88297
C	-8.28979	-4.16781	4.01984
O	-8.37544	-5.31394	4.44589
N	-7.50508	-3.22188	4.60116
C	-7.45690	-1.99343	4.11968
N	-6.68117	-1.10093	4.75281
C	-8.20720	-1.58218	2.97006
C	-8.96521	-2.52876	2.38352
H	13.74922	-4.57889	0.17470
H	13.51277	-4.64591	-1.58760
H	13.11400	-2.40707	-0.58313
H	11.68834	-3.60577	1.46095
H	9.91243	-4.39374	0.07951
H	9.87628	-1.76129	-1.46420
H	10.20546	-5.32423	-1.81736
H	8.78790	-6.65339	-3.40037
H	6.29881	-4.94934	-5.50941
H	6.97761	-6.47914	-5.05416
H	9.24469	-2.84054	0.60891
H	12.43861	-6.40134	-0.49884
H	10.51456	1.64724	1.27681
H	10.49350	0.69553	-0.22807
H	9.04856	2.71464	-0.28542
H	8.13050	2.12501	2.25348
H	7.00148	0.12458	1.66321
H	5.95047	1.40592	-0.90573
H	7.63518	-1.42250	0.55451
H	7.05728	-3.78019	-0.06969
H	4.60484	-4.43353	-2.71649
H	5.65514	-5.13630	-1.53767
H	5.67924	1.28489	1.48931
H	4.83648	5.52377	-0.25797
H	5.31785	4.03780	-1.11469
H	3.16265	5.01467	-1.88802
H	2.42033	5.41049	0.74906
H	2.26734	3.11159	1.32003
H	0.94878	2.46026	-1.35834
H	3.64572	1.66596	0.87949
H	4.25796	-0.69424	1.46465
H	2.53645	-3.49453	-0.16238
H	3.77371	-3.03317	0.95137
H	0.59627	3.42140	0.83435
H	-1.87985	5.61016	-2.46563
H	-0.72783	4.26341	-2.64190
H	-3.02310	3.75291	-3.44238
H	-4.07119	4.96168	-1.18690
H	-3.23816	3.37482	0.36518
H	-3.91315	1.06219	-1.51667
H	-1.36077	2.63704	0.46301

H	0.15166	1.33741	1.98368
H	0.01284	-2.32354	2.02597
H	0.77499	-0.91425	2.67623
H	-4.83036	2.68053	0.03836
H	-7.82895	1.87923	-3.56242
H	-6.17809	1.25478	-3.32520
H	-7.91471	-0.51161	-3.48439
H	-9.55988	0.95996	-1.82110
H	-8.18184	0.79012	0.10360
H	-7.61017	-2.15060	-0.50875
H	-6.19632	1.06144	0.28460
H	-4.42652	1.33919	2.04016
H	-3.02741	-1.60702	3.72038
H	-3.01922	0.12099	3.62442
H	-9.24973	-0.59583	0.36332
H	-11.16783	-4.15190	-1.89191
H	-9.44890	-3.82840	-1.55744
H	-10.28979	-5.97738	-0.61101
H	-12.55688	-4.63939	0.26023
H	-11.44674	-3.34309	1.93163
H	-9.68448	-5.72137	2.67835
H	-9.54710	-2.35856	1.48420
H	-8.15417	-0.57387	2.58768
H	-6.08950	-1.41361	5.50588
H	-6.53487	-0.17841	4.37791
H	-11.89499	-4.75970	2.90831
H	-12.74850	-6.59460	1.52076

****

ss-G6 M06-2X/TZVP

O	10.84266	5.43401	1.43360
C	11.54912	4.25160	1.77681
C	10.72033	3.04686	1.39153
O	9.55816	2.95330	2.22696
C	10.18965	3.08387	-0.04675
O	10.12395	1.72334	-0.48451
C	8.78927	3.65160	0.13516
C	8.38922	2.93585	1.41202
N	7.29885	3.53691	2.14082
C	6.79511	4.81982	2.07905
N	5.79826	5.01456	2.89211
C	5.63175	3.80652	3.53486
C	4.64966	3.38138	4.47958
O	3.71510	4.00078	4.96465
N	4.85149	2.03186	4.82522
C	5.79894	1.19491	4.29779
N	5.80362	-0.08152	4.75541
N	6.69447	1.58616	3.42901
C	6.55486	2.88123	3.07983
P	9.84394	1.36325	-2.04755
O	11.03099	1.73711	-2.90850
O	8.52745	1.94199	-2.53507
O	9.74510	-0.25019	-1.92609
C	9.68359	-1.04037	-0.73965
C	8.27028	-1.47512	-0.41744
O	7.53989	-0.39467	0.18084
C	7.45628	-1.90504	-1.65371
O	6.67188	-3.03979	-1.26899
C	6.55106	-0.70448	-1.88539
C	6.25762	-0.34438	-0.43916
N	5.65527	0.94186	-0.20968
C	5.81649	2.10554	-0.93391
N	5.05214	3.07239	-0.50879
C	4.34839	2.52764	0.54436
C	3.30495	3.07801	1.35267
O	2.77213	4.17394	1.29766
N	2.87547	2.13561	2.31013
C	3.31050	0.84218	2.42051
N	2.76672	0.10426	3.43392
N	4.23475	0.33082	1.65506
C	4.71506	1.20891	0.74603
P	6.02976	-4.02699	-2.40271
O	7.01239	-5.12250	-2.74941
O	5.47525	-3.23789	-3.56810
O	4.76387	-4.58866	-1.55417
C	4.94068	-5.14022	-0.25086
C	3.73359	-4.80417	0.60290

O	3.58849	-3.38157	0.70057
C	2.38692	-5.31995	0.07823
O	1.58551	-5.57377	1.24447
C	1.89865	-4.13972	-0.74141
C	2.34954	-2.98040	0.12875
N	2.49311	-1.73272	-0.58008
C	3.18877	-1.45955	-1.73640
N	3.02940	-0.22902	-2.14129
C	2.17594	0.33761	-1.21693
C	1.59374	1.64091	-1.14388
O	1.70588	2.59074	-1.90193
N	0.77171	1.75287	0.00096
C	0.52100	0.76345	0.91522
N	-0.23878	1.07963	2.00316
N	1.03319	-0.43290	0.82400
C	1.83949	-0.58575	-0.24355
P	-0.04405	-5.52791	1.28242
O	-0.48658	-6.33334	2.47858
O	-0.65257	-5.86604	-0.05926
O	-0.35736	-3.93915	1.51736
C	-0.32315	-3.34138	2.81053
C	-1.62981	-2.62207	3.07699
O	-1.79850	-1.47764	2.22918
C	-2.87375	-3.47632	2.82973
O	-3.85479	-3.05354	3.78394
C	-3.20789	-3.17568	1.37027
C	-2.67434	-1.76077	1.14344
N	-1.99110	-1.58922	-0.13087
C	-1.32297	-2.51255	-0.91198
N	-0.78275	-1.98636	-1.97415
C	-1.09420	-0.64682	-1.89828
C	-0.83376	0.41947	-2.81232
O	-0.26770	0.39350	-3.89382
N	-1.35066	1.63189	-2.31641
C	-2.04093	1.78874	-1.14574
N	-2.36130	3.06533	-0.79422
N	-2.32416	0.80113	-0.33975
C	-1.83726	-0.38584	-0.76147
P	-5.46539	-3.02935	3.53965
O	-6.11469	-2.99033	4.90055
O	-5.93051	-4.09773	2.57857
O	-5.65485	-1.59909	2.76923
C	-5.31223	-0.40579	3.47391
C	-6.04737	0.76808	2.86732
O	-5.58987	1.04199	1.54102
C	-7.56085	0.57626	2.73465
O	-8.14775	1.87154	2.91036
C	-7.69570	-0.02022	1.33681
C	-6.52879	0.59647	0.57237
N	-5.89278	-0.31391	-0.36504
C	-5.79173	-1.69038	-0.35555
N	-5.13331	-2.15889	-1.37790
C	-4.77330	-1.04343	-2.10315
C	-4.09294	-0.91614	-3.35272
O	-3.67445	-1.78036	-4.10636
N	-3.94876	0.44429	-3.69964
C	-4.45100	1.51129	-3.00198
N	-4.18018	2.75484	-3.49186
N	-5.10092	1.38624	-1.87914
C	-5.23237	0.10474	-1.48304
P	-9.45661	2.44957	2.13248
O	-9.94489	3.63546	2.92769
O	-10.46623	1.37957	1.79298
O	-8.80914	2.94048	0.71288
C	-7.83987	3.98882	0.72934
C	-7.83687	4.67974	-0.61574
O	-7.37320	3.79582	-1.63954
C	-9.21033	5.16024	-1.08004
O	-8.98782	6.31981	-1.87182
C	-9.71851	3.99087	-1.91602
C	-8.43104	3.40613	-2.49855
N	-8.45087	1.96002	-2.63343
C	-8.98210	1.01527	-1.77902
N	-8.72515	-0.20700	-2.14931
C	-7.97454	-0.07292	-3.29774
C	-7.36952	-1.06094	-4.13561
O	-7.41675	-2.27679	-4.06397
N	-6.62804	-0.44522	-5.16645

C	-6.49448	0.90229	-5.36702
N	-5.64309	1.29558	-6.35270
N	-7.07644	1.80066	-4.61881
C	-7.78374	1.26295	-3.60047
H	12.50380	4.19768	1.24348
H	11.74546	4.20857	2.85176
H	11.32816	2.14949	1.52611
H	10.82186	3.67206	-0.71325
H	8.87551	4.72341	0.30343
H	8.09981	1.90127	1.20646
H	7.20307	5.55642	1.40653
H	4.16866	1.64971	5.46921
H	6.36180	-0.72060	4.20905
H	4.93607	-0.46348	5.10296
H	8.13722	3.43511	-0.70494
H	11.34286	6.19337	1.74778
H	10.28477	-1.92937	-0.93393
H	10.10473	-0.50025	0.10752
H	8.31706	-2.31025	0.28647
H	8.09449	-2.14485	-2.50433
H	7.11946	0.10045	-2.35511
H	5.58756	-1.07749	0.02042
H	6.54225	2.15961	-1.73471
H	2.14568	2.46278	2.93513
H	2.90851	-0.88959	3.30921
H	1.81190	0.33297	3.68449
H	5.67250	-0.95372	-2.47102
H	5.05643	-6.22447	-0.32115
H	5.82724	-4.71568	0.22296
H	3.90296	-5.21089	1.60181
H	2.47953	-6.24095	-0.49687
H	2.43422	-4.12916	-1.68960
H	1.62497	-2.77791	0.91643
H	3.79600	-2.21122	-2.22431
H	0.34103	2.66394	0.12603
H	-0.72686	0.26293	2.36967
H	-0.84625	1.88245	1.90685
H	0.83018	-4.13904	-0.92569
H	-0.17254	-4.10136	3.58021
H	0.49579	-2.61856	2.83932
H	-1.62047	-2.26170	4.10641
H	-2.67554	-4.53763	2.98673
H	-2.68448	-3.90074	0.75347
H	-3.46639	-1.00766	1.15503
H	-1.27410	-3.55691	-0.64773
H	-1.15402	2.44723	-2.88576
H	-3.05573	3.11937	-0.06177
H	-2.51258	3.72289	-1.54728
H	-4.26673	-3.25228	1.15364
H	-5.58878	-0.49857	4.52765
H	-4.23348	-0.23864	3.39875
H	-5.83922	1.64869	3.47669
H	-7.96208	-0.08863	3.50003
H	-7.57592	-1.09562	1.43238
H	-6.83196	1.45825	-0.02541
H	-6.20786	-2.28675	0.44133
H	-3.49069	0.61026	-4.58951
H	-4.80960	3.46126	-3.13137
H	-4.00596	2.83361	-4.48467
H	-8.65379	0.18169	0.86961
H	-8.08216	4.71462	1.51009
H	-6.85058	3.56506	0.92715
H	-7.15031	5.52713	-0.56498
H	-9.86686	5.38301	-0.23572
H	-10.21992	3.27494	-1.27018
H	-8.22226	3.78800	-3.49792
H	-9.54090	1.29981	-0.90060
H	-6.12564	-1.08774	-5.76839
H	-5.73853	2.26044	-6.63408
H	-5.43830	0.65506	-7.10525
H	-10.40622	4.30899	-2.69771
H	-9.83167	6.60835	-2.23677

****

ss-T6 M06-2X/TZVP

C	-8.63974	-3.07724	1.53643
N	-7.89531	-4.23976	1.53192

C	-6.92721	-4.48024	2.48962
N	-6.84845	-3.51344	3.46744
C	-7.56780	-2.33103	3.55461
C	-8.54002	-2.13199	2.48927
C	-8.13787	-5.25936	0.52119
O	-7.80596	-4.74640	-0.75905
C	-8.63637	-5.35746	-1.75377
C	-9.61849	-6.26999	-1.00603
C	-9.58761	-5.71572	0.41103
C	-9.30133	-4.27861	-2.57783
O	-10.14853	-3.52078	-1.71413
P	-10.33674	-1.92020	-1.90153
O	-10.89652	-1.38053	-0.60260
O	-9.08602	-7.58737	-1.07171
O	-6.21640	-5.46581	2.50888
O	-7.36402	-1.55191	4.47090
C	-9.40275	-0.91104	2.55089
O	-8.76447	-1.52041	-2.10058
C	-8.34079	-0.16643	-1.92309
C	-6.94316	-0.03213	-2.56537
O	-6.00337	-0.06531	-1.48514
C	-6.69544	-0.57504	-0.34997
C	-8.06020	0.08600	-0.44912
C	-6.76128	1.23073	-3.37617
O	-7.34374	2.36190	-2.73098
P	-6.59739	3.50733	-1.86350
O	-6.90224	3.33619	-0.38420
N	-5.92713	-0.32247	0.84962
C	-5.28385	-1.39057	1.44950
N	-4.63689	-1.06996	2.62099
C	-4.47604	0.18687	3.19032
C	-5.08626	1.27094	2.43657
C	-5.77576	0.96409	1.32184
O	-5.30283	-2.52760	1.02000
O	-3.85570	0.31340	4.23280
C	-4.93109	2.66804	2.95069
O	-5.04943	3.13125	-2.20473
C	-3.98705	3.91488	-1.65276
C	-2.69065	3.48937	-2.37659
O	-1.95945	2.68016	-1.44934
C	-2.87815	2.27230	-0.44092
C	-3.71451	3.51953	-0.20681
C	-1.84162	4.64950	-2.84341
O	-1.76427	5.67818	-1.85838
P	-0.56056	5.94769	-0.80921
O	-0.98507	5.55403	0.59634
N	-2.14861	1.75143	0.69576
C	-2.20531	0.39116	0.94278
N	-1.53541	-0.00207	2.07851
C	-0.77152	0.78182	2.93350
C	-0.67959	2.18058	2.54522
C	-1.36829	2.59002	1.46337
O	-2.81374	-0.40265	0.25203
O	-0.23273	0.28371	3.90741
C	0.15919	3.09267	3.38419
O	0.54960	4.91781	-1.41235
C	1.84701	4.84277	-0.81382
C	2.73805	4.01239	-1.76472
O	2.90373	2.73192	-1.14699
C	1.86874	2.60805	-0.17754
C	1.81390	3.99149	0.44993
C	4.08442	4.63740	-2.05036
O	4.67652	5.20237	-0.88147
P	5.78015	4.52155	0.08882
O	5.14177	4.04672	1.38409
N	2.16235	1.50096	0.70664
C	1.39348	0.35506	0.60203
N	1.68942	-0.60640	1.54066
C	2.70780	-0.59216	2.48505
C	3.54386	0.59751	2.44941
C	3.22805	1.57425	1.57830
O	0.50610	0.20101	-0.21444
O	2.85159	-1.53222	3.24842
C	4.69935	0.67711	3.39679
O	6.22140	3.26918	-0.85602
C	7.24662	2.37238	-0.41781
C	7.60788	1.47710	-1.62615
O	7.08437	0.17716	-1.33675

C	6.07123	0.36695	-0.35277
C	6.70214	1.37361	0.59578
C	9.08587	1.40153	-1.93494
O	9.90102	1.29896	-0.76832
P	10.41298	-0.05918	-0.05031
O	9.49877	-0.46090	1.09544
N	5.69545	-0.91121	0.20884
C	4.44116	-1.41685	-0.08479
N	4.15992	-2.61105	0.53627
C	5.01187	-3.39472	1.30315
C	6.34448	-2.83901	1.48221
C	6.61092	-1.63634	0.93955
O	3.62897	-0.85567	-0.79383
O	4.62445	-4.45818	1.75831
C	7.34175	-3.63452	2.26430
O	10.28003	-1.08118	-1.31439
C	10.59928	-2.46810	-1.16005
C	10.56157	-3.08121	-2.56281
O	9.20434	-3.49469	-2.76401
C	8.44954	-3.30652	-1.57209
C	9.47053	-3.19585	-0.44795
C	11.50251	-4.25053	-2.75345
O	11.28839	-5.19478	-1.71246
N	7.51018	-4.40823	-1.43357
C	6.17004	-4.16650	-1.65951
N	5.36312	-5.26426	-1.46660
C	5.74880	-6.56025	-1.14091
C	7.18934	-6.75816	-1.05062
C	7.98353	-5.68635	-1.20555
O	5.71749	-3.08724	-1.99152
O	4.90896	-7.42723	-0.97984
C	7.69833	-8.14063	-0.79321
O	11.85893	0.11472	0.35816
O	6.93316	5.47968	0.29293
O	-0.08788	7.38012	-0.92537
O	-6.95969	4.87269	-2.40625
O	-11.12000	-1.57309	-3.14807
H	12.53112	-3.87697	-2.73562
H	11.30837	-4.69827	-3.73183
H	10.80629	-2.31366	-3.29948
H	11.57179	-2.58251	-0.67870
H	9.82206	-4.17191	-0.12157
H	7.85023	-2.39848	-1.64447
H	9.06483	-5.76408	-1.18552
H	4.37050	-5.10041	-1.58813
H	9.09844	-2.62973	0.39792
H	11.84922	-5.96074	-1.86994
H	9.38195	2.33116	-2.42144
H	9.27828	0.56875	-2.61092
H	7.09750	1.87940	-2.50725
H	8.10392	2.93860	-0.05351
H	7.53303	0.91475	1.13407
H	5.16705	0.77937	-0.80548
H	7.58770	-1.16725	1.03376
H	3.23561	-2.98479	0.35540
H	5.99830	1.83576	1.27995
H	3.94306	5.46282	-2.74855
H	4.74754	3.89828	-2.49805
H	2.20607	3.88862	-2.71300
H	2.24073	5.84616	-0.65224
H	2.71346	4.18246	1.03757
H	0.91741	2.37208	-0.65878
H	3.81565	2.48694	1.50871
H	1.10604	-1.43421	1.51001
H	0.91805	4.17326	1.03461
H	-2.31407	5.09750	-3.71826
H	-0.84649	4.29784	-3.11251
H	-2.96422	2.88509	-3.24650
H	-4.20172	4.97591	-1.78022
H	-3.11855	4.29715	0.27497
H	-3.50512	1.45412	-0.80050
H	-1.33516	3.62436	1.12705
H	-1.60674	-0.98753	2.30301
H	-4.63266	3.33415	0.34130
H	-7.28474	1.11576	-4.32587
H	-5.70216	1.39836	-3.56791
H	-6.77328	-0.89369	-3.21765
H	-9.06315	0.52414	-2.35864

H	-7.98806	1.15872	-0.26142
H	-6.79589	-1.65898	-0.42153
H	-6.25160	1.73364	0.71814
H	-4.19989	-1.84894	3.09940
H	-8.81657	-0.37381	0.17606
H	-9.89833	-4.73362	-3.37146
H	-8.53671	-3.64008	-3.02055
H	-8.01845	-5.97281	-2.41232
H	-10.61592	-6.23355	-1.44819
H	-10.26951	-4.86916	0.48495
H	-7.48341	-6.09107	0.77351
H	-9.33187	-2.96842	0.70683
H	-6.15725	-3.68428	4.18815
H	-9.84489	-6.46108	1.16214
H	-9.67262	-8.17902	-0.58742
H	8.78671	-8.14978	-0.76727
H	7.32164	-8.51757	0.15912
H	7.35953	-8.82715	-1.57075
H	8.23350	-3.04296	2.46734
H	6.91295	-3.95896	3.21316
H	7.63708	-4.53404	1.71586
H	5.14666	1.67003	3.37405
H	4.37345	0.46146	4.41519
H	5.46645	-0.05896	3.13775
H	0.01977	4.12922	3.07957
H	-0.10910	2.99628	4.43702
H	1.22001	2.84000	3.29384
H	-5.56718	3.35303	2.39137
H	-5.20280	2.71835	4.00582
H	-3.89369	3.00515	2.86414
H	-10.12405	-0.91546	1.73391
H	-9.93931	-0.88149	3.50095
H	-8.79912	-0.00170	2.49335

****

ss-A2C2A2 M06-2X/TZVP

O	11.59757	-5.19179	-1.06123
C	12.15176	-3.91544	-1.34222
C	11.10921	-2.85542	-1.06725
O	10.04960	-2.94594	-2.02844
C	10.43501	-2.98558	0.30353
O	10.10770	-1.66448	0.74302
C	9.15562	-3.74342	-0.02035
C	8.80696	-3.11240	-1.35777
N	7.92815	-3.88631	-2.20255
C	7.59999	-5.22087	-2.15511
N	6.82309	-5.60113	-3.13185
C	6.63497	-4.45775	-3.88055
C	5.89008	-4.18567	-5.04262
N	5.14484	-5.12200	-5.65074
N	5.92404	-2.94456	-5.54368
C	6.62922	-2.00880	-4.89864
N	7.34736	-2.13429	-3.79102
C	7.31293	-3.38477	-3.32313
P	9.92935	-1.36723	2.33969
O	11.28487	-1.25134	2.99946
O	8.95949	-2.33230	2.98332
O	9.17483	0.06489	2.21839
C	9.78204	1.14719	1.51330
C	8.69350	2.01919	0.92356
O	7.96345	1.30374	-0.08010
C	7.64722	2.49283	1.93413
O	7.20725	3.77904	1.48964
C	6.56983	1.42541	1.82190
C	6.63614	1.03414	0.34851
N	6.29770	-0.35426	0.10178
C	6.53933	-1.45165	0.89753
N	6.01247	-2.55390	0.43620
C	5.38742	-2.17131	-0.73120
C	4.61630	-2.87525	-1.66911
N	4.31887	-4.18518	-1.51067
N	4.12764	-2.21255	-2.71975
C	4.36904	-0.89993	-2.81783
N	5.05633	-0.12433	-1.99002
C	5.55095	-0.81256	-0.95923
P	5.92335	4.53082	2.17058
O	6.18851	6.01494	2.11594

O	5.58205	3.92832	3.51073
O	4.72591	4.13075	1.12825
C	4.82575	4.65916	-0.19739
C	3.54875	4.37941	-0.95227
O	3.41630	2.97265	-1.17023
C	2.27425	4.82652	-0.22567
O	1.38392	5.33352	-1.22526
C	1.76891	3.53409	0.39610
C	2.18162	2.50193	-0.64757
N	2.37817	1.15202	-0.14408
C	1.84245	0.05490	-0.85825
O	1.23764	0.25083	-1.90455
N	2.02295	-1.18628	-0.33552
C	2.72821	-1.36330	0.76368
N	2.83723	-2.61477	1.24452
C	3.34970	-0.27877	1.46589
C	3.14581	0.95383	0.95809
P	-0.11720	5.85201	-0.82899
O	-0.43384	7.04991	-1.69061
O	-0.25719	6.00773	0.66538
O	-1.03977	4.57715	-1.27578
C	-1.19476	4.31911	-2.67368
C	-2.33191	3.34715	-2.89370
O	-1.98586	2.05243	-2.39293
C	-3.63962	3.73262	-2.19290
O	-4.70188	3.30874	-3.05614
C	-3.57486	2.92642	-0.90615
C	-2.89625	1.64744	-1.37524
N	-2.15311	0.92403	-0.35458
C	-2.27552	-0.48130	-0.26472
O	-3.03432	-1.07231	-1.02214
N	-1.53154	-1.12144	0.67613
C	-0.70150	-0.45369	1.45103
N	-0.04580	-1.13666	2.40668
C	-0.50226	0.96038	1.33445
C	-1.24788	1.59228	0.40588
P	-6.26946	3.34223	-2.59295
O	-7.09350	3.76407	-3.78523
O	-6.45068	4.11258	-1.30716
O	-6.55185	1.77194	-2.24130
C	-6.66579	0.82146	-3.29878
C	-7.42411	-0.39420	-2.80936
O	-6.69709	-1.08829	-1.79228
C	-8.78849	-0.09765	-2.18429
O	-9.59818	-1.25031	-2.44115
C	-8.43909	0.09751	-0.71531
C	-7.26695	-0.85913	-0.51106
N	-6.26154	-0.36433	0.41018
C	-5.90500	0.92783	0.70592
N	-4.97035	1.01879	1.61454
C	-4.68954	-0.29180	1.94269
C	-3.79851	-0.87589	2.85422
N	-2.98878	-0.13229	3.64608
N	-3.79610	-2.20649	2.98035
C	-4.62728	-2.92529	2.21639
N	-5.48626	-2.49225	1.30322
C	-5.48153	-1.16295	1.20834
P	-10.92378	-1.64632	-1.57325
O	-11.82440	-2.44746	-2.48139
O	-11.52364	-0.45235	-0.87315
O	-10.28002	-2.61553	-0.41947
C	-9.70320	-3.84959	-0.85575
C	-9.54265	-4.78623	0.31883
O	-8.55012	-4.28917	1.22018
C	-10.80892	-4.98871	1.15154
O	-10.78450	-6.33398	1.61090
C	-10.62576	-3.99677	2.29312
C	-9.11076	-3.99721	2.48884
N	-8.58017	-2.73342	2.95470
C	-8.81734	-1.49798	2.40503
N	-8.10989	-0.54077	2.93778
C	-7.34840	-1.18033	3.89542
C	-6.35606	-0.72787	4.77891
N	-5.97779	0.57180	4.83493
N	-5.79189	-1.61130	5.61001
C	-6.15712	-2.89556	5.52805
N	-7.04326	-3.44893	4.71013
C	-7.61585	-2.54430	3.91287

H	13.02210	-3.71737	-0.70812
H	12.45695	-3.84102	-2.38967
H	11.57814	-1.87257	-1.14674
H	11.07123	-3.49372	1.02977
H	9.40182	-4.79629	-0.14745
H	8.33118	-2.13488	-1.22896
H	7.95575	-5.86302	-1.36555
H	6.60809	-1.02047	-5.34559
H	5.16421	-6.07584	-5.33111
H	4.67735	-4.90106	-6.51438
H	8.38723	-3.60524	0.73426
H	10.39518	1.73486	2.20051
H	10.40851	0.77008	0.70301
H	9.16540	2.88393	0.45400
H	8.04807	2.57397	2.94443
H	6.84598	0.58985	2.45980
H	5.93797	1.61571	-0.26099
H	7.13469	-1.39317	1.79701
H	3.94509	-0.41312	-3.68941
H	4.87718	-4.72071	-0.86304
H	3.97352	-4.67872	-2.32060
H	5.59230	1.78994	2.12046
H	4.99384	5.73795	-0.15322
H	5.66386	4.18777	-0.71813
H	3.60600	4.88635	-1.91807
H	2.46865	5.59836	0.51824
H	2.28689	3.37971	1.34112
H	1.43939	2.41934	-1.44082
H	3.58702	1.84577	1.38595
H	3.95277	-0.43817	2.34757
H	2.54621	-3.37121	0.64209
H	3.54475	-2.82356	1.93115
H	0.69927	3.55864	0.57885
H	-1.41240	5.25255	-3.19800
H	-0.27105	3.89105	-3.07222
H	-2.50662	3.27144	-3.96891
H	-3.71740	4.80543	-2.01594
H	-2.96029	3.46955	-0.19089
H	-3.61849	0.93931	-1.77767
H	-1.16084	2.65225	0.20195
H	0.20638	1.48964	1.95420
H	-0.08671	-2.14416	2.38915
H	0.70628	-0.70566	2.92018
H	-4.54974	2.75025	-0.46911
H	-7.19709	1.26518	-4.14447
H	-5.66799	0.51405	-3.62228
H	-7.54746	-1.07526	-3.65284
H	-9.26713	0.78131	-2.61743
H	-8.13483	1.13127	-0.58220
H	-7.58716	-1.82102	-0.10063
H	-6.36473	1.76970	0.21072
H	-4.58318	-3.99924	2.36134
H	-2.83063	0.82729	3.37558
H	-2.20477	-0.61024	4.06628
H	-9.27308	-0.10531	-0.05243
H	-10.35114	-4.31705	-1.60142
H	-8.72468	-3.65561	-1.30444
H	-9.20686	-5.75225	-0.06469
H	-11.71087	-4.78978	0.56867
H	-10.97028	-3.01380	1.97724
H	-8.78726	-4.74290	3.21665
H	-9.53407	-1.38599	1.60454
H	-5.65828	-3.56758	6.21767
H	-6.17240	1.13334	4.01657
H	-5.07921	0.74260	5.26483
H	-11.15679	-4.29109	3.19668
H	-11.54810	-6.47710	2.18086
H	12.23793	-5.86638	-1.30660

****

ds-A4_M06-2X/TZVP

O	2.45727	-7.00780	-6.03441
C	3.53923	-6.53928	-6.82965
C	4.36652	-5.57040	-6.01415
O	3.65814	-4.33915	-5.81131
C	4.72302	-6.09005	-4.61758
O	6.05307	-5.64133	-4.33737

C	3.67060	-5.43902	-3.73527
C	3.39964	-4.11284	-4.43192
N	2.02518	-3.62771	-4.29711
C	1.80486	-2.32744	-3.87519
O	2.68024	-1.60581	-3.43716
N	0.50379	-1.89697	-3.98932
C	-0.54393	-2.58178	-4.56874
O	-1.62122	-2.02411	-4.75828
C	-0.26515	-3.95996	-4.93474
C	-1.37589	-4.79318	-5.49111
C	0.99988	-4.39580	-4.80708
P	6.73166	-5.75743	-2.85425
O	8.19826	-6.05509	-3.04852
O	5.94993	-6.67206	-1.94244
O	6.53224	-4.23275	-2.29652
C	7.08293	-3.17601	-3.08228
C	6.91632	-1.86728	-2.35016
O	5.53131	-1.55196	-2.20913
C	7.52330	-1.84098	-0.94200
O	8.19723	-0.58408	-0.81399
C	6.31398	-2.02790	-0.03578
C	5.14386	-1.47496	-0.84483
N	3.88118	-2.19162	-0.65092
C	2.70830	-1.44828	-0.61128
O	2.68505	-0.23258	-0.58131
N	1.55145	-2.18849	-0.57881
C	1.44106	-3.55544	-0.67986
O	0.33678	-4.08760	-0.79245
C	2.69520	-4.28435	-0.65621
C	2.65222	-5.77952	-0.61258
C	3.83444	-3.56728	-0.69769
P	8.46695	0.22214	0.57603
O	9.70189	1.06400	0.36551
O	8.43592	-0.66851	1.79476
O	7.13508	1.16899	0.64489
C	6.85754	2.00256	-0.48058
C	5.73241	2.94462	-0.12534
O	4.53730	2.20246	0.12107
C	5.99319	3.78417	1.12965
O	5.46230	5.08652	0.87093
C	5.24990	3.01925	2.21424
C	4.09987	2.35443	1.46450
N	3.69409	1.05447	1.99537
C	2.33792	0.76176	2.03541
O	1.46779	1.56796	1.76326
N	2.03099	-0.51281	2.44422
C	2.91516	-1.52348	2.73891
O	2.50644	-2.65580	2.99493
C	4.31861	-1.15456	2.70867
C	5.32876	-2.17272	3.13331
C	4.62936	0.09120	2.30354
P	5.25128	6.21155	2.03854
O	5.54415	7.56171	1.42865
O	5.98645	5.85425	3.30777
O	3.65208	6.05899	2.33482
C	2.77103	6.27517	1.22810
C	1.34366	6.11828	1.68499
O	1.13687	4.77576	2.12900
C	0.93260	7.02986	2.84612
O	-0.41013	7.43140	2.59673
C	1.02996	6.11205	4.05793
C	0.64451	4.76329	3.45678
N	1.18986	3.60060	4.14319
C	0.36349	2.50879	4.35299
O	-0.82728	2.50417	4.09800
N	0.98653	1.41731	4.90570
C	2.31888	1.29541	5.22467
O	2.75533	0.24238	5.68554
C	3.13632	2.46895	4.97519
C	4.58030	2.40467	5.35841
C	2.53703	3.53753	4.41924
O	-5.05020	-7.07308	6.12426
C	-6.12158	-6.17262	6.36308
C	-6.12043	-5.10096	5.29578
O	-4.97351	-4.25405	5.43979
C	-6.05059	-5.63859	3.86298
O	-6.73121	-4.69639	3.03069
C	-4.56156	-5.58829	3.55700

C	-4.19708	-4.28373	4.24630
N	-2.80693	-4.12459	4.59880
C	-1.82584	-5.06752	4.80016
N	-0.68713	-4.55991	5.18189
C	-0.92525	-3.20369	5.24728
C	-0.09297	-2.10357	5.51940
N	1.20721	-2.23254	5.82566
N	-0.65010	-0.88123	5.49553
C	-1.94482	-0.75190	5.17533
N	-2.80615	-1.70536	4.86471
C	-2.23553	-2.91507	4.90089
P	-7.40402	-5.17998	1.62437
O	-8.69463	-5.92082	1.89654
O	-6.40512	-5.89952	0.74511
O	-7.65230	-3.71597	0.97240
C	-8.33360	-2.68175	1.68121
C	-7.74880	-1.34152	1.28046
O	-6.38286	-1.24836	1.69823
C	-7.72567	-1.08269	-0.22655
O	-7.77460	0.33867	-0.39515
C	-6.36015	-1.60771	-0.63497
C	-5.51758	-1.19608	0.56538
N	-4.35599	-2.02084	0.80595
C	-4.22263	-3.38667	0.69209
N	-3.05112	-3.82283	1.06988
C	-2.37315	-2.68959	1.46597
C	-1.07244	-2.48242	1.96332
N	-0.19688	-3.47559	2.14972
N	-0.73065	-1.22775	2.29915
C	-1.61424	-0.23298	2.13910
N	-2.84432	-0.30893	1.65806
C	-3.16996	-1.56391	1.32857
P	-8.18666	1.00260	-1.83127
O	-9.62810	1.45821	-1.78602
O	-7.80695	0.10240	-2.98463
O	-7.16849	2.26882	-1.84353
C	-7.16425	3.20591	-0.76895
C	-5.88978	4.02622	-0.82771
O	-4.74472	3.18063	-0.70261
C	-5.65799	4.79334	-2.12866
O	-4.76443	5.86178	-1.79766
C	-4.93188	3.77426	-2.98969
C	-4.05591	3.08385	-1.94874
N	-3.75935	1.69534	-2.22158
C	-4.53320	0.73565	-2.83500
N	-4.02235	-0.46300	-2.76096
C	-2.85660	-0.29830	-2.04316
C	-1.88550	-1.20643	-1.57967
N	-1.97043	-2.52626	-1.76749
N	-0.85448	-0.70271	-0.88150
C	-0.80290	0.61121	-0.62167
N	-1.67195	1.54348	-0.97632
C	-2.67968	1.03099	-1.69071
P	-4.54118	7.14022	-2.78866
O	-5.50902	8.24620	-2.43329
O	-4.50979	6.72655	-4.24268
O	-3.01639	7.51483	-2.36110
C	-2.69687	7.73104	-0.98657
C	-1.23738	7.41020	-0.74842
O	-0.97558	6.02902	-1.03814
C	-0.24243	8.20248	-1.59476
O	0.92980	8.35055	-0.79954
C	0.00023	7.28578	-2.78375
C	-0.10324	5.89898	-2.15387
N	-0.61009	4.88725	-3.05892
C	-1.70507	4.98411	-3.88790
N	-2.00354	3.86257	-4.48374
C	-1.06328	2.97004	-4.01994
C	-0.91000	1.58755	-4.21560
N	-1.74492	0.87814	-4.99138
N	0.11750	0.98161	-3.60154
C	0.92331	1.70321	-2.80412
N	0.84037	2.99210	-2.51324
C	-0.18684	3.58028	-3.13667
H	4.18152	-7.37046	-7.13595
H	3.17418	-6.02678	-7.72382
H	5.28252	-5.34897	-6.56412
H	4.69109	-7.17774	-4.56208

H	2.78049	-6.06670	-3.73168
H	4.03513	-3.31312	-4.04847
H	1.30067	-5.38404	-5.13633
H	0.35210	-0.89640	-3.75319
H	4.01435	-5.30437	-2.71747
H	8.14573	-3.35863	-3.26448
H	6.56219	-3.12324	-4.04191
H	7.39127	-1.08752	-2.94988
H	8.25709	-2.63431	-0.80008
H	6.20458	-3.09208	0.16164
H	4.92758	-0.43764	-0.58130
H	4.79380	-4.06001	-0.79480
H	0.66410	-1.63631	-0.61592
H	6.42824	-1.52040	0.91424
H	7.74713	2.57773	-0.75226
H	6.55983	1.38252	-1.33065
H	5.56171	3.61050	-0.97360
H	7.05739	3.87126	1.35078
H	5.93400	2.29150	2.64427
H	3.19974	2.96988	1.49326
H	5.66195	0.39488	2.18262
H	1.01450	-0.75002	2.45006
H	4.89857	3.67276	3.00323
H	2.91567	7.27780	0.81942
H	2.97915	5.53715	0.44850
H	0.69604	6.31668	0.82898
H	1.58715	7.90009	2.92606
H	2.05408	6.11130	4.42598
H	-0.43506	4.62789	3.45404
H	3.09617	4.42581	4.14917
H	0.37533	0.60232	5.11388
H	0.36078	6.40801	4.86417
H	-0.71086	7.97612	3.33278
H	-7.08452	-6.69265	6.32928
H	-6.01828	-5.69012	7.33895
H	-7.02308	-4.49705	5.40991
H	-6.48735	-6.63410	3.77025
H	-4.07540	-6.43158	4.04320
H	-4.45435	-3.42154	3.62350
H	-2.01198	-6.11761	4.64161
H	-2.31527	0.26767	5.15632
H	1.65091	-3.10595	5.58639
H	1.78731	-1.39472	5.80483
H	-4.35503	-5.57449	2.49091
H	-9.39779	-2.71003	1.43545
H	-8.20788	-2.81060	2.75736
H	-8.32088	-0.55691	1.77897
H	-8.55321	-1.56573	-0.74734
H	-6.40238	-2.69035	-0.72897
H	-5.13587	-0.17778	0.45563
H	-5.03458	-4.01805	0.35727
H	-1.26154	0.74940	2.44093
H	-0.37067	-4.36179	1.70336
H	0.74570	-3.25427	2.46575
H	-6.00526	-1.16728	-1.56071
H	-8.03649	3.86166	-0.83679
H	-7.18807	2.67474	0.18517
H	-5.89897	4.72250	0.01265
H	-6.58072	5.17425	-2.56916
H	-5.65378	3.08478	-3.41918
H	-3.08677	3.57749	-1.84265
H	-5.48304	0.96718	-3.29242
H	0.05911	0.93190	-0.04250
H	-2.67097	-2.88652	-2.39437
H	-1.23542	-3.13564	-1.41436
H	-4.34722	4.24202	-3.77425
H	-2.89309	8.77294	-0.72083
H	-3.30326	7.08095	-0.35371
H	-1.02721	7.58577	0.30940
H	-0.63850	9.17693	-1.88808
H	-0.78565	7.43308	-3.52142
H	0.86324	5.52920	-1.80829
H	-2.26227	5.90277	-3.99645
H	1.72039	1.14326	-2.32417
H	-2.58447	1.33624	-5.31059
H	-1.75395	-0.13820	-4.90705
H	0.96894	7.44869	-3.25352
H	1.61631	8.76333	-1.33567

H	-1.01182	-5.78250	-5.76474
H	-2.17727	-4.90687	-4.75838
H	-1.80824	-4.32082	-6.37445
H	3.65128	-6.20079	-0.72815
H	2.23422	-6.11694	0.33867
H	2.00976	-6.17072	-1.40297
H	6.33955	-1.77262	3.05179
H	5.14882	-2.46859	4.16907
H	5.25409	-3.07505	2.52247
H	5.08123	3.35300	5.16976
H	4.67499	2.16651	6.41916
H	5.09415	1.61437	4.80549
H	1.93044	-7.62246	-6.55516
H	-5.01640	-7.70773	6.84647

****

ds-G4 M06-2X/TZVP

O	-4.69776	-6.81099	6.20900
C	-5.79603	-5.95024	6.47160
C	-5.89676	-4.91569	5.37264
O	-4.78764	-4.01081	5.43209
C	-5.87042	-5.49669	3.95598
O	-6.63092	-4.61034	3.13031
C	-4.40048	-5.39273	3.57788
C	-4.06285	-4.04974	4.20454
N	-2.66865	-3.81490	4.48801
C	-1.64893	-4.70884	4.74213
N	-0.53284	-4.12815	5.07441
C	-0.82150	-2.77874	5.04963
C	-0.00944	-1.63865	5.31559
O	1.17270	-1.61267	5.66138
N	-0.72466	-0.44994	5.16026
C	-2.05771	-0.35942	4.85592
N	-2.60049	0.87721	4.87913
N	-2.81362	-1.40653	4.60777
C	-2.14398	-2.56942	4.69972
P	-7.36172	-5.16116	1.77939
O	-8.62337	-5.91449	2.13935
O	-6.39207	-5.89205	0.87779
O	-7.68020	-3.72573	1.09067
C	-8.39297	-2.71474	1.80282
C	-7.86664	-1.35345	1.39484
O	-6.50104	-1.20612	1.79463
C	-7.87977	-1.08925	-0.11252
O	-8.01772	0.32675	-0.27139
C	-6.49412	-1.53606	-0.54302
C	-5.65222	-1.10743	0.65126
N	-4.46400	-1.90407	0.84738
C	-4.31707	-3.27188	0.71023
N	-3.10904	-3.67847	0.97285
C	-2.41534	-2.53244	1.30125
C	-1.05410	-2.34203	1.67120
O	-0.17384	-3.19671	1.79342
N	-0.77095	-1.00068	1.90807
C	-1.67284	0.03018	1.82639
N	-1.20399	1.24363	2.14341
N	-2.93862	-0.13874	1.49923
C	-3.24212	-1.42208	1.23884
P	-8.35480	0.97369	-1.72996
O	-9.83841	0.91103	-2.02312
O	-7.49018	0.36796	-2.82009
O	-7.95110	2.51449	-1.42930
C	-7.14720	3.01212	-0.36013
C	-5.73943	3.34186	-0.80293
O	-4.95091	2.15113	-0.88924
C	-5.65169	4.00918	-2.18843
O	-4.65380	5.02955	-2.10126
C	-5.14143	2.88059	-3.07613
C	-4.18774	2.21674	-2.09714
N	-3.71517	0.89863	-2.41635
C	-4.40127	-0.14562	-3.00500
N	-3.73811	-1.26562	-2.97867
C	-2.55018	-0.95946	-2.33993
C	-1.45192	-1.78067	-1.94662
O	-1.31666	-3.00034	-2.07225
N	-0.45497	-1.03329	-1.32583
C	-0.52246	0.30407	-1.03301

N	0.52367	0.80775	-0.36998
N	-1.55910	1.05987	-1.33556
C	-2.52348	0.38003	-1.97846
P	-4.79102	6.41375	-2.94975
O	-5.89567	7.27560	-2.37866
O	-4.86327	6.15306	-4.43750
O	-3.31207	7.00887	-2.63091
C	-2.90285	7.20378	-1.27584
C	-1.41787	6.94337	-1.14384
O	-1.11577	5.56536	-1.40386
C	-0.53256	7.74589	-2.09644
O	0.69112	7.98325	-1.40751
C	-0.34207	6.79318	-3.26639
C	-0.33070	5.42691	-2.58243
N	-0.86111	4.36442	-3.41148
C	-2.03429	4.38646	-4.14177
N	-2.28188	3.25880	-4.73967
C	-1.22538	2.44314	-4.39637
C	-0.97929	1.07752	-4.70149
O	-1.68679	0.30498	-5.35386
N	0.22290	0.63933	-4.15445
C	1.07365	1.40141	-3.39396
N	2.21973	0.80696	-3.01937
N	0.82358	2.65105	-3.05776
C	-0.33288	3.10854	-3.57071
O	2.84099	-7.74779	-5.15396
C	3.98897	-7.51938	-5.96102
C	4.72443	-6.31056	-5.42778
O	3.95209	-5.12396	-5.65378
C	4.99183	-6.37160	-3.91948
O	6.29400	-5.81640	-3.69667
C	3.88462	-5.50988	-3.33794
C	3.64461	-4.47524	-4.42907
N	2.26755	-3.99267	-4.49875
C	1.97933	-2.63853	-4.29316
O	2.87371	-1.87159	-3.92669
N	0.71507	-2.21288	-4.52352
C	-0.24423	-3.06308	-4.88538
N	-1.44018	-2.56925	-5.18643
C	0.00825	-4.46983	-4.99780
C	1.27942	-4.87543	-4.82183
P	6.89838	-5.67945	-2.18272
O	8.37952	-5.96357	-2.25131
O	6.08177	-6.46403	-1.18468
O	6.64360	-4.09825	-1.86100
C	7.27115	-3.12024	-2.68559
C	7.26019	-1.79276	-1.96277
O	5.92780	-1.34323	-1.71864
C	7.94610	-1.80867	-0.59237
O	8.63314	-0.55946	-0.45881
C	6.78763	-2.05266	0.37569
C	5.55877	-1.51502	-0.35597
N	4.34667	-2.34649	-0.27442
C	3.12141	-1.68735	-0.50677
O	3.09704	-0.45524	-0.50452
N	2.01695	-2.42898	-0.74038
C	2.05337	-3.75649	-0.66206
N	0.98424	-4.44315	-1.05796
C	3.22850	-4.43312	-0.21137
C	4.35176	-3.69616	-0.09573
P	8.71040	0.39779	0.85579
O	9.82462	1.38266	0.59937
O	8.75322	-0.37079	2.15392
O	7.25627	1.14624	0.80010
C	6.93295	1.87668	-0.38533
C	5.82826	2.86179	-0.08515
O	4.61891	2.17677	0.24326
C	6.11156	3.80243	1.08984
O	5.52696	5.05756	0.72971
C	5.41730	3.10982	2.25609
C	4.22767	2.42485	1.58918
N	3.80418	1.15573	2.18288
C	2.42725	0.87290	2.25354
O	1.62045	1.79141	2.10776
N	2.03333	-0.40344	2.47230
C	2.92645	-1.37119	2.65327
N	2.49210	-2.62712	2.72733
C	4.32755	-1.08555	2.73076

C	4.71072	0.17157	2.43211
P	5.24973	6.29038	1.76208
O	5.50711	7.57716	1.01542
O	5.95380	6.10252	3.08348
O	3.64733	6.11158	2.03556
C	2.77172	6.17884	0.90641
C	1.33776	6.18141	1.37779
O	1.02597	4.93731	2.00848
C	1.00227	7.27100	2.39862
O	-0.33643	7.67730	2.13545
C	1.11460	6.54057	3.73175
C	0.64929	5.13384	3.36311
N	1.23456	4.06033	4.15883
C	0.41879	3.02244	4.63151
O	-0.80793	3.13356	4.56352
N	1.01025	1.92342	5.15899
C	2.33443	1.82242	5.20775
N	2.86726	0.67747	5.63652
C	3.18628	2.89709	4.80335
C	2.58946	3.96334	4.24029
H	-6.73460	-6.51328	6.50223
H	-5.66812	-5.43155	7.42580
H	-6.82154	-4.35222	5.51372
H	-6.26687	-6.51232	3.91437
H	-3.85511	-6.19727	4.06714
H	-4.38589	-3.22312	3.56519
H	-1.79692	-5.77325	4.65755
H	-0.17148	0.41966	5.26246
H	-3.49521	0.93958	4.41584
H	-1.97740	1.67895	4.75623
H	-4.24397	-5.40612	2.50351
H	-9.45801	-2.78566	1.56965
H	-8.25036	-2.83470	2.87800
H	-8.46367	-0.59271	1.90183
H	-8.69066	-1.61304	-0.62177
H	-6.48406	-2.61669	-0.66377
H	-5.31050	-0.07576	0.54500
H	-5.14293	-3.91561	0.44439
H	0.21372	-0.77283	2.13909
H	-1.83469	2.02190	2.04469
H	-0.19949	1.42699	2.19027
H	-6.17979	-1.05319	-1.46171
H	-7.62872	3.92932	-0.01854
H	-7.11276	2.29779	0.46198
H	-5.29706	4.01474	-0.06289
H	-6.60851	4.42245	-2.50864
H	-5.95972	2.19702	-3.31171
H	-3.29607	2.83376	-1.94550
H	-5.40281	-0.00290	-3.38570
H	0.40829	-1.54608	-1.06390
H	0.51975	1.79406	-0.16690
H	1.41272	0.30758	-0.32564
H	-4.64957	3.24544	-3.97323
H	-3.11997	8.23217	-0.97686
H	-3.43825	6.51952	-0.61504
H	-1.13616	7.17361	-0.11327
H	-1.00051	8.68727	-2.39206
H	-1.18997	6.87497	-3.94390
H	0.67653	5.11328	-2.30488
H	-2.66278	5.26119	-4.18711
H	0.44645	-0.36329	-4.30276
H	2.69691	1.18729	-2.21595
H	2.37895	-0.18191	-3.21652
H	0.57836	6.98062	-3.81709
H	1.30441	8.41920	-2.00973
H	4.66373	-8.38026	-5.93086
H	3.70510	-7.33006	-6.99989
H	5.67286	-6.21475	-5.95968
H	4.96441	-7.39227	-3.54001
H	2.99251	-6.11966	-3.18965
H	4.26476	-3.59015	-4.30063
H	1.59903	-5.90408	-4.94813
H	-0.77432	-5.16330	-5.26383
H	-1.61422	-1.56460	-5.12665
H	-2.21182	-3.18888	-5.36751
H	4.16813	-5.05368	-2.39552
H	8.30411	-3.40867	-2.90001
H	6.72330	-3.03008	-3.62658

H	7.74891	-1.05502	-2.60155
H	8.69282	-2.59962	-0.52094
H	6.74253	-3.12198	0.55267
H	5.25930	-0.53966	0.02024
H	5.29639	-4.17106	0.10919
H	3.25663	-5.50495	-0.08813
H	0.14418	-3.95513	-1.37852
H	0.96485	-5.44474	-0.96006
H	6.93460	-1.56224	1.33396
H	7.81250	2.41955	-0.74248
H	6.60003	1.18055	-1.16055
H	5.65506	3.45552	-0.98494
H	7.17882	3.93947	1.26729
H	6.11107	2.39168	2.68914
H	3.34319	3.06120	1.60577
H	5.75416	0.44450	2.33817
H	5.05106	-1.86311	2.92550
H	1.51641	-2.84563	2.50999
H	3.14992	-3.38331	2.82317
H	5.12018	3.81597	3.02422
H	2.96705	7.08990	0.33451
H	2.94130	5.31004	0.26389
H	0.70549	6.30200	0.49659
H	1.68641	8.11895	2.32553
H	2.15088	6.54642	4.06551
H	-0.42722	5.02937	3.46879
H	3.14708	4.77916	3.79575
H	4.25980	2.81577	4.87736
H	2.27056	-0.14700	5.74107
H	3.86631	0.55371	5.61079
H	0.48919	6.98534	4.50435
H	-0.59498	8.33104	2.79455
H	-4.59319	-7.41078	6.95386
H	2.36458	-8.50874	-5.50071

ONIOM Optimized geometries

****
ss-C4 B3LYP-D3/6-31G(d,p) mechanical embedding
****

H	17.05919	27.28177	30.47253
O	16.79772	26.78585	29.66032
C	16.97805	25.39629	29.96666
H	16.16707	24.85809	29.46846
H	16.87195	25.23663	31.05109
C	18.30829	24.79786	29.50005
H	18.49197	23.90095	30.10292
O	18.23345	24.38136	28.12928
C	19.08533	25.19031	27.31635
H	19.94132	24.60136	26.98812
N	18.32212	25.53609	26.09312
C	17.90451	26.77255	25.76887
H	18.20923	27.59188	26.40709
C	17.12779	26.99792	24.66761
H	16.79664	28.00140	24.41322
C	16.72513	25.85630	23.91484
N	15.90090	26.02824	22.81067
H	15.35002	25.20832	22.54435
H	15.29185	26.84324	22.85503
N	17.15834	24.63733	24.17319
C	18.02562	24.42468	25.22331
O	18.52476	23.33583	25.45235
C	19.51342	25.75915	29.59707
H	19.32598	26.55467	30.34000
C	19.50234	26.36823	28.18137
H	18.72265	27.13572	28.20171
H	20.46904	26.79671	27.91980
O	20.64935	25.01049	29.97093
P	22.21746	25.37357	29.45503
O	23.13478	24.84169	30.51472
O	22.28002	26.79364	28.96716
O	22.29067	24.42142	28.12010
C	22.56063	23.02144	28.25041
H	21.61974	22.48957	28.44908
H	23.24962	22.84700	29.08097

C	23.15905	22.51772	26.95274
H	23.43351	21.46537	27.06120
O	22.16447	22.66106	25.91015
C	22.71145	23.45431	24.87756
H	23.17891	22.81333	24.13057
N	21.62830	24.14628	24.16623
C	21.42911	25.47562	24.17834
H	22.05546	26.08071	24.82591
C	20.48561	26.04694	23.37001
H	20.34177	27.12458	23.35193
C	19.77576	25.18536	22.48125
N	18.97176	25.72543	21.49298
H	18.14596	25.19618	21.23934
H	18.73270	26.71754	21.61550
N	19.91496	23.87605	22.48300
C	20.84296	23.29688	23.31306
O	21.04565	22.09562	23.32936
C	24.39968	23.30721	26.46282
H	24.95231	23.77060	27.28584
C	23.75858	24.34062	25.53705
H	23.29755	25.11920	26.14678
H	24.47566	24.76788	24.83170
O	25.23988	22.50412	25.64622
P	26.39761	21.53413	26.37094
O	25.71345	20.68810	27.41353
O	27.63603	22.31752	26.67544
O	26.72733	20.61906	25.03612
C	25.90631	19.50576	24.73053
H	25.02988	19.46736	25.38380
H	26.48242	18.58077	24.88516
C	25.48548	19.53182	23.27610
H	25.03908	18.55603	23.01577
O	24.52312	20.57184	23.05561
C	24.56899	20.85481	21.66531
H	23.97854	20.13703	21.08806
N	23.94752	22.15789	21.42536
C	24.42813	23.26126	22.04301
H	25.24290	23.11529	22.74408
C	23.87913	24.48664	21.80531
H	24.26510	25.38311	22.28386
C	22.75645	24.53503	20.92710
N	22.20960	25.76321	20.56813
H	21.21974	25.75853	20.32762
H	22.35335	26.49926	21.27617
N	22.24236	23.47167	20.35303
C	22.83804	22.24201	20.52306
O	22.45942	21.25942	19.90658
C	26.63707	19.76690	22.27080
H	27.51784	20.17328	22.76756
C	26.05142	20.79078	21.28070
H	26.52365	21.76618	21.43106
H	26.19000	20.49934	20.24144
O	26.95259	18.49232	21.70838
P	28.12966	18.38204	20.56432
O	28.55583	16.94508	20.51308
O	29.10546	19.51278	20.64826
O	27.23399	18.71905	19.19622
C	26.08378	17.97138	18.88273
H	25.34050	18.02326	19.68799
H	26.32466	16.91139	18.71202
C	25.51112	18.57018	17.61230
H	24.71907	17.92872	17.20291
O	24.96429	19.87342	17.90393
C	25.54502	20.81238	17.01410
H	24.98994	20.83234	16.06735
N	25.44221	22.14576	17.57242
C	26.46439	22.77611	18.20353
H	27.40862	22.25118	18.27287
C	26.30264	24.02639	18.70829
H	27.11881	24.53402	19.21142
C	25.02139	24.64262	18.53659
N	24.83577	25.93440	18.92960
H	23.90814	26.24797	19.20549
H	25.59409	26.38027	19.45050
N	23.99242	24.02750	17.97245
C	24.13268	22.74564	17.50817
O	23.20414	22.09110	17.05431
C	26.59432	18.80015	16.53793

H	27.45327	18.13329	16.66238
C	26.94678	20.26599	16.75715
H	27.58781	20.33090	17.63616
H	27.44462	20.71428	15.89127
O	25.97707	18.59646	15.25944
H	26.41773	19.19321	14.60766

****

ss-C4 B3LYP-D3/6-31G(d,p) electrical embedding

****

H	15.76203	28.23439	29.03443
O	16.72577	28.09639	29.18278
C	16.88722	27.13347	30.22495
H	15.93374	26.63040	30.39843
H	17.16544	27.64363	31.14818
C	17.94646	26.05318	29.92831
H	18.00242	25.38740	30.79191
O	17.53152	25.30562	28.78977
C	18.55296	25.35106	27.80605
H	19.20228	24.47344	27.92830
N	17.88519	25.35712	26.46552
C	17.18120	26.44515	26.04398
H	17.15065	27.30081	26.70309
C	16.52137	26.43263	24.85798
H	15.95945	27.29433	24.52165
C	16.51922	25.19720	24.12925
N	15.83795	25.11927	22.96942
H	15.67254	24.20983	22.53560
H	15.18801	25.85486	22.73349
N	17.17886	24.12068	24.55316
C	17.89741	24.15701	25.70956
O	18.54438	23.18948	26.13270
C	19.35606	26.59813	29.65302
H	19.47235	27.61207	30.04271
C	19.40625	26.58320	28.12740
H	18.96732	27.50598	27.75668
H	20.41972	26.48546	27.74506
O	20.31046	25.72862	30.25176
P	21.88852	25.98887	30.16206
O	22.56242	25.41762	31.34587
O	22.10838	27.41187	29.84581
O	22.32501	25.10592	28.90486
C	22.30374	23.68920	29.00342
H	21.30490	23.36441	29.29671
H	23.00757	23.36858	29.77249
C	22.66637	23.00089	27.68892
H	22.71998	21.92664	27.87429
O	21.64080	23.23720	26.73593
C	22.25691	23.35900	25.47643
H	22.53290	22.36542	25.10420
N	21.34706	24.05946	24.52883
C	20.92024	25.32458	24.79405
H	21.23989	25.76439	25.72956
C	20.10870	25.98134	23.92537
H	19.75743	26.98339	24.12963
C	19.72864	25.26324	22.74898
N	18.96822	25.85370	21.79290
H	18.58542	25.19783	21.11519
H	18.38042	26.63227	22.04902
N	20.12116	24.02143	22.50754
C	20.89873	23.35079	23.39187
O	21.21427	22.16012	23.23544
C	24.00398	23.46964	27.08097
H	24.49873	24.20274	27.72283
C	23.53454	24.13281	25.78519
H	23.32581	25.18075	25.99622
H	24.25086	24.05737	24.97234
O	24.84440	22.33606	26.88456
P	26.24648	22.39196	26.09891
O	27.19083	21.49943	26.80660
O	26.61656	23.80418	25.85731
O	25.83763	21.71192	24.71257
C	25.41240	20.35421	24.67874
H	24.49565	20.24337	25.26019
H	26.18315	19.72766	25.12907
C	25.16104	19.83566	23.26009
H	24.87391	18.78974	23.33917
O	24.08969	20.54865	22.65564

C	24.51818	21.05513	21.40082
H	24.24142	20.34750	20.61233
N	23.89732	22.39216	21.17981
C	24.11146	23.38204	22.09433
H	24.72982	23.12749	22.94578
C	23.56359	24.61000	21.93347
H	23.73178	25.40711	22.64323
C	22.70203	24.77498	20.80448
N	22.09007	25.95858	20.60418
H	21.33487	25.99970	19.93659
H	22.14326	26.68429	21.31062
N	22.47330	23.80146	19.92726
C	23.07392	22.58442	20.04771
O	22.93674	21.67908	19.21252
C	26.40373	19.94346	22.36564
H	27.29523	20.16464	22.95924
C	26.04588	21.12372	21.47126
H	26.39067	22.03802	21.95285
H	26.48664	21.04132	20.48314
O	26.58752	18.73472	21.64122
P	27.83102	18.51605	20.65492
O	28.17703	17.08152	20.65539
O	28.89952	19.48429	20.96775
O	27.27044	18.90428	19.22428
C	26.22264	18.17727	18.60010
H	25.33671	18.17681	19.23781
H	26.53095	17.14430	18.43704
C	25.86186	18.81068	17.25051
H	25.09111	18.20065	16.77812
O	25.35364	20.11832	17.48172
C	26.08296	21.02635	16.67984
H	25.62180	21.05171	15.68777
N	26.00960	22.37461	17.30963
C	26.79568	22.70765	18.37075
H	27.54181	21.99662	18.69606
C	26.61932	23.88704	19.01963
H	27.22579	24.15633	19.87323
C	25.59142	24.74889	18.52498
N	25.34136	25.91897	19.15384
H	24.72494	26.56877	18.67616
H	26.09232	26.31914	19.70358
N	24.84233	24.43031	17.47403
C	24.98787	23.22939	16.85663
O	24.26109	22.89276	15.90860
C	27.07143	18.95336	16.30256
H	27.88220	18.27653	16.58179
C	27.47120	20.40890	16.49310
H	28.09295	20.49012	17.38072
H	27.98807	20.81644	15.62533
O	26.68944	18.75955	14.94906
H	27.39080	19.11275	14.35792

****
ss-C4 B3LYP-D3/TZVP mechanical embedding
****

H	16.34919	27.82630	30.07009
O	16.88697	27.19776	29.54592
C	17.05975	26.01387	30.34269
H	16.15473	25.39632	30.29641
H	17.23702	26.29118	31.38974
C	18.25013	25.20376	29.86234
H	18.43070	24.42835	30.61127
O	17.98621	24.56078	28.60780
C	18.79018	25.14916	27.56837
H	19.51358	24.41606	27.23291
N	17.93206	25.40880	26.40788
C	17.11260	26.47782	26.33801
H	17.14595	27.18726	27.15430
C	16.25498	26.63914	25.29934
H	15.61725	27.51269	25.23855
C	16.21745	25.60625	24.31794
N	15.29902	25.67983	23.30077
H	15.41283	25.05483	22.51327
H	14.97237	26.59962	23.03450
N	16.94913	24.51852	24.39486
C	17.84601	24.36341	25.42057
O	18.54113	23.37228	25.53073

C	19.53668	26.02519	29.66736
H	19.51791	26.93727	30.27332
C	19.47079	26.36771	28.17441
H	18.86916	27.26883	28.06547
H	20.45584	26.55233	27.76040
O	20.61990	25.20111	30.05091
P	22.20755	25.51440	29.59725
O	23.06906	24.91045	30.67632
O	22.34814	26.94574	29.14275
O	22.29792	24.60565	28.22114
C	22.26096	23.18119	28.30645
H	21.23069	22.84634	28.47343
H	22.87875	22.83824	29.13997
C	22.77811	22.58687	27.01483
H	22.84885	21.50317	27.13253
O	21.86194	22.87294	25.93544
C	22.53707	23.62786	24.94058
H	22.87080	22.97209	24.14422
N	21.57718	24.53903	24.30416
C	21.38664	25.80405	24.70844
H	22.00802	26.17960	25.50666
C	20.44608	26.58994	24.12251
H	20.29199	27.61598	24.43096
C	19.69664	26.02320	23.05120
N	18.72298	26.81625	22.46599
H	18.30694	26.46798	21.60320
H	19.02151	27.78377	22.33976
N	19.82376	24.77470	22.67360
C	20.74314	23.96073	23.28577
O	20.86930	22.78386	23.01295
C	24.14018	23.14166	26.56180
H	24.73777	23.49280	27.40084
C	23.72578	24.27710	25.63088
H	23.42981	25.12740	26.23738
H	24.52819	24.54982	24.94822
O	24.81947	22.16411	25.79490
P	26.39203	21.75941	26.20472
O	26.33952	20.84448	27.40567
O	27.28201	22.97641	26.15631
O	26.68070	20.87872	24.83485
C	26.04700	19.61418	24.70281
H	25.09423	19.58716	25.23497
H	26.69207	18.83238	25.12163
C	25.81377	19.32511	23.23877
H	25.56048	18.26579	23.12043
O	24.71484	20.12651	22.75238
C	25.09584	20.72523	21.51893
H	24.83972	20.09099	20.67340
N	24.33077	21.95673	21.31803
C	24.56430	23.03831	22.08901
H	25.27103	22.92551	22.89911
C	23.93010	24.21288	21.85147
H	24.12990	25.09485	22.44712
C	22.99233	24.24191	20.78105
N	22.44504	25.44681	20.40721
H	21.58435	25.46799	19.86749
H	22.52764	26.22547	21.06518
N	22.66727	23.17808	20.08638
C	23.34603	21.99950	20.27449
O	23.15179	21.02985	19.56553
C	27.02760	19.63218	22.33584
H	27.93081	19.78678	22.92463
C	26.60071	20.91412	21.62752
H	26.83984	21.74517	22.29123
H	27.09561	21.05853	20.67360
O	27.21369	18.49590	21.48725
P	27.89706	18.53597	19.98891
O	28.32103	17.10699	19.74447
O	28.84138	19.70704	19.83614
O	26.57858	18.91777	19.04576
C	25.75305	17.92376	18.46675
H	24.78686	17.90503	18.97782
H	26.21876	16.93654	18.54165
C	25.52605	18.28530	17.00822
H	24.88014	17.53606	16.54104
O	24.84952	19.54749	16.92690
C	25.70575	20.53203	16.38973
H	25.38472	20.76577	15.36742

N	25.58089	21.78653	17.13036
C	26.51144	22.27017	17.98531
H	27.39200	21.67507	18.16974
C	26.34421	23.47836	18.57826
H	27.09266	23.87055	19.25355
C	25.18072	24.21990	18.22859
N	25.05506	25.53814	18.60825
H	24.11499	25.85096	18.85027
H	25.70125	25.81715	19.35647
N	24.21338	23.71795	17.49850
C	24.33571	22.46993	16.95737
O	23.42699	21.91829	16.36316
C	26.83000	18.44619	16.21380
H	27.63065	17.83326	16.62687
C	27.11046	19.93199	16.36097
H	27.66014	20.07724	17.28454
H	27.68924	20.33910	15.52907
O	26.60078	18.06769	14.83634
H	27.19157	18.63326	14.28228

****
ss-C4 B3LYP-D3/TZVP electrical embedding
****

H	15.65489	28.11814	29.06418
O	16.63274	28.02660	29.16832
C	16.85160	27.08237	30.22043
H	15.91351	26.56208	30.42567
H	17.13793	27.61047	31.13131
C	17.92137	26.01290	29.92284
H	17.96045	25.33107	30.77562
O	17.53518	25.28478	28.76194
C	18.57993	25.34752	27.80413
H	19.22871	24.46861	27.92191
N	17.92513	25.35867	26.45907
C	17.30334	26.47620	25.99126
H	17.32497	27.35089	26.61527
C	16.64982	26.47023	24.81151
H	16.14973	27.35489	24.44689
C	16.54500	25.21446	24.13248
N	15.84102	25.14575	23.00086
H	15.66436	24.25060	22.54738
H	15.39835	25.97589	22.62649
N	17.11823	24.10925	24.60710
C	17.83421	24.13469	25.75432
O	18.39937	23.13628	26.22168
C	19.33623	26.56154	29.69042
H	19.44412	27.56811	30.10102
C	19.42930	26.57086	28.16671
H	19.00893	27.50382	27.80612
H	20.45169	26.47351	27.80668
O	20.27009	25.67876	30.29961
P	21.84962	25.93304	30.25009
O	22.49957	25.34372	31.43778
O	22.08123	27.35885	29.95690
O	22.29647	25.06567	28.98818
C	22.27937	23.64801	29.06724
H	21.28258	23.31017	29.35828
H	22.98767	23.31916	29.82884
C	22.64776	22.99271	27.73735
H	22.71420	21.91536	27.90024
O	21.61830	23.23856	26.78818
C	22.24438	23.38469	25.53627
H	22.55912	22.40001	25.17107
N	21.32363	24.04117	24.56689
C	20.92423	25.32691	24.74644
H	21.25790	25.82700	25.64293
C	20.11646	25.93397	23.84671
H	19.79441	26.95422	23.98384
C	19.70569	25.15280	22.72646
N	18.92914	25.68200	21.76077
H	18.54807	25.02306	21.08675
H	18.43745	26.54396	21.92016
N	20.08255	23.89577	22.56686
C	20.86156	23.27757	23.47744
O	21.17183	22.07999	23.38505
C	23.98116	23.48928	27.13714
H	24.47538	24.20682	27.79711
C	23.50024	24.18594	25.86313

H	23.26768	25.22095	26.10969
H	24.21832	24.15228	25.04830
O	24.82619	22.36571	26.90152
P	26.23222	22.45162	26.12421
O	27.19343	21.58319	26.83909
O	26.57541	23.86900	25.87902
O	25.85292	21.75937	24.73563
C	25.47987	20.38661	24.69799
H	24.56737	20.24008	25.27829
H	26.27329	19.78851	25.14756
C	25.24971	19.86143	23.27814
H	24.99529	18.80749	23.35627
O	24.15616	20.53985	22.67456
C	24.57818	21.07862	21.43177
H	24.33142	20.37395	20.63091
N	23.91298	22.39504	21.22733
C	24.10179	23.38309	22.14586
H	24.71845	23.13848	22.99847
C	23.53015	24.59491	21.99029
H	23.68302	25.39076	22.70110
C	22.67888	24.75380	20.85835
N	22.04987	25.92130	20.65973
H	21.34952	25.98204	19.93869
H	22.11026	26.66286	21.34650
N	22.47378	23.77906	19.98171
C	23.08954	22.57616	20.09871
O	22.96682	21.67548	19.26074
C	26.48972	20.01099	22.38410
H	27.37732	20.24098	22.98003
C	26.10269	21.19765	21.51112
H	26.41591	22.11206	22.01352
H	26.55229	21.15012	20.52424
O	26.69910	18.82054	21.63630
P	27.94188	18.64743	20.63761
O	28.33244	17.22360	20.63072
O	28.98055	19.65033	20.94386
O	27.35063	19.02340	19.21458
C	26.33204	18.25405	18.59206
H	25.44729	18.22045	19.23055
H	26.68084	17.23352	18.43215
C	25.94478	18.86617	17.23962
H	25.19058	18.22884	16.77671
O	25.39662	20.15864	17.46462
C	26.09996	21.08478	16.66011
H	25.63300	21.09919	15.67064
N	25.99787	22.43051	17.28879
C	26.78118	22.77968	18.34274
H	27.53436	22.08094	18.66986
C	26.59084	23.95437	18.98587
H	27.19474	24.23218	19.83529
C	25.54817	24.79588	18.50022
N	25.27620	25.95623	19.12931
H	24.63082	26.59056	18.67079
H	26.02068	26.37370	19.67347
N	24.80407	24.45849	17.45710
C	24.96131	23.26373	16.84406
O	24.22490	22.91318	15.91215
C	27.14307	19.03957	16.28197
H	27.97378	18.38480	16.55526
C	27.50468	20.50517	16.46738
H	28.13073	20.60269	17.35086
H	28.00625	20.92594	15.59665
O	26.75687	18.83605	14.93112
H	27.42679	19.23728	14.33475

****
ss-C4 M06-2X/6-31G(d,p) mechanical embedding
****

H	16.77931	27.71398	30.36377
O	17.21435	27.05995	29.77988
C	17.35991	25.85883	30.52754
H	16.40513	25.31407	30.57054
H	17.67039	26.08383	31.55886
C	18.42219	24.98400	29.89066
H	18.61054	24.13877	30.56299
O	17.96186	24.49076	28.63426
C	18.81846	24.93251	27.58194

H	19.47725	24.13149	27.24851
N	17.97479	25.27760	26.43281
C	17.02583	26.23041	26.56041
H	16.93063	26.69985	27.53916
C	16.23262	26.56811	25.51243
H	15.47627	27.34135	25.60344
C	16.43454	25.85218	24.28903
N	15.63442	26.13878	23.21329
H	16.00906	25.94320	22.29076
H	15.14441	27.02816	23.23466
N	17.28963	24.86173	24.15888
C	18.11259	24.53474	25.21826
O	18.92415	23.63228	25.14395
C	19.75571	25.71469	29.62758
H	19.84383	26.60879	30.26241
C	19.60098	26.10611	28.15335
H	18.99865	27.02027	28.10505
H	20.56143	26.27073	27.66933
O	20.78510	24.79813	29.90427
P	22.29480	24.95614	29.20890
O	23.24712	24.18443	30.05027
O	22.51935	26.38345	28.82962
O	22.00157	24.14710	27.81385
C	22.16168	22.73159	27.78622
H	21.17454	22.25376	27.86217
H	22.79886	22.40524	28.61290
C	22.81075	22.38051	26.46930
H	23.06183	21.31629	26.43395
O	21.89314	22.70794	25.41523
C	22.52056	23.58819	24.52472
H	22.98873	23.03009	23.71310
N	21.50856	24.42637	23.87158
C	21.15010	25.64186	24.31271
H	21.67380	26.04490	25.17133
C	20.18634	26.36381	23.67638
H	19.91039	27.36334	23.99889
C	19.63697	25.78574	22.49550
N	18.71249	26.52263	21.75804
H	18.65207	26.23482	20.78512
H	18.94068	27.52484	21.78196
N	19.88509	24.56242	22.10449
C	20.78332	23.79592	22.81728
O	20.97497	22.62329	22.57163
C	24.08578	23.21602	26.21457
H	24.50914	23.59495	27.15129
C	23.56488	24.34329	25.33310
H	23.11572	25.09532	25.98232
H	24.35177	24.78534	24.71591
O	25.03762	22.48380	25.46769
P	26.14748	21.68088	26.40708
O	25.39354	20.83850	27.39019
O	27.23054	22.61263	26.82309
O	26.74079	20.72478	25.21221
C	26.01995	19.55863	24.87486
H	25.06141	19.51565	25.40069
H	26.60756	18.67766	25.17182
C	25.81097	19.50291	23.37838
H	25.51076	18.48599	23.07943
O	24.79282	20.43668	23.00440
C	25.16708	20.94093	21.73608
H	24.93517	20.23481	20.93608
N	24.41067	22.14853	21.41570
C	24.67345	23.31256	22.04572
H	25.36643	23.27645	22.88193
C	24.06343	24.46682	21.66933
H	24.27387	25.40614	22.17117
C	23.06675	24.36852	20.64857
N	22.43500	25.51863	20.21700
H	21.50736	25.42114	19.80902
H	22.46231	26.30023	20.88340
N	22.74194	23.24157	20.06829
C	23.45809	22.09585	20.34806
O	23.29850	21.07531	19.70770
C	27.09582	19.86250	22.57722
H	27.93117	20.05427	23.25523
C	26.67183	21.14375	21.85965
H	26.87672	21.96862	22.54960
H	27.18853	21.30965	20.91628

O	27.44310	18.74946	21.76215
P	27.82294	18.80516	20.18071
O	28.25923	17.41605	19.85025
O	28.67610	19.99616	19.85293
O	26.34916	19.12951	19.51466
C	25.54467	18.10396	18.97124
H	24.56733	18.12119	19.46778
H	26.01235	17.12233	19.11047
C	25.33576	18.39167	17.49025
H	24.66808	17.63716	17.05259
O	24.72333	19.67178	17.36024
C	25.62136	20.58214	16.78248
H	25.33592	20.74850	15.72861
N	25.50562	21.88817	17.40943
C	26.37382	22.35714	18.33992
H	27.18521	21.70597	18.65094
C	26.23523	23.61536	18.83169
H	26.93467	24.02264	19.55298
C	25.18258	24.40901	18.27568
N	25.15398	25.76448	18.51688
H	24.22058	26.17981	18.54874
H	25.69615	26.06472	19.33450
N	24.26168	23.92894	17.47747
C	24.31993	22.61365	17.09225
O	23.41245	22.06590	16.49530
C	26.63733	18.47704	16.67701
H	27.40064	17.79250	17.05362
C	27.01288	19.93911	16.84985
H	27.52287	20.07678	17.80108
H	27.67686	20.28358	16.05071
O	26.38009	18.17945	15.29968
H	26.48238	19.02315	14.79146

****
ss-C4 M06-2X/6-31G(d,p) electrical embedding
****

H	15.85734	28.42158	29.06989
O	16.83150	28.28351	29.15605
C	17.02464	27.32374	30.19889
H	16.05885	26.88702	30.46130
H	17.41399	27.82104	31.08872
C	17.97643	26.17046	29.83210
H	17.99991	25.46702	30.66728
O	17.46945	25.50688	28.68163
C	18.51792	25.34290	27.74224
H	19.04564	24.40344	27.94658
N	17.90542	25.35679	26.37726
C	17.17939	26.43054	25.96412
H	17.13903	27.27530	26.63850
C	16.51868	26.41981	24.78511
H	15.93929	27.27138	24.45570
C	16.51805	25.17565	24.06653
N	15.76167	25.06539	22.96487
H	15.61572	24.15484	22.52908
H	15.07419	25.77687	22.76730
N	17.22878	24.12802	24.46083
C	17.98655	24.19274	25.58790
O	18.72002	23.27191	25.95428
C	19.41833	26.60964	29.54022
H	19.58836	27.63944	29.86271
C	19.48979	26.49145	28.01986
H	19.15008	27.42294	27.57527
H	20.49426	26.26380	27.67500
O	20.31574	25.72768	30.20676
P	21.90414	25.94885	30.19265
O	22.50883	25.35150	31.40039
O	22.17434	27.36951	29.90745
O	22.38566	25.06771	28.95158
C	22.35031	23.65072	29.04174
H	21.34388	23.33226	29.31725
H	23.04010	23.32034	29.81936
C	22.72750	22.96902	27.72775
H	22.78439	21.89414	27.90807
O	21.70719	23.20623	26.76942
C	22.33075	23.35304	25.51593
H	22.62112	22.36713	25.13499
N	21.42199	24.05191	24.56467

C	21.01674	25.32574	24.80904
H	21.36163	25.78233	25.72669
C	20.20299	25.98097	23.94810
H	19.86718	26.99148	24.13736
C	19.77915	25.24038	22.79682
N	18.97227	25.79835	21.88608
H	18.68766	25.18950	21.12390
H	18.71074	26.77808	21.89632
N	20.15228	23.98874	22.57792
C	20.95533	23.33754	23.44773
O	21.29509	22.15774	23.28144
C	24.06675	23.44728	27.13123
H	24.56133	24.16866	27.78625
C	23.59866	24.13358	25.84731
H	23.38185	25.17431	26.08228
H	24.31851	24.08471	25.03592
O	24.90610	22.31646	26.91795
P	26.29663	22.37917	26.11402
O	27.24732	21.47473	26.79719
O	26.66763	23.79365	25.88819
O	25.86085	21.71912	24.72543
C	25.42600	20.36443	24.68120
H	24.51117	20.25394	25.26574
H	26.19462	19.72939	25.12341
C	25.16689	19.85813	23.25901
H	24.89121	18.80861	23.32939
O	24.08352	20.56480	22.66792
C	24.49712	21.08681	21.41356
H	24.22785	20.38141	20.62054
N	23.86249	22.41949	21.20429
C	24.03193	23.38411	22.15149
H	24.62467	23.10746	23.01508
C	23.48449	24.60931	22.00454
H	23.61495	25.39098	22.73954
C	22.66788	24.79581	20.84338
N	22.05865	25.97730	20.66302
H	21.39951	26.08559	19.90681
H	22.12108	26.70167	21.37089
N	22.48490	23.85029	19.93289
C	23.08946	22.63554	20.05093
O	22.99199	21.75306	19.19336
C	26.40102	19.98836	22.35651
H	27.29583	20.20911	22.94521
C	26.02305	21.17787	21.48345
H	26.35161	22.09052	21.98009
H	26.46456	21.11758	20.49473
O	26.58858	18.79227	21.61208
P	27.82381	18.60068	20.60981
O	28.19680	17.17277	20.60049
O	28.87752	19.58776	20.91429
O	27.24034	18.98339	19.18618
C	26.20613	18.23269	18.56666
H	25.31796	18.22820	19.20129
H	26.52937	17.20193	18.42064
C	25.83956	18.83662	17.20471
H	25.07958	18.20594	16.74274
O	25.30824	20.13762	17.41300
C	26.04829	21.05420	16.63066
H	25.59551	21.09425	15.63539
N	25.96442	22.38974	17.28459
C	26.73751	22.70276	18.35840
H	27.48900	21.99052	18.66923
C	26.54626	23.85862	19.03619
H	27.14321	24.11557	19.89987
C	25.50706	24.71693	18.55687
N	25.24440	25.86617	19.21104
H	24.62863	26.51803	18.73548
H	25.99753	26.26583	19.76044
N	24.77081	24.41643	17.49936
C	24.94187	23.23864	16.84955
O	24.23248	22.92549	15.88682
C	27.04644	18.98602	16.25464
H	27.86009	18.31182	16.53076
C	27.43980	20.44292	16.44482
H	28.06328	20.52664	17.33045
H	27.95502	20.85299	15.57731
O	26.66310	18.79396	14.90151
H	27.34681	19.17923	14.30995

****
ss-C4 M06-2X/TZVP mechanical embedding
****

H	16.68567	27.80557	30.10660
O	16.77895	27.10477	29.43708
C	17.11253	25.90272	30.13885
H	16.25178	25.22961	30.12021
H	17.35330	26.12899	31.18310
C	18.30814	25.22086	29.51381
H	18.62698	24.41671	30.17963
O	17.95733	24.65463	28.25544
C	18.69038	25.25957	27.19447
H	19.41927	24.55725	26.80090
N	17.75161	25.51525	26.09625
C	16.82449	26.49002	26.19279
H	16.87717	27.13783	27.06039
C	15.84676	26.63288	25.26749
H	15.11384	27.42527	25.33941
C	15.78542	25.64130	24.24088
N	14.81691	25.73323	23.27499
H	14.56698	24.89081	22.76631
H	14.01985	26.34275	23.42481
N	16.62336	24.64237	24.16475
C	17.65849	24.52584	25.06386
O	18.43817	23.60193	25.03231
C	19.51093	26.14937	29.26804
H	19.43615	27.05919	29.87615
C	19.36383	26.49144	27.77997
H	18.72373	27.37152	27.70798
H	20.32055	26.70744	27.31243
O	20.65637	25.42335	29.63806
P	22.18456	25.65580	29.02991
O	23.12671	25.18514	30.09796
O	22.29418	27.00724	28.38542
O	22.14178	24.54971	27.81169
C	21.97430	23.18686	28.19450
H	20.91978	22.90961	28.08599
H	22.27094	23.06033	29.23733
C	22.82351	22.28080	27.33471
H	22.80436	21.28014	27.77468
O	22.31049	22.17633	26.00878
C	23.08586	22.94358	25.10775
H	23.47166	22.28203	24.33380
N	22.17973	23.87278	24.39914
C	21.90908	25.12365	24.80281
H	22.44500	25.51451	25.65574
C	20.97524	25.88207	24.16775
H	20.77297	26.90066	24.47550
C	20.28012	25.27352	23.08415
N	19.24087	25.94651	22.48700
H	18.89397	25.57280	21.60800
H	19.27039	26.95820	22.51548
N	20.50426	24.05030	22.68686
C	21.46947	23.29723	23.29776
O	21.70972	22.15771	22.96491
C	24.27964	22.73060	27.17326
H	24.63162	23.31295	28.02703
C	24.22155	23.58472	25.90233
H	24.01498	24.60970	26.19490
H	25.16773	23.55680	25.36575
O	25.02732	21.54275	27.03733
P	26.59221	21.46102	26.51068
O	27.13161	20.23801	27.18737
O	27.24883	22.80594	26.56816
O	26.32584	21.12137	24.91149
C	25.79321	19.83728	24.63468
H	24.78952	19.74295	25.06390
H	26.43489	19.06609	25.07287
C	25.71719	19.62817	23.14288
H	25.46362	18.58088	22.94990
O	24.69948	20.46806	22.58316
C	25.24561	21.19231	21.49662
H	25.10216	20.65804	20.55890
N	24.51170	22.44327	21.33580
C	24.73763	23.51379	22.12062
H	25.49380	23.41273	22.88940
C	24.05293	24.67185	21.94830

H	24.23721	25.54399	22.56408
C	23.07724	24.68261	20.90722
N	22.44559	25.85831	20.57019
H	21.58106	25.78270	20.04873
H	22.38986	26.58878	21.28259
N	22.78211	23.63463	20.19160
C	23.46038	22.45729	20.37128
O	23.21303	21.46230	19.72862
C	27.02690	19.94626	22.39036
H	27.87844	19.98041	23.07129
C	26.72983	21.32091	21.79797
H	26.91923	22.04228	22.59372
H	27.33146	21.55003	20.92148
O	27.23870	18.89122	21.45494
P	27.80075	18.97359	19.92389
O	28.23320	17.56921	19.60874
O	28.70215	20.15756	19.70420
O	26.42207	19.34553	19.08437
C	25.48533	18.34840	18.72539
H	24.55104	18.52051	19.26706
H	25.87250	17.35135	18.95656
C	25.21274	18.45521	17.23578
H	24.53104	17.65490	16.93014
O	24.59263	19.69930	16.92236
C	25.51986	20.60592	16.37574
H	25.17420	20.87249	15.36890
N	25.50862	21.87074	17.11299
C	26.44496	22.26778	18.00923
H	27.27601	21.60648	18.21759
C	26.34432	23.47333	18.62005
H	27.10754	23.81276	19.30711
C	25.24426	24.30290	18.24334
N	25.20314	25.61238	18.64981
H	24.28618	26.04238	18.72742
H	25.79938	25.86834	19.42851
N	24.26591	23.88260	17.48673
C	24.31304	22.62901	16.94306
O	23.38384	22.14052	16.33860
C	26.46901	18.41639	16.36624
H	27.24256	17.79380	16.80561
C	26.87421	19.88405	16.28780
H	27.54726	20.11242	17.10721
H	27.38028	20.10509	15.34436
O	26.17346	17.86289	15.08143
H	25.96731	18.58288	14.44559

****
ss-C4 M06-2X/TZVP electrical embedding
****

H	15.66605	28.31330	29.01810
O	16.64312	28.22740	29.13276
C	16.85442	27.31617	30.21505
H	15.90047	26.85430	30.47841
H	17.20710	27.86252	31.09054
C	17.85648	26.18506	29.91678
H	17.89908	25.52987	30.78902
O	17.38616	25.44081	28.80057
C	18.42282	25.34735	27.83843
H	19.01208	24.44038	28.02942
N	17.78757	25.32517	26.48419
C	17.03689	26.37361	26.06085
H	16.96586	27.21852	26.72745
C	16.37965	26.32764	24.88411
H	15.77629	27.15475	24.54204
C	16.46490	25.09695	24.16092
N	15.72347	24.95948	23.02489
H	15.57350	24.00854	22.67607
H	14.90516	25.54938	22.96435
N	17.21438	24.09252	24.54382
C	17.88738	24.15016	25.72005
O	18.54725	23.21055	26.16084
C	19.28378	26.66180	29.61144
H	19.43352	27.69373	29.93684
C	19.33598	26.54875	28.09110
H	18.93900	27.46112	27.65513
H	20.34359	26.37535	27.72311
O	20.21009	25.79789	30.26134
P	21.79487	26.02678	30.18720

O	22.44799	25.45976	31.38384
O	22.04746	27.44554	29.87242
O	22.25560	25.13852	28.94500
C	22.28810	23.72412	29.07330
H	21.30821	23.37496	29.40092
H	23.02333	23.44657	29.83015
C	22.63816	23.00881	27.76968
H	22.67334	21.93788	27.97567
O	21.60996	23.25144	26.81983
C	22.23004	23.42361	25.56847
H	22.52395	22.44281	25.17756
N	21.31725	24.12052	24.61784
C	20.91481	25.39403	24.84626
H	21.25108	25.85514	25.76307
C	20.12023	26.04214	23.96602
H	19.79070	27.05552	24.13786
C	19.74261	25.31358	22.79364
N	18.98345	25.89716	21.85265
H	18.85359	25.38490	20.98485
H	18.83803	26.90102	21.82364
N	20.10313	24.05938	22.59822
C	20.86024	23.39270	23.50296
O	21.16381	22.21157	23.37479
C	23.98283	23.43839	27.14693
H	24.53769	24.10440	27.81296
C	23.50292	24.19346	25.90690
H	23.28721	25.22249	26.19213
H	24.21415	24.17642	25.08581
O	24.74858	22.26920	26.85208
P	26.15168	22.29586	26.06334
O	27.07955	21.36276	26.74084
O	26.56557	23.69682	25.84644
O	25.73088	21.66279	24.65891
C	25.29885	20.31004	24.57601
H	24.36749	20.18885	25.13174
H	26.05475	19.66538	25.02552
C	25.08383	19.83813	23.13397
H	24.80797	18.78663	23.17010
O	24.01740	20.56329	22.53274
C	24.47761	21.16491	21.33004
H	24.22804	20.51248	20.48659
N	23.86249	22.51576	21.17000
C	23.96333	23.41582	22.18438
H	24.49062	23.08393	23.06775
C	23.42721	24.64651	22.07515
H	23.50384	25.37831	22.86410
C	22.70067	24.91521	20.87470
N	22.08875	26.09824	20.73926
H	21.58245	26.31606	19.89406
H	22.20919	26.82149	21.43968
N	22.59782	24.03746	19.89127
C	23.19591	22.82045	19.97193
O	23.18825	22.00668	19.04991
C	26.34276	19.99124	22.26889
H	27.22651	20.16467	22.88918
C	26.00124	21.23371	21.45668
H	26.31526	22.11505	22.01621
H	26.47899	21.22352	20.48411
O	26.52862	18.83061	21.46594
P	27.78651	18.65672	20.48411
O	28.19195	17.23628	20.49196
O	28.81390	19.67164	20.78853
O	27.21455	19.01040	19.04650
C	26.24917	18.19366	18.39896
H	25.33931	18.15298	19.00042
H	26.62976	17.17841	18.29123
C	25.90392	18.73886	17.00583
H	25.17872	18.06462	16.54857
O	25.32457	20.02860	17.15670
C	26.12009	20.96599	16.45019
H	25.70699	21.06005	15.44159
N	26.04286	22.27963	17.15001
C	26.81133	22.55285	18.23594
H	27.51900	21.80478	18.55833
C	26.65908	23.70803	18.91717
H	27.25025	23.93080	19.79177
C	25.66571	24.61084	18.43452
N	25.44341	25.76908	19.08443

H	24.84849	26.43858	18.60636
H	26.21296	26.15286	19.62302
N	24.93329	24.34192	17.37101
C	25.05700	23.16517	16.72152
O	24.33337	22.88700	15.75922
C	27.12468	18.89248	16.07653
H	27.93389	18.21736	16.36268
C	27.51217	20.34808	16.27649
H	28.13201	20.42688	17.16442
H	28.03730	20.76217	15.41668
O	26.76209	18.70411	14.71746
H	27.44084	19.11403	14.13715

Structures from the MD simulation of ss-C_6

```
*****************************************************************************
Starting structure of ss-C6 used in MD simulation, Figure 2 in the main text
*****************************************************************************
```

H	-3.360000	-8.979000	-3.256000
O	-3.152000	-8.520000	-2.439000
C	-3.142000	-9.254000	-1.217000
H	-2.370000	-10.013000	-1.348000
H	-4.103000	-9.740000	-1.047000
C	-2.795000	-8.387000	-0.006000
H	-3.048000	-8.979000	0.874000
O	-1.362000	-8.199000	0.083000
C	-1.136000	-6.940000	0.748000
H	-0.479000	-7.229000	1.568000
N	-0.303000	-6.068000	-0.070000
C	-0.395000	-6.077000	-1.444000
H	-0.997000	-6.824000	-1.940000
C	0.249000	-5.176000	-2.180000
H	0.193000	-5.198000	-3.268000
C	0.999000	-4.151000	-1.433000
N	1.574000	-3.111000	-2.118000
H	2.083000	-2.421000	-1.584000
H	1.483000	-3.051000	-3.122000
N	1.128000	-4.159000	-0.142000
C	0.474000	-5.116000	0.599000
O	0.572000	-5.122000	1.827000
C	-3.400000	-6.987000	0.040000
H	-3.351000	-6.429000	-0.895000
C	-2.498000	-6.334000	1.065000
H	-2.877000	-5.340000	1.303000
H	-2.477000	-6.941000	1.970000
O	-4.733000	-7.008000	0.494000
P	-5.919000	-6.869000	-0.606000
O	-5.468000	-7.568000	-1.874000
O	-7.204000	-7.266000	0.063000
O	-5.879000	-5.277000	-0.889000
C	-6.286000	-4.402000	0.155000
H	-6.077000	-4.929000	1.086000
H	-7.343000	-4.163000	0.038000
C	-5.482000	-3.098000	0.123000
H	-5.518000	-2.681000	1.129000
O	-4.078000	-3.395000	-0.097000
C	-3.490000	-2.274000	-0.772000
H	-2.703000	-2.054000	-0.050000
N	-2.758000	-2.687000	-1.965000
C	-3.308000	-3.587000	-2.850000
H	-4.200000	-4.132000	-2.579000
C	-2.758000	-3.801000	-4.044000
H	-3.187000	-4.526000	-4.736000
C	-1.551000	-3.014000	-4.349000
N	-0.948000	-3.132000	-5.574000
H	-0.122000	-2.576000	-5.746000
H	-1.328000	-3.757000	-6.270000
N	-0.997000	-2.197000	-3.507000
C	-1.611000	-1.960000	-2.294000
O	-1.125000	-1.126000	-1.532000
C	-5.877000	-2.076000	-0.940000
H	-6.156000	-2.505000	-1.902000

C	-4.593000	-1.283000	-1.093000
H	-4.730000	-0.511000	-1.850000
H	-4.338000	-0.817000	-0.141000
O	-6.856000	-1.162000	-0.483000
P	-8.325000	-1.110000	-1.163000
O	-8.926000	-2.487000	-1.061000
O	-9.040000	0.096000	-0.607000
O	-7.959000	-0.841000	-2.717000
C	-7.282000	0.348000	-3.089000
H	-6.470000	0.501000	-2.378000
H	-7.981000	1.184000	-3.075000
C	-6.694000	0.210000	-4.506000
H	-5.917000	0.958000	-4.662000
O	-5.997000	-1.070000	-4.617000
C	-6.662000	-1.830000	-5.645000
H	-6.180000	-1.775000	-6.621000
N	-6.619000	-3.254000	-5.318000
C	-7.317000	-3.750000	-4.237000
H	-7.957000	-3.090000	-3.670000
C	-7.222000	-5.027000	-3.873000
H	-7.775000	-5.407000	-3.014000
C	-6.327000	-5.865000	-4.687000
N	-6.161000	-7.188000	-4.376000
H	-5.539000	-7.732000	-4.957000
H	-6.650000	-7.592000	-3.590000
N	-5.659000	-5.410000	-5.704000
C	-5.757000	-4.079000	-6.039000
O	-5.053000	-3.650000	-6.955000
C	-7.753000	0.240000	-5.618000
H	-8.677000	0.760000	-5.367000
C	-8.045000	-1.226000	-5.843000
H	-9.057000	-1.451000	-5.507000
H	-7.956000	-1.456000	-6.905000
O	-7.195000	0.752000	-6.834000
P	-7.330000	2.360000	-7.053000
O	-8.778000	2.681000	-7.311000
O	-6.559000	3.034000	-5.946000
O	-6.503000	2.508000	-8.447000
C	-6.206000	3.843000	-8.843000
H	-5.821000	4.335000	-7.950000
H	-7.112000	4.323000	-9.213000
C	-5.149000	3.921000	-9.959000
H	-4.844000	4.961000	-10.077000
O	-3.938000	3.232000	-9.532000
C	-3.747000	2.092000	-10.393000
H	-2.839000	2.374000	-10.927000
N	-3.348000	0.904000	-9.635000
C	-3.757000	0.737000	-8.329000
H	-4.261000	1.546000	-7.821000
C	-3.545000	-0.403000	-7.676000
H	-3.865000	-0.530000	-6.642000
C	-2.887000	-1.468000	-8.450000
N	-2.723000	-2.715000	-7.893000
H	-2.267000	-3.422000	-8.452000
H	-3.052000	-2.902000	-6.957000
N	-2.460000	-1.315000	-9.665000
C	-2.688000	-0.121000	-10.316000
O	-2.316000	0.000000	-11.484000
C	-5.570000	3.293000	-11.286000
H	-6.640000	3.264000	-11.492000
C	-5.042000	1.885000	-11.171000
H	-5.877000	1.189000	-11.092000
H	-4.451000	1.645000	-12.055000
O	-4.868000	3.937000	-12.359000
P	-5.782000	4.797000	-13.395000
O	-6.557000	3.814000	-14.235000
O	-6.456000	5.902000	-12.623000
O	-4.612000	5.436000	-14.329000
C	-3.938000	6.580000	-13.832000
H	-3.747000	6.373000	-12.779000
H	-4.565000	7.461000	-13.972000
C	-2.601000	6.831000	-14.549000
H	-2.160000	7.739000	-14.138000
O	-1.667000	5.778000	-14.189000
C	-2.284000	5.459000	-16.477000
H	-1.441000	6.051000	-16.834000
N	-1.199000	3.646000	-15.101000
C	-2.295000	2.999000	-14.574000

```
H    -3.219000     3.544000   -14.448000
C    -2.247000     1.722000   -14.216000
H    -3.129000     1.208000   -13.834000
C    -0.933000     1.076000   -14.340000
N    -0.774000    -0.190000   -13.838000
H     0.135000    -0.621000   -13.931000
H    -1.548000    -0.662000   -13.394000
N     0.099000     1.652000   -14.878000
C    -0.006000     2.956000   -15.320000
O     0.959000     3.470000   -15.890000
C    -2.666000     6.868000   -16.079000
H    -3.362000     6.517000   -16.841000
C    -1.287000     5.078000   -15.388000
H    -0.319000     5.466000   -15.071000
H    -1.759000     4.556000   -14.556000
O    -1.644000     7.752000   -16.558000
P    -2.050000     8.777000   -17.749000
O    -3.046000     9.780000   -17.231000
O    -2.276000     7.986000   -19.010000
O    -0.627000     9.542000   -17.924000
C    -0.246000    10.443000   -16.899000
H    -0.342000     9.919000   -15.948000
H    -0.888000    11.323000   -16.931000
C     1.211000    10.882000   -17.087000
H     1.446000    11.695000   -16.401000
O     2.090000     9.805000   -16.687000
C     2.847000     9.375000   -17.831000
H     3.872000     9.693000   -17.642000
N     2.934000     7.922000   -17.845000
C     1.784000     7.171000   -17.764000
H     0.828000     7.667000   -17.678000
C     1.821000     5.845000   -17.789000
H     0.905000     5.255000   -17.790000
C     3.163000     5.244000   -17.782000
N     3.267000     3.889000   -17.602000
H     4.193000     3.486000   -17.599000
H     2.437000     3.327000   -17.478000
N     4.256000     5.936000   -17.911000
C     4.176000     7.310000   -18.018000
O     5.189000     7.972000   -18.254000
C     1.567000    11.232000   -18.534000
H     0.711000    11.506000   -19.151000
C     2.192000     9.967000   -19.073000
H     1.586000     9.583000   -19.894000
H     3.198000    10.183000   -19.434000
O     2.555000    12.276000   -18.565000
H     2.829000    12.549000   -19.444000
```

****
Representative structure of ss-C6 form MD simulations, Figure 2 in the main text
****

```
H      14.724   13.365   26.322
O      14.569   13.280   25.379
C      13.246   12.782   25.189
H      12.937   12.059   25.944
H      13.136   12.326   24.205
C      12.247   13.919   25.314
H      11.362   13.571   24.782
O      11.875   14.234   26.597
C      11.086   15.387   26.480
H      10.066   15.089   26.239
N      11.028   16.156   27.759
C      12.176   16.384   28.491
H      12.967   15.649   28.458
C      12.177   17.376   29.404
H      13.015   17.426   30.083
C      11.009   18.160   29.573
N      10.958   19.009   30.574
H      10.090   19.478   30.795
H      11.720   18.982   31.236
N       9.943   18.043   28.814
C       9.981   17.086   27.841
O       9.116   17.153   26.915
C      12.608   15.231   24.653
H      13.653   15.453   24.872
C      11.706   16.246   25.364
H      12.279   17.102   25.720
```

H	10.932	16.534	24.653
O	12.388	15.216	23.274
P	13.358	15.992	22.322
O	14.755	15.902	22.818
O	13.025	15.516	20.940
O	13.016	17.545	22.497
C	11.746	18.084	22.056
H	10.957	17.543	22.579
H	11.677	17.863	20.991
C	11.465	19.573	22.316
H	10.585	19.833	21.728
O	11.164	19.835	23.675
C	11.968	20.857	24.162
H	11.521	21.808	23.870
N	12.349	20.901	25.578
C	13.177	19.925	26.078
H	13.625	19.136	25.492
C	13.344	19.904	27.402
H	13.903	19.086	27.833
C	12.751	20.846	28.227
N	13.153	20.835	29.409
H	12.769	21.562	29.996
H	13.918	20.240	29.694
N	11.980	21.878	27.768
C	11.688	21.816	26.428
O	10.851	22.600	26.053
C	12.622	20.501	21.937
H	13.285	20.023	21.215
C	13.305	20.633	23.279
H	13.911	19.755	23.502
H	13.943	21.493	23.482
O	12.056	21.725	21.445
P	12.861	22.977	21.102
O	14.228	22.596	20.587
O	12.051	23.939	20.309
O	13.116	23.592	22.527
C	12.271	24.566	23.122
H	11.418	24.046	23.557
H	11.782	25.201	22.383
C	12.998	25.360	24.169
H	12.303	26.137	24.489
O	13.316	24.549	25.317
C	14.726	24.585	25.473
H	14.897	25.363	26.218
N	15.329	23.355	26.050
C	16.030	22.411	25.312
H	16.056	22.520	24.238
C	16.543	21.333	25.879
H	16.863	20.487	25.289
C	16.448	21.257	27.279
N	16.921	20.132	27.850
H	16.654	19.976	28.812
H	17.528	19.564	27.276
N	15.785	22.030	28.028
C	15.265	23.138	27.443
O	14.817	24.003	28.239
C	14.249	26.138	23.661
H	14.326	26.127	22.574
C	15.264	25.157	24.144
H	15.338	24.359	23.405
H	16.235	25.635	24.265
O	14.370	27.423	24.342
P	15.645	28.387	24.209
O	16.176	28.182	22.877
O	15.135	29.746	24.547
O	16.628	27.906	25.305
C	16.236	27.994	26.607
H	15.305	27.501	26.886
H	16.136	29.047	26.871
C	17.314	27.274	27.446
H	17.098	27.426	28.503
O	17.356	25.940	27.261
C	18.730	25.648	27.293
H	19.041	25.754	28.332
N	18.998	24.305	26.784
C	18.928	23.979	25.407
H	18.622	24.756	24.723
C	19.263	22.799	24.952

H	19.210	22.578	23.896
C	19.597	21.844	25.938
N	19.886	20.573	25.700
H	20.120	20.002	26.499
H	19.920	20.231	24.751
N	19.704	22.009	27.208
C	19.385	23.252	27.665
O	19.472	23.412	28.881
C	18.694	27.870	27.337
H	18.618	28.775	26.734
C	19.360	26.786	26.558
H	19.000	26.886	25.534
H	20.448	26.744	26.601
O	19.231	28.134	28.604
P	20.619	28.896	28.944
O	20.726	30.104	28.109
O	20.695	28.952	30.402
O	21.691	27.832	28.541
C	23.072	28.127	28.404
H	23.626	28.294	29.328
H	23.200	28.999	27.764
C	23.946	27.051	27.772
H	24.985	27.344	27.622
O	23.984	25.932	28.600
C	22.992	25.015	28.142
H	22.030	25.413	28.466
N	23.148	23.725	28.725
C	22.418	23.439	29.846
H	21.482	23.890	30.140
C	22.767	22.398	30.679
H	22.118	22.065	31.475
C	23.846	21.555	30.182
N	24.203	20.480	30.850
H	25.120	20.060	30.791
H	23.672	20.243	31.676
N	24.457	21.727	29.110
C	24.150	22.835	28.384
O	24.897	23.015	27.440
C	23.265	26.582	26.459
H	22.392	27.221	26.329
C	22.854	25.082	26.622
H	21.837	24.899	26.276
H	23.636	24.474	26.165
O	24.239	26.645	25.389
P	24.746	28.080	24.763
O	23.498	28.872	24.808
O	25.483	27.871	23.540
O	25.728	28.672	25.894
C	25.822	30.048	26.263
H	24.868	30.392	26.662
H	26.173	30.715	25.475
C	26.829	30.262	27.411
H	26.843	31.333	27.611
O	26.357	29.553	28.537
C	27.487	28.929	29.096
H	27.993	29.704	29.672
N	26.973	27.791	29.908
C	27.280	26.488	29.656
H	28.031	26.300	28.903
C	26.719	25.437	30.331
H	26.939	24.412	30.070
C	25.941	25.858	31.438
N	25.286	25.044	32.159
H	24.530	25.507	32.643
H	25.140	24.082	31.888
N	25.582	27.078	31.703
C	26.151	28.065	30.996
O	25.869	29.206	31.269
C	28.278	29.719	27.119
H	28.343	29.432	26.069
C	28.334	28.508	27.950
H	27.740	27.809	27.362
H	29.358	28.254	28.225
O	29.388	30.610	27.492
H	29.297	31.342	26.878
